# Factors modulating the assembly of human β_2_-adrenergic receptor–β-arrestin complexes

**DOI:** 10.1038/s41594-026-01842-3

**Published:** 2026-07-31

**Authors:** Florian M. Wilhelm, Kristyna Pluhackova, John Janetzko, Matthieu Masureel, Erin Marsh, Jesse Hudspeth, Wenzel Gaßner, Gebhard F. X. Schertler, Brian K. Kobilka, Daniel J. Müller

**Affiliations:** 1 Department of Biosystems Science and Engineering, ETH Zurich, Basel, Switzerland; 2 Stuttgart Center for Simulation Science, Cluster of Excellence EXC 2075, University of Stuttgart, Stuttgart, Germany; 3 Department of Molecular and Cellular Physiology, Stanford University School of Medicine, Stanford, CA USA; 4 Department of Biochemistry and Molecular Genetics, University of Colorado Anschutz Medical Campus, Aurora, CO USA; 5 Department of Pharmacology, University of Colorado Anschutz Medical Campus, Aurora, CO USA; 6 Department of Pharmaceutical Sciences, Skaggs School of Pharmacy, University of Colorado Anschutz Medical Campus, Aurora, CO USA; 7 BioFrontiers Institute, University of Colorado Boulder, Boulder, CO USA; 8 Paul Scherrer Institute PSI, Villigen, Switzerland; 9 Present Address: Department of Structural Biology, Genentech Inc., San Francisco, CA USA

**Keywords:** Single-molecule biophysics, Atomic force microscopy, G protein-coupled receptors, Phospholipids

## Abstract

β-arrestins, pivotal regulators of G protein-coupled receptor (GPCR) signaling, assemble with hundreds of GPCRs. How this assembly rises to functionally distinct complexes in which β-arrestin engages the GPCR tail, core or both, remains a central question. Here employing single-molecule force spectroscopy and molecular dynamics simulations, we monitor assembly of β_2_-adrenergic receptor (β_2_AR)–β-arrestin2 (βarr2) tail, core and tail–core complexes in phospholipid membranes and dissect their mechanical and kinetic stabilities. We show that βarr2 engages the phosphorylated β_2_AR carboxy-terminus (C-tail) within milliseconds, much faster than the active receptor core. In addition, the phospholipid membrane contributes substantially to complex stability, with phosphatidylinositol 4,5-bisphosphate (PIP_2_) modulating stability and conformation. While PIP_2_ stabilizes the β_2_AR–βarr2 core, it precludes βarr2 from concomitantly binding the phosphorylated β_2_AR C-tail. βarr2 activation and PIP_2_ strengthen βarr2–membrane association through insertion of the C-edge and finger loop. These findings establish PIP_2_, alongside ligand binding and receptor phosphorylation, as a central determinant of β_2_AR–βarr2 complex assembly, offering mechanistic insight into the regulation of GPCR signaling.

## Abstract (web summary)

Wilhelm, Pluhackova and colleagues show how ligand binding, receptor phosphorylation and membrane interactions, including PIP_2_, drive the assembly of human β_2_-adrenergic receptor–β-arrestin complexes, providing insight into structural mechanisms regulating GPCR signaling.

## Main

β-arrestins are remarkably versatile regulators of G protein-coupled receptor (GPCR) signaling. Two β-arrestins, β-arrestin1 (βarr1)^1^ and β-arrestin2 (βarr2)^2^, desensitize and internalize hundreds of different GPCRs^3^ with varying affinities^4,5^. Following agonist binding and phosphorylation by GPCR kinases (GRKs)^6^, β-arrestins are recruited to the GPCR, block G protein coupling^6^ and promote receptor internalization through clathrin-mediated endocytosis^7,8^. This recruitment requires β-arrestin activation^9,10^, classically described as the displacement of its autoinhibitory carboxy-terminus (henceforth, the C-tail) by the phosphorylated receptor C-tail, yielding a β-arrestin–GPCR tail-engaged complex (tail complex)^11–14^. Active β-arrestin can also engage the transmembrane core of the GPCR to form a fully engaged β-arrestin–GPCR tail–core complex^12,14–20^. Recent studies suggest that the core of the active GPCR can also trigger β-arrestin activation, implying a physiologically relevant core-only-engaged complex (core complex)^21,22^. β-arrestin can therefore assemble structurally and functionally distinct complexes with GPCRs by engaging different structural elements^14^, including C-tail, core or intracellular loops (ICLs), and by adopting different orientations relative to the receptor^23^. The distinct engagement modes of β-arrestins with GPCRs have different functional consequences: desensitization of GPCRs requires the assembly of a tail–core complex, whereas a tail complex suffices for receptor internalization^24–26^ and the various active conformations of β-arrestins elicit distinct downstream signaling^27,28^. Understanding how these GPCR-β-arrestin complexes assemble, and how their conformational variability is encoded, is therefore central to understanding β-arrestin-mediated GPCR signaling.

Assembly of GPCR-β-arrestin complexes and activation of signaling pathways are influenced by several factors including the ligand activating the receptor^29^, the phosphorylation pattern on the GPCR C-tail^30–32^ and the membrane composition^21,33–35^. Phospholipids in particular have emerged as key modulators of ligand affinity, receptor activity and coupling of GPCRs to G proteins, GRKs and β-arrestins^21,34,36–38^. For class A GPCRs, such as the β_2_-adrenergic receptor (β_2_AR), which engage β-arrestins only transiently^4,39,40^, phosphoinositides, notably phosphatidylinositol 4,5-bisphosphate (PIP_2_), are required for β-arrestin recruitment, desensitization and internalization^21,34^. Among both β-arrestin isoforms, βarr2 is the predominant isoform mediating these processes at β_2_AR^41,42^. PIP_2_ has further been proposed to retain β-arrestins at clathrin-coated structures even after class A GPCR dissociation, potentially allowing a GPCR to activate multiple β-arrestins^21^. PIP_2_ is also believed to stabilize an active-like conformation of β-arrestin, even in the absence of a GPCR^34^. Moreover, β-arrestin was shown to transiently associate with the plasma membrane before encountering a ligand-stimulated GPCR^33^, whereas β-arrestin mutants unable to bind PIP_2_ lose this association^33^.

The transient nature of class A GPCR–β-arrestin complexes has made their structural characterization difficult without stabilizing antigen-binding fragments (Fabs) or chimeric C termini^43^. Förster resonance energy transfer (FRET) studies have probed the dynamics of the β_2_AR–βarr2 tail–core complex^28,29,32^, and truncations of β_2_AR (ICL3, C-tail) or βarr2 (finger loop) have been used to isolate binding modes^21,24,33^. Yet, how β_2_AR, the prototypical class A GPCR, assembles with βarr2 has remained largely unresolved.

Here we systematically characterize the assembly and disassembly of β_2_AR–βarr2 tail, core and tail–core complexes, using an in vitro system of human βarr2 and β_2_AR embedded in multicomponent phospholipid membranes with and without PIP_2_. This system allows to dissect how βarr2 interacts with the phosphorylated β_2_AR C-tail, the active β_2_AR transmembrane core or both. Combining dynamic atomic force spectroscopy (AFM)-based single-molecule force spectroscopy (SMFS) with atomistic molecular dynamics (MD) simulations, we quantify the mechanical stability and free-energy landscape parameters of each complex. Our data reveal the different roles for the β_2_AR tail and core in complex assembly and show how the membrane environment sculpts the dynamic landscape of the β_2_AR–βarr2 complex.

## Results

### Monitoring spontaneous binding events of βarr2 to β_2_AR

To quantify binding events of βarr2 to agonist-bound and phosphorylated β_2_AR, we expressed and purified human β_2_AR from *Spodoptera frugiperda* cell line 9 (*Sf*9) insect cells and phosphorylated the epinephrine-bound β_2_AR in vitro with purified GRK5. The phosphorylated β_2_AR was reconstituted into liposomes made from 1,2-dioleoyl-*sn*-glycero-3-phosphocholine (DOPC), 1,2-dioleoyl-*sn*-glycero-3-phosphoglycerol (DOPG) and cholesterol (Supplementary Fig. 1). Although native membranes contain a far wider lipid repertoire distributed asymmetrically between leaflets, our liposomes capture the main plasma membrane components at native concentrations^44^, while allowing variation of composition to study its impact on GPCR–β-arrestin complex assembly. Reconstituted β_2_AR retained function in radioligand binding assays (Supplementary Fig. 1c). These proteoliposomes were adsorbed onto freshly cleaved atomically flat mica and imaged by AFM in buffer solution (Supplementary Fig. 1d,e). The high-resolution AFM topographs show that the proteoliposomes opened into flat lipid membranes from which single or grouped β_2_ARs protruded. Combining AFM with confocal microscopy, we confirmed that these β_2_ARs could be activated with the full agonist BI-167107 (BI), as evidenced by binding of a fluorescently labeled nanobody specific to the intracellular surface of active β_2_AR (Nanobody 6B9 (Nb6B9))^45^ (Supplementary Fig. 2). Unlike endogenous catecholamine agonists, BI is a high-affinity full agonist stable for many hours^46^, which facilitated the detection of β_2_AR–Nb6B9 and, subsequently, β_2_AR–βarr2 complexes. No binding of Nb6B9 was detected for β_2_ARs bound to the inverse agonist ICI-118551 (ICI). Together, these data show that β_2_AR-containing liposomes can be adsorbed onto mica with preserved receptor function and with the cytoplasmic surface accessible to effectors.

**Fig. 1:**
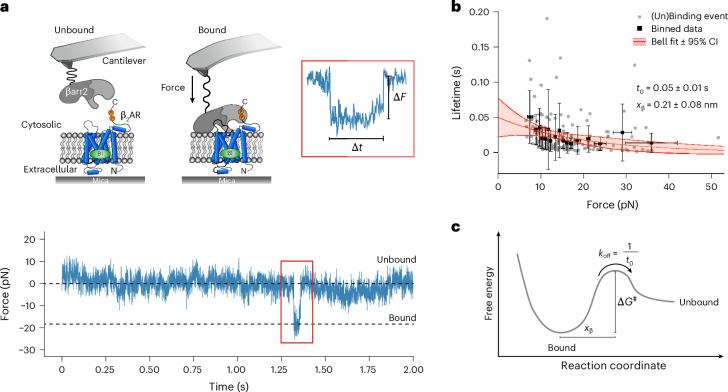
Monitoring spontaneous binding events between membrane-embedded β_2_AR and single human βarr2. **a**, AFM-based SMFS in the constant height-clamp mode. Phospholipid vesicles embedding phosphorylated and activated β_2_AR are adsorbed on mica where they open and form supported β_2_AR-containing lipid membranes (Supplementary Fig. 1). βarr2 is tethered to the tip of an AFM cantilever through a flexible PEG_27_-linker (Supplementary Fig. 3). The functionalized tip is brought into proximity of the β_2_AR-containing membrane where it is kept at distance (height) of roughly 4–8 nm. When β_2_AR binds βarr2, the cantilever detects an adhesive force ∆*F* for the duration ∆*t* of the binding event, which is recorded in FT curves, as exemplified below. The red frame enlarges a single binding event of βarr2 to β_2_AR. Phospholipids are colored gray, β_2_AR blue, phosphoserines pS355/pS356 orange and full agonist BI green. **b**, Force and lifetime of 165 single binding events of β_2_AR to βarr2 observed in 8305 individual FT curves obtained with 21 cantilevers from five independent functionalizations. Data were binned (black squares, mean ± s.d.) and fit with the Bell model^47^ (red line ± 95% confidence interval (CI, red shaded area)) to obtain an average lifetime *t*_0_ of 0.05 ± 0.01 s (mean ± s.d.) of the β_2_AR–βarr2 complex at equilibrium, that is, in the absence of externally applied force, and *x*_β_ of 0.21 ± 0.08 nm (mean ± s.d.). **c**, Free-energy landscape model and parameters describing the bound and unbound β_2_AR–βarr2 complex. The lifetime *t*_0_ of the complex is the inverse of the transition rate *k*_off_. *x*_β_ describes the distance between the bound state and the transition state toward unbinding of the complex, also referred to as the width of the free-energy valley. ∆*G*^‡^ gives the height of the free-energy barrier separating bound from unbound state, also referred to as the activation or kinetic free-energy barrier. Source data

To detect single binding events to activated β_2_AR, recombinant βarr2 bearing a single cysteine residue at position S267 was covalently linked to the tip of the AFM cantilever through a flexible polyethylene glycol (PEG_27_)-linker using maleimide chemistry (Supplementary Fig. 3). After activating β_2_AR with BI, the βarr2-functionalized tip was positioned roughly 4–8 nm above the β_2_AR membrane and kept at constant height while the force acting on the cantilever was recorded in a force–time (FT) curve (Fig. 1a). Spontaneous binding events between βarr2 and β_2_AR were detected as transient attractive forces. The binding rate (binding events per total number of FT curves) was 2.0% on β_2_AR-containing membranes versus 0.1% on receptor-free membranes, indicating that most detected events correspond to the spontaneous binding of βarr2 to β_2_AR (Supplementary Fig. 4a,b). The duration Δ*t* and force Δ*F* of all specific binding events were binned and fit to the Bell model^47^ to estimate the average lifetime *t*_0_ = 0.05 s ± 0.01 s (mean ± standard deviation (s.d.)) of the binding between βarr2 and β_2_AR at equilibrium, that is, in the absence of externally applied force (Fig. 1b). The distance *x*_β_ between the bound state and transition state to unbinding, which approximates the width of the free-energy valley stabilizing the complex (Fig. 1c), was 0.21 ± 0.08 nm (mean ± s.d.).

Further inspection of the FT curves recording binding events revealed that around 30% detected multiple binding events, typically separated by <100 ms (Supplementary Fig. 4a,c). Even when successive binding events occurred within <50 ms, which is below the average lifetime of the β_2_AR–βarr2 interaction, the duration and force of the second binding event were unaffected by the first (Supplementary Fig. 4d,e), indicating that the binding events were independent. No multiple binding steps occurred within single binding events (Supplementary Fig. 4f,g). We therefore conclude that the assay detected the (un-)binding of a single βarr2 to β_2_AR.

The results demonstrate that our setup can quantify the force, lifetime and width of the free-energy valley from single βarr2-β_2_AR binding events. However, our analysis only accurately describes single-step binding. If this binding occurs through multiple steps, the mechanical load applied by the cantilever to βarr2 on engaging β_2_AR may interfere with subsequent binding and assembly steps of the β_2_AR–βarr2 complex. To test whether such intermediate binding steps exist, we next applied force–distance (FD)-curve based AFM^48^.

### βarr2 binds the phosphorylated C-tail of β_2_AR in milliseconds

To characterize the assembly of β_2_AR–βarr2 complexes, we coadsorbed DOPC:DOPG:cholesterol membranes containing active, phosphorylated β_2_AR with purple membrane (control) onto mica and imaged the sample using FD-based AFM (Supplementary Fig. 5). For each pixel of the high-resolution topograph, the cantilever tip was approached to and retracted from the sample while FT and FD curves were recorded (Supplementary Fig. 6). In each approach and retraction cycle, the cantilever tip contacted the membrane surface for the shortest technically achievable average time of 21 ± 2 ms (mean ± standard error of the mean (s.e.m.)), during which the molecules covalently tethered to it could interact with the membrane. This enabled the characterization of distinct assembly steps of β_2_AR–βarr2 complexes at thermal equilibrium^48^. AFM tips functionalized with PEG_27_-linkers alone produced no specific adhesion events on β_2_AR membranes or purple membranes. On coupling βarr2 to the PEG_27_-linkers, specific (un-)binding events were detected exclusively on β_2_AR membranes and colocalized with β_2_AR in the topograph, confirming that βarr2 tethered to the AFM tip engages active β_2_AR specifically.

**Extended Data Fig. 1:**
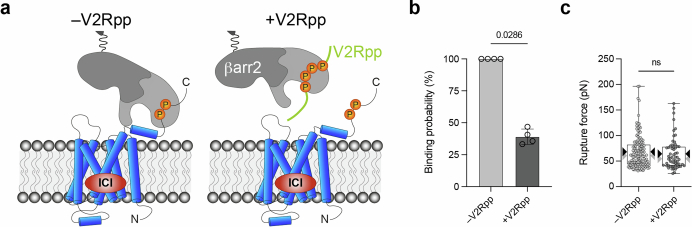
βarr2 specifically binds to the phosphorylated carboxy-terminal tail of β_2_AR in the DFS setup. **a**, Cartoon showing the competition of the phosphopeptide V2Rpp with the phosphorylated carboxy-terminal tail (C-tail) of β_2_AR for βarr2 binding in the DFS setup. β_2_AR is shown in blue, phosphates (P) in orange, and phospholipids as grey circles. βarr2 is tethered to the cantilever tip by a PEG27-linker (black). Inhibition of β_2_AR by the inverse agonist ICI (red) ensures that βarr2 can bind only to the phosphorylated C-tail of β_2_AR (left). At saturating concentrations of V2Rpp (right), βarr2 preferentially binds to the phosphopeptide and no longer interacts with β_2_AR. **b**, Binding probability of all specific (un-)binding events measured between βarr2-functionalized cantilever tips and β_2_AR reconstituted in DOPC:DOPG:cholesterol using FD-curve-based AFM at a retraction speed of 1090 nm s^−1^ and a tip-sample interaction time of ≈ 16 ms. 20 measurements (FD-curve-based topographs) without V2Rpp (–V2Rpp) and 21 measurements with V2Rpp (+V2Rpp) were conducted using 10 different AFM cantilevers from four independent functionalizations (data points). The binding probability was calculated for each βarr2-functionalized cantilever tip as the number of adhesion events per number of attempts (approach and retraction cycle of cantilever tip and sample). The means of –V2Rpp data were normalized to 100%. The binding probabilities of measurements +V2Rpp are shown relative to –V2Rpp. Bars show the mean and error bars the SD. **c**, Rupture force distribution of all specific (un-)binding events. Boxes mark the 25th to 75th percentiles, whiskers show minimum and maximum values, and median and mean are indicated by grey and black arrows. Rupture forces were extracted from measurements analyzed in **b**. *P* values were calculated using a Mann-Whitney test. Source data

We next used FD-based AFM to probe the timing and specificity of βarr2 binding to the phosphorylated β_2_AR C-tail (Extended Data Fig. 1a). To prevent βarr2 from engaging the cytoplasmic binding pocket of the β_2_AR core, the phosphorylated β_2_AR was inactivated with ICI. Keeping the interaction time at the minimum of 16 ± 3 ms (mean ± s.d.), we measured the binding probability between βarr2 and inactivated, phosphorylated β_2_AR. To verify that the binding events reflect βarr2 engaging the phosphorylated β_2_AR C-tail, the experiment was repeated in the presence of a phosphopeptide mimicking the phosphorylated C-tail of the human vasopressin 2 receptor (V2Rpp) (Extended Data Fig. 1b). V2Rpp reduced the binding probability by 61 ± 6% (mean ± s.d.), with the remaining 39% reflecting interactions of V2Rpp-activated βarr2 with the membrane. The rupture force observed on mechanically separating βarr2 from β_2_AR remained unchanged by V2Rpp (Extended Data Fig. 1c), thus suggesting that interactions of βarr2 with the phosphorylated β_2_AR C-tail and activated βarr2 with the membrane are of similar strength. Together, these experiments show that βarr2 engages the phosphorylated C-tail of β_2_AR rapidly, within milliseconds, once brought into close proximity with the receptor.

### Initial β_2_AR–βarr2 complexes assemble within tens of milliseconds

**Fig. 2:**
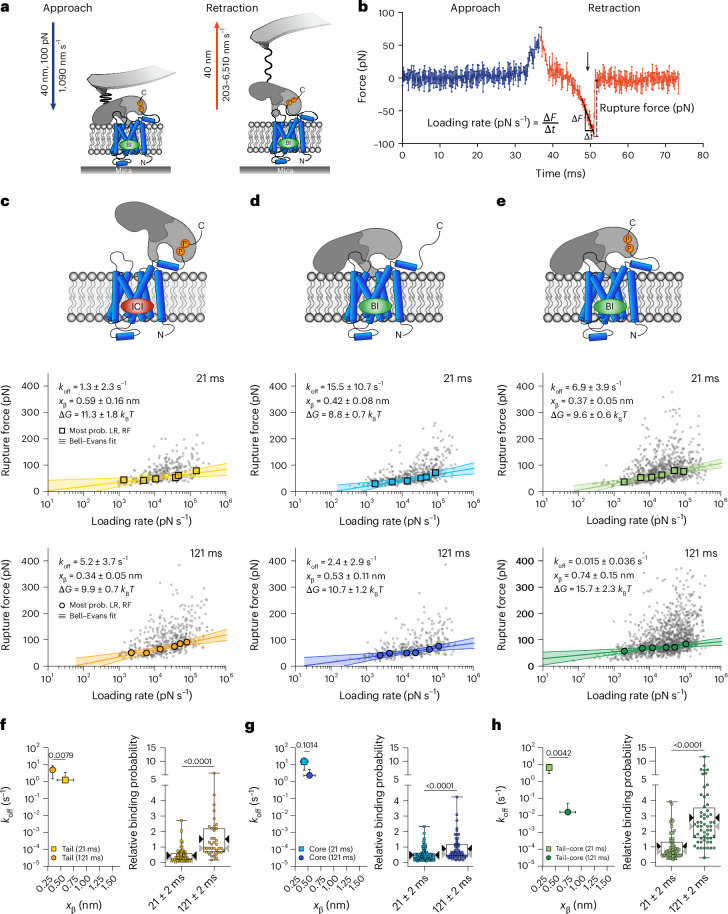
Mechanical and kinetic stability of β_2_AR–βarr2 tail, core and tail–core complexes in DOPC:DOPG:cholesterol membranes. **a**, DFS extracts mechanical and kinetic properties of β_2_AR–βarr2 interactions. While imaging β_2_AR-containing membranes, the βarr2-functionalized tip of the cantilever approaches the sample for every pixel of the resulting AFM topograph (Supplementary Fig. 6a). After tip-sample interaction times of 21 ms or 121 ms, the cantilever is retracted at 6 different speeds. Phospholipids are colored gray, β_2_AR blue, phosphoserines orange and BI in green. **b**, Representative FT curve recorded during approach (blue) and retraction (red) of the βarr2-functionalized tip detecting a specific, single (un-)binding event from β_2_AR (arrow). The rupture force of β_2_AR–βarr2 interactions and the loading rate being extracted as indicated. Shown are data points connected by lines. **c**–**e**, Cartoon (top) and (un-)binding forces (middle and bottom) of β_2_AR–βarr2 tail (**c**), core (**d**) and tail–core (**e**) complexes. LR, loading rate; RF, rupture force. To promote the tail complex, β_2_AR was inactivated by the inverse agonist ICI (red) and phosphorylated by GRK5. The core complex was obtained through BI-activation of unphosphorylated β_2_AR. The tail–core complex was promoted through BI-activation of GRK5-phosphorylated β_2_AR. DFS data were recorded at interaction times of 21 ms (middle) and 121 ms (bottom) for each retraction speed (Methods). The Bell–Evans model^67^ fit of the binned data (squares and circles) is shown in orange (tail), blue (core) and green (tail–core) as mean (line) ± 95% CI (shades). *k*_off_, *x*_β_ and ∆*G*^‡^ values are shown as mean ± s.d. **f**–**h**, *k*_off_, *x*_β_ and binding probabilities (prob.) of β_2_AR–βarr2 tail (**f**), core (**g**) and tail–core (**h**) complexes at 21 ms (squares) and 121 ms (circles). *k*_off_ and *x*_β_ values are shown as mean ± s.d. *P* values comparing *k*_off_ and *x*_β_ values are calculated applying the extra sum-of-squares *F* test to Bell–Evans fits of the DFS data. Binding probabilities are shown as boxplots where boxes mark the 25th to 75th percentiles, whiskers show minimum and maximum values, and arrows indicate median (gray) and mean (black). Data points represent individual replicates. Data collected at 21 ms interaction time comprise 65 (tail), 102 (core) and 60 (tail–core) replicates recorded with 22, 24 and 19 different cantilevers from 7, 7 and 7 independent functionalizations, respectively. Data collected at 121 ms interaction time comprise 38, 58 and 56 replicates recorded with 13, 13 and 11 different cantilevers from 3, 6 and 4 independent functionalizations, respectively. Binding probabilities are normalized to the mean of the tail–core complex at 21 ms. *P* values comparing binding probabilities are obtained from two-sided Mann–Whitney tests. Source data

Having observed binding of βarr2 to the phosphorylated β_2_AR C-tail within milliseconds, we applied dynamic SMFS (DFS) to quantify the mechanical and kinetic properties of this interaction (Fig. 2a,b). Specific (un-)binding forces of β_2_AR–βarr2 complexes were recorded over a wide range of loading rates (force per time) by FD-based AFM, with the interaction time between the βarr2-functionalized tip and the β_2_AR membrane kept at roughly 21 ms (Methods). For the β_2_AR–βarr2 tail complex, formed by βarr2 bound to phosphorylated β_2_AR inactivated by the inverse agonist ICI, the DFS data yielded a transition rate *k*_off_ of 1.3 ± 2.3 s^−1^ (mean ± s.d.), a free-energy valley width *x*_β_ of 0.59 ± 0.16 nm (mean ± s.d.) and a kinetic free-energy barrier Δ*G*^‡^ of 11.3 ± 1.8 *k*_B_*T* (mean ± s.d.)^48,49^ (Fig. 2c).

For the β_2_AR–βarr2 core complex, formed by BI-activated, unphosphorylated β_2_AR so that βarr2 engages the active cytoplasmic cavity of the receptor (core) without phosphate-mediated tail contacts, we obtained *k*_off_ = 15.5 ± 10.7 s^−1^, *x*_β_ = 0.42 ± 0.08 nm and Δ*G*^‡^ = 8.8 ± 0.7 *k*_B_*T* (Fig. 2d). Statistically, the parameters measured for tail and core complexes were indistinguishable (Supplementary Fig. 7a). Finally, BI-activated, phosphorylated β_2_AR was used to probe the β_2_AR–βarr2 tail–core complex, which yielded *k*_off_ = 6.9 ± 3.9 s^−1^, *x*_β_ = 0.37 ± 0.05 nm and Δ*G*^‡^ = 9.6 ± 0.6 *k*_B_*T*, considerably different from the core complex but similar to the tail complex (Fig. 2e and Supplementary Fig. 7b,c).

The 21 ms interaction time between the βarr2-functionalized tip and β_2_AR membrane was shorter than the roughly 50-ms lifetime measured in height-clamp experiments (Fig. 1) and the roughly 22–50-s lifetimes reported by FRET in cells^28,29,32^, so the complexes detected here likely reflect initial engagement. Because the parameters describing the β_2_AR–βarr2 tail–core complex lie between those measured for the tail and core complexes, our data suggest that within the first 21 ms βarr2 engages either the phosphorylated C-tail or the active core of β_2_AR, yielding a mixed population of βarr2–β_2_AR complexes.

### Fully engaged β_2_AR–βarr2 complexes take about 100 ms to assemble

To test whether βarr2–β_2_AR tail, core or tail–core complexes mature over time, we increased the interaction time to 121 ± 2 ms. The mechanical and kinetic properties of the tail and tail–core complexes changed considerably. The β_2_AR–βarr2 tail complex destabilized kinetically with *k*_off_ increasing fourfold to 5.2 ± 3.7 s^−1^ and *x*_β_ narrowing to 0.34 ± 0.05 nm, indicating reduced conformational variability, while Δ*G*^‡^ dropped 1.1-fold to 9.9 ± 0.7 *k*_B_*T* (Fig. 2c,f and Supplementary Fig. 7d). Nonetheless, the relative binding probability of βarr2 to β_2_AR (Methods) increased 3.5-fold from 0.43 ± 0.06 (mean ± s.e.m.) to 1.48 ± 0.20. The β_2_AR–βarr2 core complex stabilized only marginally over time, with *k*_off_ = 2.4 ± 2.9 s^−1^, *x*_β_ = 0.53 ± 0.11 nm and Δ*G*^‡^ = 10.7 ± 1.2 *k*_B_*T* (Fig. 2d,g and Supplementary Fig. 7e). The probability of βarr2 binding the β_2_AR core increased 1.8-fold from 0.46 ± 0.04 to 0.85 ± 0.10. By contrast, the β_2_AR–βarr2 tail–core complex kinetically stabilized markedly within 121 ms and *k*_off_ dropped roughly 500-fold to 0.015 ± 0.036 s^−1^ (Fig. 2e,h and Supplementary Fig. 7f), in good agreement with the 0.02–0.045 s^−1^ transition rates reported for βarr2–β_2_AR interactions in cells^28,29,32^. In addition, *x*_β_ doubled to 0.74 ± 0.15 nm, indicating increased conformational variability and Δ*G*^‡^ increased 1.6-fold to 15.7 ± 2.3 *k*_B_*T*, which was the highest free-energy barrier observed among all β_2_AR–βarr2 complexes characterized here. The relative binding probability of βarr2 to β_2_AR increased 2.9-fold to 2.9 ± 0.3, being the highest among the three β_2_AR–βarr2 complexes.

**Extended Data Fig. 2:**
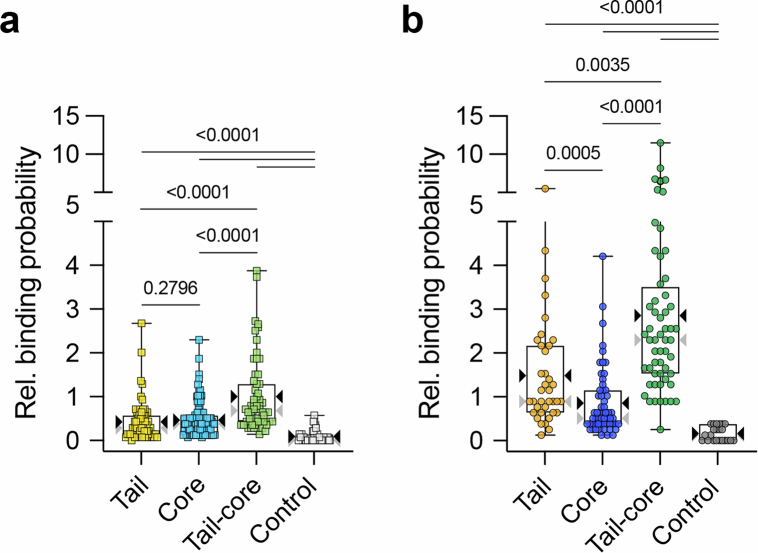
Binding probabilities of βarr2 to activated and phosphorylated β2AR compared to inactivated and unphosphorylated β_2_AR. **a** and **b**, Relative binding probabilities of βarr2 to β_2_AR in DOPC:DOPG:cholesterol membranes as revealed from DFS at (a) ≈ 21 ms and (b) ≈ 121 ms tip-sample interaction time. Binding probabilities were measured between βarr2 and ICI-inactivated, GRK5-phosphorylated β_2_AR (tail), BI-activated, unphosphorylated β_2_AR (core), and BI-activated, GRK5-phosphorylated β_2_AR (tail-core). Inactivated, unphosphorylated β_2_AR (control) served as a control for non-specific interactions of βarr2 with β_2_AR. Binding probabilities are shown as boxplots where boxes mark the 25th to 75th percentiles, whiskers show minimum and maximum values, and median and mean are indicated by grey and black arrows, respectively. Data are normalized to the mean of the binding probability of the β_2_AR-βarr2 tail-core complex measured at ≈ 21 ms interaction time. *P* values, indicated on the top, were calculated by applying an unpaired, nonparametric Mann-Whitney test. Data at ≈ 21 ms were obtained with 22 (tail), 24 (core), 19 (tail-core), and 9 (control) different cantilevers from 7 (tail, core, tail-core) and 3 (control) independent functionalizations. Data at ≈ 121 ms were obtained with 13 (tail), 13 (core), 11 (tail-core), and 13 (control) different cantilevers from 3 (tail), 6 (core), 4 (tail-core), and 3 (control) independent functionalizations. Source data

To confirm the specificity of βarr2 binding to the phosphorylated tail and/or the active core of β_2_AR, we measured the binding probability of βarr2 to unphosphorylated, ICI-bound β_2_AR at both interaction times (Extended Data Fig. 2). The low relative binding probabilities of 0.10 ± 0.05 at 21 ms and of 0.16 ± 0.04 at 121 ms suggest that βarr2 does not engage inactive, unphosphorylated β_2_AR. The few binding events observed in this control did not allow for robust fitting of the data and extraction of mechanical and kinetic parameters and are therefore considered nonspecific.

Together, these results show that within 21 ms βarr2 can assemble stable complexes with the phosphorylated C-tail of β_2_AR, while concurrent binding of βarr2 to the tail and core of β_2_AR or to the core alone yields β_2_AR–βarr2 complexes with mechanical and kinetic properties similar to the β_2_AR–βarr2 tail complex. At a 121-ms interaction time, the tail complex becomes less stable than the core complex, which only marginally stabilizes (Supplementary Fig. 7g), and the substantially stabilized tail–core complex represents a fully engaged β_2_AR–βarr2 assembly distinct from either the tail or core complex (Supplementary Fig. 7h,i).

### PIP_2_ promotes βarr2 binding to transmembrane core of β_2_AR

**Fig. 3:**
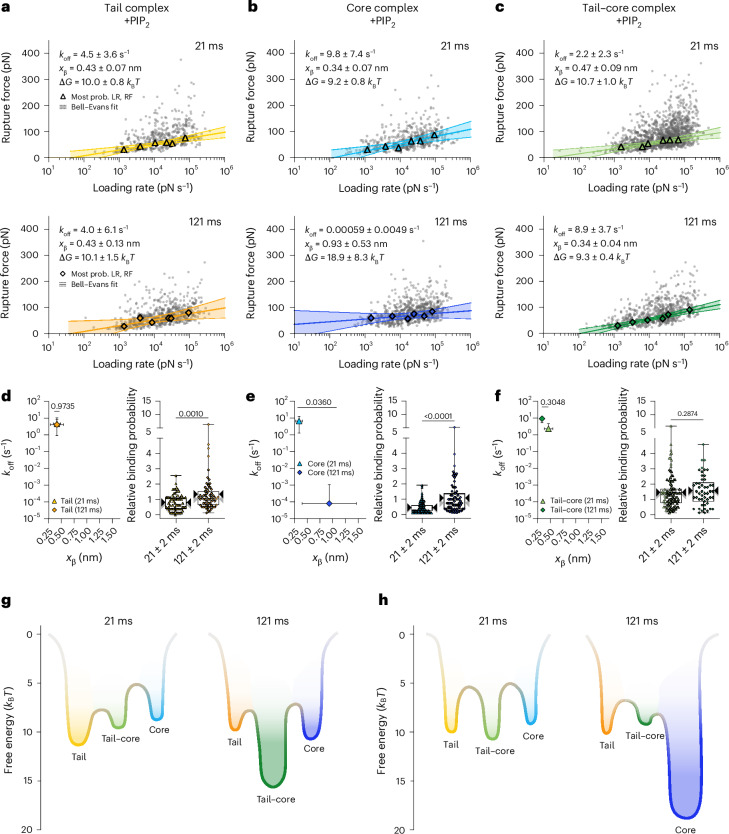
PIP_2_ promotes the assembly of the β_2_AR–βarr2 core complex. **a**–**c**, DFS data of β_2_AR–βarr2 tail (**a**), core (**b**) and tail–core (**c**) complexes in DOPC:DOPG:cholesterol:PIP_2_ membranes detected at 21 ms (top) and 121 ms (bottom) interaction times. DFS data are binned (21 ms triangle, 121 ms diamond) for each retraction speed (Methods). The Bell–Evans model^67^ fit of the binned data is shown in orange (tail), blue (core) and green (tail–core) as mean (line) ± 95% CI (shaded area). **d**–**f**, *k*_off_, *x*_β_ and binding probabilities of β_2_AR–βarr2 tail (**d**), core (**e**) and tail–core (**f**) complexes at 21 ms (triangles) and 121 ms (diamonds) in the presence of PIP_2_. *k*_off_, *x*_β_ and ∆*G*^‡^ values are shown as mean ± s.d. *P* values comparing *k*_off_ and *x*_β_ values are calculated applying the extra sum-of-squares *F* test to Bell–Evans fits. Binding probabilities are shown as boxplots where boxes mark the 25th to 75th percentiles, whiskers show minimum and maximum values, and arrows indicate median (gray) and mean (black). Data points represent the probability of βarr2 binding to β_2_AR in every replicate. Data collected at 21 ms interaction time comprise 68 (tail), 117 (core) and 109 (tail–core) replicates recorded with 16, 21 and 18 different cantilevers from 6, 6 and 6 independent functionalizations, respectively. Data collected at 121 ms interaction time comprise 59, 71 and 50 replicates recorded with 15, 17 and 11 different cantilevers from 8, 6 and 6 independent functionalizations, respectively. Binding probabilities are normalized to the mean of the tail–core complex at 21 ms in the absence of PIP_2_ (Fig. 2h). *P* values comparing binding probabilities are obtained from two-sided Mann–Whitney tests. **g**,**h**, Schematic free-energy landscapes depicting an early (21 ms) and a late (121 ms) timepoint of the binding of βarr2 to β_2_AR reconstituted in a membrane without (**g**) and with (**h**) PIP_2_. Free-energy minima roughly reflect ∆*G*^‡^ values obtained from DFS. Source data

**Extended Data Fig. 3:**
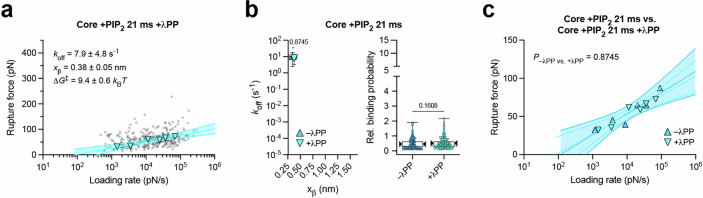
Lambda protein phosphatase-treatment of β_2_AR does not affect binding of βarr2 to the β_2_AR core. **a**, DFS data characterizing the binding of βarr2 to lambda protein phosphatase (λPP)-treated, BI-activated β_2_AR at ≈ 21 ms interaction time in the presence of PIP2. Single (un-)binding events are shown as grey dots. DFS data are binned (down-triangles) for each retraction speed with the most probable loading rate and rupture force obtained by kernel density estimation (Methods). The Bell-Evans model^68^ fit to the binned data is shown in cyan as mean (line) ± 95% CI (shaded area). DFS data represent 87 replicates obtained with 18 different cantilevers from 3 independent functionalizations. *k*_off_, *x*_β_, and Δ*G*^‡^ values are given as mean ± SD. **b**, Comparison of *k*_off_ values, *x*_β_ values, and binding probabilities from β_2_AR-βarr2 core complexes measured in the presence of PIP_2_ at ≈ 21 ms with λPP-treated and untreated β_2_AR. Data is shown as mean with error bars showing SD. *P* values comparing *k*_off_ and *x*_β_ values are calculated by applying the extra sum-of-squares *F* test to the Bell-Evans fit of the DFS data (shown in c). Binding probabilities are shown as boxplots where boxes mark the 25th to 75th percentiles, whiskers show minimum and maximum values, and median and mean are indicated by grey and black arrows, respectively. Data points represent the probability of βarr2 binding to the β_2_AR in every replicate. Binding probability data are normalized to the mean of the tail-core complex at ≈ 21 ms interaction time in the absence of PIP_2_. *P* values comparing binding probabilities are obtained from Mann-Whitney tests. **c**, Statistical comparison of DFS data obtained from core complexes ± λPP in the presence of PIP_2_ at ≈ 21 ms. *P* values comparing the two conditions are calculated by applying the extra sum-of-squares *F* test to the Bell-Evans fit, shown as lines (mean) ± 95% CI (shaded area), of the binned DFS data. Data of core –λPP in **b** and **c** were taken from Fig. 3b,e. Source data

Given the importance of phosphoinositides for class A GPCRs, such as β_2_AR, to assemble with β-arrestins in cells, we tested the effect of adding PIP_2_ to our proteoliposomes^21,33,34^. Proteoliposomes composed of DOPC, DOPG, cholesterol, PIP_2_ and β_2_AR were adsorbed as before (Supplementary Fig. 1f,g) and the resulting β_2_AR–βarr2 complexes characterized by DFS (Fig. 3a–c and Supplementary Fig. 8a–i). At a 21-ms interaction time, the tail complex showed *k*_off_ = 4.5 ± 3.6 s^−1^, *x*_β_ = 0.43 ± 0.07 nm and Δ*G*^‡^ = 10.0 ± 0.8 *k*_B_*T*, the core complex *k*_off_ = 9.8 ± 7.4 s^−1^, *x*_β_ = 0.34 ± 0.07 nm and Δ*G*^‡^ = 9.2 ± 0.8 *k*_B_*T*, and the tail–core complex *k*_off_ = 2.2 ± 2.3 s^−1^, *x*_β_ = 0.47 ± 0.09 nm and Δ*G*^‡^ = 10.7 ± 1.0 *k*_B_*T*. The three β_2_AR–βarr2 complexes thus exhibited similar stabilities in PIP_2_ membranes. Because β_2_AR purified from insect cells shows low basal phosphorylation (Supplementary Fig. 1b), we ruled out an effect on our measurements by treating BI-activated β_2_AR with lambda protein phosphatase to remove the basal phosphorylation (Extended Data Fig. 3). The core complex formed between such treated BI-activated β_2_AR and βarr2 was indistinguishable from untreated samples.

Extending the interaction time to 121 ms left the β_2_AR–βarr2 tail complex essentially unchanged with *k*_off_ = 4.0 ± 6.1 s^−1^, *x*_β_ = 0.43 ± 0.13 nm and Δ*G*^‡^ = 10.1 ± 1.5 *k*_B_*T* (Fig. 3a,d and Supplementary Fig. 8d). However, the core complex stabilized considerably with *k*_off_ dropping >16,000-fold to 0.00059 ± 0.0049 s^−1^, *x*_β_ increased 2.7-fold to 0.93 ± 0.53 nm and Δ*G*^‡^ rose twofold to 18.9 ± 8.3 *k*_B_*T* (Fig. 3b,e and Supplementary Fig. 8e). The tail–core complex, by contrast, did not stabilize and its parameters *k*_off_ = 8.9 ± 3.7 s^−1^, *x*_β_ = 0.34 ± 0.04 nm and Δ*G*^‡^ = 9.3 ± 0.4 *k*_B_*T* remained similar to the tail–core complex at 21 ms and to the tail complex at 121 ms (Fig. 3c,f and Supplementary Fig. 8f,h). Only the core complex therefore stabilized with time in the presence of PIP_2_ (Supplementary Fig. 8d–f). At 121 ms the binding probability remained highest for the tail–core complex and increased 1.6-fold and 2.3-fold for tail and core complexes, respectively. These data suggest that PIP_2_ promotes the assembly of a stable β_2_AR–βarr2 core complex while suppressing the assembly of a stable tail–core complex. In fact, tail and tail–core complexes are essentially indistinguishable in lifetime and mechanical stability, suggesting that in PIP_2_ membranes βarr2 selectively engages the phosphorylated C-tail of β_2_AR on extending the interaction time to 121 ms.

**Extended Data Fig. 4:**
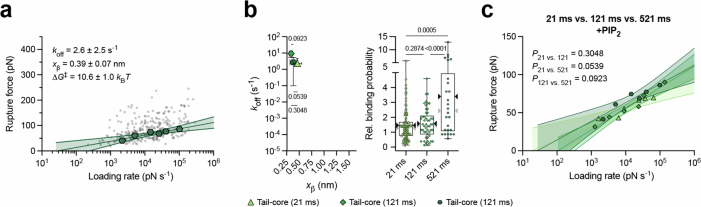
β_2_AR-βarr2 tail-core complex formation within ≈ 521 ms in DOPC:DOPG:cholesterol:PIP_2_ membranes. **a**, DFS data of β_2_AR-βarr2 tail-core complex detected at ≈ 521 ms interaction time in the presence of PIP_2_. Single (un-)binding events are shown as grey dots. DFS data are binned (hexagons) for each retraction speed with the most probable loading rate and rupture force obtained by kernel density estimation (Methods). The Bell-Evans model^2^ fit to the binned data is shown in dark green as mean (line) ± 95% CI (shaded area). *k*_off_, *x*_β_, and Δ*G*^‡^ values are given as mean ± SD. **b**, *k*_off_ values, *x*_β_ values, and binding probabilities from β_2_AR-βarr2 tail-core complexes measured by SMFS at interaction times of ≈ 21 ms (triangles), ≈ 121 ms (diamonds), and ≈ 521 ms (hexagons). Data is shown as mean with error bars showing SD. *P* values comparing koff and *x*_β_ values are calculated by applying the extra sum-of-squares *F* test to the Bell-Evans fit of the DFS data (shown in **c**). Binding probabilities are shown as boxplots where boxes mark the 25th to 75th percentiles, whiskers show minimum and maximum values, and median and mean are indicated by grey and black arrows, respectively. Data points represent the probability of βarr2 binding to the β_2_AR in every replicate. Data collected at 521 ms interaction time comprise 26 replicates recorded with 8 different AFM cantilevers from 2 independent functionalizations. Binding probability data are normalized to the mean of the tail-core complex at ≈ 21 ms interaction time in the absence of PIP_2_. *P* values comparing binding probabilities are obtained from Mann-Whitney tests. **c**, Comparison of DFS data of β_2_AR-βarr2 tail-core complex detected at ≈ 21 ms (triangles), ≈ 121 ms (diamonds), and ≈ 521 ms (hexagons) interaction time. *P* values comparing the three conditions are calculated by applying the extra sum-of-squares *F* test to the Bell-Evans fit, shown as lines (mean) ± 95% CI (shaded area), of the binned DFS data. Data of ≈ 21 ms and ≈ 121 ms in **b** and **c** were taken from Fig. 3c,f. Source data

To test whether the β_2_AR–βarr2 tail–core complex simply requires more time to stably assemble in PIP_2_ membranes, we extended the interaction time to 521 ± 2 ms (Extended Data Fig. 4). The complex did not substantially change its properties with *k*_off_ = 2.6 ± 2.5 s^−1^, *x*_β_ = 0.39 ± 0.07 nm and Δ*G*^‡^ = 10.6 ± 1.0 *k*_B_*T*, confirming that the tail–core complex does not stabilize further with time and remains indistinguishable from tail–core complexes at 21 and 121 ms. The binding probability, however, increased 2.8-fold relative to 121 ms.

**Extended Data Fig. 5:**
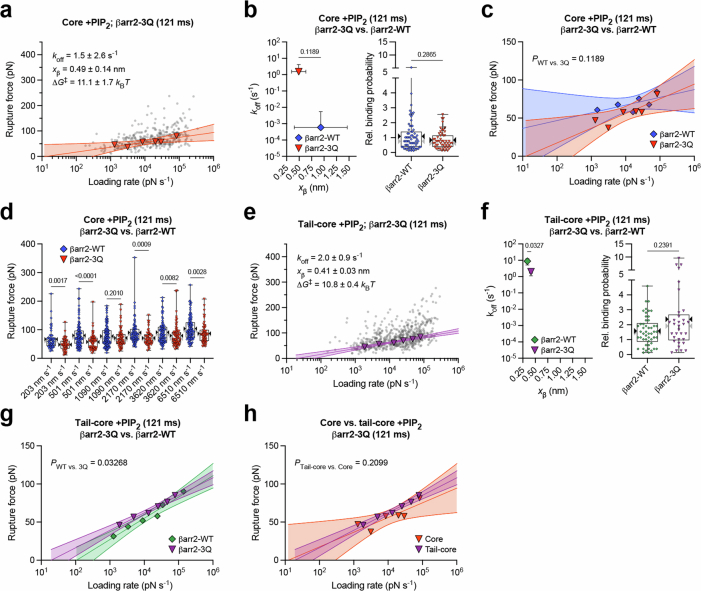
DFS measurements of core and tail-core complexes of βarr2-3Q mutant with β_2_AR in membranes with PIP_2_ at 121 ms interaction time. **a**, DFS data of β_2_AR-βarr2-3Q core complexes in the presence of PIP_2_ at 121 ms interaction time from 53 replicates recorded with 13 different AFM cantilevers from 4 independent functionalizations. DFS data are binned for each retraction speed (Methods). The Bell-Evans model fit to the binned data is shown in red as mean (line) ± 95% CI (shaded area). **b**, Comparison of *k*_off_, *x*_β_, and binding probabilities of β_2_AR-βarr2-3Q and β_2_AR-βarr2-WT core complexes at 121 ms in the presence of PIP2. *k*_off_ and *x*_β_ values are shown as mean ± SD. *P* values comparing *k*_off_ and *x*_β_ values are calculated by applying the extra sum-of-squares *F* test to the Bell-Evans fits of the DFS data shown in **c**. **c**, Comparison of Bell-Evans fits to the DSF data of β_2_AR-βarr2-3Q and β_2_AR-βarr2-WT core complexes at 121 ms in the presence of PIP_2_. *P* values are calculated applying the extra sum-of-squares *F* test. The shaded area represents ± 95% CI. **d**, Comparison of rupture forces recorded at different retraction speeds of β_2_AR-βarr2-3Q (red triangles) and β_2_AR-βarr2-WT core complexes at 121 ms in the presence of PIP_2_. **e**, DFS data of β_2_AR-βarr2-3Q tail-core complexes in the presence of PIP_2_ at 121 ms interaction time from 33 replicates recorded with 6 different AFM cantilevers from 3 independent functionalizations. DFS data are binned for each retraction speed (Methods). The Bell-Evans model1 fit to the binned data is shown in purple as mean (line) ± 95% CI (shaded area). **f**, Comparison of *k*_off_, *x*_β_, and binding probabilities of β_2_AR-βarr2-3Q and β_2_AR-βarr2-WT tail-core complexes at 121 ms in the presence of PIP_2_. *k*_off_ and *x*_β_ values are shown as mean ± SD. *P* values comparing *k*_off_ and *x*_β_ values are calculated by applying the extra sum-of-squares *F* test to Bell-Evans fits of the DFS data shown in **g**. **g**, Comparison of Bell-Evans fits to the DFS data of β_2_AR-βarr2-3Q tail-core and β_2_AR-βarr2-WT tail-core complexes at 121 ms. *P* values are calculated applying the extra sum-of-squares *F* test. The shaded area represents ± 95% CI. **h**, Comparison of Bell-Evans fits to the DFS data of β_2_AR-βarr2-3Q core and tail-core complexes. *P* values are calculated by applying the extra sum-of-squares *F* test. The shaded area represents ± 95% CI. Binding probabilities and rupture forces in **b**, **d**, and **f** are shown as boxplots where boxes mark the 25th to 75th percentiles, whiskers show minimum and maximum values, and median (grey arrows) and mean (black arrows) are indicated. Binding probability data are normalized to the mean of the tail-core complex at 21 ms in the absence of PIP_2_ (Fig. 2h). *P* values comparing binding probabilities and rupture forces in **b**, **d**, and **f** are obtained from Mann-Whitney tests. Data collected with βarr2-WT are taken from Fig. 3b,e and Fig. 3c,f for the core and tail-core complex, respectively. Source data

As β-arrestins bind PIP_2_ through several positively charged residues mainly on the concave surface of its C-lobe^17,50^, we asked whether the specific interaction was required to stabilize the β_2_AR–βarr2 core complex. We repeated the DFS measurements of core and tail–core complexes at 121 ms of interaction time in PIP_2_ membranes using a PIP_2_-binding-deficient βarr2 mutant (K233Q, R237Q, K251Q; henceforth, βarr2-3Q)^51^^,52^ (Extended Data Fig. 5). This blunting of the PIP_2_-binding capability of βarr2 impairs βarr2 recruitment to β_2_AR^33,34^, translocation to clathrin-coated structures^21,33^ and β_2_AR internalization^50^. The β_2_AR–βarr2-3Q core complex yielded *k*_off_ = 1.5 ± 2.6 s^−1^, *x*_β_ = 0.49 ± 0.14 nm and Δ*G*^‡^ = 11.1 ± 1.7 *k*_B_*T* (Extended Data Fig. 5a). Compared with the wild-type β_2_AR–βarr2 (βarr2-WT) core complex *k*_off_ markedly increased and *x*_β_ decreased, indicating the β_2_AR–βarr2-3Q complex to be less stable, although the binding probabilities remained unchanged (Extended Data Fig. 5b). While fitting the Bell–Evans model to the DFS data obtained with βarr2-3Q and βarr2-WT core complexes showed no difference (Extended Data Fig. 5c), the rupture force required to separate β_2_AR–βarr2-3Q core complexes, however, was lower than for β_2_AR–βarr2-WT core complexes (Extended Data Fig. 5d), suggesting that βarr2-3Q engages the core of β_2_AR less stably than βarr2-WT. Conversely, in PIP_2_ membranes the β_2_AR–βarr2-3Q tail–core complex with *k*_off_ = 2.0 ± 0.9 s^−1^, *x*_β_ = 0.41 ± 0.03 nm and Δ*G*^‡^ = 10.8 ± 0.4 *k*_B_*T* (Extended Data Fig. 5e), increased stability compared with the β_2_AR–βarr2-WT tail–core complex (Extended Data Fig. 5f,g). The binding probability of the β_2_AR–βarr2-3Q tail–core complex did not change. In addition, the mechanical and kinetic properties of β_2_AR–βarr2-3Q core and tail–core complexes were no longer distinguishable (Extended Data Fig. 5h) as opposed to β_2_AR–βarr2-WT tail–core and β_2_AR–βarr2-WT core complexes, which showed clear differences from each other in the presence of PIP_2_ (Supplementary Fig. 8i). Taken together, these data show that stabilization of the core complex and destabilization of the tail–core complex depend on βarr2 binding PIP_2_ through its C-lobe.

In summary, the parameters quantified by DFS contour the free-energy landscape of the β_2_AR–βarr2 complex at the time of initial engagement and 100 ms later, without and with PIP_2_ in the membrane (Fig. 3g,h and Supplementary Fig. 8k–p). Without PIP_2_, βarr2 first forms a kinetically stable tail complex with the phosphorylated C-tail of β_2_AR that matures into a stable tail–core complex through engagement of the active receptor core. PIP_2_ levels the kinetic stabilities of early βarr2–β_2_AR complexes, which over time reorganize toward core-bound states of β_2_AR, whereas the tail and tail–core complexes do not stabilize further.

### PIP_2_ constrains the structural variability of β_2_AR–βarr2 complexes

**Fig. 4:**
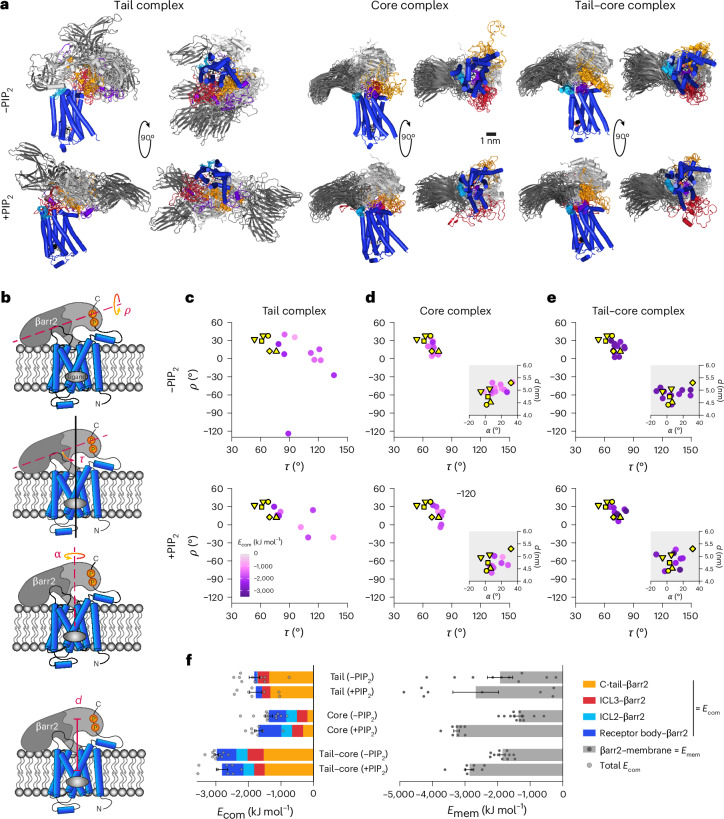
β_2_AR–βarr2 complexes reveal different structural variabilities and interaction energies. **a**, β_2_AR–βarr2 tail, core and tail–core complexes visualized from the side (left) and extracellular (right) perspective after >1 µs equilibration MD simulations performed in DOPC:DOPG:cholesterol membranes without (top) and with (bottom) PIP_2_. Lipids and solvent are not shown. β_2_AR is colored blue, ICL2 light blue, ICL3 red and carboxy-terminal tail (C-tail) orange. βarr2 is shown as a cartoon with the N-lobe colored light gray, C-lobe dark gray and finger loop purple. **b**, Orientation of βarr2 relative to β_2_AR and membrane. *ρ* describes the rotation of βarr2 along its long axis, *τ* the tilt of the long βarr2 axis relative to the membrane plane and *α* the rotation of βarr2 around the membrane normal centered at the receptor transmembrane helix bundle. *d* gives the distance between the centers of mass of the β_2_AR core and βarr2 N-lobe (Methods). *d* and *α* are evaluated only for complexes with arrestin inserted into the receptor core. **c**, *ρ* and *τ* of β_2_AR–βarr2 tail complexes in membranes without (top, *n* = 10) and with (bottom, *n* = 8) PIP_2_. **d**, *ρ*, *τ*, *α* and *d* of β_2_AR–βarr2 core complexes in membranes without (top, *n* = 12) and with (bottom, *n* = 8) PIP_2_. **e**, *ρ*, *τ*, *α* and *d* of β_2_AR–βarr2 tail–core complexes in membranes without (top, *n* = 11) and with (bottom, *n* = 8) PIP_2_. Data points in **c**–**e** are colored based on the interaction energy *E*_com_ between βarr2 and β_2_AR. For comparison, angles and *d* were estimated for the experimentally resolved structures of β_1_-adrenoceptor–formoterol–βarr1 (PDB 6TKO, yellow up-triangles)^12^, M2 muscarinic receptor–βarr1 (PDB 6U1N, squares)^54^, 5-HT2B/LSD–βarr1 (PDB 7SRS, diamonds)^16^, rhodopsin–arrestin-1 (PDB 5DGY, circles)^55^ and cannabinoid receptor1–βarr1 (PDB 8WU1^19^ and 8WRZ^20^, down triangles). **f**, Bar graphs of average interaction energies *E*_com_ and *E*_mem_ during the last 10 ns of MD simulations between β_2_AR–βarr2 (left) and βarr2–membrane (right) of different β_2_AR–βarr2 complexes. *E*_com_ is split into contributions of β_2_AR C-tail (orange), ICL2 (light blue), ICL3 (red) and receptor body (blue). Data points represent individual simulations. Error bars give the s.e.m. (*n* ≥ 8, see **c**–**e** for values). Source data

To interpret the measured interactions of βarr2, β_2_AR and the membrane, we performed atomistic MD simulations. β_2_AR–βarr2 tail, core and tail–core complexes were modeled against experimentally solved arrestin–GPCR structures, and against spontaneously assembled β_2_AR–βarr2 complexes in coarse-grained simulations^53^ using protein sequences and membrane compositions equivalent to our experiments (Fig. 4a). Although these starting configurations increase structural plausibility, they may bias sampling toward local free-energy minima (Methods). Therefore, in the absence of independent experimental validation, the resulting ensembles should be interpreted with appropriate caution. After simulation for at least 1 µs, the core and tail–core complexes in the absence and presence of PIP_2_ spanned the conformational variability previously observed for structures of βarr1–β_1_AR^12^, βarr1–M2R^54^, βarr1–5-HT2B^16^, arr1–Rho^55^ and βarr1–CB1^19,20^ (Fig. 4a–e). Compared with membranes lacking PIP_2_, in core complexes the presence of PIP_2_ constrained the distribution of the rotation angle *α* of βarr2 relative to the β_2_AR core by roughly 10° and βarr2 inserted roughly 0.5 nm deeper into the β_2_AR core. In tail–core complexes, PIP_2_ reduced the span of *α* by 5° and broadened the insertion depth. All core and tail–core complexes in membranes without or with PIP_2_ stably anchored the C-edge of βarr2 (represented by βarr2 residues L192, K227 and R332) to the membrane, in agreement with previous MD simulations of the C-domain of arr1^56^ (Supplementary Fig. 9). In tail complexes, the long, flexible β_2_AR C-tail, to which βarr2 bound at phosphorylated S355 and S356, enabled βarr2 to bind to the membrane. The C-tail flexibility allowed βarr2 to adopt diverse orientations relative to β_2_AR and membrane (Fig. 4a–c). From 10 simulations of tail complexes without PIP_2_, 4 showed C-edge binding to the membrane and 4 the finger loop (represented by βarr2 residues L69, D70, V71 and L72) bound to the membrane with interaction probabilities >70%. In two simulations, neither the C-edge nor the finger loop interacted with the membrane. Conversely, in all eight simulations of tail complexes with PIP_2_, βarr2 oriented the finger loop toward the membrane, which correlated with a reduced variance of the rotation angle *ρ*. In 6 of the 8 simulations the finger loop interacted with the membrane with binding probabilities >70% (Supplementary Fig. 9). The C-edge of βarr2 bound to the membrane in four out of eight tail complexes in the presence of PIP_2_, as reflected also by the variability of *τ*. Taken together, our simulations show that PIP_2_ constrains the orientation of βarr2 relative to receptor and membrane, preferentially positions the finger loop of βarr2 toward the membrane and shapes the conformation of β_2_AR–βarr2 complexes.

### PIP_2_-dependent membrane stabilization of β_2_AR–βarr2 complexes

Next, we examined the interaction energy *E*_com_ between βarr2 and β_2_AR and the interaction energy *E*_mem_ stabilizing βarr2 in tail, core and tail–core complexes (Fig. 4f). *E*_com_ was insensitive to PIP_2_ and was the smallest for β_2_AR–βarr2 core complexes, slightly higher for tail complexes and twofold higher for tail–core than for core complexes. In tail and tail–core complexes the phosphorylated C-tail dominated *E*_com_. In core complexes, the receptor body and the intracellular loop 2 (ICL2) together contributed >50% to *E*_com_. In all complexes, ICL3 substantially contributed to stabilizing the β_2_AR–βarr2 interaction. While *E*_com_ was largely unaffected by PIP_2_, *E*_mem_ markedly increased in its presence for every complex. Without PIP_2_, *E*_mem_ and *E*_com_ were comparable for tail and core complexes and *E*_mem_ was smaller than *E*_com_ for tail–core complexes. PIP_2_ doubled *E*_mem_ for core complexes, making the membrane the dominant stabilizing factor, and rendered *E*_mem_ the largest contribution to the total interaction energy (*E*_tot_ = *E*_com_ + *E*_mem_) in tail complexes and 50% of *E*_tot_ in tail–core complexes. Absolute interaction energies from MD trajectories are qualitative, force-field-dependent measures of intermolecular stabilization^57^ and should not be interpreted as binding free energies. Considering this caveat, our simulations indicate that the membrane substantially contributes to the interaction energy stabilizing all β_2_AR–βarr2 complexes, and that PIP_2_ selectively amplifies this membrane-mediated stabilization relative to other anionic lipids.

### PIP_2_ draws ICL3 of β_2_AR to the membrane without activating β_2_AR

**Extended Data Fig. 6:**
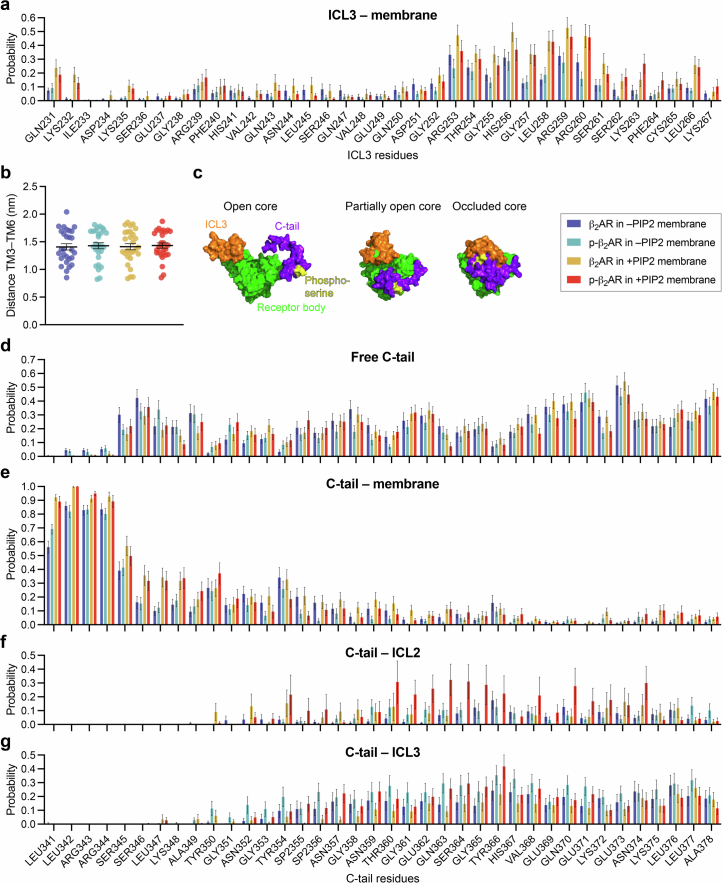
MD simulations of unphosphorylated and phosphorylated β_2_AR in membranes without and with PIP_2_. **a**, Interaction probability of β_2_AR intracellular loop 3 (ICL3) residues with phospholipids of the membrane when β_2_AR is unphosphorylated or phosphorylated (p-β_2_AR) and embedded in a membrane without PIP_2_ (–PIP_2_) and unphosphorylated or phosphorylated in a membrane with PIP_2_ (+PIP_2_). For color codes see subfigure **c**. **b**, Distance between transmembrane helix 3 (TM3) and TM6 of β_2_AR simulated in **a**. Bars indicate mean and error bars s.e.m. **c**, Molecular surfaces from the cytoplasmic view of β_2_AR with an open, partially open, and occluded core. Receptor body colored green, ICL3 orange, C-tail purple, and phosphoserines on C-tail yellow. **d**, Probability of β_2_AR C-tail residues being free, that is, not interacting with any lipids or other receptor residues. Zero probability indicates that a residue is always interacting with lipids or other receptor residues. **e-g**, Interaction probability of **e** β_2_AR C-tail residues with phospholipids of the membrane, **f** β_2_AR C-tail residues with the ICL2, and **g** β_2_AR C-tail residues with ICL3. Bar graphs in **a,d-g** show the mean ± s.e.m. of 28 simulations for every condition. β_**2**_AR was equilibrated for 550 ns, and the mean (interaction) probability was calculated for the following 100 ns of simulation. Source data

PIP_2_ has been shown to stabilize active GPCRs conformations^37,57,58^ and modulate G protein recruitment and selectivity^37,58^. Thus, we conducted 4 × 28 atomistic MD simulations, 650 ns each, of agonist-bound unphosphorylated and phosphorylated β_2_AR in membranes with and without PIP_2_ (Extended Data Fig. 6). In the presence of PIP_2_, ICL3 of β_2_AR bound more frequently to membrane phospholipids (Extended Data Fig. 6a), consistent with ref. ^57^, in which it was reported that PIP_2_ specifically draws ICL3 to the membrane and thereby promotes an open conformation of the β_2_AR core. However, the transmembrane helix 3 (TM3) and TM6 distance distribution, a hallmark of receptor activation, was indistinguishable across all four simulated conditions (Extended Data Fig. 6b), indicating that PIP_2_ alone does not influence β_2_AR opening under our conditions. This apparent discrepancy may reflect the higher PIP_2_ concentration used in ref. ^57^ (10 mol% PIP_2_, well above physiological levels^59,60^) compared with the 3 mol% used here or their use of a β_2_AR construct lacking the C-tail.

Further, a recent study^11^ proposed an autoinhibitory role of the β_2_AR C-tail in modulating β_2_AR–βarr2 coupling. To address this, we analyzed the interaction probabilities of β_2_AR C-tail residues with β_2_AR and the membrane in above simulations (Extended Data Fig. 6c–g). Across all conditions, the receptor core appeared fully open, partially occluded or fully occluded by the C-tail (Extended Data Fig. 6c), but quantification of the per-residue binding probabilities revealed few differences between conditions. The probability of a free C-tail, showing no interactions with phospholipids or β_2_AR, did not differ among phosphorylated and unphosphorylated β_2_AR in membranes without or with PIP_2_ (Extended Data Fig. 6d), nor did its interaction probability with phospholipids (Extended Data Fig. 6e). C-tail binding to ICL2 increased by around 10% when β_2_AR was phosphorylated and PIP_2_ was present (Extended Data Fig. 6f). Although such binding can occlude the β_2_AR and reduce accessibility to βarr2, the absolute probability remains low. C-tail–ICL3 interactions were slightly elevated upon C-tail phosphorylation but unaffected by PIP_2_ (Extended Data Fig. 6g).

Collectively, our simulations indicate that physiological PIP_2_ levels promote ICL3-membrane interactions without altering the active conformation of β_2_AR, on timescales sufficient to capture TM3–TM6 distance changes. Phosphorylation of the β_2_AR C-tail slightly enhances its interactions with ICL3 and with ICL2 in the presence of PIP_2_.

### PIP_2_ binding and βarr2 activation stabilize βarr2 on membranes

**Fig. 5:**
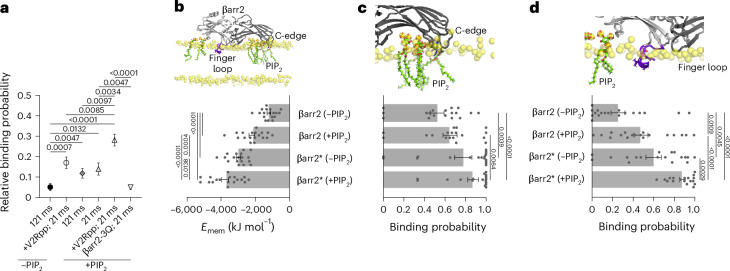
PIP_2_ and βarr2 activation promote βarr2 interaction with membrane. **a**, Binding probabilities measured with DFS of βarr2-WT with and without the activating phosphopeptide mimicking the phosphorylated C-tail of the V2Rpp and of βarr2-3Q without V2Rpp. Binding probability of βarr2-WT with lipid membranes without PIP_2_ (−PIP_2_) at 121 ms interaction time without V2Rpp (40 replicates with 14 cantilevers from 3 independent functionalizations) and with V2Rpp at 21 ms (66 replicates, 22 cantilevers, 3 independent functionalizations); binding probability of βarr2-WT with lipid membranes with PIP_2_ (+PIP_2_) at 121 ms (28 replicates, 8 cantilevers, 4 independent functionalizations) and 21 ms (43 replicates, 13 cantilevers, 4 independent functionalizations) without V2Rpp and at 21 ms with V2Rpp (33 replicates, 9 cantilevers, 4 independent functionalizations); binding probability of βarr2-3Q with lipid membranes +PIP_2_ at 21 ms (73 replicates, 24 cantilevers, 3 independent functionalizations). Binding probabilities are shown as mean ± s.e.m. and are normalized to the binding probability of the β_2_AR–βarr2 tail–core complex (−PIP_2_) at 21 ms (Fig. 2h). **b**, Top: exemplary MD simulation snapshot of active βarr2 (gray) interacting with a PIP_2_ (green)-containing DOPC:DOPG:cholesterol membrane (yellow spheres). Bottom: average *E*_mem_ of βarr2–membrane interactions during the last 10 ns of MD simulations of inactive and active (*) βarr2 with membranes without and with PIP_2_. **c**,**d**, Detailed views on exemplary MD simulation snapshot from **b** (top) and binding probability of the C-edge (bottom) (**c**) and finger loop (**d**) of βarr2 to the membrane for βarr2–membrane simulations. N-lobe of βarr2 is light gray, C-lobe dark gray, C-edge residues L192, K227 and R332 are shown as sticks, finger loop is purple with residues L69–L72 shown as sticks. PIP_2_ molecules in contact with βarr2 are shown as sticks, phosphates yellow, oxygens red and lipid carbons green. Data in **b**–**d** are shown as mean ± s.e.m. of 18 simulations (data points) per condition. *P* values in **a**–**d** are obtained from two-sided Mann–Whitney tests. Source data

As PIP_2_ increases *E*_mem_ between βarr2 and the membrane and retains βarr2 at the membrane (Fig. 4f), we examined βarr2–membrane interactions experimentally and by simulations. At both interaction times of 21 ms and 121 ms, DFS detected a twofold higher binding probability of βarr2 to DOPC:DOPG:cholesterol:PIP_2_ membranes lacking β_2_AR compared with membranes without PIP_2_ (Fig. 5a). These similar binding probabilities show that βarr2 binding to PIP_2_ membranes happens within milliseconds once βarr2 is in close proximity. We therefore continued our membrane binding measurements at the 21-ms interaction time.

Because we used full-length βarr2, including the autoinhibitory C-tail, we expected it to adopt mostly inactive conformations before membrane engagement. To probe how active βarr2 interacts with membranes without and with PIP_2_, we repeated DFS in the presence of V2Rpp. Activation of βarr2 by V2Rpp increased the binding probability threefold to membranes lacking PIP_2_ and twofold to PIP_2_ membranes. Notably, βarr2-3Q bound PIP_2_ membranes with the same probability as βarr2-WT bound PIP_2_-free membranes (Fig. 5a), indicating that βarr2-3Q cannot sense PIP_2_ and consequently cannot exploit it to stabilize membrane interactions. V2Rpp has been shown to activate βarr-3Q and expose its finger loop^34^. Simulations presented here and elsewhere^33^ show that βarr2 can interact with phospholipids through residues of the finger loop. As these residues are unaltered in βarr2-3Q, we expect V2Rpp-activated βarr2-3Q to bind more readily to membranes, albeit less than activated βarr2-WT.

Overall, the binding probability of βarr2 to membranes without β_2_AR was substantially lower than in the presence of β_2_AR (Figs. 2, 3 and 5). In PIP_2_ membranes at 21 ms, for example, the binding probability without β_2_AR was 0.14 ± 0.03 compared with 0.47 ± 0.03 for the β_2_AR–βarr2 core complex. This marked reduction in binding probability is consistent with cellular observations that only few βarr2 molecules localize to the plasma membrane when GPCRs are not activated^33^, indicating that inactive βarr2 rarely engages the membrane. Although βarr2 activation alone increases membrane interactions independently of PIP_2_, only the combination of βarr2 activation and PIP_2_ binding increases βarr2–membrane binding to levels comparable to βarr2 binding to β_2_AR, suggesting that βarr2 activation and PIP_2_ binding are necessary for prolonged membrane association observed after receptor binding.

Collectively, our experimental findings show that PIP_2_ promotes membrane engagement of inactive βarr2 and further stabilizes that of active βarr2, although βarr2–membrane interactions remain less frequent than βarr2-β_2_AR interactions. These results are consistent with reports that βarr2 can, albeit rarely, associate with the plasma membrane before GPCR recruitment^33^, and that PIP_2_ stabilizes active βarr2 at the membrane even without a bound GPCR^21^.

MD simulations corroborated that both βarr2 activation and PIP_2_ strengthened *E*_mem_ (Fig. 5b), and the simulated *E*_mem_ correlated well with the binding probabilities measured by DFS. The βarr2 C-edge and finger loop correspondingly increased binding probabilities to PIP_2_ membranes upon activation (Fig. 5c,d), in agreement with βarr2–membrane interactions described for tail complexes in PIP_2_-free and PIP_2_ membranes (Fig. 4c). Structural snapshots further revealed that the finger loop of inactive βarr2 was released from the N-lobe and C terminus binding pocket in both membrane compositions (28% of simulations without PIP_2_ and 33% with PIP_2_). In this extended conformation, several hydrophobic finger loop residues interact with the membrane and stabilize βarr2, consistent with previous observations^33^.

Together, these findings establish PIP_2_ as a key determinant of βarr2–membrane association and β_2_AR engagement, providing a mechanistic link between membrane composition and the regulation of GPCR–β-arrestin signaling.

## Discussion

Class B GPCRs, such as the V2 vasopressin receptor (V2R), bind β-arrestins tightly^4^ and β-arrestin interactions with this class, predominantly through V2R C-tail fusion receptors, are well characterized^12,14,24,54^. Unlike class A GPCRs, such as β_2_AR, which engage β-arrestins only transiently at the plasma membrane, class B GPCRs remain associated with β-arrestins after endocytosis. This distinction is encoded in part by the receptor C-tail^4,39,40^, and in part by the membrane. Class A GPCRs require β-arrestins binding to phosphoinositides for assembly, desensitization and internalization, whereas this dependence is largely absent for class B GPCRs, consistent with their persistent endosomal association^34^. Because the transient and conformationally flexible nature of class A GPCR–β-arrestin complexes precludes high-resolution structural characterization^43^, dynamic biophysical methods are best suited to dissect them. AFM has proven to be a valuable tool for characterizing the dynamic binding of GPCR–ligand complexes at the single-molecule level^48,61^. Here we applied several complementary AFM-based SMFS modes in combination with atomistic MD simulations to quantify the kinetic, mechanical and energetic properties of β_2_AR–βarr2 complexes and the role of PIP_2_ in their assembly and free-energy landscape. By controlling the β_2_AR state, namely phosphorylated, activated or both phosphorylated and activated, we could characterize β_2_AR–βarr2 tail, core and tail–core complexes.

**Fig. 6:**
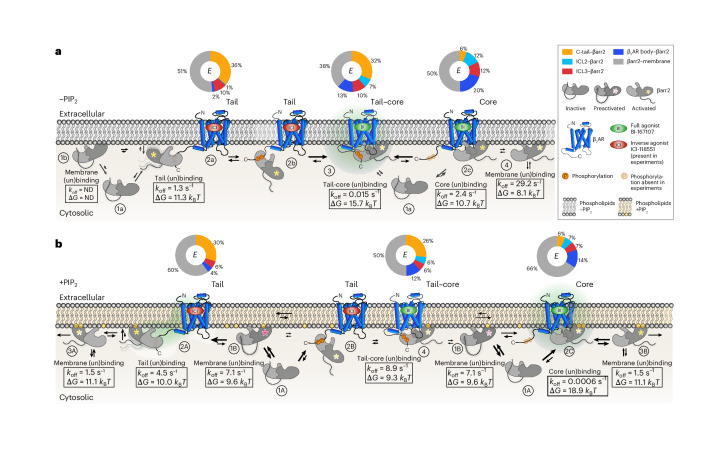
Model of the assembly of β_2_AR–βarr2 complexes in the absence and presence of PIP_2_. **a**, βarr2 binding to β_2_AR in DOPC:DOPG:cholesterol membranes without PIP_2_. Inactive βarr2 in the cytosol (1a) partitions to the membrane (1b), where brief interactions occur. βarr2 binds to the phosphorylated C-tail of activated β_2_AR to assemble a tail complex within milliseconds, while transiently interacting with the membrane (2a) or only engaging the receptor C-tail (2b). Within tens of milliseconds, tail complexes can mature into mechanically and kinetically more stable tail–core complexes through binding to the active receptor core (3). A tail–core complex can also be assembled through initial core binding (2c), albeit at a much lower probability. Membrane interactions of active βarr2 (4) are more frequent than those of inactive βarr2 (1b) but remain weak. Pie charts show relative contributions of β_2_AR C-tail, ICL2, ICL3 and body, and of membrane to the interaction energy *E* of different βarr2–β_2_AR complexes derived from equilibrium MD simulations. **b**, βarr2 binding to β_2_AR in DOPC:DOPG:cholesterol:PIP_2_ membranes. Inactive βarr2 in the cytosol (1A) may spontaneously preassociate with the membrane^33^ with moderate affinity, where it adopts a preactivated conformation on interaction with PIP_2_ (1B)^34^. Within milliseconds, βarr2 can bind to the phosphorylated β_2_AR C-tail (2A, 2B). If βarr2 first binds to the receptor C-tail, contacts between βarr2 and the membrane are established (transition 2B to 2A). Receptor tail binding promotes βarr2 to adopt an active conformation and associate more stably with the membrane. If the C-tail of β_2_AR is minimally or not phosphorylated, stable β_2_AR–βarr2 core complexes can assemble within tens of milliseconds (2C). The receptor core and interactions of βarr2 with PIP_2_ can activate arrestin and stabilize it at the membrane. As previously shown^21,33^, βarr2 can remain membrane-associated after dissociating from β_2_AR (3A, 3B). Assembly of a stable tail–core complex (4) either from a tail (2B) or a core (2C) complex was not observed experimentally. Transition rates *k*_off_ and free-energy barrier heights Δ*G*^‡^ originate from DFS measurements at 21 ms (tail complex, βarr2–membrane interactions; Figs. 2 and 3 and Supplementary Fig. 10) and 121 ms (core, tail–core complexes; Figs. 2 and 3). Bigger arrows correspond to a higher probability of transition based on our results and previously reported observations. ND, not determined.

Height-clamp SMFS captured spontaneous binding and unbinding of single βarr2 to agonist-activated, GRK5-phosphorylated β_2_AR, yielding a β_2_AR–βarr2 complex lifetime of roughly 50 ms, which is considerably shorter than the 20–50 seconds previously reported^28,29,32^. This likely reflects the nonequilibrium height-clamp experiment, in which the deflecting cantilever applies a counterforce to βarr2 when it binds β_2_AR, which shifts the β_2_AR–βarr2 complex out of equilibrium, thereby reducing the lifetime of the interaction^47^. The counterforce prevents the binding event from maturing, which may be necessary if complex assembly involves intermediate steps. Thus, to probe how β_2_AR–βarr2 complexes stabilize at equilibrium (that is, in the absence of externally applied force), we applied DFS to β_2_AR–βarr2 tail, core and tail–core complexes without and with PIP_2_. βarr2 engaged the phosphorylated C-tail of β_2_AR within around 20 ms (Fig. 6). Assembly of this early tail-engagement complex was independent of PIP_2_, with kinetic stabilities matching those observed by single-molecule fluorescence microscopy in cells^33^. Over longer interaction times, however, the β_2_AR–βarr2 tail complex destabilized without PIP_2_ but remained stable in its presence.

β_2_AR–βarr2 core complexes assembled less readily than tail complexes within roughly 20 ms, and without PIP_2_ stabilized only marginally at longer interaction times. PIP_2_ markedly altered this behavior, it increased the probability of core complex assembly, prolonged its lifetime and increased ∆*G*^‡^, while a concomitant increase in *x*_b_ indicates greater conformational variability. Given that the β_2_AR core can drive βarr2 into active-like conformations^21,22^, this larger conformational variability may reflect a broader ensemble of such conformations, and the receptor core was shown to be sufficient to stabilize βarr2 at the plasma membrane and promote internalization^21^. Indeed, in the presence of PIP_2_, the β_2_AR–βarr2 core complex becomes the most stable of the three, supporting a model in which βarr2 engages the β_2_AR transmembrane core independently of receptor phosphorylation to adopt a functionally relevant active conformation^22^. Phosphorylation-independent β-arrestin recruitment to the receptor core has been documented for several GPCRs^51,62,63^, reinforcing the biological relevance of the core-only β_2_AR–βarr2 interaction.

The β_2_AR–βarr2 tail–core complex behaved differently. Without PIP_2_, βarr2 engaged active, phosphorylated β_2_AR within 21 ms with properties intermediate between tail and core complexes, suggesting binding to either the β_2_AR tail or core at this early stage. At longer interaction times, this intermediate matured into fully engaged tail–core complex, with a transition rate slightly higher than that of the PiP_2_-stabilized core complex and a lifetime comparable to those measured by FRET in cells^28,29,32^. In PIP_2_ membranes, the tail–core complex failed to stabilize even after >500 ms, instead retaining the lifetime and stability of the tail complex. This indicates that only β_2_AR–βarr2 core complexes stabilize when PIP_2_ is present. Control DFS measurements with the βarr2-3Q mutant whose PIP_2_ binding was blunted reversed this behavior and the core complex destabilized while the tail–core complex gained some stability in PIP_2_ membranes, thus underscoring the central role of PIP_2_ in modulating β_2_AR–βarr2 complex assembly.

Our MD simulations support a role of membrane phosphoinositides in shaping the conformational dynamics of β_2_AR–βarr2 complexes. Consistent with cryogenic-electron microscopy showing that tail, core and tail–core complexes adopt multiple conformations^12,16,19,20,54,55^, we find that the orientation of βarr2 relative to β_2_AR is governed by β_2_AR core interactions and by βarr2 C-edge engagement. βarr2 samples a wide range of orientations in the absence of PIP_2_, but this conformational heterogeneity of β_2_AR–βarr2 complexes is reduced in the presence of PIP_2_. The impact is strongest in tail-only complexes, where βarr2 binds to the flexible β_2_AR C-tail and PIP_2_ attracts the positively charged residues of the C-lobe of βarr2, including the known PIP_2_ binding residues K233, R237 and K251^50^, to the membrane. As a result, the βarr2 finger loop inserts into the membrane and orients βarr2.

PIP_2_ also raises the interaction energies stabilizing all three β_2_AR–βarr2 complexes, exclusively through enhanced βarr2–membrane interactions. This effect of PIP_2_ was most pronounced for core complexes, although β_2_AR–βarr2 interaction energies and the individual contributions of the C-tail, ICL2, ICL3 and transmembrane domain remained unaltered by PIP_2_. In agonist-bound (un)phosphorylated β_2_AR simulations, ICL3 interacts with the membrane more frequently when PIP_2_ is present. Although higher PIP_2_ concentrations were previously proposed to correlate with an increased TM3–TM6 distance and thus a more active C-tail-lacking receptor^57^, our simulations at moderate PIP_2_ levels show no such activation. Nevertheless, ICL3 sequestration at PIP_2_ membranes could render the β_2_AR core more accessible to βarr2 and so favor core complex stability.

Phosphorylation of the β_2_AR C-tail promoted C-tail–ICL2 interactions at selected residues in PIP_2_ membranes, but the probability of the β_2_AR C-tail being free, interacting neither with the membrane nor the receptor, was comparable across phosphorylation states and membrane compositions. Our simulations therefore cannot corroborate a β_2_AR C-tail autoinhibitory mechanism preventing tail–core complex formation^11,64^, rather, our experimental data indicate that the lack of tail–core complex stabilization in PIP_2_ membranes arises from activated βarr2 being retained at the membrane through PIP_2_ binding.

The absence of tail–core complex stabilization in our experiments appears to diverge from work on the class B GPCR neurotensin type 1 receptor (NTSR1), where PIP_2_ was reported to stabilize a tail–core conformation^34^. However, those previous observations were made with detergent-solubilized NTSR1 and βarr1, in which the absence of β-arrestin–membrane interactions confers additional rotational freedom, which potentially allows complex conformations that accommodate both tail and core binding, as observed in the βarr1–neurotensin receptor 1 structure^17^. SMFS instead let us probe the dynamics and stability of βarr2 complexes with the class A GPCR β_2_AR in complex phospholipid membranes, where our experiments and simulations indicate that tail–core complexes fail to stabilize in the presence of PIP_2_ because of the stabilizing effect of PIP_2_ on βarr2–membrane interactions.

Our simulations of inactive, C-tail truncated preactivated and phosphopeptide-activated βarr2 with and without PIP_2_, reveal that (1) PIP_2_ increases the interaction energy with the membrane and orients βarr2 at the membrane, (2) (pre)activated βarr2 has a higher propensity for membrane association and (3) the finger loop of inactive βarr2 can extend from the inactive state to engage phospholipids. Corroborating DFS measurements show that phosphopeptide activation and PIP_2_ individually and synergistically promote βarr2–membrane association. Consistent with this, receptor-activated βarr2 has indeed been shown to remain membrane-associated after receptor dissociation^21,33^, and PIP_2_ alone can induce active-like β-arrestin conformations^34,52^, which may explain why inactive βarr2 preferentially binds PIP_2_ membranes.

Collectively, our data support a model where β_2_AR–βarr2 tail–core complexes cannot stabilize in the presence of PIP_2_ because, once activated by the phosphorylated tail, βarr2 establishes energetically stable interactions with the PIP_2_ membrane. For active βarr2, attached to the phosphorylated C-tail of β_2_AR and to PIP_2_ to insert its finger loop into the receptor core, it would first have to unbind from the membrane, overcoming a substantial free-energy barrier. We cannot exclude that inactive βarr2 first binds the core of activated, phosphorylated β_2_AR and subsequently engages the phosphorylated C-tail. However, our DFS experiments show that βarr2 binds the phosphorylated C-tail more readily than the core, so the tail–core complexes formed through this alternative route are less probable.

In conclusion, by developing an in vitro assay to monitor the assembly and stabilities of human β_2_AR–βarr2 complexes in two defined membrane environments, we determined the contributions of phosphoinositides as well as receptor phosphorylation and activation to β_2_AR–βarr2 tail, core and tail–core complex formation. Extending these pathways to native cell membranes, where composition and properties are far more intricate, is an exciting next challenge that may reveal further layers of regulation. Combining our experiments with simulations, we propose an updated β_2_AR–βarr2-binding model, key elements of which have been corroborated across other GPCRs^21,33,34^ (Fig. 6). Inactive βarr2 first transiently preassociates with the membrane. Within milliseconds βarr2 can then bind PIP_2_ or the phosphorylated β_2_AR C-tail. In the presence of PIP_2_, membrane-preassociated^33^ βarr2 bound to the phosphorylated C-tail of β_2_AR is fully activated^34^, binds the membrane more strongly and so enters a feed-forward cycle. This finding is in line with recent single-molecule FRET studies^65^. In the absence of C-tail phosphorylation, βarr2 can still engage the active β_2_AR core from either cytosolic or transient membrane-associated states within tens of milliseconds. This core complex is substantially stabilized by activation of βarr2 by both the receptor core and PIP_2_, as expected in PIP_2_-enriched plasma membranes and clathrin-coated regions^66^. Notably, the fully engaged tail–core complex of βarr2 with the transmembrane core and phosphorylated C-tail of β_2_AR assembles only in membranes lacking PIP_2_. Together, these results uncover a membrane-driven mechanism that governs the temporal and structural control of β_2_AR–βarr2 assembly, illuminating how phosphoinositides modulate GPCR signaling outcomes.

## Methods

### Experimental section

#### General

BI and V2Rpp (ARGRpTPPpSLGPQDEpSCpTpTApSpSpSLAKDTSS) were obtained by custom synthesis (the latter from the Tufts University Core Facility).

#### βarr2 purification

The parental Cys-less human βarr2 (C17S, C60V, C126S, C141L, C151V, C243V, C252V, C270S, C409S) or Cys-less βarr2 bearing the additional 3Q mutations (K233Q, R237Q, K251Q), with a single cysteine reintroduced on the cytoplasmic face of the C-domain (S267C) were prepared as previously described in ref. ^34^. Briefly, the construct carries an N-terminal 6× Histidine tag, followed by a 3C protease site, a GG linker, an AviTag and a GGSGGS linker. The sequence was codon-optimized for expression in *Escherichia coli* and cloned into a pET-15b vector. βarr2 constructs were transformed into NiCo21(DE3) competent *E. coli* (NEB) and large-scale cultures were grown in terrific broth plus ampicillin at 37 °C until an optical density at 600 nm (OD_600_) of 1.0 was reached. Cells were then transferred to room temperature (RT) and induced with 25 μM isopropyl-β-D-thiogalactoside (IPTG) when the OD_600_ reached 2.0. Cells were collected 20 h postinduction and resuspended in lysis buffer: 50 mM HEPES pH 7.4, 500 mM NaCl, 15% glycerol, 7.13 mM β-mercaptoethanol, to a final volume of 40 ml l^−1^ of cells. Cells were lysed by sonication and the clarified lysate was incubated with Ni-sepharose resin and batch incubated for 1.5 h at 4 °C. The resin was washed with 10 resin volumes of wash buffer (20 mM HEPES pH 7.4, 500 mM NaCl, 10% glycerol, 7.13 mM β-mercaptoethanol, 20 mM imidazole), followed by 10 resin volumes of wash buffer plus more 20 mM imidazole. The protein was then eluted in 5 resin volumes of wash buffer plus 200 mM imidazole. Eluted protein was mixed with 1:20 (w:w) 3C protease while being dialyzed overnight into 20 mM HEPES pH 7.4, 200 mM NaCl, 2 mM β-mercaptoethanol, 10% glycerol. The digest was reapplied to Ni-sepharose resin equilibrated in dialysis buffer and the flow through was diluted with NaCl-free dialysis buffer to bring the NaCl concentration to 75 mM (final). The protein was then purified by ion exchange chromatography (monoQ 10/100 GL, GE Healthcare), followed by size-exclusion using a Superdex200 Increase 10/300 GL column (GE Healthcare) with SEC buffer (20 mM HEPES pH 7.4, 300 mM NaCl, 10% glycerol). Purified protein was concentrated to 100–300 μM using a 30-kDa spin concentrator, aliquoted, flash frozen in liquid nitrogen and stored at −80 °C.

#### β_2_AR purification and phosphorylation

Human β_2_AR bearing an N-terminal Flag epitope tag, a C-terminal 6× His tag, and the mutations C77V, C327S, C341L, C378A, C406A, M96T, M98T, N187E was expressed in *Sf*9 insect cells (Expression Systems) using the BestBac baculovirus system (Expression Systems). Cells were infected at 4 × 10^6^ cells per ml and incubated for 48 h at 27 °C. Receptor was extracted as described in ref. ^68^. Briefly, the receptor was first purified by Flag affinity chromatography, then by alprenolol sepharose chromatography to enrich for functional receptors. The alprenolol sepharose eluate was concentrated on Flag affinity resin, and then washed with ligand-free buffer for 30 min at RT to remove alprenolol. Receptor was eluted, aliquoted, flash frozen with 20% glycerol (final) in liquid nitrogen and stored at −80 °C.

β_2_AR was phosphorylated in mixed micelles using DOPS and GRK5. β_2_AR in *n*-dodecyl-β-ᴅ-maltoside (DDM) (1 μM) was mixed with DOPS (100 μM final) and epinephrine (200 μM final), and then was incubated for 45 min at 25 °C before shifting to 30 °C and mixing with shaking for an additional 15 min. ATP was added to 1 mM final, followed by 100 nM GRK5. After 30 min a second portion of GRK5 was added and incubated for a further 30 min. Following phosphorylation DDM/CHS was added to the reaction to 1%/0.1% final, and the sample was incubated at 25 °C for 15 min. The phosphorylated β_2_AR was purified by Flag affinity purification and the eluate was combined and subjected to size-exclusion chromatography (SEC) on a Superdex200 Increase 10/300 GL column (GE healthcare). β_2_AR phosphorylation was assessed by ProQ and Sypro Ruby staining, following the manufacturer’s protocols.

#### Liposome preparation and β_2_AR reconstitution

Lipid stocks in chloroform were mixed in the desired molar proportions, dried with a gentle stream of argon to form a lipid film on the side walls of a glass vial and dried overnight using a SpeedVac to remove residual solvent. The mixture was resuspended in HNE buffer (20 mM HEPES pH 7.4, 100 mM NaCl, 1 mM EDTA) to 5 mg ml^−1^ final. Lipid suspensions were mixed by vortexing and brief sonication cycles to ensure all lipids were resuspended. Lipid stocks were flash frozen and stored at −80 °C.

For reconstitution, lipid stocks were thawed in warm water and extruded first by 11 passes through a 0.4-µm membrane in a 1-ml mini-extruder, followed by 11 passes through a 0.1-µm membrane in a 1-ml mini-extruder. Liposomes were incubated with an empirically determined concentration of DDM sufficient to permeabilize but not dissolve the liposomes for 3 h at 25 °C. β_2_AR in DDM was added to the liposomes such that the lipid:protein ratio was 30:1 (w:w). The mixture was equilibrated for 3 h at 4 °C before BioBeads were added at a ratio of 10:1 BioBeads:DDM (w:w). After another hour, a second portion of BioBeads was added at a ratio of 10:1 BioBeads:DDM (w:w). After another hour, a third portion of BioBeads was added at a ratio of 10:1 BioBeads:DDM (w:w), and this mixture was equilibrated overnight. The following morning, BioBeads were removed by centrifugation and proteoliposomes were diluted with NH buffer (20 mM HEPES pH 7.4 and 100 mM NaCl) and pelleted by ultracentrifugation (2 h at 200,000*g*). Isolated liposomes were resuspended in NH buffer and evaluated by radioligand binding.

#### Radioligand binding

Radioligand binding was performed as previously described in ref. ^45^. Briefly, 500 μl of binding reactions contained 50-fmol receptor, 0.5 nM ^3^H dihydroalprenolol (^3^H-DHA), 100 mM NaCl, 20 mM HEPES pH 7.5 and 0.1% bovine serum albumin. Reactions were mixed, then incubated for 4 h at RT, filtered through a Brandel 48-well harvester onto filters pretreated with 0.1% polyethylenimine and quantified by liquid scintillation counting. All measurements were performed in triplicate.

#### GRK5 expression and purification

Full-length wild-type human GRK5 was modified with a C-terminal hexa-histidine tag and cloned into pVL1392 vector for baculovirus production. GRK5 was expressed and purified as previously published in ref. ^69^. Briefly, *Sf9* insect cells (Expression Systems) were infected with a BestBac-derived baculovirus at a density of 3.5 × 10^6^ cells per ml and collected 48 h postinfection. Cells were resuspended, lysed by sonication and the supernatant was applied to Ni-NTA resin. The resin was washed with lysis buffer and GRK5 eluted with lysis buffer supplemented with 200 mM imidazole. The combined eluate was then subjected to cation-exchange chromatography using a MonoS 10/100 column (GE healthcare) and eluted with a linear gradient of NaCl. Fractions containing GRK5 were combined and run on a Superdex200 10/300 GL column (GE healthcare). GRK5 was aliquoted, flash frozen and stored at −80 °C.

#### Nb6B9 expression, purification and labeling

Nb6B9 was expressed and purified as previously described in ref. ^45^. Briefly, Nb6B9 cloned in the expression vector pET26b, containing an N-terminal pelB signal sequence, and a C-terminal 8× His tag was transformed into BL21(DE3) Rosetta2 *E. coli* (Novagen). Cells were grown in Terrific Broth, induced at OD_600_ 0.8 with 1 mM IPTG and shaken at 22 °C for 24 h. Periplasmic protein, obtained by osmotic shock, was purified using Ni-sepharose, dialyzed into 20 mM HEPES, 100 mM NaCl, concentrated to around 200 µM, supplemented with glycerol (20% final), flash frozen in liquid nitrogen and stored at −80 °C.

Nb6B9 was labeled as follows: Nb6B9 was diluted to roughly 30 µM in phosphate buffer (pH 7) and reacted with ATTO488-NHS (in dimethyl-sulfoxide (DMSO), 350 µM final) for 4 h at 25 °C with constant mixing. The reaction was quenched with excess hydroxylamine (30 min, 25 °C), and then chilled on ice. Excess dye was removed by flowing the reaction over equilibrated Ni-sepharose and washed until no dye eluted. Labeled Nb6B9 was eluted with 20 mM HEPES, 100 mM NaCl pH 7.4, 200 mM imidazole, concentrated and subjected to SEC on a Superdex200 Increase 10/300 GL column (GE Healthcare) with SEC buffer (20 mM HEPES pH 7.4, 100 mM NaCl). Pooled fractions were concentrated to about 200 µM, supplemented with glycerol (20% final), flash frozen in liquid nitrogen and stored at −80 °C.

#### SMFS

SMFS was done using an AFM (Bioscope Resolve, NanoScope 9.4R3 software, Bruker) placed on an active damping table (Table Stable and Park Systems) housed in a noise-insulating box.

In height-clamp SMFS, FT curves were collected in the FD-AFM mode (Peak Force QNM mode, Bruker). The cantilever tip was set to (1) approach the sample with a speed of 0.5 µm s^−1^ until a force of 100 pN was detected, (2) retract by 4–8 nm, (3) stay at the defined height for 2 s recording the cantilever deflection (3.086 kHz sampling rate) and (4) retract by 100 nm. Steps (1) to (4) were repeated. Binding events were characterized by a sudden change in force in an FT curve. Data on β_2_AR membranes comprised 8,305 FT curves from 21 cantilevers and 5 independent tip functionalizations (Fig. 1). Control experiments on membranes without β_2_AR comprised 3,038 FT curves from 13 cantilevers and 5 independent tip functionalizations.

For DFS, the AFM was operated in the FD-AFM mode (force–volume mode, Bruker). β_2_AR-containing membranes were imaged with cantilever tips functionalized with βarr2 at an approach speed of 1,090 nm s^−1^, an imaging force of 100 pN, a ramp size of 40 nm, 96 pixels or 128 pixels per line, and 256 or 512 points per FD curve. Six retraction speeds of 203 nm s^−1^, 501 nm s^−1^, 1,090 nm s^−1^, 2,170 nm s^−1^, 3,260 nm s^−1^ and 6,510 nm s^−1^ were used, yielding an average tip-sample interaction time of 21 ± 2 ms (no delay time added) or 121 ± 2 ms (with a 100-ms retraction delay). Cantilevers were calibrated by ramping on mica (deflection sensitivity) and thermal tuning (spring constant). All measurements were performed at RT. Data with membranes without β_2_AR were obtained with 13 and 9 (+V2Rpp) cantilevers from 4 independent functionalizations (Fig. 5 and Supplementary Figs. 10 and 11).

#### Functionalization of cantilever tip

Tips of AFM cantilevers were covalently functionalized with PEG_27_-linker–βarr2 as follows. Cantilevers (AC40, Bruker) with a tip radius of 8 nm, a nominal spring constant of 0.07 N m^−1^ and resonance frequency of 25 kHz in water were used. For amino-functionalization of the cantilever tips, a solution of 3.3 mg ethanolamine hydrochloride (Sigma-Aldrich) in 6.6 ml of DMSO (Sigma-Aldrich) with molecular sieve 0.3-nm beads (Supelco) was degassed for 30 min. Cantilevers were cleaned for 10 min in ultraviolet radiation and ozone (UV-O cleaner, Jetlight) and submerged in the above solution overnight at RT. Amino-functionalized cantilevers were washed three times in DMSO, gently rinsed with ethanol (Sigma-Aldrich) and dried with nitrogen gas. To introduce the PEG_27_-linker, the amino-functionalized cantilevers were immersed in a solution of 1 mg of maleimidopropionyl-PEG_27_ NHS ester (Polypure) in 500 µl of chloroform (Sigma-Aldrich) and 30 ml of triethylamine (Sigma-Aldrich) for 2 h at RT. Next, PEG_27_-functionalized cantilevers were incubated in chloroform 3 times for 10 min each and subsequently dried with air. Subsequently, the cantilever tips were functionalized with 2 µM βarr2 in 100 µl of Dulbecco’s PBS (no CaCl_2_, no MgCl_2_; Gibco) supplemented with 2 µl of 100 mM EDTA (pH 7.5), 5 µl of 1 M HEPES (pH 7.5), 2 µl of 100 µM tris(2-carboxyethyl)phosphine hydrochloride and 2 µl of 1 M HEPES (pH 9.6) for 4 h at RT. Finally, the cantilevers were washed in Dulbecco’s PBS and stored in the same solution at 4 °C for up to 1 week. The combination of a sharp tip with a radius of the same size as βarr2 and a low βarr2/PEG_27_-linker ratio ensured that only one βarr2 bound at the apex of the cantilever tip, thus allowing us to measure single-molecule interactions^70^.

#### Sample preparation for SMFS

Mica disks (roughly 0.1 mm thick, 8 mm diameter) were punched using a ‘punch and die’ set (Precision Brand Products) and glued (Araldite rapid) on microscope glass slides (Menzel-Glass, 76× 26 mm, Thermo Scientific). Teflon rings were glued around the mica disks to prevent buffer from spreading beyond mica. Reconstituted β_2_AR was diluted 150-fold in 70 µl of imaging buffer (150 mM KCl (Merck), 20 mM HEPES (AppliChem), pH 7.4) containing either 10 µM ICI (Tocris) or 5 µM BI (custom synthesis) and centrifuged at 16,100*g*, 4 °C, for 15 min. Then, the supernatant was removed and the remaining sample was resuspended in 70 µl of imaging buffer supplemented with the ligand and incubated at RT for 15 min for ligand binding to β_2_AR. Mica was cleaved using adhesive tape to create a clean, atomically flat surface, onto which 50 µl of ligand-bound β_2_AR was adsorbed for 25 min. Next, the sample was washed 10 times with 50 µl of imaging buffer containing ligand. Buffers were prepared with nanopure water (0.055 µS cm^−1^) and analytical grade chemicals.

For the control experiment in Extended Data Fig. 3, unphosphorylated β_2_AR reconstituted in PIP_2_ membranes was incubated with 10 U lambda protein phosphatase (New England Biolabs) per nanomole of receptor and 2.5 mM MnCl_2_ in NEBuffer for protein metallophosphatases (New England Biolabs) overnight at 4 °C. Subsequently, the sample was prepared for SMFS as described above.

#### Combined FD-based AFM imaging and confocal microscopy

The AFM was mounted on an inverted confocal microscope (LSM 800, Carl Zeiss) and operated in the FD-AFM mode (Peak Force QNM mode, Bruker). Sample supports were prepared on thin coverslips (Menzel-Glass, 24× 60 mm, no. 1, Thermo Scientific) using transparent glue (Araldite Crystal). Then 1 µl of reconstituted phosphorylated β_2_AR was diluted in 50 µl of imaging buffer containing either 5 µM BI or 10 µM ICI, and centrifuged for 15 min at 16,100*g* and 4 °C The supernatant was removed, and the remaining sample was resuspended in 100 µl of imaging buffer containing the ligand and incubated at RT for 15 min. Mica was cleaved with adhesive tape and 50 µl of sample was adsorbed for 45 min. The sample was washed 10 times with 50 µl imaging buffer containing ligand. To prevent nonspecific nanobody binding, the sample was blocked with 45 µl of 3% (w/v) bovine serum albumin (Sigma-Aldrich) in ligand-containing imaging buffer for 1 h at RT, then incubated with 45 µl of 700 nM Nb6B9-ATTO488 in 3% (w/v) bovine serum albumin for 1 h at RT, and finally washed 10 times with 50 µl of imaging buffer containing ligand. Confocal images were acquired using a 10 mW, 488 nm laser at 4% nominal power, master gain 750 V and pinhole 1 airy unit through a 63× water immersion objective (421787-9970-799, numerical aperture 1.20, Carl Zeiss). Images were collected in 8-bit mode with 4-line averaging to obtain a mean intensity.

#### Analysis of FT and FD curves

FT curves from height-clamp measurements were analyzed in R^71^ using scripts developed in-house. First, FT-curve files were preprocessed to normalize the starting time to zero and convert force units to picoNewtons. Second, an interactive Shiny application^72^ scripted in R in-house was used to view preprocessed FT curves and select binding events. Third, for every event, the lifetime Δ*t* and interaction force Δ*F* were calculated. Δ*t* was calculated as the time difference between the first (*t*_1_) and last (*t*_2_) data point of an event, that is, the last and the first data point at the level of the baseline before and after the event, respectively. Δ*F* was calculated as the absolute value of the difference between the mean force between *t*_1_ and *t*_2_ (*F*_2_) and the baseline (*F*_1_) defined as the mean force of ≤50 data points before and after *t*_1_ and *t*_2_, respectively.

FD curves from DFS measurements were analyzed for specific β_2_AR–βarr2-binding events using in-house MATLAB (MathWorks) scripts. The flexible PEG_27_-linker tethering βarr2 to the cantilever tip allowed selection of single (un-)binding events between βarr2 and β_2_AR (Supplementary Fig. 6b–d). Nonspecific adhesion events were excluded by a 3–20 nm distance filter, which discarded (nonspecific) events <3 nm from the contact point and those >20 nm beyond it (longer than the stretched PEG_27_-linker). Single β_2_AR–βarr2 interactions were defined by FD curves containing exactly one specific adhesion event with a force greater than five times the baseline noise s.d. To estimate the tip-sample contact time, the time from the first to the last tip-sample contact was extracted from FT curves recorded at the experimental conditions described. The contact times were averaged from three FT curves recorded with three different cantilevers at each retraction speed.

#### Extraction of parameters of the free-energy landscape

Kinetic and energetic parameters of β_2_AR–βarr2 complexes were extracted by fitting binned height-clamp and DFS data with the Bell model^47^ and the Bell–Evans model^67^, respectively, in GraphPad Prism.

The number of bins for height-clamp data was determined by the Freedman–Diaconis rule^73^ and the binned data were fit with the Bell model using the following equation:

1 $$t\left(F\right)={t}_{0}{\mathrm{e}}^{\frac{-{x}_{\beta }F}{{k}_{\mathrm{B}}T}}$$

where *t*(*F*) is the interaction lifetime as a function of force, *t*_0_ the interaction lifetime at equilibrium, that is, at zero external force, *x*_β_ the distance between the bound state and the transition state toward unbinding, *k*_B_ the Boltzmann constant and *T* the absolute temperature.

DFS data were binned according to the retraction speeds at which they had been acquired. The most probable rupture force and the most probable loading rate of each speed were calculated with kernel density estimation in R applying a Gaussian kernel and determining the bandwidth with Silverman’s ‘rule of thumb’^74^ (Supplementary Fig. 11). The six resulting bins of each dataset were fit with the Bell–Evans model in GraphPad Prism using the following equation:

2 $$F\left({\mathrm{LR}}\right)=\left(\frac{{k}_{\mathrm{B}}T}{{x}_{\beta }}\right)\mathrm{ln}\frac{{LR}{x}_{\beta }}{{k}_{0}{k}_{\mathrm{B}}T}$$

where *F*(LR) is the (un-)binding force as a function of the loading rate, LR, and *k*_0_ the off-rate at zero externally applied force.

The free-energy difference between bound and transition state, Δ*G*^‡^, which describes the height of the free-energy barrier separating bound and unbound states, was estimated from the Arrhenius equation:

3 $${\rm\Delta }{G}^{\ddagger }=-{k}_{\mathrm{B}}T\mathrm{ln}\left({\tau }_{D}{k}_{0}\right)$$

where *τ*_D_ is the Arrhenius frequency, for which we chose 10^−5^ s (refs. ^75–77^). Errors in Δ*G*^‡^ were obtained by error propagation from errors in *k*_0_.

#### Determination of β_2_AR–βarr2-binding probability

The probability of βarr2 binding to β_2_AR was calculated from DFS measurements as the number of specific (un-)binding events divided by the number of pixels in each measurement. The number of pixels corresponded to the number of attempts, that is, approach and retraction cycles of the cantilever in every measurement. Binding probabilities from every measurement were normalized to the mean of the β_2_AR–βarr2 tail–core complex at 21 ± 2 ms interaction time.

### MD simulations

#### Complex preparation

So far, no crystal structure of β_2_AR in complex with βarr2 has been resolved. In the absence of such structures, atomistic MD simulations can yield mechanistic insight^78–80^ through ensemble sampling of receptor–arrestin conformations, provided multiple independent replicas are generated and analyzed comparatively, with conclusions drawn from ensemble differences rather than individual trajectories^81^. Following this ensemble-based strategy, we built 8 models for each β_2_AR–βarr2 tail, core and tail–core complex and simulated each for >1 µs using all-atom MD simulations to obtain conformational ensembles. All simulations are listed in Supplementary Table 1. The initial orientation of βarr2 in the binding pocket of β_2_AR (core and tail–core complexes) was either taken from previous β_2_AR–βarr2 simulations^53^ or derived from β-arrestin-receptor complexes, that is, arrestin-1 (arr1) with rhodopsin (rho) (Protein Data Bank (PDB) 5DGY)^55^ or β-arrestin1 (βarr1) with β_1_-adrenergic receptor (β_1_AR) (PDB 6TKO)^12^. In the last cases, the structure of the active β_2_AR from the complex of β_2_AR/BI/Gs (PDB 3SN6)^82^ was fit onto the receptor (rhodopsin or β_1_AR), β-arrestin was mutated to βarr2 and the missing residues were modeled by Modeller 9.19 ref. ^83^. The structure of β_2_AR in the tail complexes originated from the crystal structures of inactive β_2_AR with the inverse agonist ICI (PDB 3NY8)^84^ and β-arrestin was localized in the solution. Therefore, all β-arrestin–membrane interactions in tail complexes established spontaneously. The binding of βarr2 to the C-tail of β_2_AR phosphorylated on serine residues 355 and 356^38^, present in tail and tail–core complexes, was modeled either based on the interaction pose of V2Rpp bound to βarr1 in the structure PDB 4JQI^9^ or on the β_1_AR-βarr1 structure PDB 6TKO. Thus we attempted already in the initial structures to cover the plasticity observed in the receptor–arrestin interactions^85^. This plasticity involves (1) the orientation of arrestin relative to the receptor, (2) the shape of the β-arrestin finger loop (helical in the arr1–rho and loop in β_1_AR–βarr1 and M2R–βarr1) and (3) the conformations the phosphorylated C-tail of β_2_AR adopts toward binding β-arrestin^30,85–87^. Although our models of β_2_AR–βarr2 complexes are based either on experimental structures of arrestin–GPCR complexes or on self-assembled β_2_AR–βarr2 configurations from our previous coarse-grained work^53^, both chosen to maximize structural plausibility, we cannot fully exclude bias toward nonnative free-energy minima. Therefore, the resulting ensembles should be interpreted with appropriate caution in the absence of independent experimental validation.

Mirroring the experimental membrane composition, simulations used symmetric plasma membrane mimics of either DOPC, DOPG and cholesterol in a 53:36:10 molar ratio, or DOPC:DOPG:cholesterol:PIP_2_ in a 52:35:10:3 molar ratio. Each protein–membrane system was immersed in 150 mM NaCl solution.

#### Protein structures

β_2_AR (residues D29-E362) was mutated to match the experimental construct. In detail, two methionines were replaced by threonine (M96T and M98T), three cysteines were mutated (C77V, C327S and C341L) and the glycosylation site N187 was replaced by glutamic acid. Moreover, E122, positioned inside the membrane core of the receptor, was protonated. In two of the three studied complex types, that is, tail–core and tail, β_2_AR was phosphorylated on S355 and S356, located at the C-tail^38^. Each phosphate carried a double negative charge. Both the ICL3 and residues of the C-tail of β_2_AR that were not specifically bound to β-arrestin were modeled as loops in Modeller9.19 (ref.^83^) with multiple orientations. The pose of the inverse agonist ICI in inactive β_2_AR originated from PDB 3NY8 (ref. ^84^) and the pose of the agonist adrenaline in active β_2_AR came from PDB 3SN6 (ref. ^82^).

Active βarr2 was based on the active conformation in PDB 5TV1 (ref. ^88^) comprising residues G2-P349 with mutations C17S, C60V, C126S, C141L, C151V, C243V, C252V and C270S and a new cysteine at position 267 (S267C) for the coupling of the cantilever tip. Inactive βarr2 (based on chain B from PDB 3P2D^89^) contained the same mutations across residues G2-S409, with missing residues 2–5 and 349–384 taken from the AlphaFold model^90^ of βarr2 (UniProt AF-P32121).

#### Simulation setup and workflow

The simulation setup followed our established multiscaling strategy^91^. The modeled complex were fitted on CG-presimulated and backmapped β_2_AR–βarr2 complexes, overlapping water molecules and ions were removed and the systems neutralized by addition or removal of ions. The systems were then stepwise energy minimized using the steepest descent algorithm. First for 5,000 steps freezing all protein and ligand atoms, for 5,000 steps freezing the protein backbone only and then for 5,000 steps without any restrictions. Next, velocities were generated at 310 K and a short preequilibration was conducted in an NpT ensemble (using Berendsen thermostat and barostat) for 5,000 steps using the timestep of 0.2 fs and position restraining all nonhydrogen atoms of the proteins and the ligands. In all following simulations, the simulation conditions described in detail in the following section were applied. In the first equilibration, lasting 10 ns, the nonhydrogen atoms of proteins and ligands were position restrained. The second equilibration, lasting 10 ns and position restraining the protein backbone only, was followed by production run simulation of 1–2 µs per (p)β_2_AR–βarr2 complex at 310 K. Then the system was cooled down to 300 K and simulated for 20 ns.

In simulations of membrane-embedded β_2_AR, the C-tail of β_2_AR was extended until residue 378 by alignment of presimulated Glu362-Cys378 to the C-tail of 8 conformations of membrane-embedded β_2_AR/ADR, which was taken from individual β_2_AR–βarr2 tail–core simulations in the PIP_2_ membrane after removal of βarr2 and the solvent and resolvating the system after C-tail extension. In addition, the phosphates from S355 and S356 were removed to obtain unphosphorylated β_2_AR. From all systems, PIP_2_ lipids were also removed and the total amount of neutralizing sodium atoms adjusted to obtain identical β_2_AR conformations embedded in a membrane without PIP_2_. All 32 systems were energy minimized, using the same three-step strategy as described above. Then, velocities were generated at 310 K and the systems were equilibrated for 5,000 steps using 0.2 fs timestep using position restraints on the protein. Next, 2 ns of position-restraint simulations with 2 fs timestep were performed followed by 10 ns, in which all but the C-tail of the receptor were position restraint. After that, four independent replicas of position-restraint simulations of the protein backbone were performed for each system with following conditions: (1) position restraining all backbone atoms and using velocities from the previous step; (2) position restraining all backbone atoms and generating new velocities; (3) position restraining backbone atoms of residues until residue 340 and implementing the velocities from the previous step and (4) position restraining backbone atoms of residues until residue 340 and generating new velocities. Thus, four independent production run simulations of 650 ns each for each conformation could be started. These simulations were performed at 310 K using the cufix correction^92^ to the force field to improve the sampling. βarr2-membrane simulations were initiated either with randomly rotated βarr2 in solution above a preequilibrated membrane or with βarr2 in the membrane-interacting pose from the tail complexes. After resolvation and ion insertion, the same equilibration strategy was conducted as described above.

#### Simulation conditions

MD simulations were run using GROMACS^93^ versions 2018–2021 using the all-atom CHARMM36 force field^94^ for lipids^95^ and cholesterol^96^, CHARMM36m for proteins^97^ and the TIP4p water model^98^, using a 2-fs timestep. Force-field parameters for ligands (ICI and adrenaline) were generated by CHARMM-Gui^99^. CHARMM36 is well established for MD simulations of membrane proteins, including GPCRs^81,100^. Simulations were performed at 310 K, which was controlled by the Nosé–Hoover thermostat with time constant 0.5 ps, and at 1 bar, controlled by the Parrinello–Rahman barostat^101,102^, with time constant 5 ps and compressibility of 4.5 × 10^−5^ bar^−1^ in a semiisotropic manner. Electrostatics were treated with particle mesh Ewald^103^ above a 1.2 nm cutoff, and the Lennard–Jones 12-6 potential was smoothly switched to 0 between 0.8 nm and 1.2 nm. Center of mass motion of the system was linearly removed every 100 steps. These parameters follow our previously validated settings for the CHARMM36 force field^53,104,105^.

#### Analysis of MD data

For analysis, β_2_AR was partitioned into ICL2 (residues 137–147), ICL3 (residues 237–264), C-tail (residues 341–361) and receptor core (the remaining residues, 29–136, 148–236, 265–340). βarr2 was split into the finger loop (64–78) and the remaining residues (2–63 and 79–349/409), of which the last sometimes divided into N-lobe (2–173) and C-lobe (174–349/409). To estimate the interaction energy *E*_mem_ between βarr2 and membrane, only residues 2–349 were used for both active (residues 2–349) and inactive (residues 2–409) βarr2 to ensure comparability.

C-edge binding was defined as the average probability of β_2_AR residues L192, K227 and R332 being within 0.3 nm to any membrane atom over the last 10 ns of each simulation. Finger loop binding was defined analogously for residues L69, D70, V71 and L72.

The distance *d* between the N-lobe of βarr2 and the β_2_AR core was calculated as the distance from the center of mass of the backbone of βarr2 residues 2–63 and 79–174 and the backbone of β_2_AR residues 31–60, 67–96, 103–136, 147–171, 179–186, 197–236, 265–298 and 305–326. Smaller *d* values correlate with deeper insertion of βarr2 into β_2_AR. The tilt angle *τ* is the angle between the first principal axis of βarr2 backbone and the membrane normal. If the C-lobe of βarr2 is closer to the membrane than the N-lobe, *τ* is smaller than 90°. The rotation angle *α* is the rotation of βarr2 around the membrane normal relative to the reference structure, which reflects the position of arr1 in the rho–arr1 complex (PDB 5DGY)^55^. The rotation angle *ρ* describes the rotation of βarr2 around its first principal axis relative to the membrane plane. To avoid complications resulting from the determination and switching of the second and the third principal axis, the angle was estimated as the angle between backbone atoms of βarr2 residues 336, 328, 232 and 259 and the membrane plane. (In the case of βarr1 in PDB 6TKO, the complementary residues were 343, 327, 231 and 258 and in case of arr1 in PDB 5DGY the corresponding residues were 1348, 1334, 1238 and 1265.) *ρ* is zero if the C-lobe β-sheets of βarr2 are parallel to the lipid membrane and the finger loop points toward the membrane. The last 10 ns of each simulation trace was analyzed.

In simulations of membrane-embedded β_2_AR, the analysis of β_2_AR interactions with residues of itself or the membrane was conducted residue-by-residue over the last 100 ns of the production run simulations (lasting 650 ns each). A residue is interacting with the object of interest, for example, membrane, or a different part of the protein, if any atom of these residues is found closer than 0.3 nm to any atom of the object of interest. The interaction probabilities were averaged over all 28 simulations of each simulation condition and standard error of the mean are given.

Time-dependent analyses used to assess the evolution and ensemble behavior of the all-atom MD simulations of β_2_AR–βarr2 complexes in membranes without and with PIP_2_, βarr2–membrane interactions and membrane-embedded β_2_ARs with longer C-tails are compiled in Supplementary Information (Supplementary Tables 2–4 and Supplementary Figs. 13–56).

### Statistics and reproducibility

#### Experiments

No statistical method was used to predetermine sample size. Experiments were performed in at least three biological replicates. SMFS adhesion events weaker than five times the s.d. of the experimental baseline noise (roughly 15 pN) were excluded from analysis. Further, adhesion events between βarr2 and β_2_AR occurring closer than 3 nm to the tip-sample contact point (to avoid taking into account nonspecific tip-sample interaction events) and further than 20 nm from the tip-sample contact point (to avoid taking into account binding events longer than the stretching length of the PEG-spacer and the tethered βarr2, and events occurring from macromolecular aggregates adhering to the cantilever tip) were excluded. FD curves containing multiple adhesion events were excluded as likely nonspecific. When multiple tip-sample interaction times were recorded, the acquisition of contact times was randomized. The data analysis was independently verified by a blinded researcher from outside the author list.

Height-clamp data were binned in R using the Freedman–Diaconis rule. The s.d. of each bin was calculated in R while bins were fitted in GraphPad Prism (version 9 and higher) using a nonlinear fit based on equation (1) to determine the mean ± s.d. of *t*_0_ and *x*_β_. Statistical analysis of DFS data was performed in GraphPad Prism. *P* values for *k*_off_ and *x*_β_ comparisons used the extra sum-of-squares *F* test on the Bell–Evans fits of the DFS data acquired at shorter and longer interaction times, while *P* values for binding probability of comparisons used the two-sided Mann–Whitney test.

#### MD simulations

No formal statistical power calculation was performed to predetermine sample sizes for MD simulations. Instead, sample sizes were chosen based on established practice and computational feasibility. At least eight independent MD replicas (*N* ≥ 8) per condition were generated from different initial models. This number of replicas is widely used to ensure adequate conformational sampling and assess reproducibility^106^. Convergence and robustness were evaluated by comparing key observables (for example, structural metrics, interaction frequencies) across replicas, consistent with current recommendations to emphasize ensemble-based MD simulations over single-trajectory interpretation. No simulations were excluded from analysis.

### Reporting summary

Further information on research design is available in the Nature Portfolio Reporting Summary linked to this article.

## Online content

Any methods, additional references, Nature Portfolio reporting summaries, source data, extended data, supplementary information, acknowledgements, peer review information; details of author contributions and competing interests; and statements of data and code availability are available at 10.1038/s41594-026-01842-3.

## Supplementary information

Supplementary Information Supplementary Figs. 1–56, Tables 1–4 and uncropped scans of gels.

Reporting Summary

Peer Review File

Supplementary Data 1 Source data for Supplementary Figs. 1, 4 and 6–11.

Supplementary Table Supplementary Table 1.

## Source data

Source Data Fig. 1 Statistical source data describing the example curve.

Source Data Fig. 2 Statistical source data describing the example curve.

Source Data Fig. 3 Statistical source data.

Source Data Fig. 4 Statistical source data.

Source Data Fig. 5 Statistical source data.

Source Data Extended Data Fig. 1 Statistical source data.

Source Data Extended Data Fig. 2 Statistical source data.

Source Data Extended Data Fig. 3 Statistical source data.

Source Data Extended Data Fig. 4 Statistical source data.

Source Data Extended Data Fig. 5 Statistical source data.

Source Data Extended Data Fig. 6 Statistical source data.

## Data Availability

The raw data generated in this study and used to generate the main figures, Extended Data and Supplementary Information are available from Universität Stuttgart at 10.18419/DARUS-5513. The source data for the Supplementary Information section ‘MD data analysis’ have also been deposited under the same DOI. Additional data and materials are available from the corresponding author upon reasonable request. Source data are provided with this paper.

## Code availability

In-house R scripts for FT-curve analysis, MATLAB scripts for DFS analysis and README files are available in the folder ‘AFM_Analysis_Scripts.sitx’ from the Universität Stuttgart at 10.18419/DARUS-5513.
