# Supplementary material for: Factors modulating the assembly of human β2-adrenergic receptor–β-arrestin complexes
Source: Nat Struct Mol Biol. 2026 Jul 31;33(8):1158–70. doi: 10.1038/s41594-026-01842-3 (PMC13468150; doi:10.1038/s41594-026-01842-3)
Supplement: Supplementary file 1 [file 41594_2026_1842_MOESM1_ESM.pdf]

nature structural & molecular biology

Supplementary information https://doi.org/10.1038/s41594-026-01842-3

Factors modulating the assembly of human β_2-adrenergic receptor–β-arrestin complexes

In the format provided by the authors and unedited

Supplementary Information

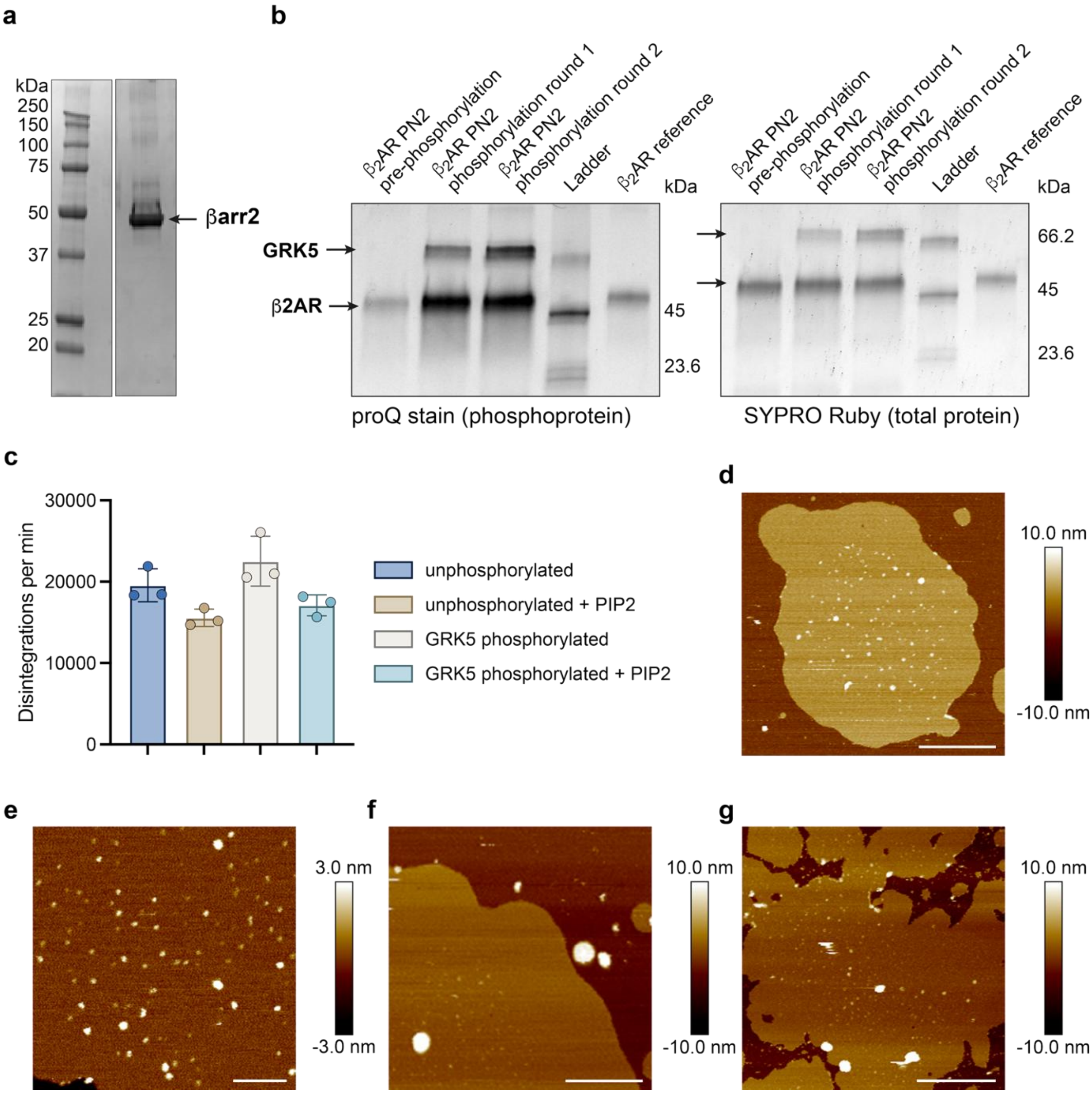

Supplementary Figure 1. Preparation and characterization of human β-arrestin2 and β_2-adrenergic receptor samples. a, SDS-PAGE gel of purified human β-arrestin2 (βarr2). **b,** SDS-PAGE gel stained with proQ (left) and Sypro Ruby (right) showing phosphoprotein and total protein, respectively, from β_2-adrenergic receptor (β_2AR) phosphorylation experiments. PeppermintStick phosphoprotein ladder (Thermo Fisher) is used. **c,** Radioligand binding to human β_2AR post reconstitution into liposomes demonstrates reconstituted receptors remain functional and are of similar abundance across samples. Liposomes were comprised of DOPC:DOPG:cholesterol (54:36:10 molar ratio) (without PIP_2) and DOPC:DOPG:cholesterol:PIP_2 (52:35:10:3 molar ratio) (with PIP_2). Bars represent the mean of three experimental replicates (data points) and error bars the SD. **d,** Atomic force microscopy (AFM) topograph of DOPC:DOPG:cholesterol liposomes with β_2AR adsorbed to atomically flat, freshly cleaved mica (dark brown). Upon attachment to mica the proteoliposomes open and adsorb as flat membranes. Receptors (white spots) are sparsely distributed and protrude from the phospholipid membrane (light brown). Scale bar, 550 nm. **e,** AFM topograph of DOPC:DOPG:cholesterol liposomes with phosphorylated β_2AR adsorbed

to atomically flat mica (brown). Proteins (white spots) are sparsely distributed and protrude from the phospholipid membrane (yellow). Scale bar, 200 nm. **f**, AFM topograph of DOPC:DOPG:cholesterol:phosphatidylinositol 4,5-bisphosphate (PIP_2) liposomes with β_2AR adsorbed to mica (brown). Proteins (white spots) are sparsely distributed and protrude from the phospholipid membrane (yellow). Scale bar, 500 nm. **g**, AFM topograph of DOPC:DOPG:cholesterol:PIP_2 liposomes with phosphorylated β_2AR adsorbed to atomically flat mica (brown). Proteins (white spots) are sparsely distributed and protrude from the phospholipid membrane (yellow). Scale bar, 500 nm.

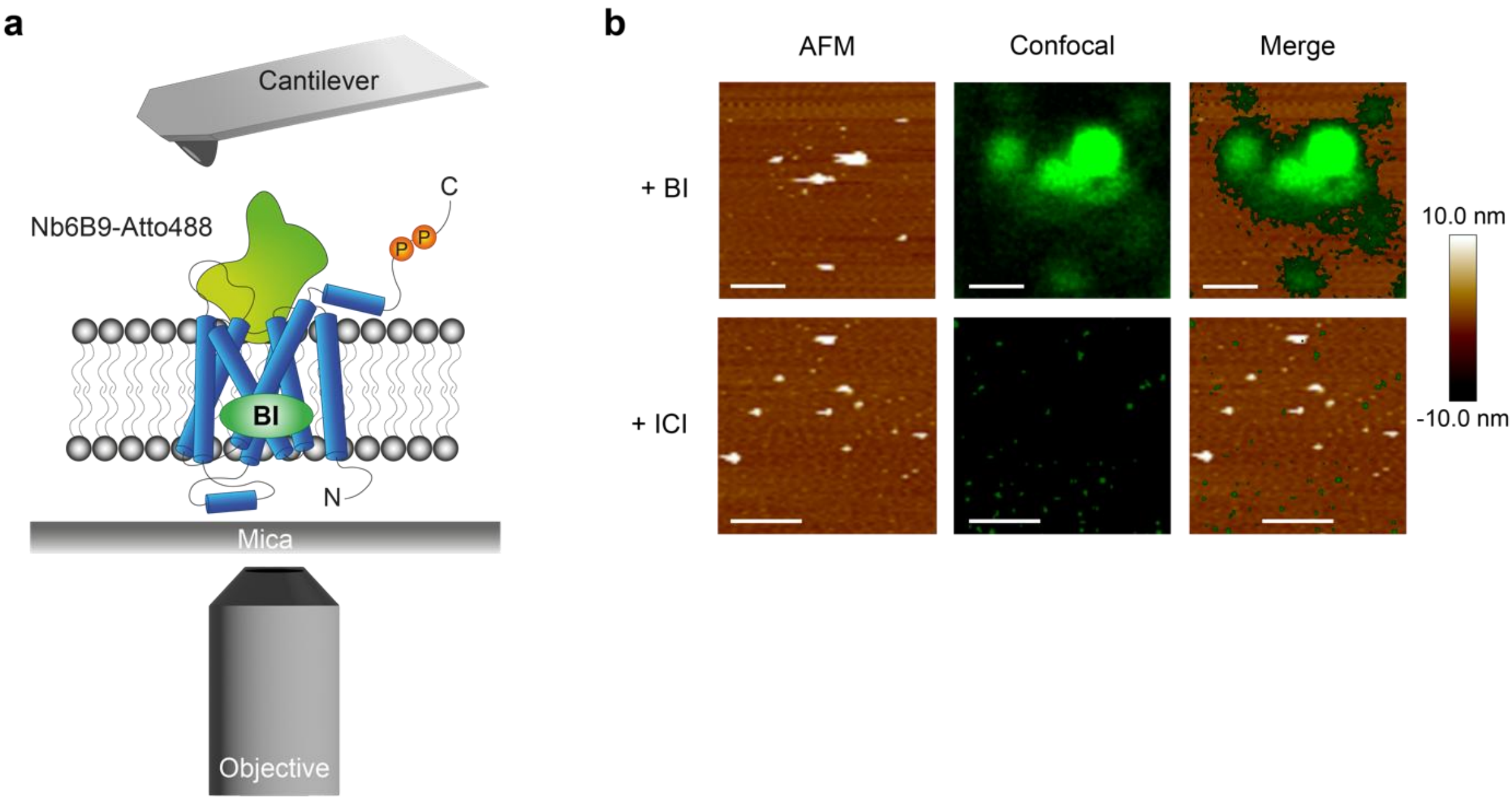

Supplementary Figure 2. β_2AR adopts active and inactive conformations in lipid membranes used for AFM-based SMFS. a, Schematic of combined AFM and confocal microscopy imaging of β_2AR membranes adsorbed to mica in buffer solution. The mica is mounted on a glass microscope slide (not shown) to enable simultaneous imaging with AFM and confocal microscopy. β_2AR activated by the full agonist BI-167107 (BI) is visualized using the nanobody Nb6B9 labelled with the fluorophore ATTO488 (Nb6B9-ATTO488). Nb6B9 specifically binds to the agonist-induced active conformation of the β_2AR and not to the β_2AR inactivated by the inverse agonist ICI-118551 (ICI). **b,** AFM topographs (left), confocal microscopy images (middle), and their merges (right) of the same area of a supported β_2AR membrane incubated with Nb6B9-ATTO488. Top, DOPC:DOPG:cholesterol membrane with β_2AR activated by BI. Bottom, DOPC:DOPG:cholesterol membrane with β_2AR inactivated by ICI. Scale bars, 500 nm. AFM topographs and confocal microscopy images are representative of three independent replicates of each condition.

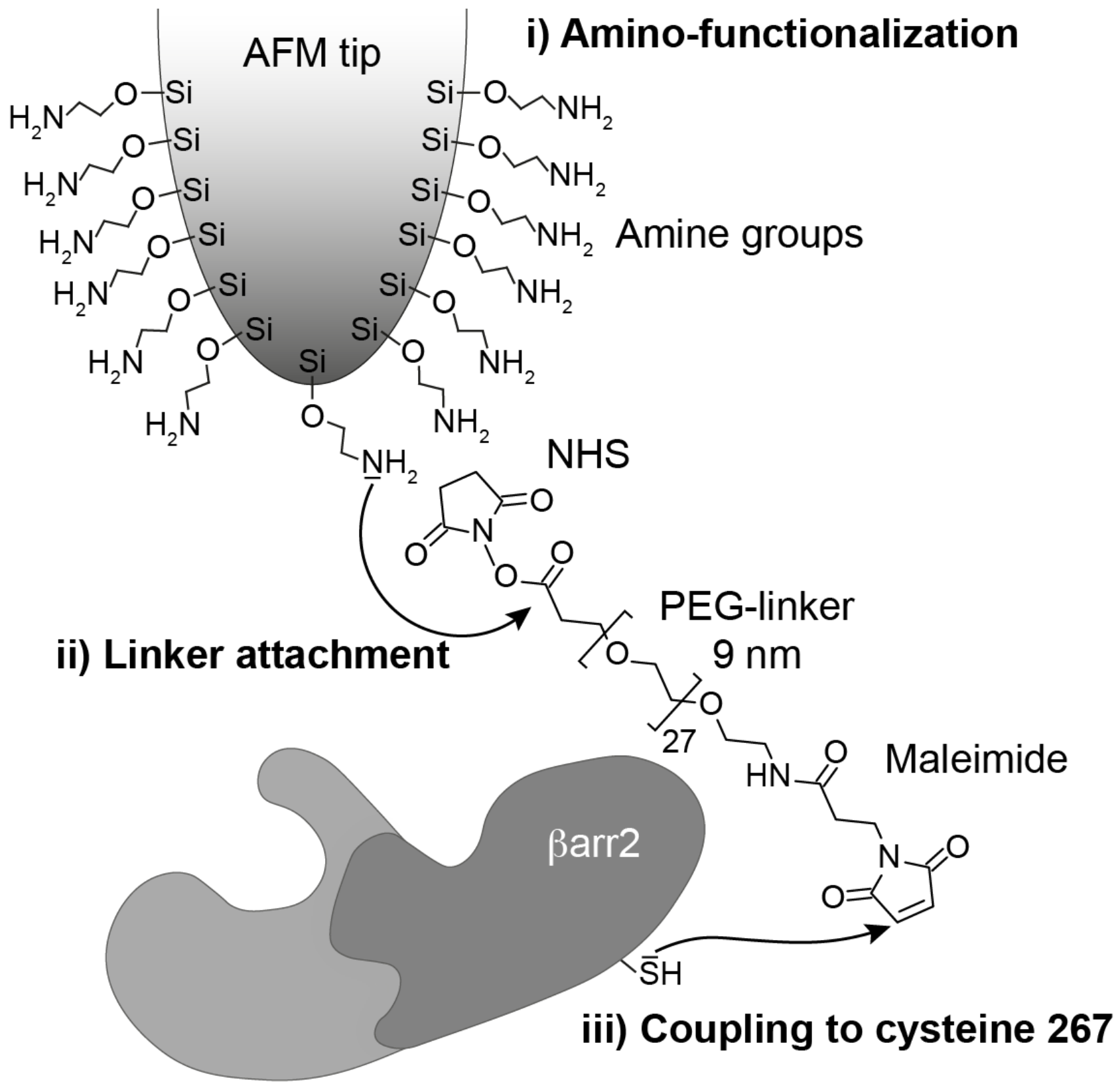

Supplementary Figure 3. Conjugation of βarr2 to the AFM tip. Shown are chemical reactions applied to covalently attach βarr2 to the tip of the AFM cantilever. In step *i)* the tip of the AFM cantilever is amino-functionalized with ethanolamine to obtain a surface functionalization with primary amines; in step *ii)* a heterobifunctional polyethylene glycol (PEG_{27})-linker is linked through the N-hydroxysuccinimide (NHS) group to primary amines; and in step *iii)* the free maleimide group of the PEG_{27}-linker reacts with the thiol group of Cys267 on the C-lobe of βarr2.

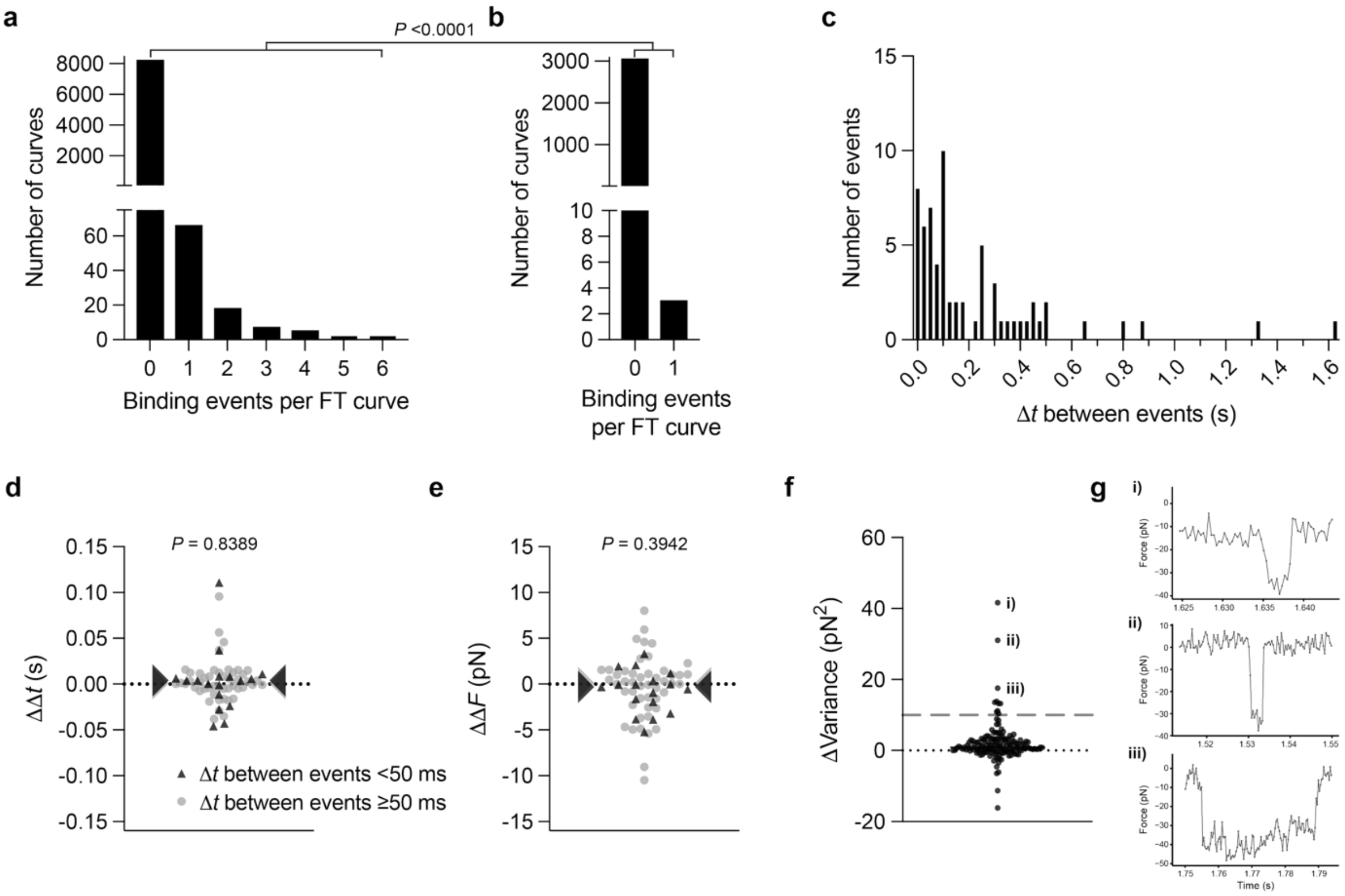

Supplementary Figure 4. Analysis of binding events and force steps in height clamp measurements. a and **b**, Number of individual binding events detected per force-time (FT) curve in (**a**) DOPC:DOPG:cholesterol membranes containing β_2AR and (**b**) DOPC:DOPG:cholesterol membranes without β_2AR. *P* values comparing both distributions in (**a**) and (**b**) are obtained by two-sided Mann–Whitney tests. **c**, Distribution of the time Δt between consecutive binding events of the same FT curves analyzed in **a**. **d** and **e**, Comparison of the difference in binding event duration $\Delta\Delta t$ (**d**) and binding force $\Delta\Delta F$ (**e**) between two consecutive binding events separated by < 50 ms (black triangles) and ≥ 50 ms (grey circles). $\Delta\Delta t$ and $\Delta\Delta F$ were calculated by subtracting the duration/change in force of the preceding (un-)binding event from the duration/change in force of the following binding event. *P* values comparing the datasets with Δt between binding events < 50 ms and ≥ 50 ms in (**d**) and (**e**) are obtained from two-sided Mann–Whitney tests. **f**, Difference of the variance of the force of a binding event if a single force step was assumed (one degree of freedom, *i.e.*, one ΔF) and if multiple force steps were assumed (three degrees of freedom, *i.e.*, one ΔF for every step and Δt between steps). The dotted line marks no difference in variance and the dashed line marks a difference in variance of 10 pN^2. **g**, Examples of single binding events detected in FT curves. *i)*, *ii)*, and *iii)* show the single events with the largest difference in variance, *i.e.*, the highest probability of multiple force steps. Despite the lower variance when considering multiple (un-)binding steps, it is difficult to distinguish any possible substep from noise or drift within an event. The data analyzed was taken from Fig. 1.

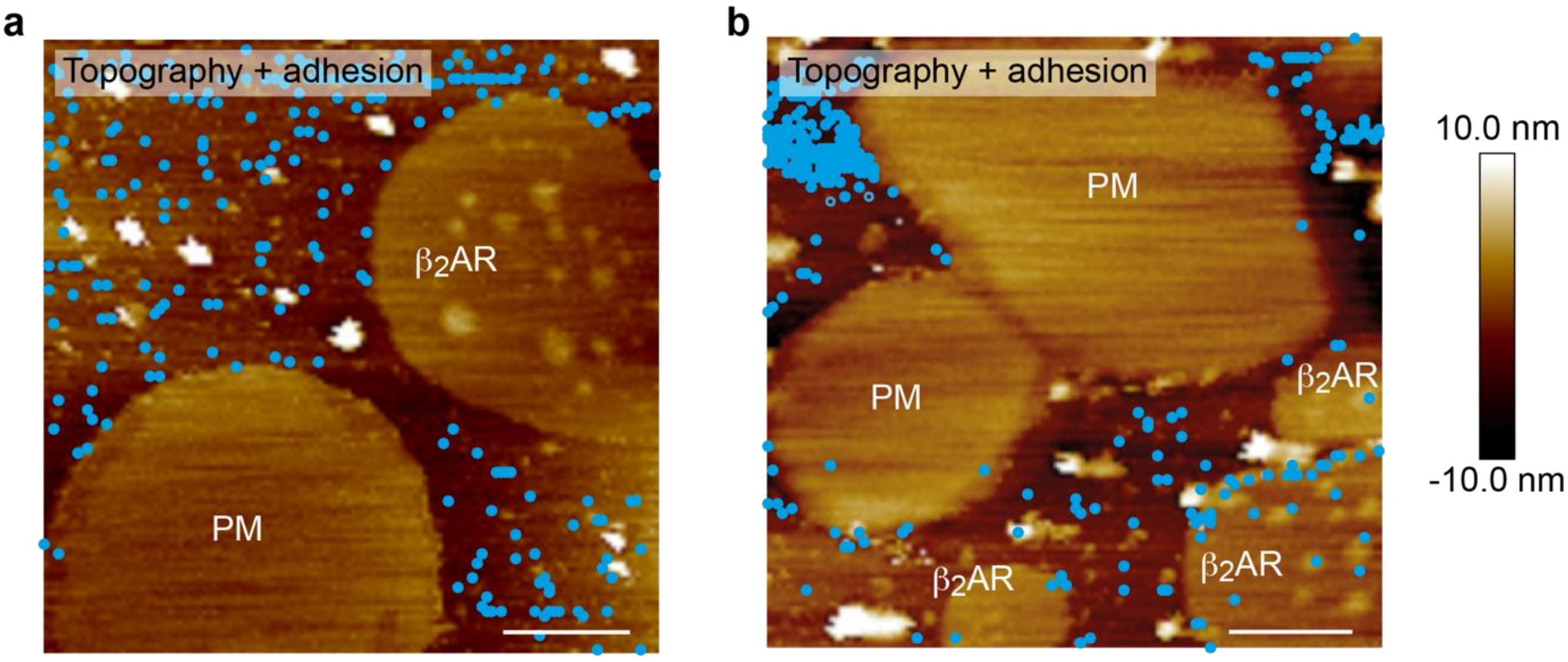

Supplementary Figure 5. βarr2-functionalized cantilever tips specifically bind β_2AR. a, FD curve-based AFM topograph (brown) and overlaid nonspecific adhesion events (blue dots) recorded with cantilever tips functionalized only with PEG_{27}-linker (see Supplementary Fig. 3). Nonspecific adhesion events detected locate at the mica support but not with phosphorylated, activated β_2AR protruding from the DOPC:DOPG:cholesterol membrane nor with purple membrane (PM), comprising the light-driven proton pump bacteriorhodopsin. The topograph was recorded collecting FD curves at a retraction speed of 1090 nm s^{-1} and a tip-sample interaction time of ≈ 21 ms. The data is representative of 10 FD curve based AFM measurements using two functionalized AFM tips and retraction speeds ranging from 501 nm s^{-1} to 6510 nm s^{-1}. Adhesion events (blue dots) were detected in FD curves recorded of the topograph. Adhesion events occurring within the nonspecific tip-sample distance of <3 nm and above the length of the stretched PEG_{27}-linker of >20 nm were excluded from analysis[1]. Scale bar, 200 nm. **b,** FD curve-based AFM topograph (brown) and overlaid adhesion events (blue dots) recorded with βarr2-functionalized cantilever tips (see Supplementary Fig. 3). While specific adhesion events locate with phosphorylated, activated β_2AR protruding from the DOPC:DOPG:cholesterol membrane, no such events were detected on purple membrane. Nonspecific adhesion events were detected with the supporting mica between the membranes. The topograph was recorded collecting FD curves at a retraction speed of 1090 nm s^{-1} and a tip-sample interaction time of ≈ 21 ms. The example is representative of seven FD curve based AFM measurements conducted with two functionalized AFM tips at retraction speeds ranging from 501 nm s^{-1} to 6510 nm s^{-1}. Adhesion events occurring within the nonspecific tip-sample distance of <3 nm and above the length of the PEG_{27}-linker of >20 nm were excluded from analysis[1]. Scale bars, 180 nm.

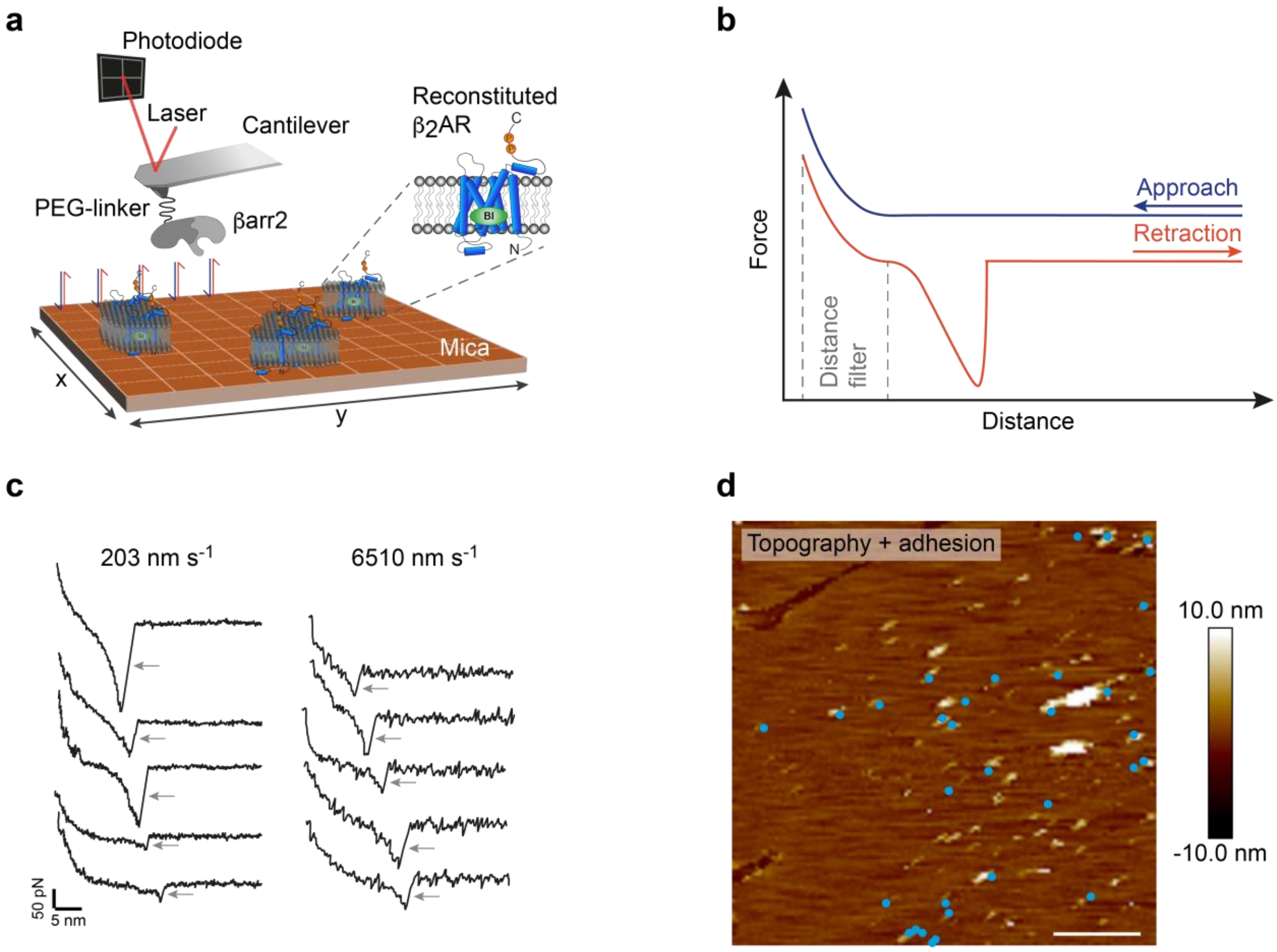

Supplementary Figure 6. Detection of specific (un-)binding events of β$_2$AR and βarr2 by AFM-based single-molecule force spectroscopy (SMFS). a, For every pixel of the FD curve-based AFM topograph, the βarr2-functionalized cantilever tip approaches to and retracts from the sample. The sample consists of the supporting mica that is covered with phospholipid membranes containing β$_2$ARs. The cantilever tip is approached to the sample until reaching a force of 100 pN. Subsequently, to separate tip and sample, the cantilever is retracted at predefined speeds ranging from 203 nm s^{-1} to 6510 nm s^{-1}. During each approach and retraction cycle, a force-distance (FD) curve is recorded (exemplified in **b**). The time between approach and retraction of the functionalized tip can be varied to adjust the interaction time between β$_2$AR and βarr2. In this work, we used delay times of 0 ms and 100 ms, resulting in average interaction times of 21 ± 2 ms and 121 ± 2 ms, respectively. A single topograph samples up to 16,384 pixels and for every pixel records an FD curve. All experiments are recorded in buffer solution at room temperature. **b,** Illustration of an approach (blue) and a retraction (red) FD curve. The retraction curve shows an unbinding event between β$_2$AR and βarr2 at a distance that corresponds to the stretching of the flexible PEG$_{27}$-linker. A distance filter is used to remove non-specific (un-)binding events occurring at a distance much shorter (<3 nm) or longer (>20 nm) than the length of the PEG$_{27}$-linker. **c,** Exemplary FD curves recorded during retraction of the cantilever tip that display unbinding events of β$_2$AR and βarr2 at retraction speeds of 203 nm s^{-1} (left) and 6510 nm s^{-1} (right), respectively. Unbinding events are indicated by arrows. **d,** FD curve-based AFM topograph of reconstituted ligand-activated and phosphorylated β$_2$AR recorded with a βarr2-functionalized cantilever tip at ≈ 21 ms interaction time. The topograph is overlaid with (un-)binding events detected between βarr2 and β$_2$ARs (blue dots) that were filtered with a distance filter (see **c**) to exclude non-specific events. Scale bar, 200 nm.

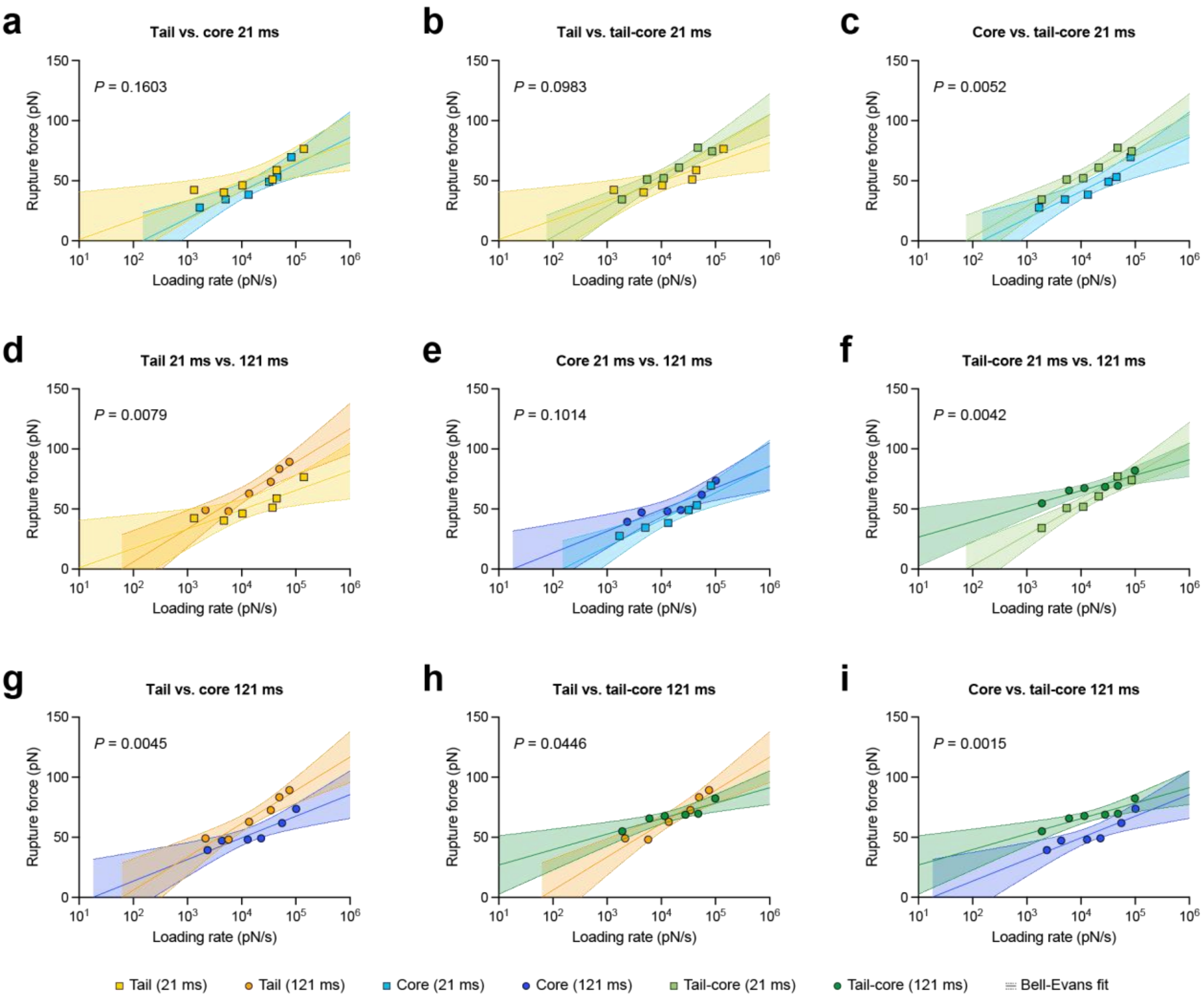

Supplementary Figure 7. Statistical comparison of DFS data obtained from β$_2$AR-βarr2 tail, core, and tail-core complexes in DOPC:DOPG:cholesterol membranes. Comparison of β$_2$AR-βarr2 **a**) tail with core complex, **b**) tail with tail-core, and **c**) core with tail-core complexes at ≈ 21 ms interaction time and of **d**) tail, **e**) core, and **f**) tail-core complexes at ≈ 21 ms and ≈ 121 ms interaction time. Comparisons of β$_2$AR-βarr2 **g**) tail with core, **h**) tail with tail-core, and **i**) core with tail-core complexes at ≈ 121 ms interaction time. Statistical differences were estimated between pairs of DFS data fit with the Bell–Evans model (line shows the mean, shaded area represents ± 95% CI). *P* values are calculated by applying the extra sum-of-squares *F* test to the Bell–Evans fit of DFS data acquired at ≈ 21 ms and ≈ 121 ms interaction time.

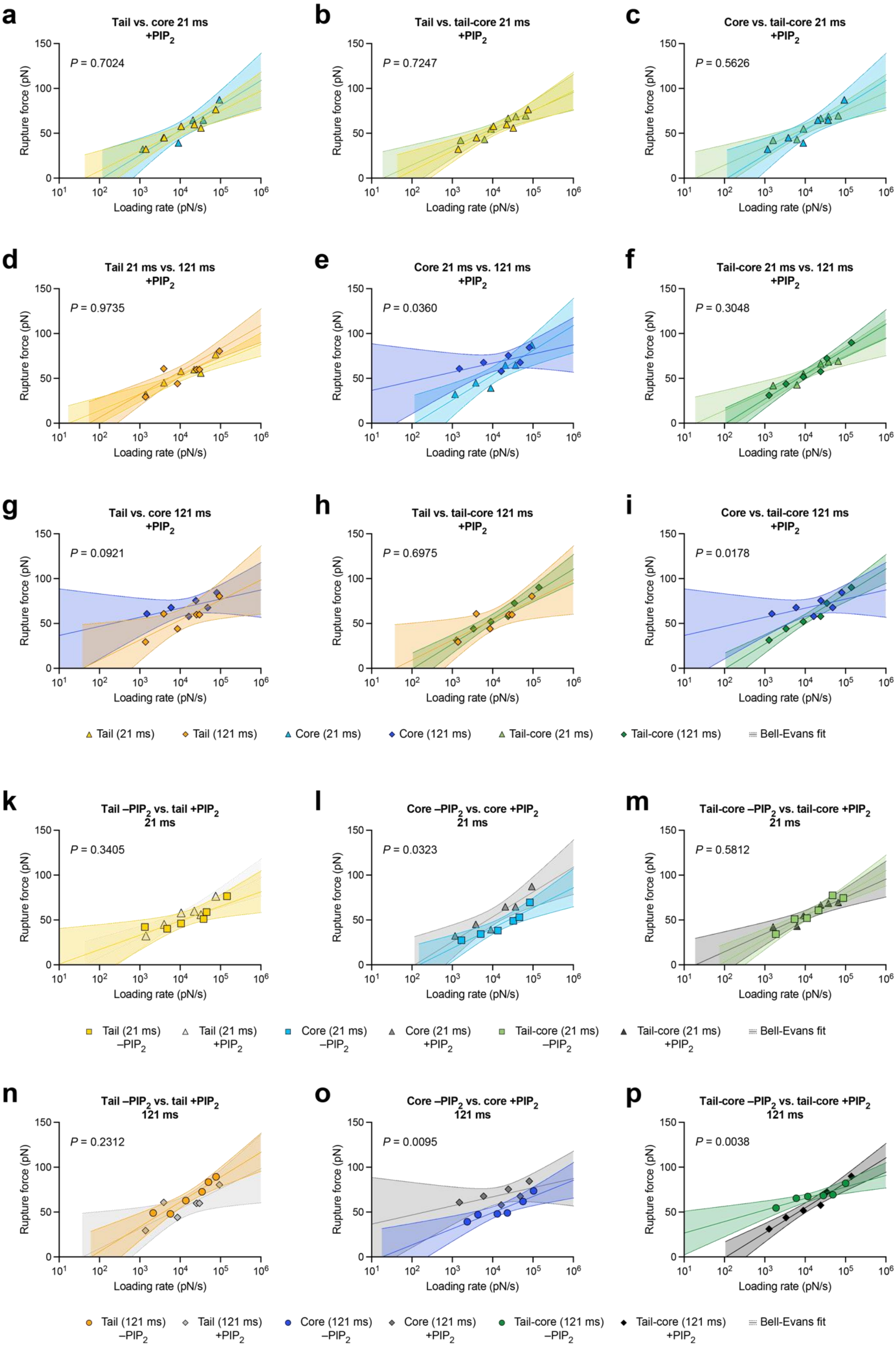

a
Tail vs. core 21 ms +PIP2
P = 0.7024
b
Tail vs. tail-core 21 ms +PIP2
P = 0.7247
c
Core vs. tail-core 21 ms +PIP2
P = 0.5626
d
Tail 21 ms vs. 121 ms +PIP2
P = 0.9735
e
Core 21 ms vs. 121 ms +PIP2
P = 0.0360
f
Tail-core 21 ms vs. 121 ms +PIP2
P = 0.3048
g
Tail vs. core 121 ms +PIP2
P = 0.0921
h
Tail vs. tail-core 121 ms +PIP2
P = 0.6975
i
Core vs. tail-core 121 ms +PIP2
P = 0.0178
Rupture force (pN)
Loading rate (pN/s)
Tail (21 ms)
Tail (121 ms)
Core (21 ms)
Core (121 ms)
Tail-core (21 ms)
Tail-core (121 ms)
Bell-Evans fit
k
Tail –PIP2 vs. tail +PIP2 21 ms
P = 0.3405
l
Core –PIP2 vs. core +PIP2 21 ms
P = 0.0323
m
Tail-core –PIP2 vs. tail-core +PIP2 21 ms
P = 0.5812
Tail (21 ms) –PIP2
Tail (21 ms) +PIP2
Core (21 ms) –PIP2
Core (21 ms) +PIP2
Tail-core (21 ms) –PIP2
Tail-core (21 ms) +PIP2
Bell-Evans fit
n
Tail –PIP2 vs. tail +PIP2 121 ms
P = 0.2312
o
Core –PIP2 vs. core +PIP2 121 ms
P = 0.0095
p
Tail-core –PIP2 vs. tail-core +PIP2 121 ms
P = 0.0038
Tail (121 ms) –PIP2
Tail (121 ms) +PIP2
Core (121 ms) –PIP2
Core (121 ms) +PIP2
Tail-core (121 ms) –PIP2
Tail-core (121 ms) +PIP2
Bell-Evans fit

Supplementary Figure 8. Statistical comparison of DFS data obtained from β_2AR-βarr2 tail, core, and tail-core complexes in the presence and in the absence of PIP_2. Comparison of **a**) β_2AR-βarr2 tail with core complex, **b**) β_2AR-βarr2 tail with tail-core, and **c**) β_2AR-βarr2 core with tail-core complexes at ≈ 21 ms interaction time and of **d**) tail, **e**) core, and **f**) tail-core complexes at ≈ 21 ms and ≈ 121 ms interaction time. Comparisons of β_2AR-βarr2 complexes at ≈ 121 ms interaction time are shown for **g**) tail with core, **h**) tail with tail-core, and **i**) core with tail-core complexes. Data obtained in the presence of PIP_2 are compared to the equivalent data obtained in the absence of PIP_2 for **k**) tail, **l**) core, and **m**) tail-core complexes at ≈ 21 ms interaction time as well as for **n**) tail, **o**) core, and **p**) tail-core complexes at ≈ 121 ms interaction time. Statistical differences were estimated between pairs of DFS data fit with the Bell–Evans model (line shows the mean, shaded area represents ± 95% CI). *P* values are calculated by applying the extra sum-of-squares *F* test to the Bell–Evans fit of the DFS data.

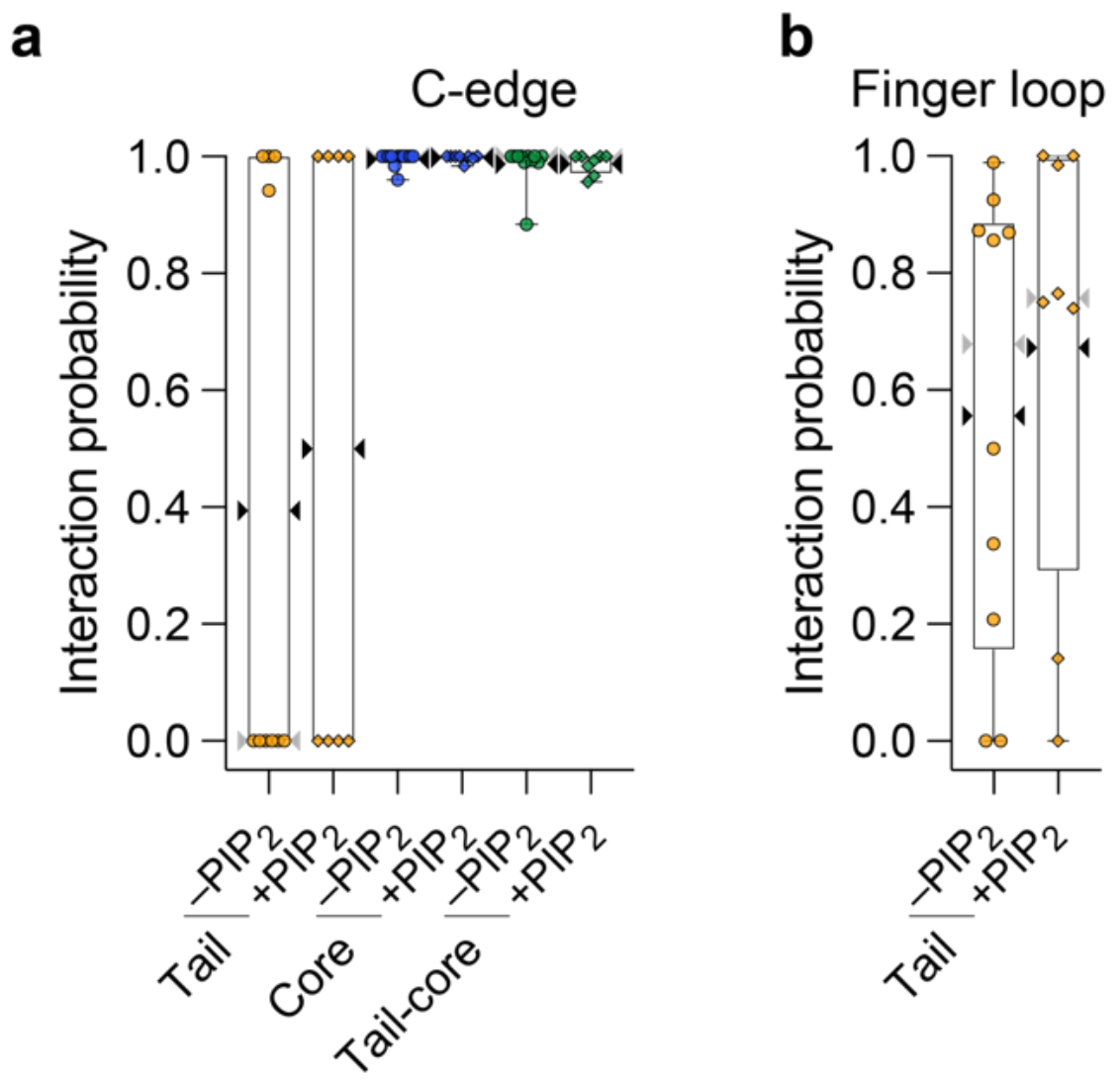

Supplementary Figure 9. Interaction probability of βarr2 C-edge and finger loop with phospholipid membranes in β_2AR-βarr2 equilibrated MD simulations. a, Interaction probability of βarr2 C-edge with –PIP_2 and +PIP_2 membranes estimated as the average interaction probability of residues L192, K227, and R332 of βarr2 with the membrane. **b**, Interaction probability of βarr2 finger loop with –PIP_2 and +PIP_2 membranes estimated as the average interaction probability of residues L69, D70, V71, and L72 of βarr2 with the membrane. Data are shown as boxplots where boxes mark the 25th to 75th percentiles, whiskers show minimum and maximum values, and median and mean are indicated by grey and black arrows, respectively. Data points represent the average interaction probability over the last 10 ns of individual equilibration all-atom simulations.

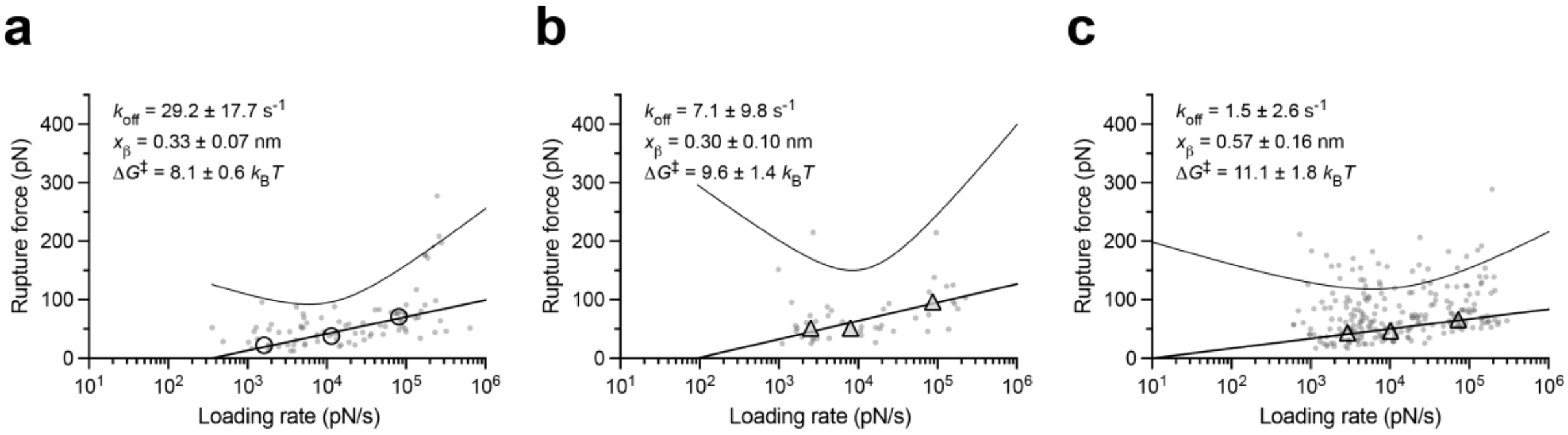

Supplementary Figure 10. DFS data of inactive and active βarr2 interacting with membranes with and without PIP2. a-c, DFS data of interactions of V2Rpp-activated βarr2 with a membrane without PIP2 (**a**), inactive βarr2 with a PIP_2-containing membrane (**b**), and V2Rpp-activated βarr2 with a PIP_2-containing membrane (**c**) measured at 21 ms. DFS data are binned (circles, up-triangles) for each retraction speed (Methods). The Bell–Evans model[2] fit to the binned data is shown as mean (line) ± 95% CI.

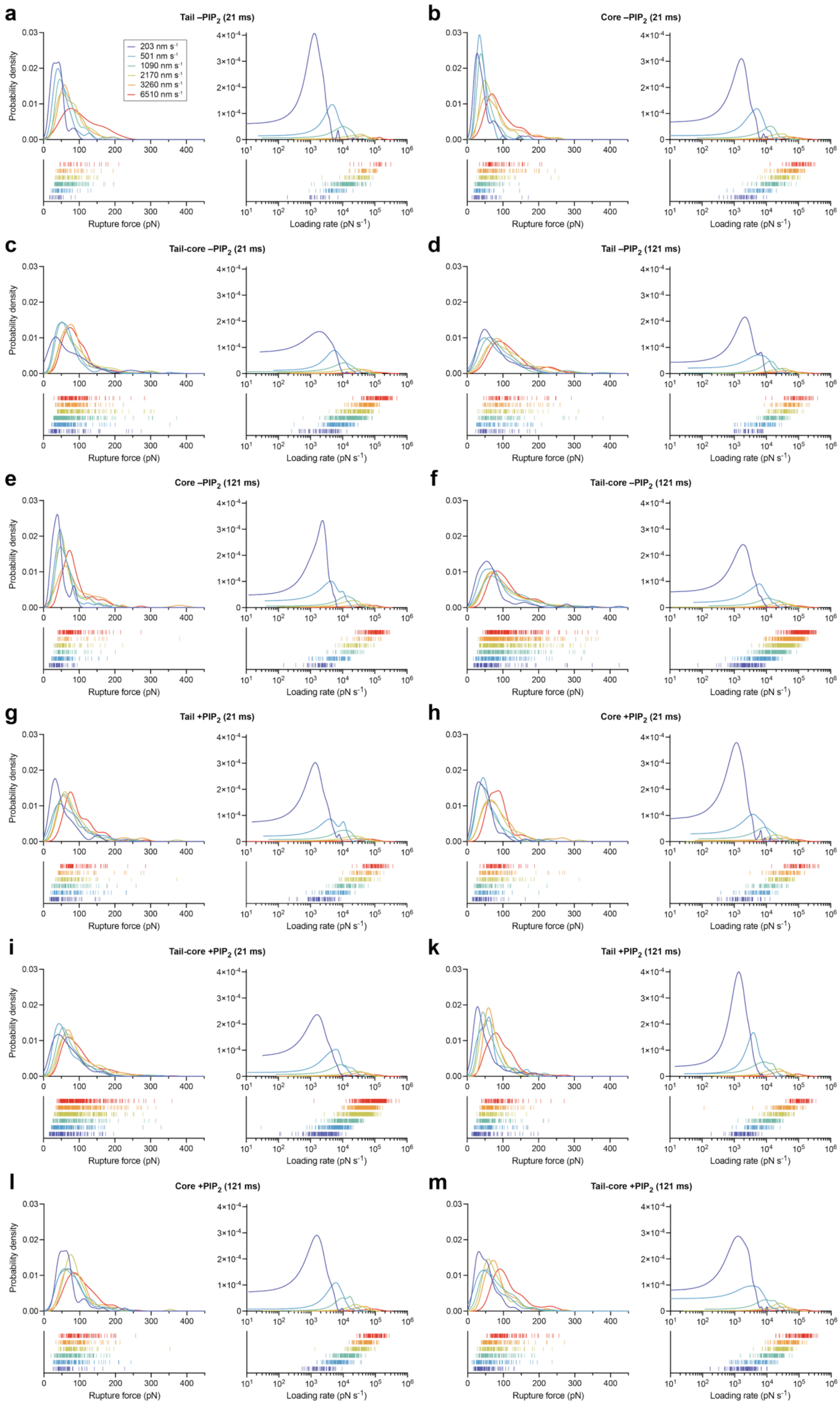

a
Tail –PIP2 (21 ms)
b
Core –PIP2 (21 ms)
c
Tail-core –PIP2 (21 ms)
d
Tail –PIP2 (121 ms)
e
Core –PIP2 (121 ms)
f
Tail-core –PIP2 (121 ms)
g
Tail +PIP2 (21 ms)
h
Core +PIP2 (21 ms)
i
Tail-core +PIP2 (21 ms)
k
Tail +PIP2 (121 ms)
l
Core +PIP2 (121 ms)
m
Tail-core +PIP2 (121 ms)
203 nm s-1
501 nm s-1
1090 nm s-1
2170 nm s-1
3260 nm s-1
6510 nm s-1
Probability density
Rupture force (pN)
Loading rate (pN s-1)

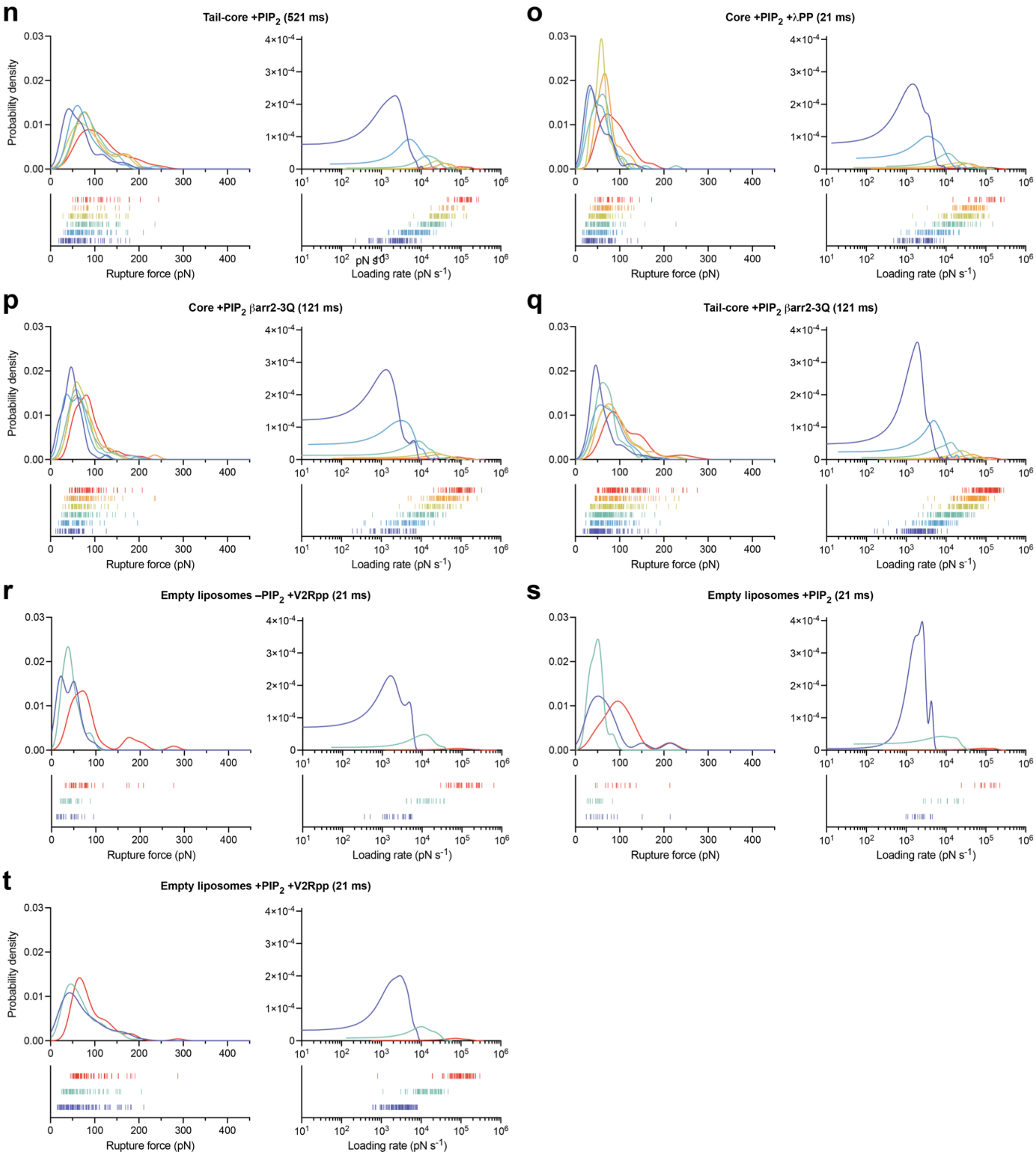

Supplementary Figure 11. Kernel density estimation (KDE) of DFS datasets. a-f, KDEs of β$_2$AR-βarr2 complexes measured in the absence of PIP$_2$ (–PIP$_2$) at 21 ms for (**a**) tail, (**b**) core, and (**c**) tail-core complexes and at 121 ms for (**d**) tail, (**e**) core, and (**f**) tail-core complexes. **g-q**, KDEs of β$_2$AR-βarr2 complexes measured in the presence of PIP$_2$ (+PIP$_2$) at 21 ms for (**g**) tail, (**h**) core, and (**i**) tail-core complexes, at 121 ms for (**k**) tail, (**l**) core, and (**m**) tail-core complexes, at 521 ms for (**n**) the tail-core complex, and (**o**) at 21 ms for the core complex treated with lambda protein phosphatase (+λPP). KDEs measured with βarr2-3Q at 121 ms for (**p**) core and (**q**) tail-core complexes. **r-t**, KDEs of βarr2-membrane interactions at 21 ms with (**r**) V2Rpp –PIP2, (**s**) without V2Rpp +PIP$_2$, and (**t**) with V2Rpp +PIP$_2$. The top graphs show kernel density estimations (KDEs), while the bottom graphs show the distribution of rupture force (left) and loading rate (right) separated by the retraction speed. DFS data were taken from Fig. 2 and 3, Extended Data Fig. 3, 4, and 5, and Supplementary Fig. 10.

a

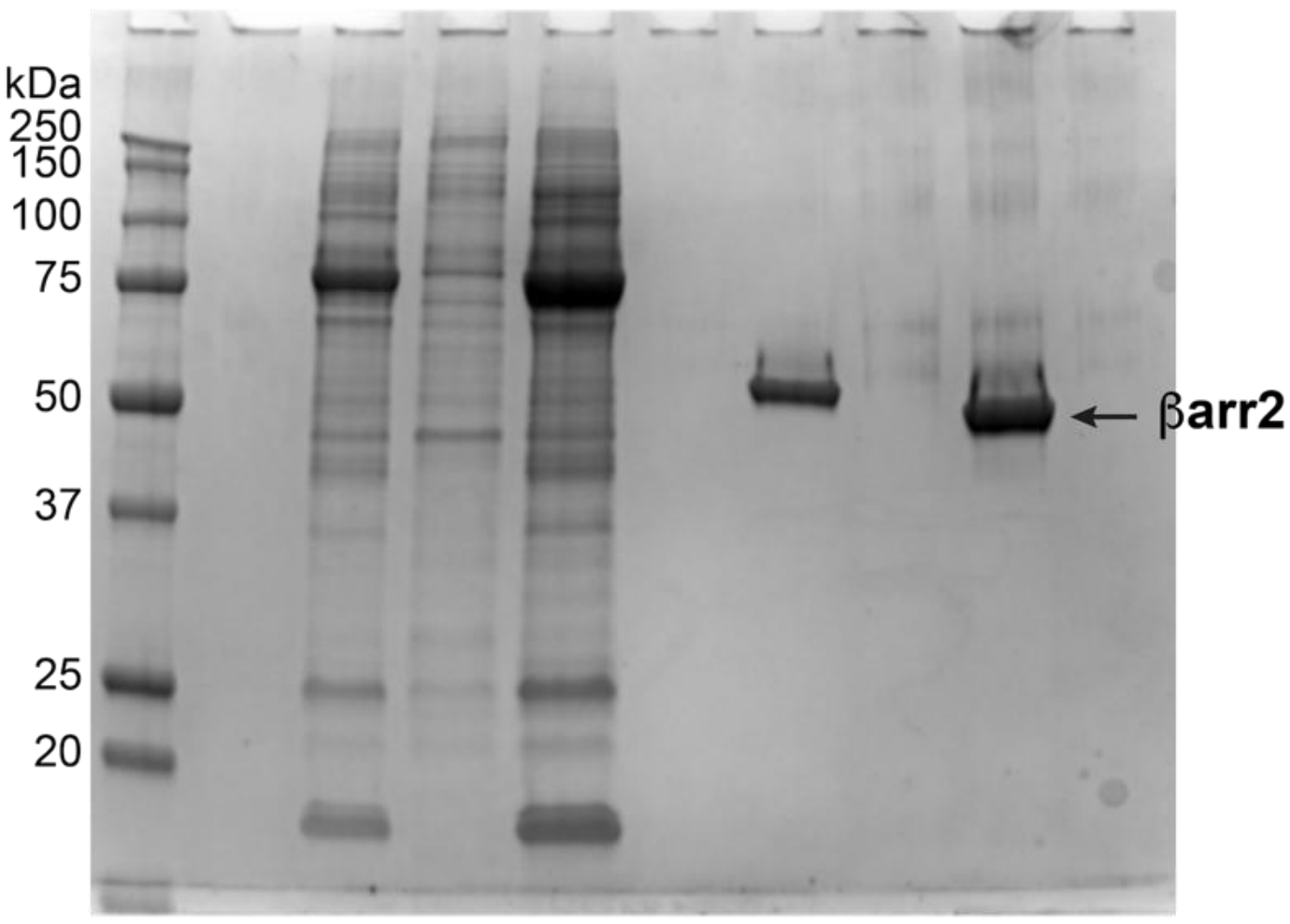

b

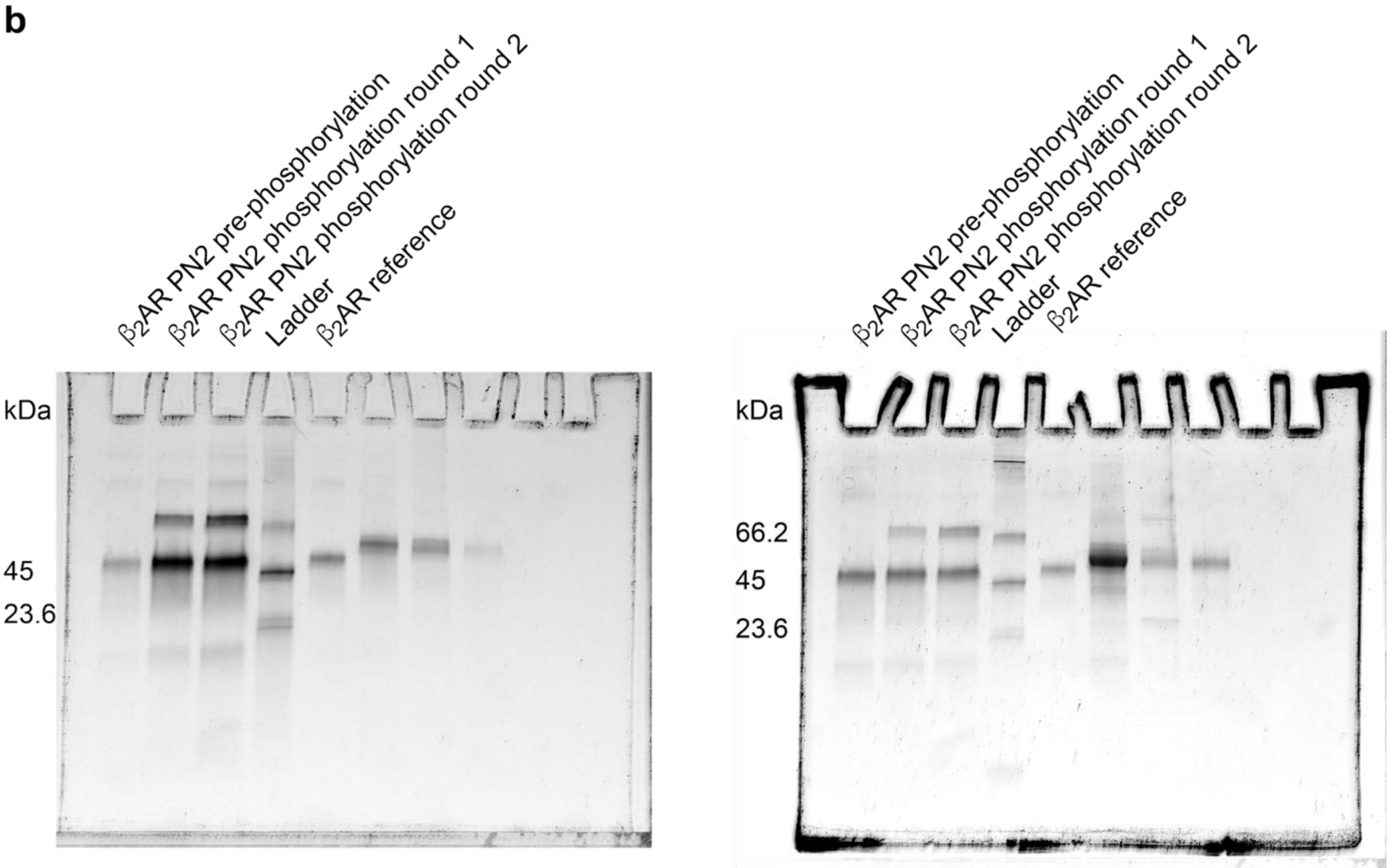

Supplementary Figure 12. Uncropped scans of SDS-PAGE gels presented in Supplementary Figure 1. a, SDS-PAGE gel of purified human β-arrestin2 (βarr2). Purified βarr2 is shown in the second lane from the right. **b**, SDS-PAGE gel stained with proQ (left) and Sypro Ruby (right) showing phosphoprotein and total protein from β_2-adrenergic receptor (β_2AR) phosphorylation experiments. PeppermintStick phosphoprotein ladder (Thermo Fisher) is used.

Supplementary Table 1. Summary of performed all-atom MD simulations. βarr2* denotes βarr2 in the active state. [a] Simulations were initiated from the tail-core complexes by deletion of the adrenaline-bound receptor. [b] In the spontaneous binding simulations of active βarr2* to the membrane, the active state of βarr2* was stabilized by the C-tail of β_2AR (residues L342–E362) bearing phosphorylation on residues S355 and S356. pβ_2AR/ADR denotes receptor (residues D29–A378) phosphorylated on S355 and S356 and activated by adrenaline. In β_2AR/ADR the two phosphorylation sites are removed. The total simulation time amounts to ≈ 164 µs.

**βarr2–β_2AR complexes**	**Production runs**
Tail –PIP_2	10 x 1 µs
Core –PIP_2	12 x 1 µs
Tail-core –PIP_2	10 x 1 µs, 1 x 2 µs
Tail +PIP_2	8 x 1 µs
Core +PIP_2	8 x 1 µs
Tail-core +PIP_2	7 x 1 µs, 1 x 2 µs
**Membrane binding of βarr2**	
βarr2 –PIP_2	18 x 0.5 µs
βarr2 +PIP_2	18 x 0.5 µs
βarr2* –PIP_2	8 x 0.2 µs[a]; 10 x 0.5 µs[b]
βarr2* +PIP_2	4 x 0.2 µs[a]; 14 x 0.5 µs[b]
**Membrane-embedded β_2AR**	
pβ_2AR/ADR +PIP_2	28 x 0.65 µs
pβ_2AR/ADR –PIP_2	28 x 0.65 µs
β_2AR/ADR +PIP_2	28 x 0.65 µs
β_2AR/ADR –PIP_2	28 x 0.65 µs
**Total**	**164.2 µs**

MD data analysis

Simulation system sizes and compositions

Complex name	Proteins and ligands	Membrane composition	Box size<br>Box shape	Solvent	Number of replicas and simulation length
Tail<br>-PIP_2	pβ_2AR,ICI,<br>βarr2*	250 DOPC,<br>166 DOPG,<br>46 Chol	13.3x11.5x16.7 nm^3<br>hexagonal	~ 60 000,<br>232 Na^+,<br>83 Cl^-	10 x 1 µs
Tail<br>+PIP_2	pβ_2AR,ICI,<br>βarr2*	254 DOPC,<br>170 DOPG,<br>48 Chol,<br>18 DOPI	13.5x11.7x12.7 nm^3<br>hexagonal	~ 43 000<br>water, 223<br>Na^+, 6 Cl^-	8 x 1 µs
Core<br>-PIP_2	β_2AR*, ADR,<br>βarr2*	248 DOPC,<br>164 DOPG,<br>44 Chol	13.2x11.4x15.1 nm^3<br>hexagonal	53 000,<br>214 Na^+,<br>71 Cl^-	12 x 1 µs
Core +PIP_2	β_2AR*, ADR,<br>βarr2*	283 DOPC,<br>188 DOPG,<br>54 Chol,<br>14 DOPI	14.2x12.3x13.9 nm^3	~55 000<br>water, 254<br>Na^+, 27 Cl^-	8 x 1 µs
Tail-core<br>-PIP_2	pβ_2AR*, ADR,<br>βarr2*	248 DOPC,<br>164 DOPG,<br>44 Chol	13.2x11.4x15.2 nm^3<br>hexagonal	~ 53 000<br>water, 217<br>Na^+, 70 Cl^-	10 x 1 µs<br>1 x 2 µs
Tail-core<br>+PIP_2	pβ_2AR*, ADR,<br>βarr2*	283 DOPC,<br>189 DOPG,<br>54 Chol,<br>14 DOPI	14.4x12.5x13.4 nm^3	~ 55 000<br>water, 255<br>Na^+, 23 Cl^-	7 x 1 µs<br>1 x 2 µs

Supplementary Table 2. Characteristics and composition of MD simulations of membrane-embedded β arr2/**β2AR complexes.** β 2AR* stands for active β 2AR (residues 29‑362, neutral termini), p β 2AR*-longer is active, phosphorylated β 2AR (residues 29‑362, neutral termini, phosphorylation on S355 and S356), ADR denotes adrenaline (total charge +1), ICI is β 2AR-selective inhibitor ICI-118551 (total charge +1. β arr2* is β arr2 (residues 2‑349, neutral termini) in active conformation. DOPI is PIP2 (called DOPI25 in CHARMM) with total charge of ‑4.

Proteins	Membrane composition	Box size<br>Box shape	Solvent	Number of replicas and simulation length
βarr2*,C-tailpp	250 DOPC,<br>166 DOPG,<br>46 Chol	12.8x11.1x16.6 nm^3<br>hexagonal	~60 000 water, 228 Na^+, 72 Cl^-	10 x 500 ns
βarr2*, C-tailpp	254 DOPC,<br>170 DOPG,<br>48 Chol,<br>16 DOPI	13.2x11.5x16.1 nm^3<br>hexagonal	~60 000 water, 252 Na^+, 28 Cl^-	14 x 500 ns
βarr2	254 DOPC,<br>170 DOPG,<br>48 Chol,<br>16 DOPI	13.3x11.5x16.0 nm^3<br>hexagonal	~60 000 water, 258 Na^+, 24 Cl^-	17 x 500 ns,<br>1 x 200 ns
βarr2	250 DOPC,<br>166 DOPG,<br>46 Chol	12.9x11.1x16.6 nm^3<br>hexagonal	~60 000 water, 234 Na^+, 68 Cl^-	18 x 500 ns
βarr2*	250 DOPC,<br>166 DOPG,<br>46 Chol	12.9x11.21x17.1 nm^3<br>hexagonal	~62 000 water, 234 Na^+, 80 Cl^-	8 x 200 ns
βarr2*	254 DOPC,<br>170 DOPG,<br>48 Chol,<br>16 DOPI	13.3x11.5x12.9 nm^3<br>hexagonal	~44 000 water, 225 Na^+, 3 Cl^-	4 x 200 ns

Supplementary Table 3. Characteristics and composition of MD simulations of βarr2-membrane interactions. βarr2* stands for βarr2 (residues 2–349, neutral termini) in active conformation, βarr2 is inactive βarr2 (residues 2–409, neutral termini), C-tailpp denotes pβ_2AR C-tail (residues 342–362, phosphorylated on S355 and S356, neutral termini). DOPI is PIP_2 (called DOPI25 in CHARMM) with total charge of –4.

Proteins and ligands	Membrane composition	Box size<br>Box shape	Solvent	Number of replicas and simulation length
β_2AR*-longer/ADR	270 DOPC,<br>184 DOPG,<br>52 Chol,<br>14 DOPI	14.1x12.2x12.7 nm^3<br>hexagonal	~50 000 water,<br>247 Na^+, 14 Cl^-	28 x 650 ns
pβ_2AR*-longer/ADR	270 DOPC,<br>184 DOPG,<br>52 Chol,<br>14 DOPI	14.1x12.2x12.7 nm^3<br>hexagonal	~50 000 water,<br>249 Na^+, 12 Cl^-	28 x 650 ns
β_2AR*-longer/ADR	270 DOPC,<br>184 DOPG,<br>52 Chol	14.0x12.1x12.7 nm^3<br>hexagonal	~50 000 water,<br>191 Na^+, 14 Cl^-	28 x 650 ns
pβ_2AR*-longer/ADR	270 DOPC,<br>184 DOPG,<br>52 Chol,	14.0x12.1x12.7 nm^3<br>hexagonal	~50 000 water,<br>193 Na^+, 15 Cl^-	28 x 650 ns

Supplementary Table 4. Characteristics and composition of MD simulations of membrane-embedded β_2AR. β_2AR*-longer stands for active β_2AR (residues 29–378, neutral termini), pβ_2AR*-longer is active, phosphorylated β_2AR (residues 29–378, neutral termini, phosphorylation on S355 and S356), ADR denotes adrenaline (total charge +1). DOPI is PIP_2 (called DOPI25 in CHARMM) with total charge of –4.

Time-dependent analysis of all-atom MD simulations of β$_2$AR/βarr2 complexes

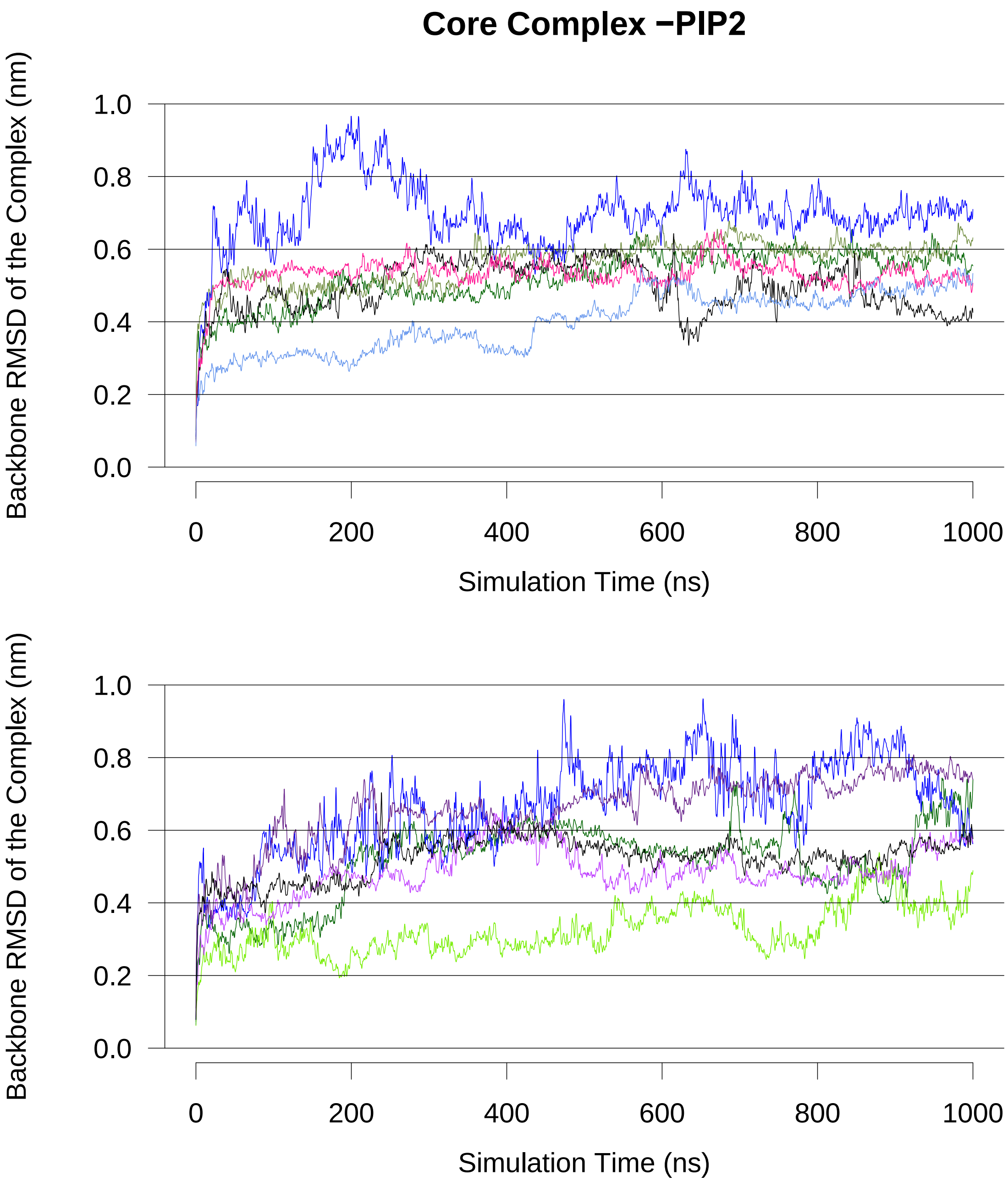

Supplementary Figure 13. Root mean square deviation (RMSD) over time of backbone atoms of β$_2$AR/βarr2 core type complexes in a membrane without PIP$_2$. No residues were excluded from the analysis, the complex was fitted on Cα atoms of all residues prior to analysis. Shown are 12 MD simulations. For clarity time traces of 6 simulations are shown in the plot at the top and 6 at the bottom.

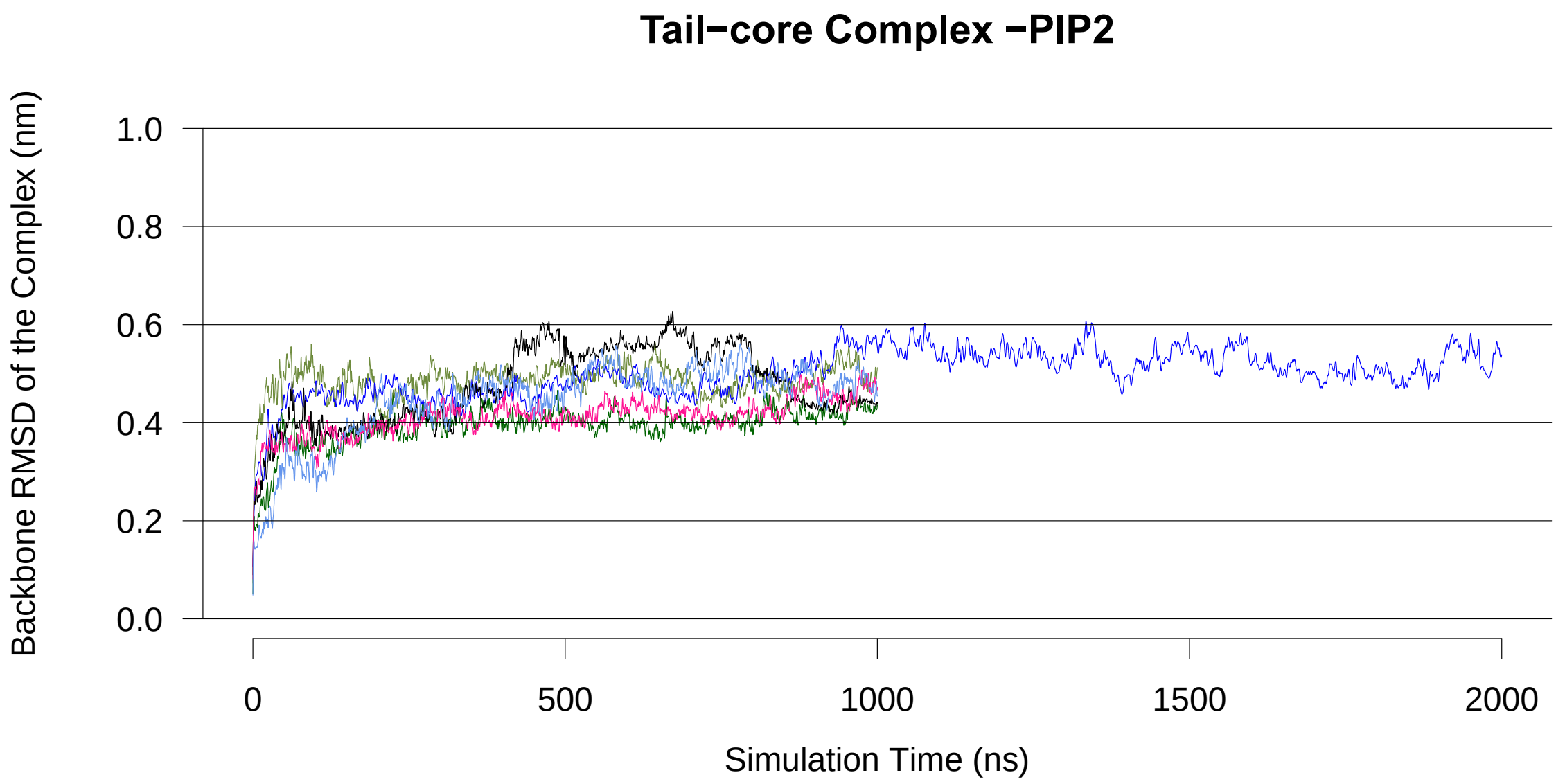

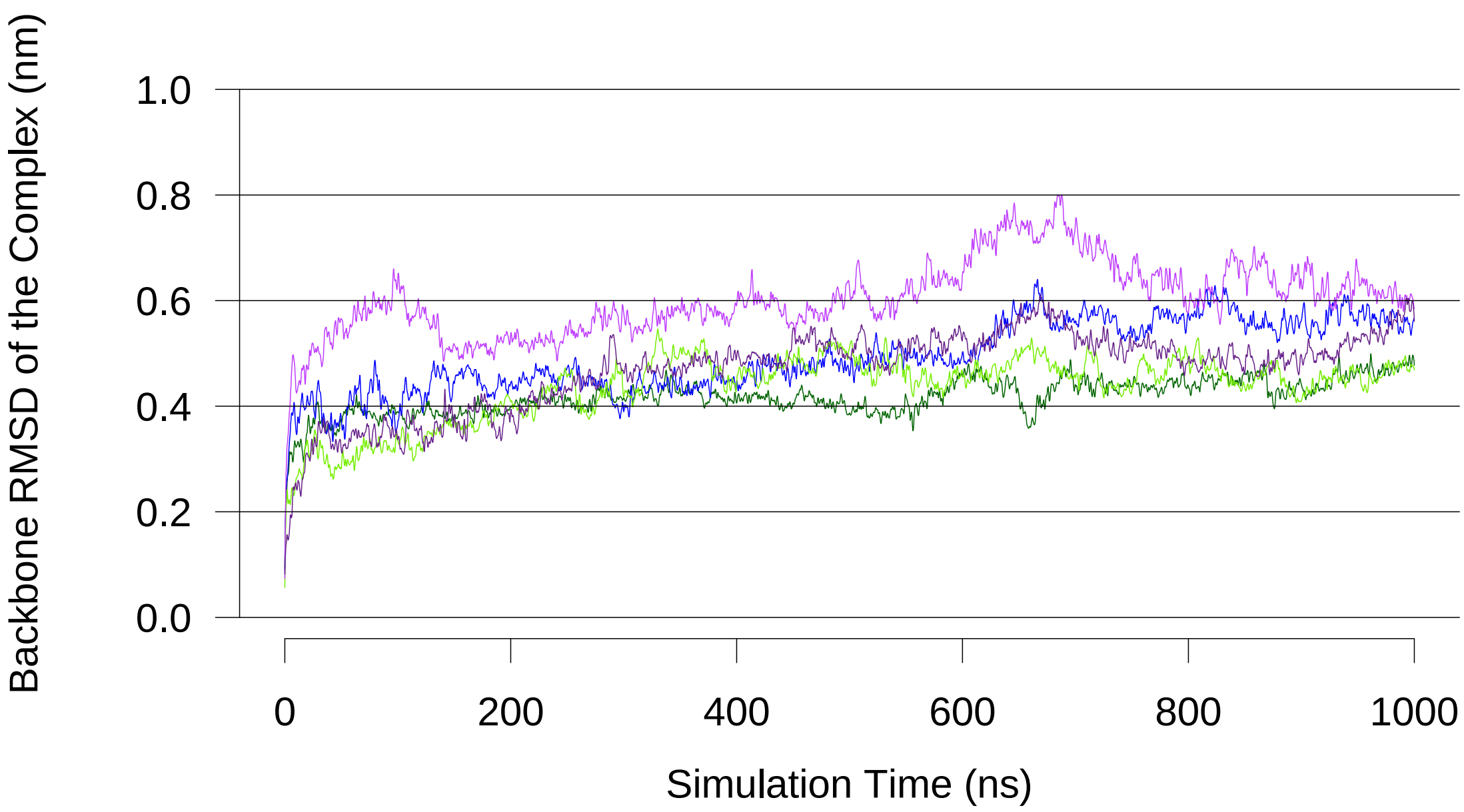

Supplementary Figure 14. Root mean square deviation (RMSD) over time of backbone atoms of β$_2$AR/βarr2 tail-core complexes in a membrane without PIP$_2$. No residues were excluded from the analysis, the complex was fitted on Cα atoms of all residues prior to analysis. Shown are 11 MD simulations. For clarity time traces of 6 simulations are shown in the plot at the top and 5 time traces are shown at the bottom. One random simulation was performed for 2 μs to test for RMSD stability.

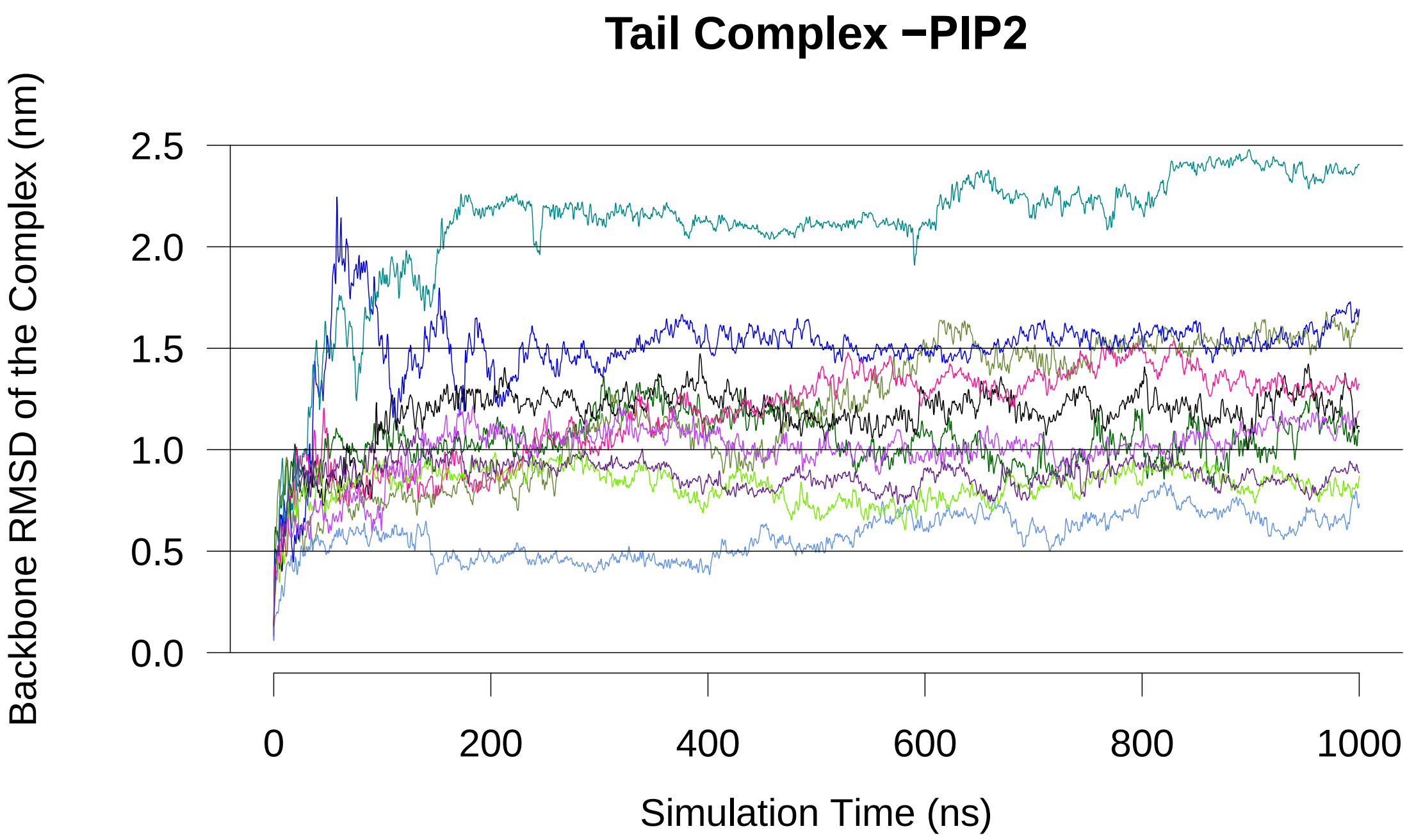

Supplementary Figure 15. Root mean square deviation (RMSD) over time of backbone atoms of β_2AR/βarr2 tail complexes in a membrane without PIP_2. No residues were excluded from the analysis, the complex was fitted on Cα atoms of all residues prior to analysis. Shown are 10 MD simulations.

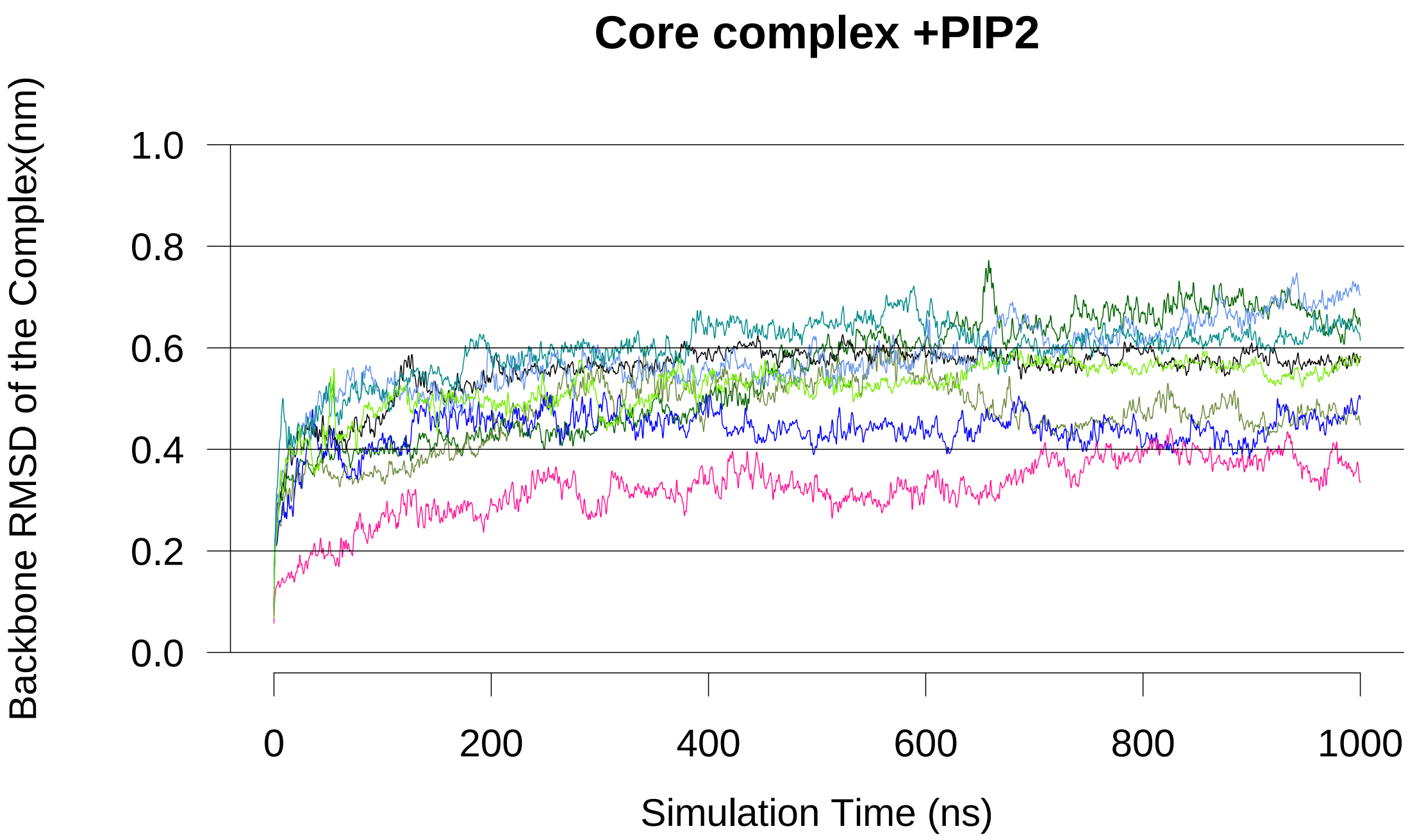

Supplementary Figure 16. Root mean square deviation (RMSD) over time of backbone atoms of β_2AR/βarr2 core complexes in a membrane with PIP_2. No residues were excluded from the analysis, the complex was fitted on Cα atoms of all residues prior to analysis. Shown are 8 MD simulations.

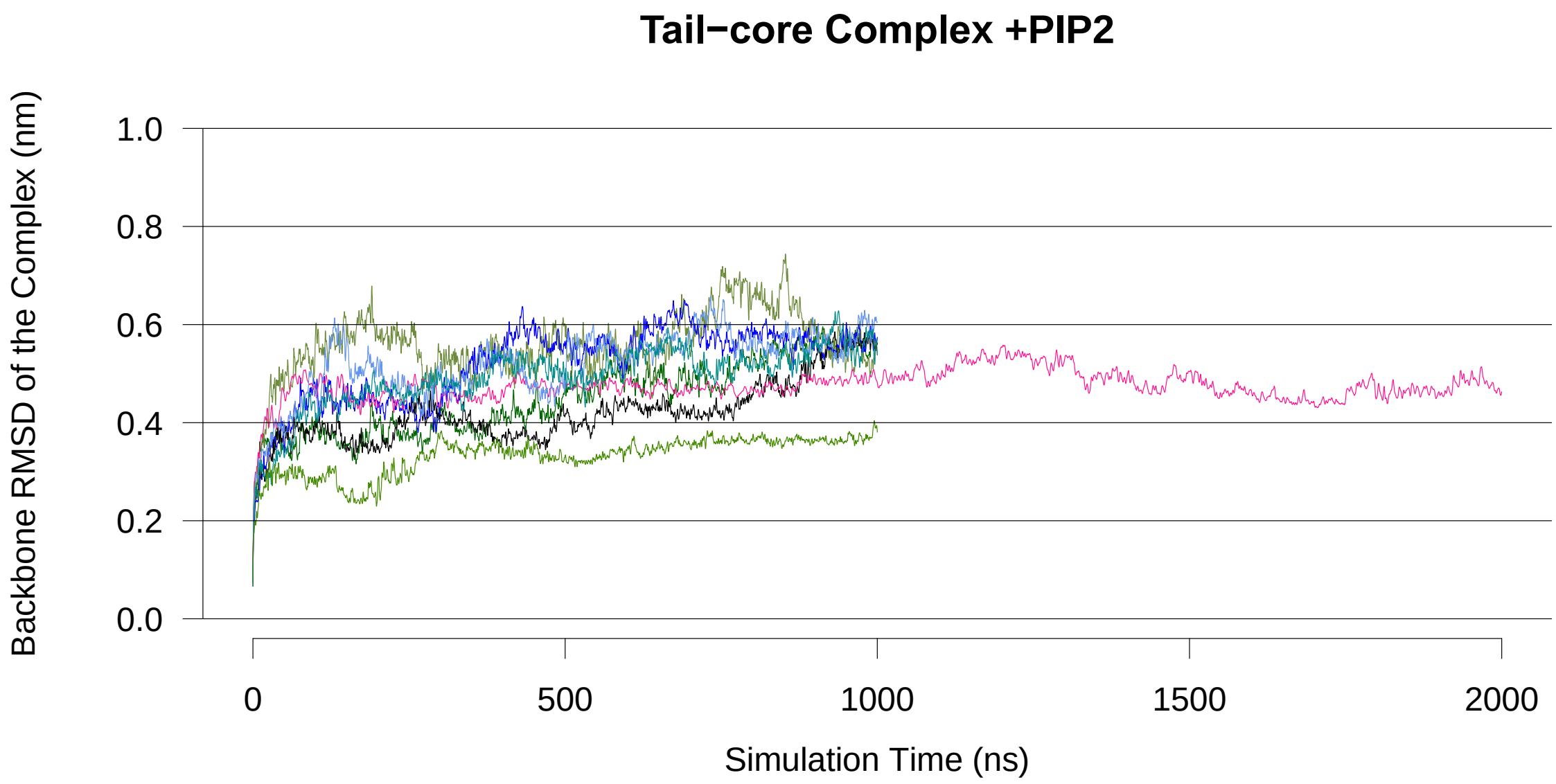

Supplementary Figure 17. Root mean square deviation (RMSD) over time of backbone atoms of β$_2$AR/βarr2 tail-core complexes in a membrane with PIP$_2$. No residues were excluded from the analysis, the complex was fitted on Cα atoms of all residues prior to analysis. Shown are 8 MD simulations. One random simulation was performed for 2 μs to test for RMSD stability.

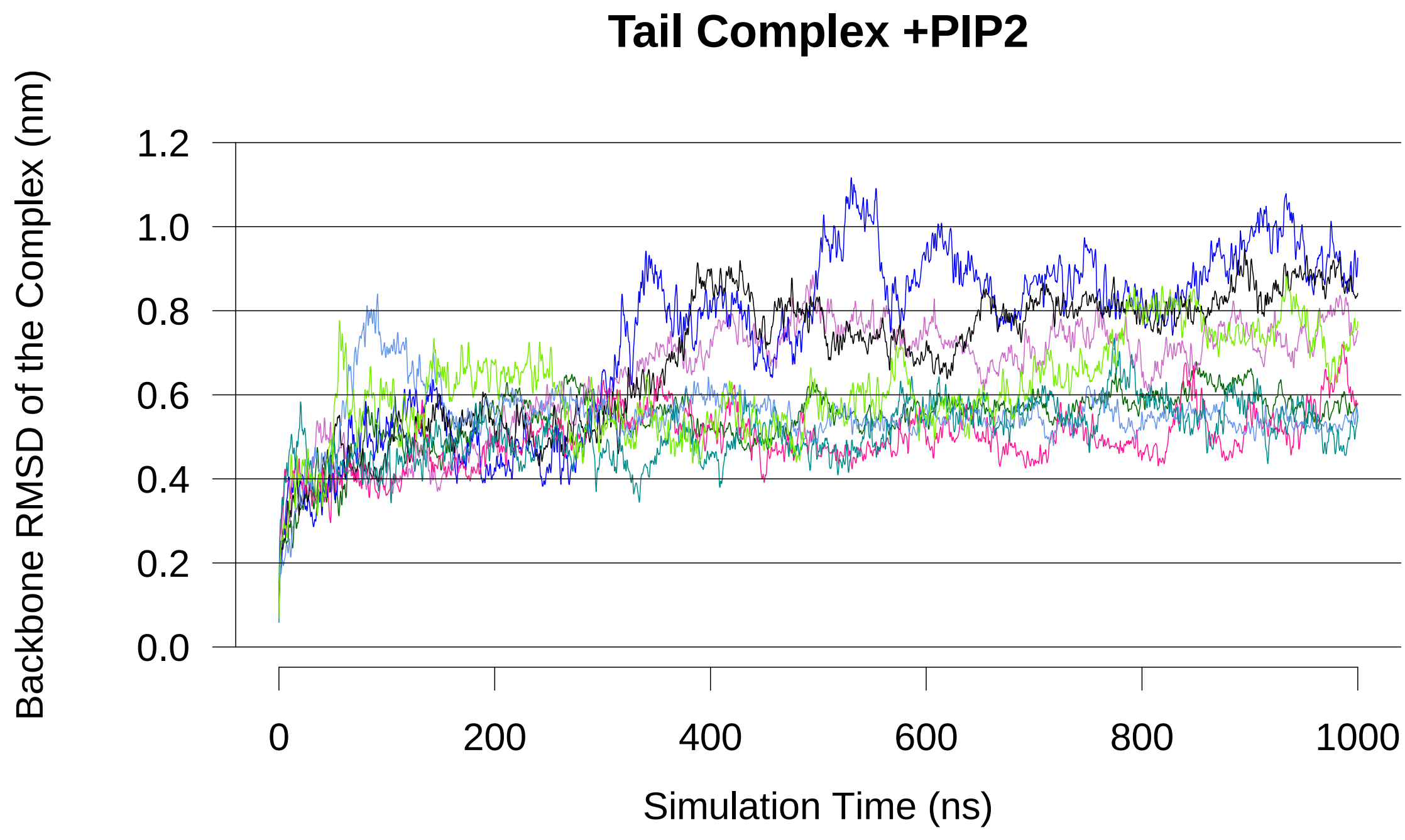

Supplementary Figure 18. Root mean square deviation (RMSD) over time of backbone atoms of β$_2$AR/βarr2 tail complexes in a membrane with PIP$_2$. No residues were excluded from the analysis, the complex was fitted on Cα atoms of all residues prior to analysis. Shown are 8 MD simulations.

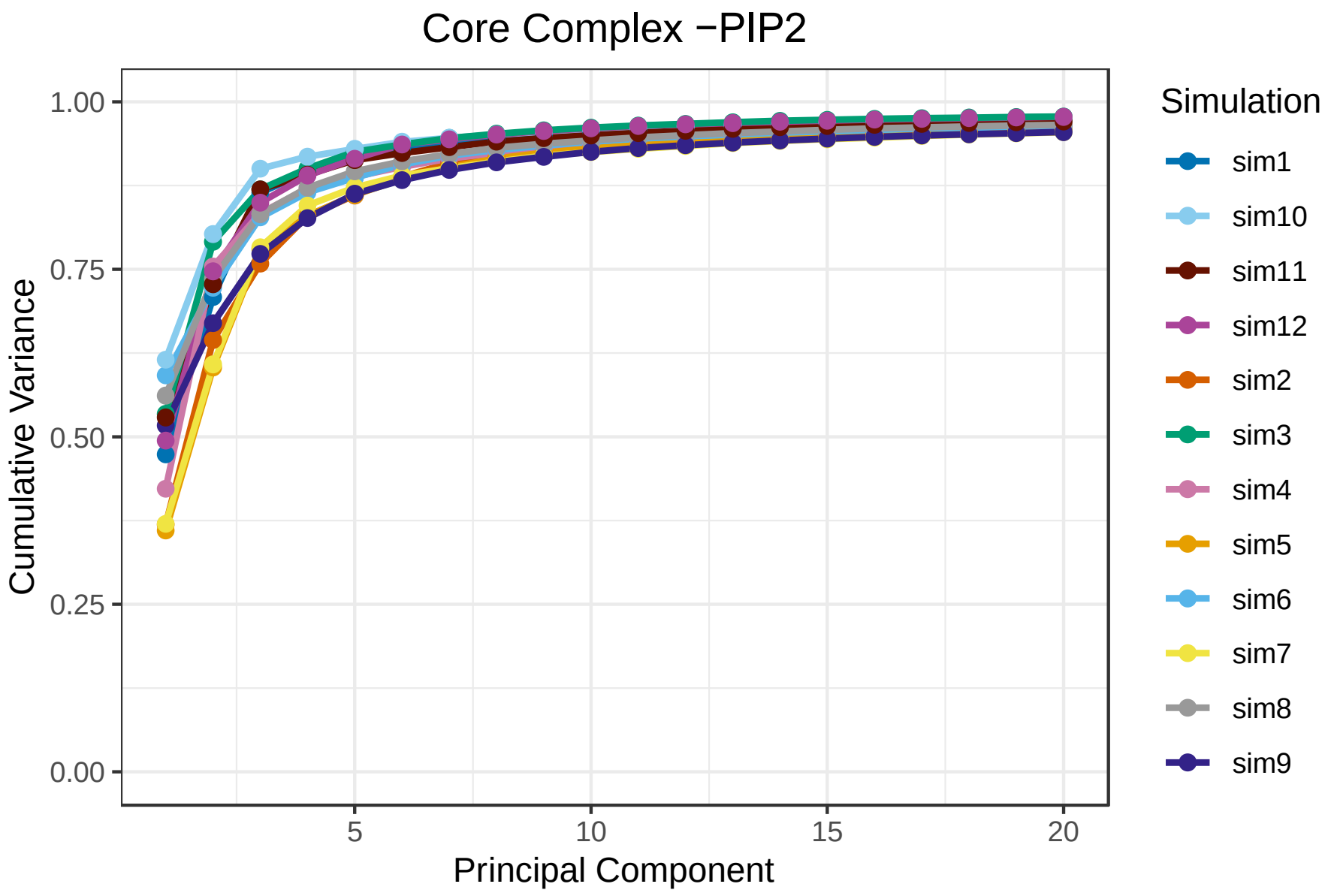

Supplementary Figure 19. Cumulative variance explained by principal component analysis (PCA) for individual MD simulations of the core arrestin–receptor complex in absence of PIP$_2$. Principal components were derived from a reference ensemble constructed by combining trajectory segments from 400 to 1000 ns of all 12 independent MD simulations after alignment on receptor Cα atoms. For each simulation, cumulative variance was calculated from projections of the full 0–1000 ns trajectory onto this common PCA basis. The close overlap and near-parallel behavior of the cumulative variance curves indicate consistent sampling of the dominant collective motions across simulations. In all cases, the first three principal components account for more than 75% of the total variance, suggesting that the essential dynamics of arrestin relative to the receptor are well captured by a low-dimensional subspace.

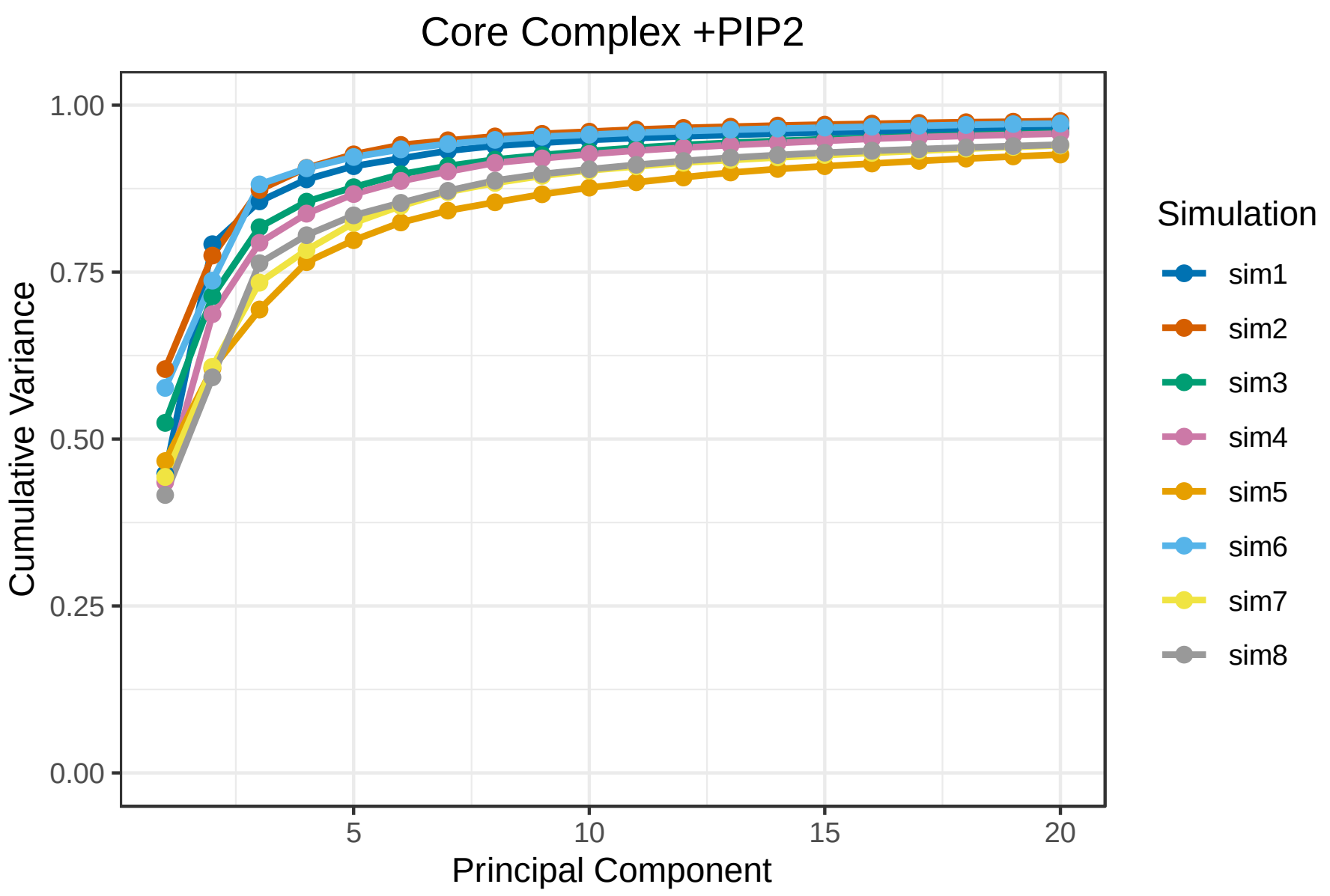

Supplementary Figure 20. Cumulative variance explained by principal component analysis (PCA) for individual MD simulations of the core arrestin–receptor complex in presence of PIP$_2$. Principal components were derived from a reference ensemble constructed by combining trajectory segments from 400 to 1000 ns of all eight independent MD simulations after alignment on receptor Cα atoms. For each simulation, cumulative variance was calculated from projections of the full 0–1000 ns trajectory onto this common PCA basis. The close overlap and near-parallel behavior of the cumulative variance curves indicate consistent sampling of the dominant collective motions across simulations. In all cases, the first four principal components account for more than 75% of the total variance, suggesting

that the essential dynamics of arrestin relative to the receptor are well captured by a low-dimensional subspace.

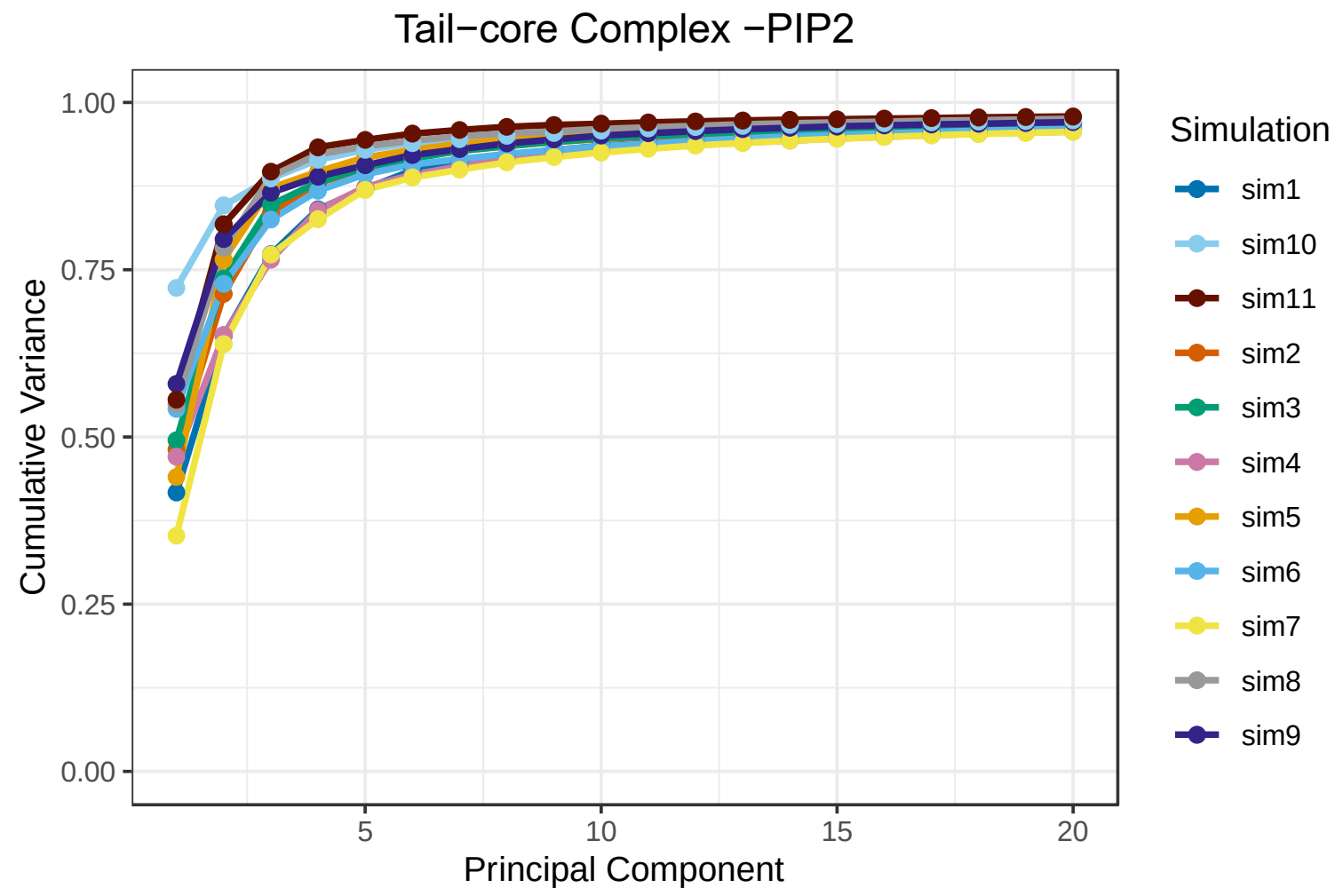

Supplementary Figure 21. Cumulative variance explained by principal component analysis (PCA) for individual MD simulations of the tail-core arrestin–receptor complex in absence of PIP$_2$. Principal components were derived from a reference ensemble constructed by combining trajectory segments from 400 to 1000 ns of all 11 independent MD simulations after alignment on receptor Cα atoms. For each simulation, cumulative variance was calculated from projections of the full 0–1000 ns trajectory onto this common PCA basis. The close overlap and near-parallel behavior of the cumulative variance curves indicate consistent sampling of the dominant collective motions across simulations. In all cases, the first three principal components account for more than 75% of the total variance, suggesting that the essential dynamics of arrestin relative to the receptor are well captured by a low-dimensional subspace.

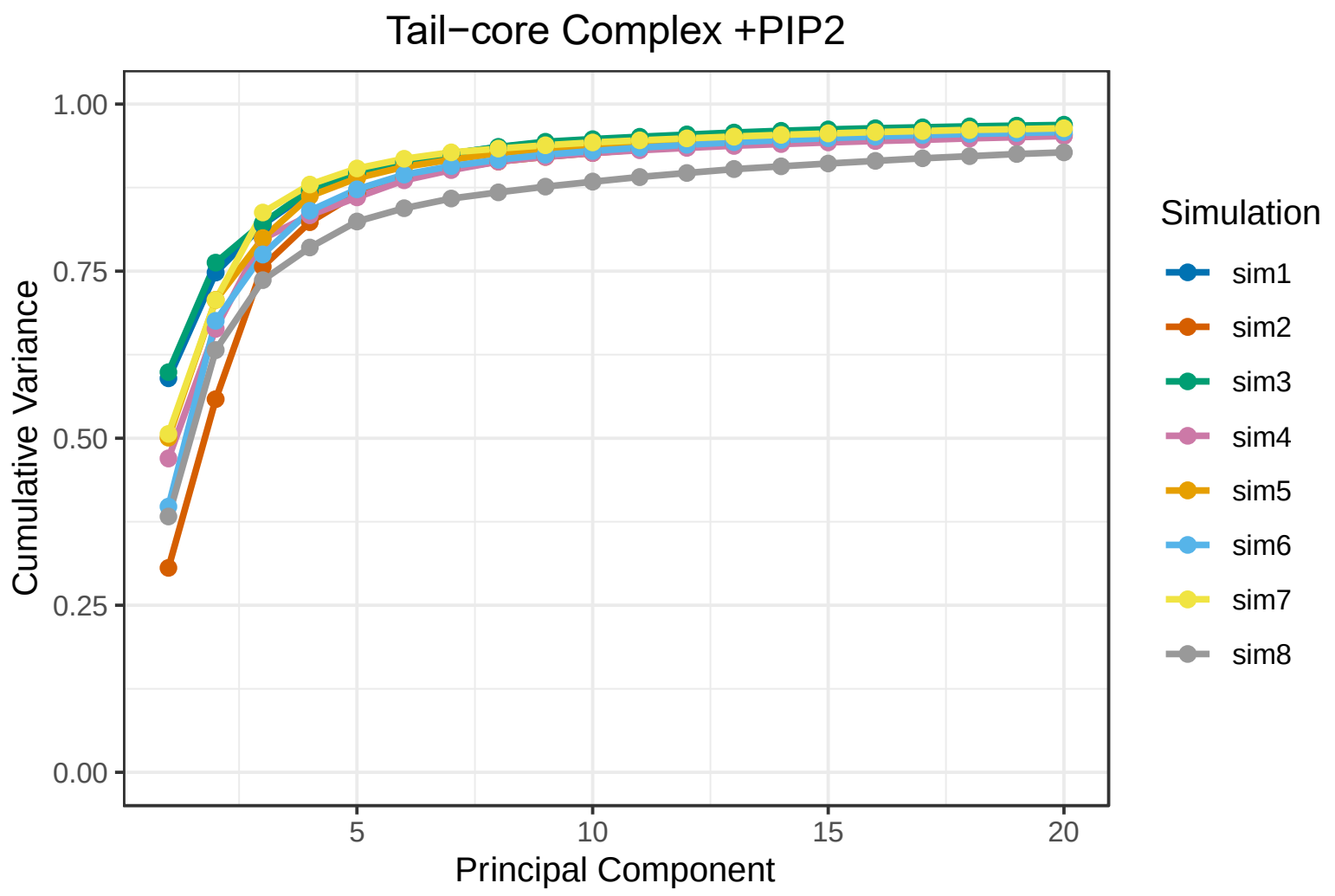

Supplementary Figure 22. Cumulative variance explained by principal component analysis (PCA) for individual MD simulations of the tail-core arrestin–receptor complex in presence of PIP$_2$. Principal components were derived from a reference ensemble constructed by combining trajectory segments from 400 to 1000 ns of all eight independent MD simulations after alignment on receptor Cα atoms. For each simulation, cumulative variance was calculated from projections of the full 0–1000 ns trajectory onto this common PCA basis. The close overlap and near-parallel behavior of the cumulative variance curves indicate consistent sampling of the dominant collective motions across simulations. In all cases, the first three principal components account for more than 75% of the total variance, suggesting that the essential dynamics of arrestin relative to the receptor are well captured by a low-dimensional subspace.

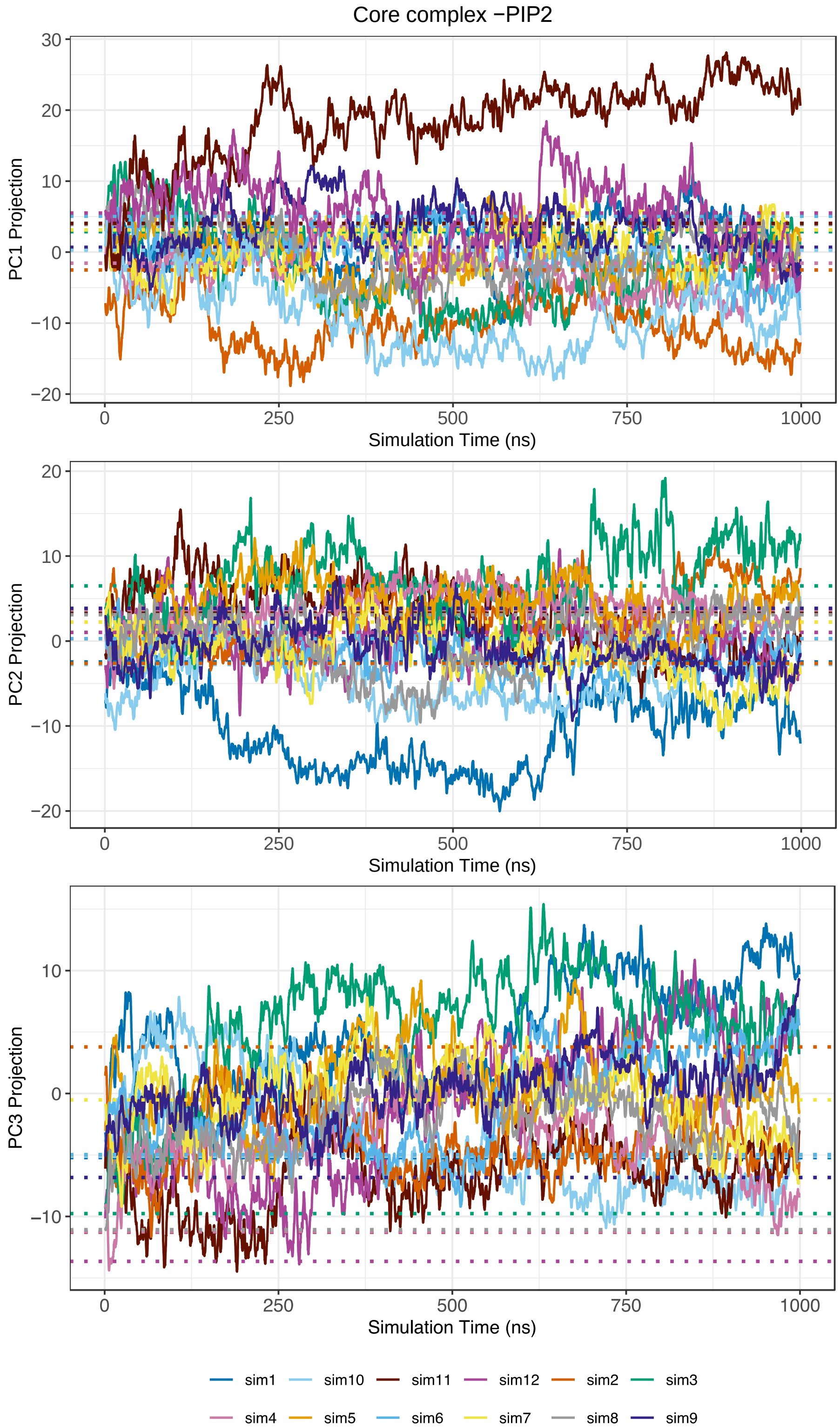

Supplementary Figure 23. Time courses of the first three principal components (PC1–PC3) describing arrestin motion relative to the receptor in individual core complex MD simulations in a membrane lacking PIP$_2$. To derive global principal components representing the dominant arrestin motions relative to the receptor, trajectory segments from 400 to 1000 ns of all independent simulations were combined after alignment on receptor Cα atoms, excluding the flexible intracellular loop 3 (ICL3) and C-terminal tail. Individual simulations were subsequently projected onto these ensemble-specific components. Visual inspection of the corresponding eigenvectors indicates that PC1–PC3 capture distinct combinations of arrestin tilting and rotational motions around the finger loop, as well as relative rearrangements between the N- and C-lobes. The dashed lines indicate the individual values at the beginning of the simulations. Shown are 10 MD simulations.

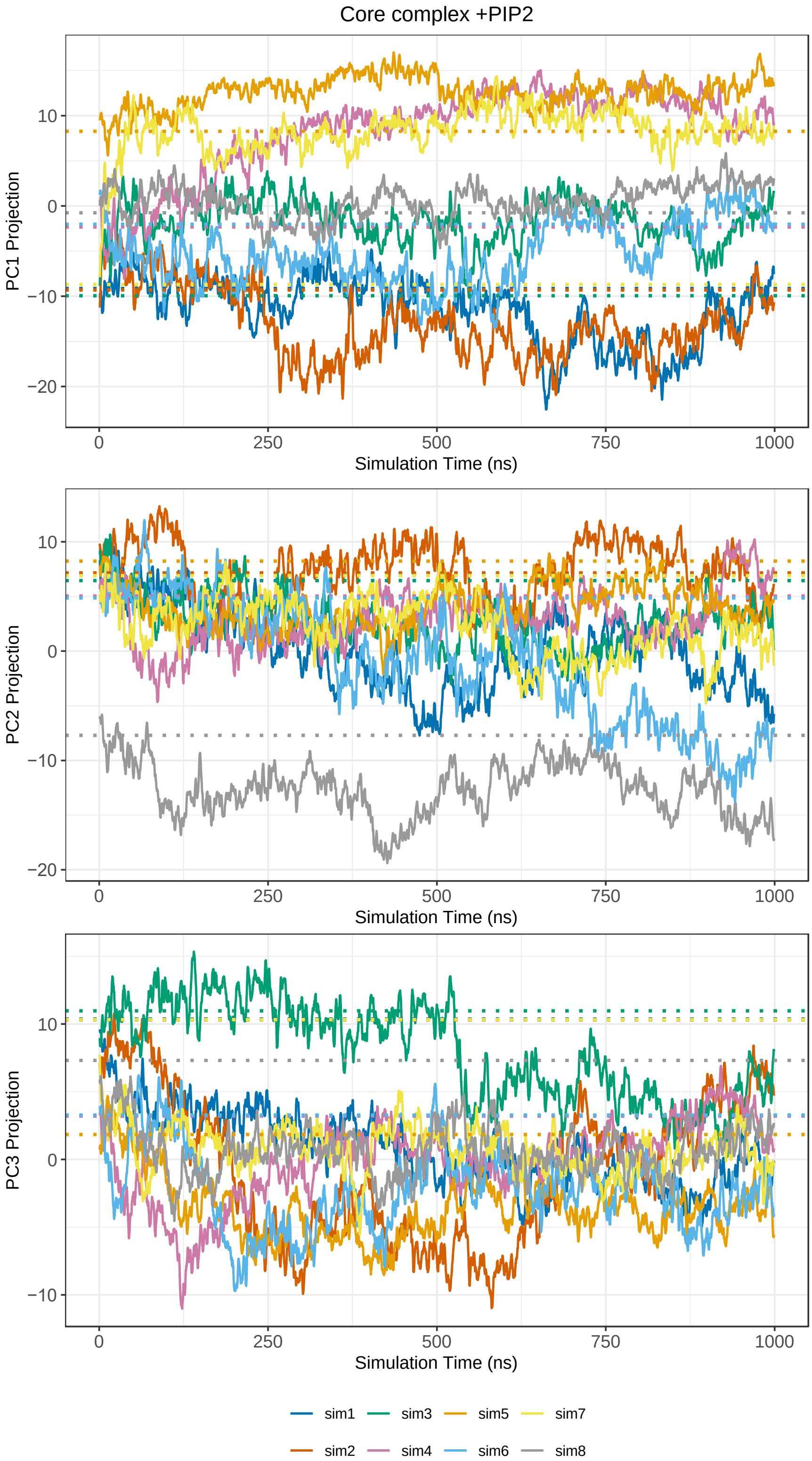

Supplementary Figure 24. Time courses of the first three principal components (PC1–PC3) describing arrestin motion relative to the receptor in individual core complex MD simulations in the membrane with PIP$_2$. To derive global principal components representing the dominant arrestin motions relative to the receptor, trajectory segments from 400 to 1000 ns of all independent simulations were combined after alignment on receptor Cα atoms, excluding the flexible intracellular loop 3 (ICL3) and C-terminal tail. Individual simulations were subsequently projected onto these ensemble-specific components. Visual inspection of the corresponding eigenvectors indicates that PC1–PC3 capture distinct combinations of arrestin tilting and rotational motions around the finger loop, as well as relative

rearrangements between the N- and C-lobes. The dashed lines indicate the individual values at the beginning of the simulations. Shown are 8 MD simulations.

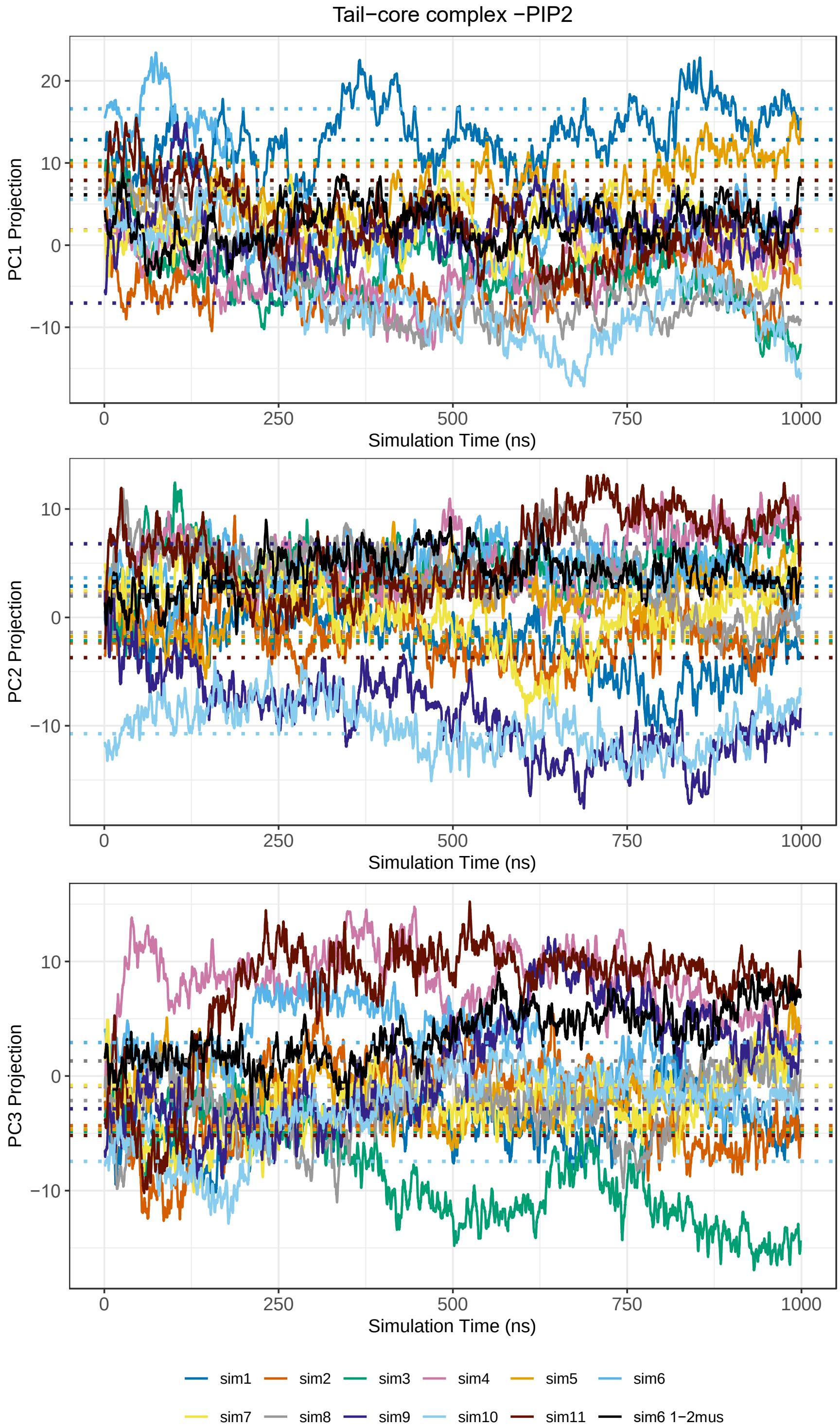

Supplementary Figure 25. Time courses of the first three principal components (PC1–PC3) describing arrestin motion relative to the receptor in individual tail–core complex MD simulations in a membrane without PIP_2. To derive global principal components representing the dominant arrestin motions relative to the receptor, trajectory segments from 400 to 1000 ns of all independent simulations were combined after alignment on receptor Cα atoms, excluding the flexible intracellular loop 3 (ICL3) and C-terminal tail. Individual simulations were subsequently projected onto these ensemble-specific components. Visual inspection of the corresponding eigenvectors indicates that PC1–PC3 capture distinct combinations of arrestin tilting and rotational motions around the finger loop, as well as relative rearrangements between the N- and C-lobes. In black, the time course of 1–2 μs of simulation 6 is given. The dashed lines indicate the individual values at the beginning of the simulations.

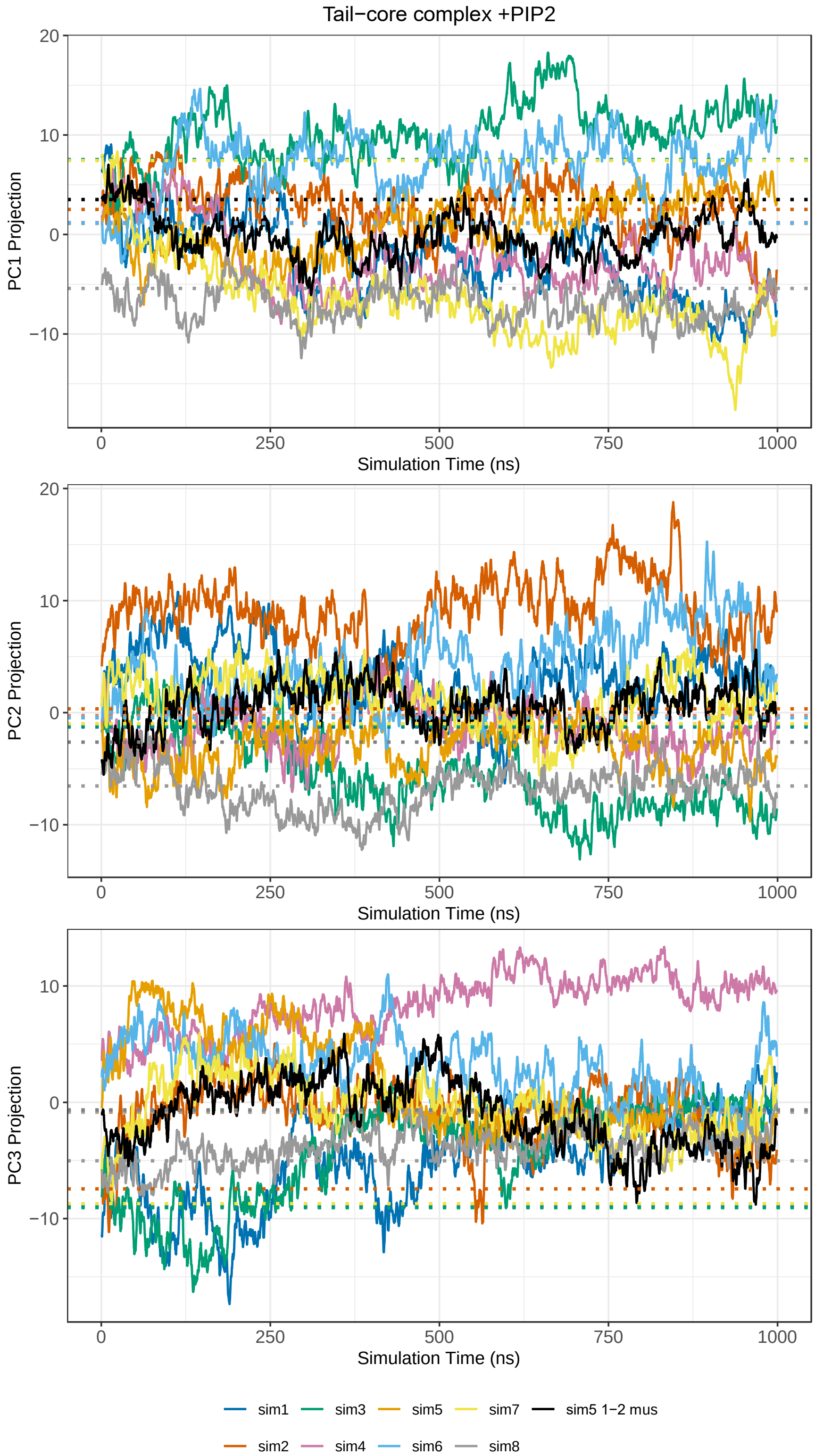

Supplementary Figure 26. Time courses of the first three principal components (PC1–PC3) describing arrestin motion relative to the receptor in individual tail–core complex simulations in a membrane with PIP$_2$. To derive global principal components representing the dominant arrestin motions relative to the receptor, trajectory segments from 400 to 1000 ns of all eight independent MD simulations were combined after alignment on receptor Cα atoms, excluding the flexible intracellular loop 3 (ICL3) and C-terminal tail. Individual simulations were subsequently projected onto these ensemble-specific components. Visual inspection of the corresponding eigenvectors indicates that PC1–PC3 capture distinct combinations of arrestin tilting and rotational motions around the finger loop, as well as relative rearrangements between the N- and C-lobes. In black, the time course of 1–2 µs of simulation 5 is given. The dashed lines indicate the individual values at the beginning of the simulations.

Time-dependent analysis of all-atom MD simulations of βarr2/membrane interactions

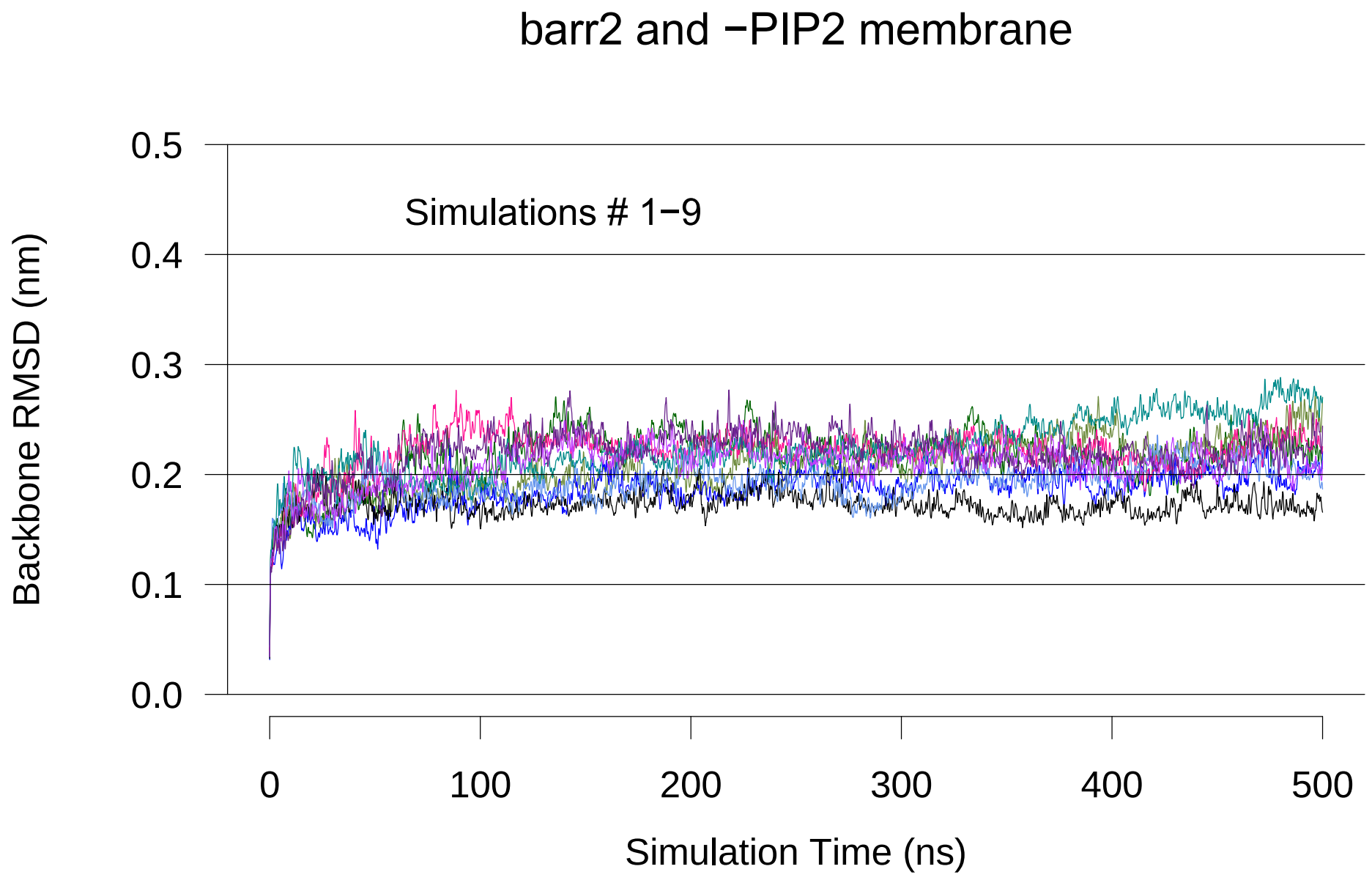

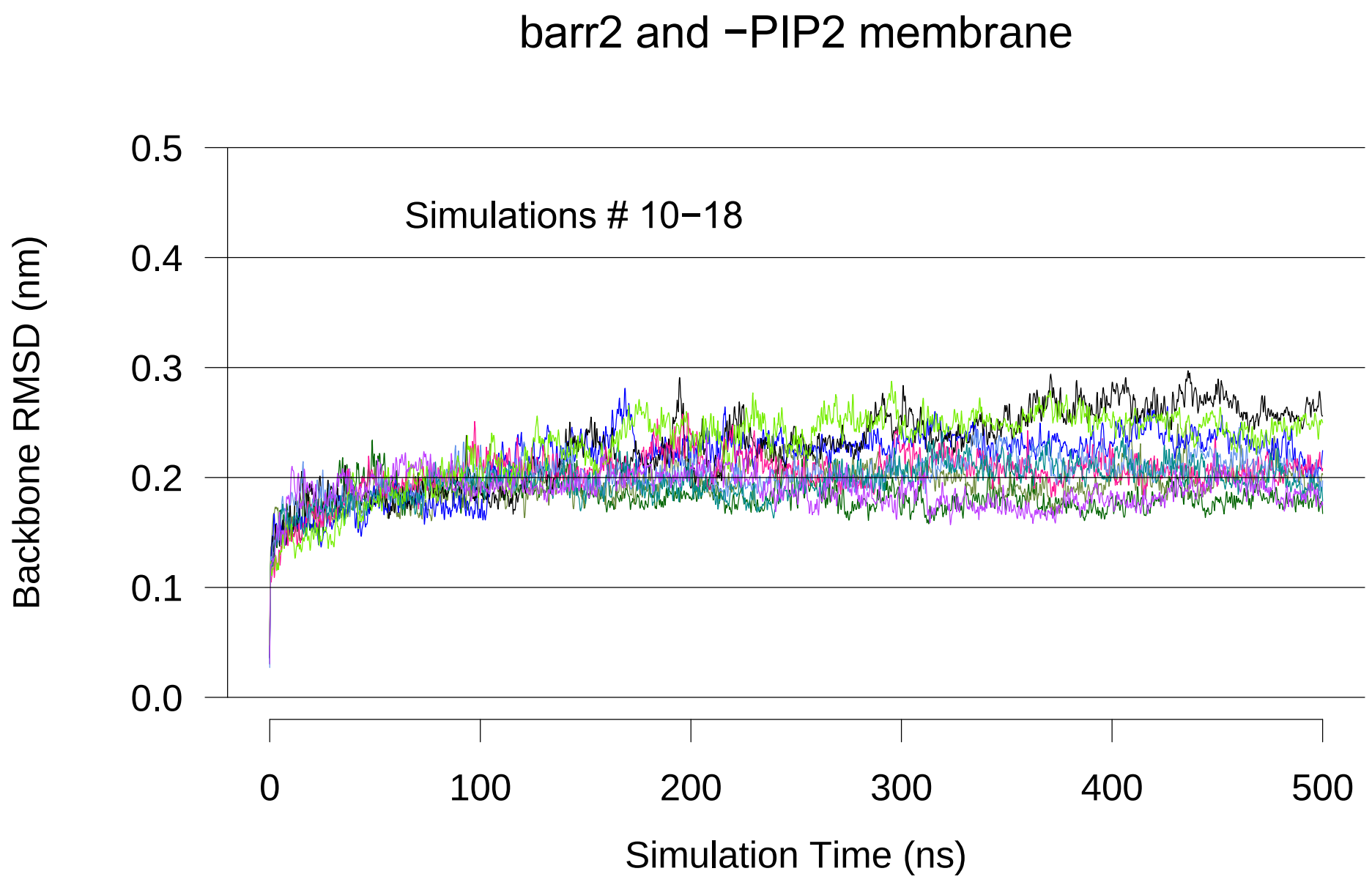

Supplementary Figure 27. Root mean square deviations (RMSD) over time of backbone atoms of inactive βarr2 interacting with a membrane without PIP_2. The flexible termini were excluded from the analysis, i.e. the analysis was performed on arrestin residues 8–347 only. Arrestin was fitted on Cα atoms of the same residues prior to analysis. RMSD of simulations 1–9 is shown at the top and of simulations 10–18 at the bottom. Shown are 18 MD simulations.

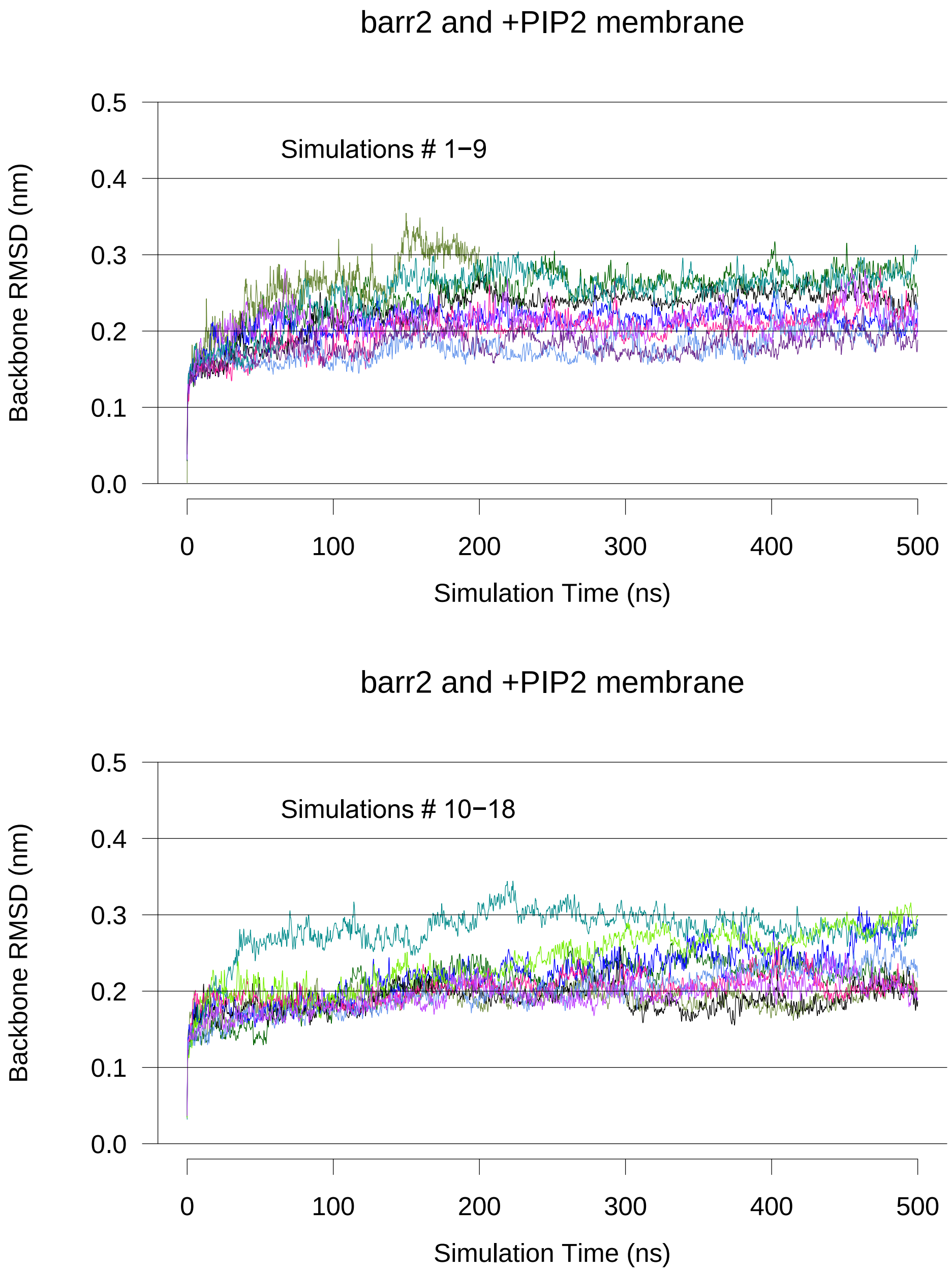

Supplementary Figure 28. Root mean square deviations (RMSD) over time of backbone atoms of inactive βarr2 interacting with a membrane with PIP_2. The flexible termini were excluded from the analysis, i.e. the analysis was performed on arrestin residues 8–347 only. Arrestin was fitted on Cα atoms of the same residues prior to analysis. RMSD of simulations 1–9 is shown at the top and of simulations 10–18 at the bottom. Shown are 18 MD simulations.

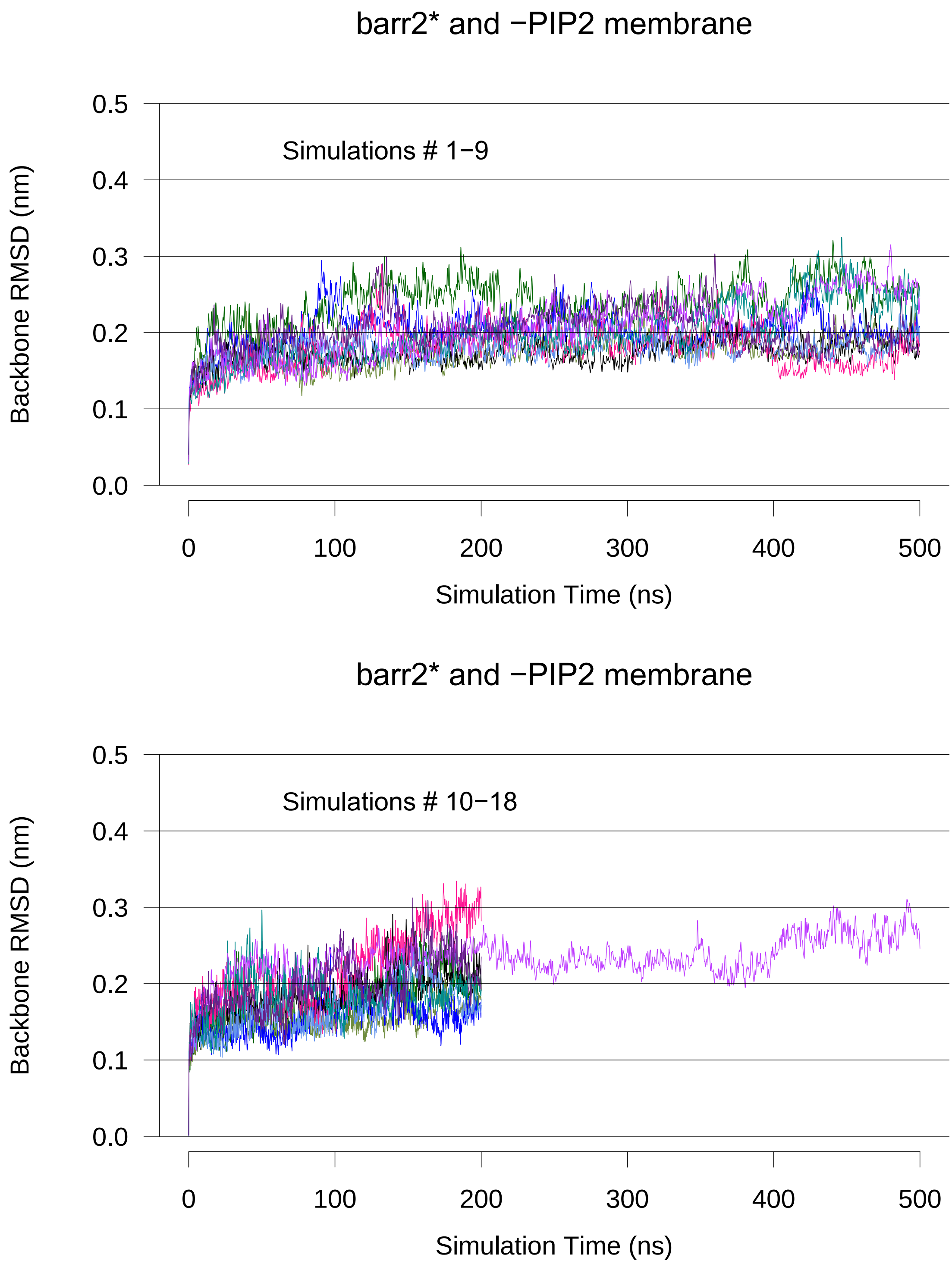

Supplementary Figure 29. Root mean square deviations (RMSD) over time of backbone atoms of active βarr2* interacting with a membrane without PIP_2. The flexible termini were excluded from the analysis, i.e. the analysis was performed on arrestin residues 8–347 only. Arrestin was fitted on Cα atoms of the same residues prior to analysis. RMSD of MD simulations 1–9 is shown at the top and of MD simulations 10–18 at the bottom. In simulations 1–10 the active state of βarr2* was stabilized by a phosphorylated C-tail peptide (comprising β_2AR residues 342–362 and being phosphorylated on residues 355 and 356). Simulations 11–18 are conducted for 200 ns only as they originate from tail-core complexes by deleting adrenaline-bound receptor. Shown are 18 MD simulations.

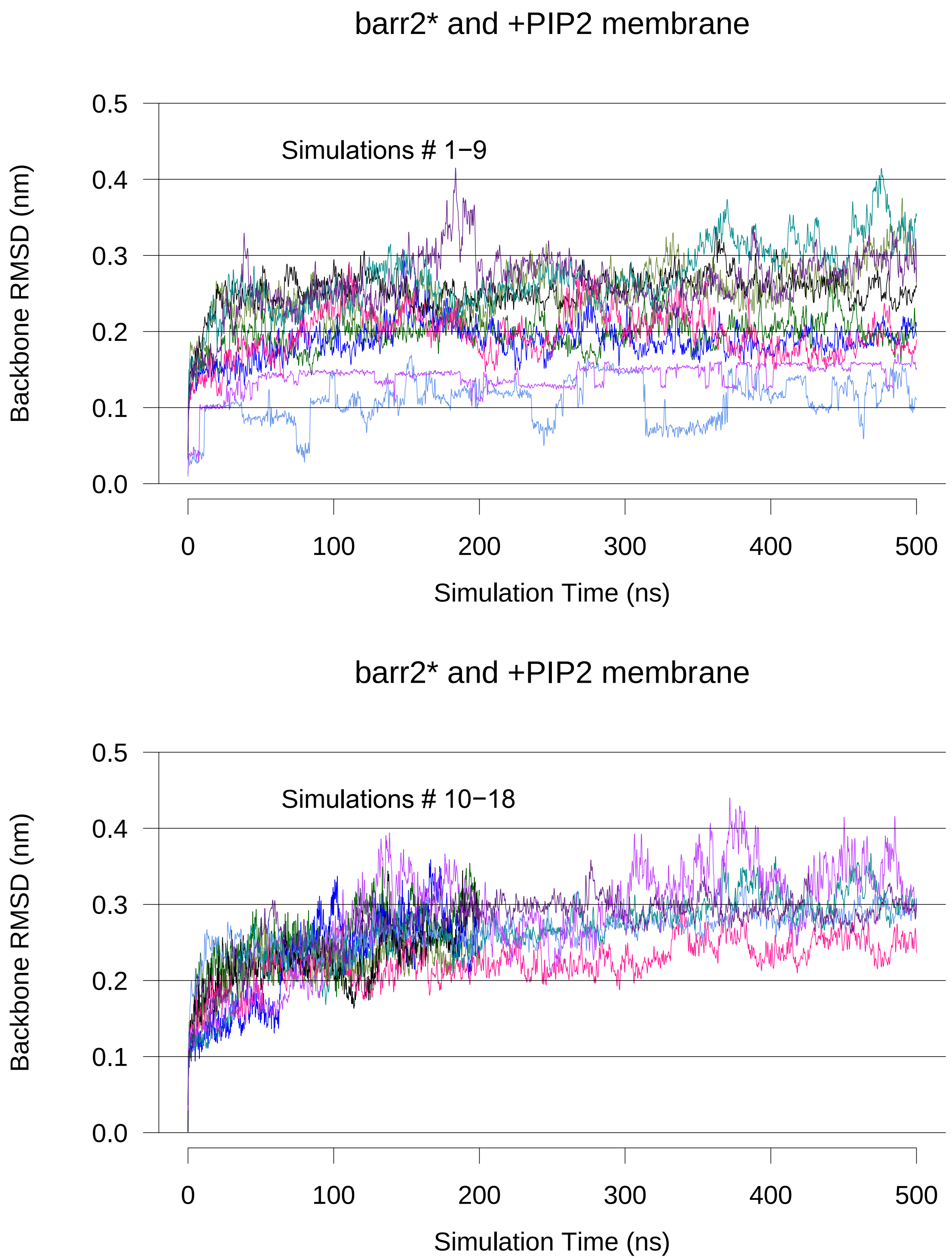

Supplementary Figure 30. Root mean square deviations (RMSD) over time of backbone atoms of active βarr2* interacting with a membrane with PIP_2. The flexible termini were excluded from the analysis, i.e. the analysis was performed on arrestin residues 8–347 only. Arrestin was fitted on Cα atoms of the same residues prior to analysis. RMSD of MD simulations 1–9 is shown at the top and of MD simulations 10–18 at the bottom. In simulations 1–14 the active state of βarr2* was stabilized by a phosphorylated C-tail peptide (comprising β_2AR residues 342–362 and being phosphorylated on residues 355 and 356). Simulations # 15–18 are conducted for 200 ns only as they originate from tail-core complexes by deleting adrenaline-bound receptor. Shown are 18 MD simulations.

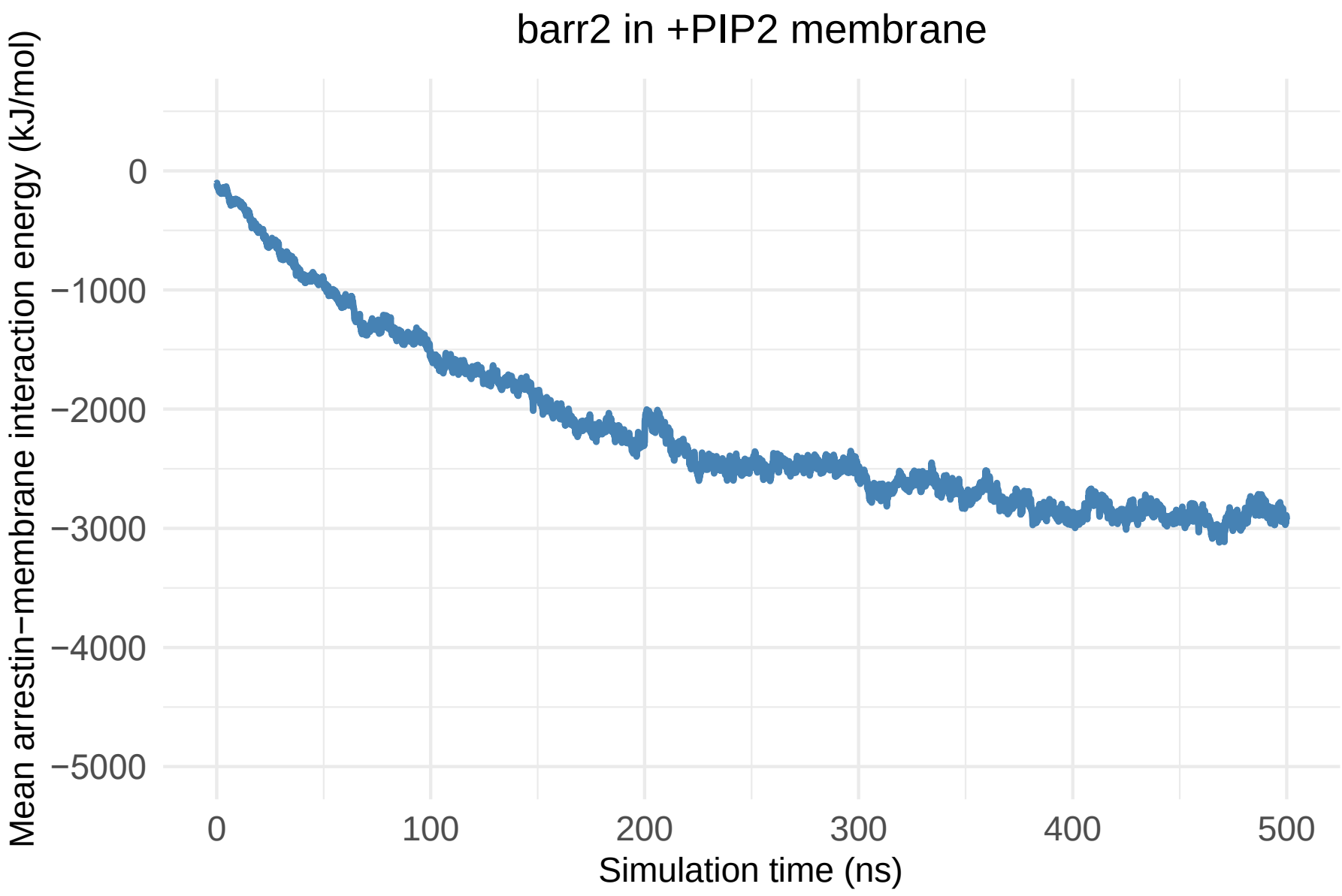

Supplementary Figure 31. Mean interaction energy of inactive βarr2 with PIP_2-containing membrane upon spontaneous binding. The shaded area denotes the SD from 18 MD simulations.

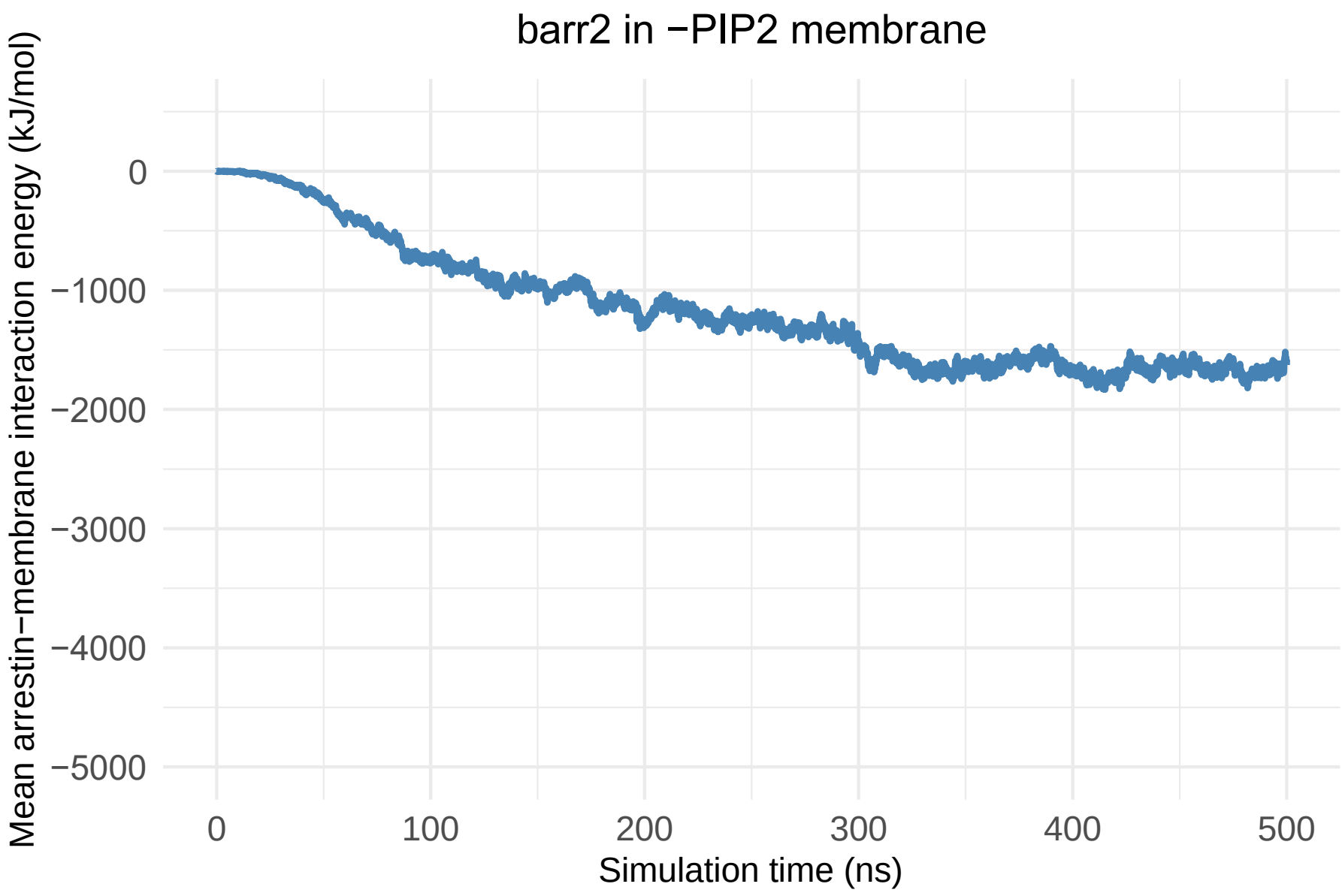

Supplementary Figure 32. Mean interaction energy of inactive βarr2 with PIP_2-lacking membrane upon spontaneous binding. The shaded area denotes the SD from 18 MD simulations.

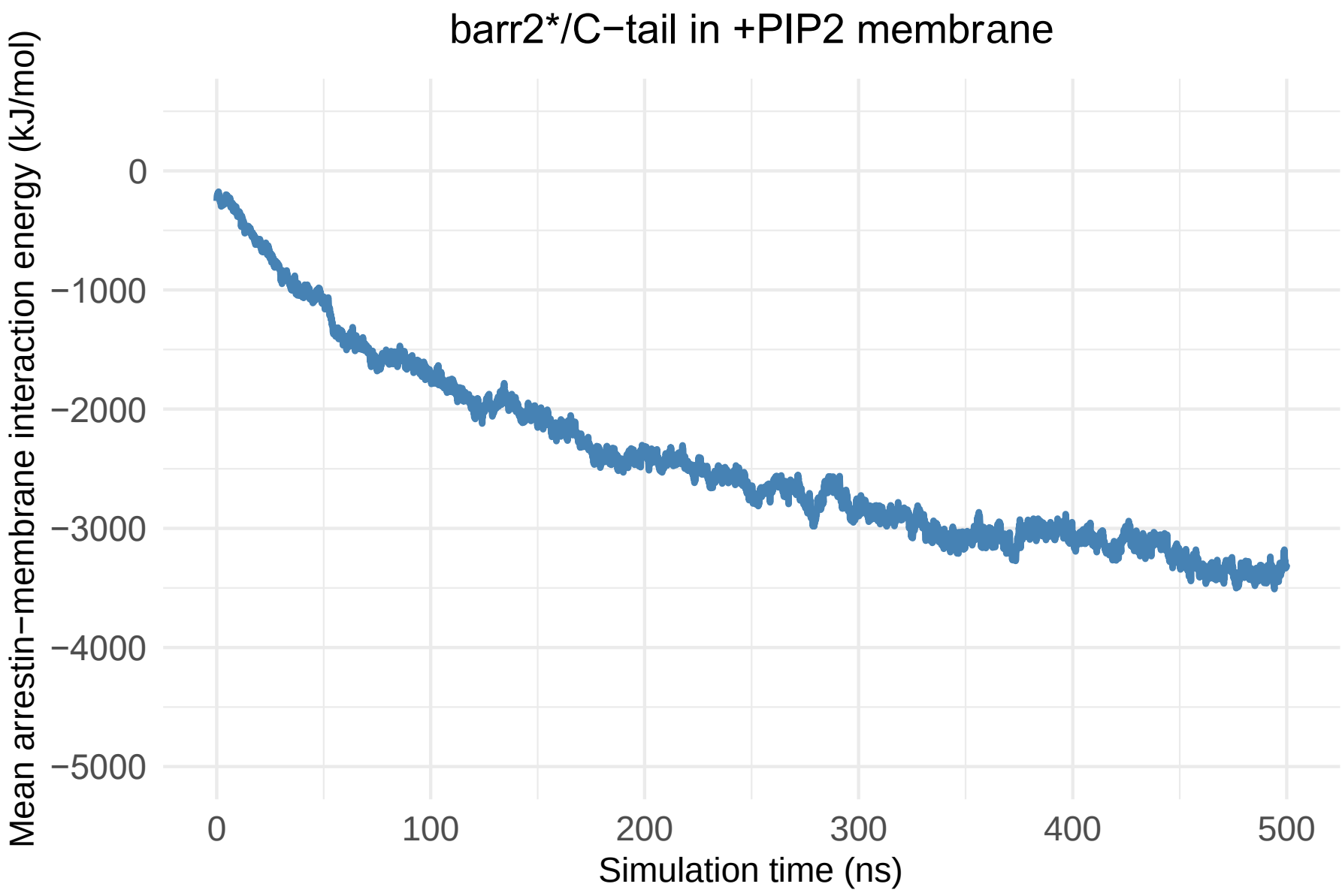

Supplementary Figure 33. Mean interaction energy of βarr2* with PIP_2-containing membrane upon spontaneous binding. βarr2* is stabilized in the active state by a phosphorylated C-tail peptide (comprising β_2AR residues 342–362 and being phosphorylated on residues 355 and 356). The shaded area denotes the SD from 14 MD simulations.

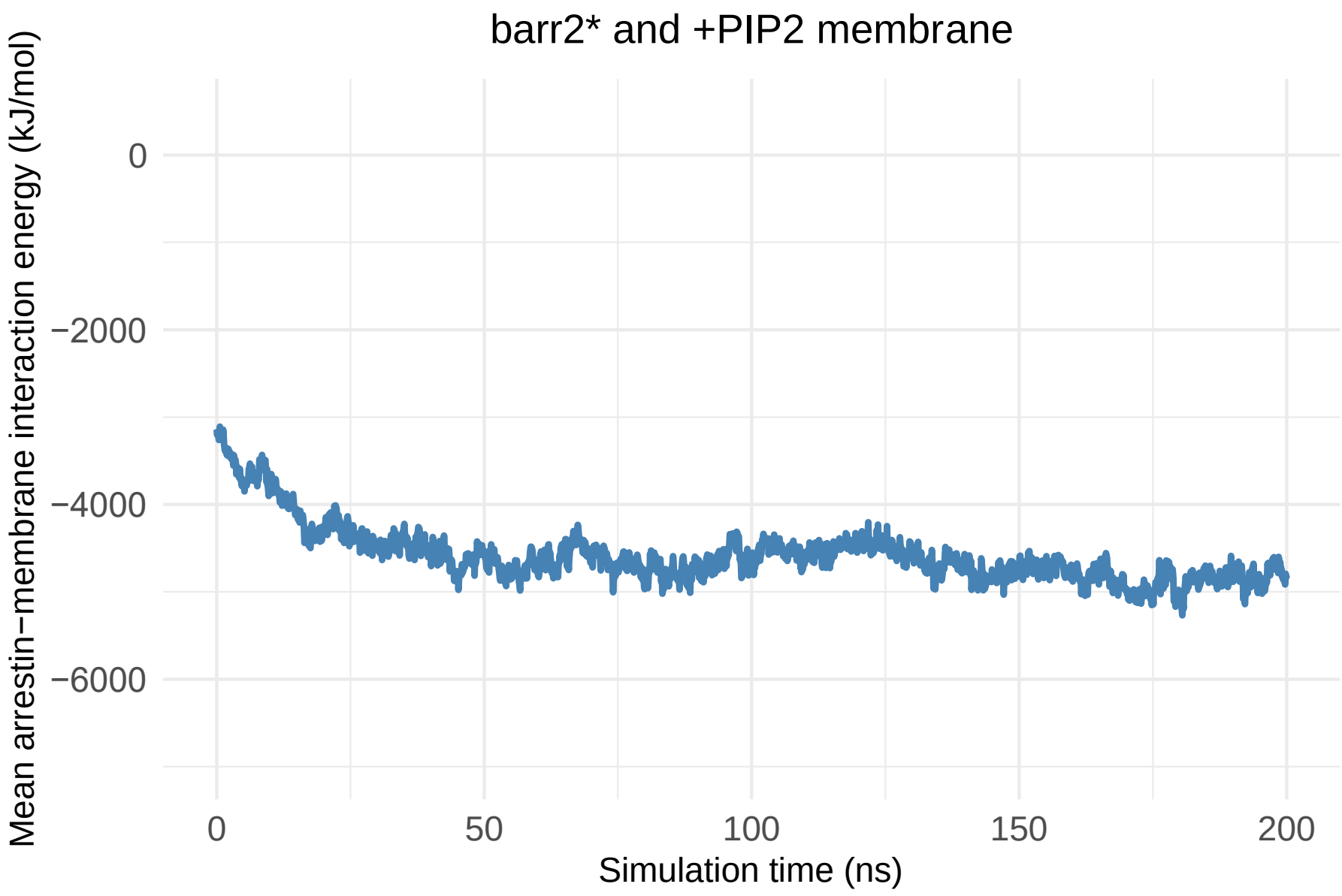

Supplementary Figure 34. Mean interaction energy of βarr2* with PIP_2-containing membrane. Simulations were initiated from the tail-core complexes in PIP_2-containing membrane by deletion of the adrenaline-bound receptor. The shaded area denotes the SD from 4 MD simulations.

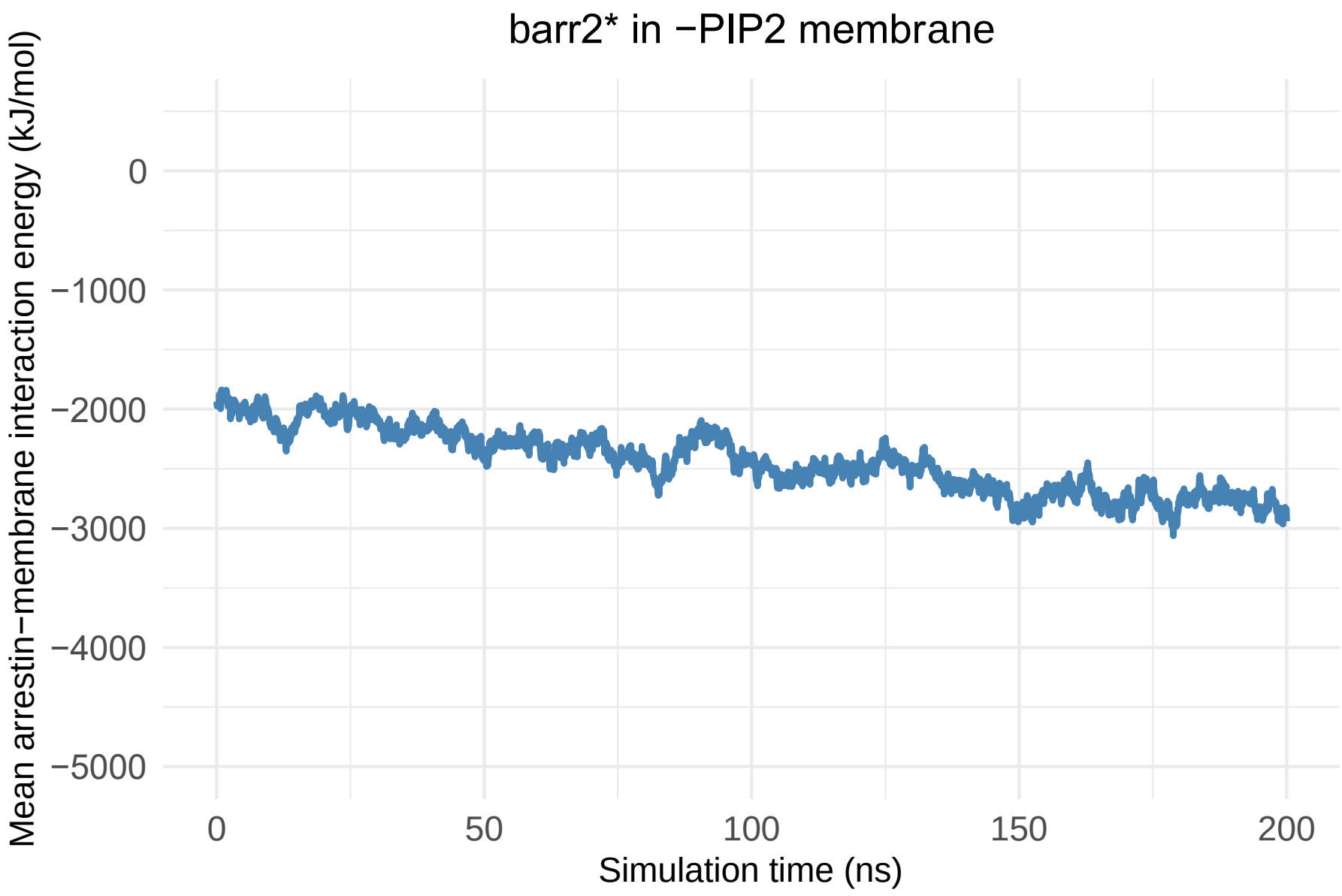

Supplementary Figure 35. Mean interaction energy of βarr2* with PIP_2-lacking membrane. Simulations were initiated from the tail-core complexes by deletion of the adrenaline-bound receptor. The shaded area denotes the SD from 8 MD simulations.

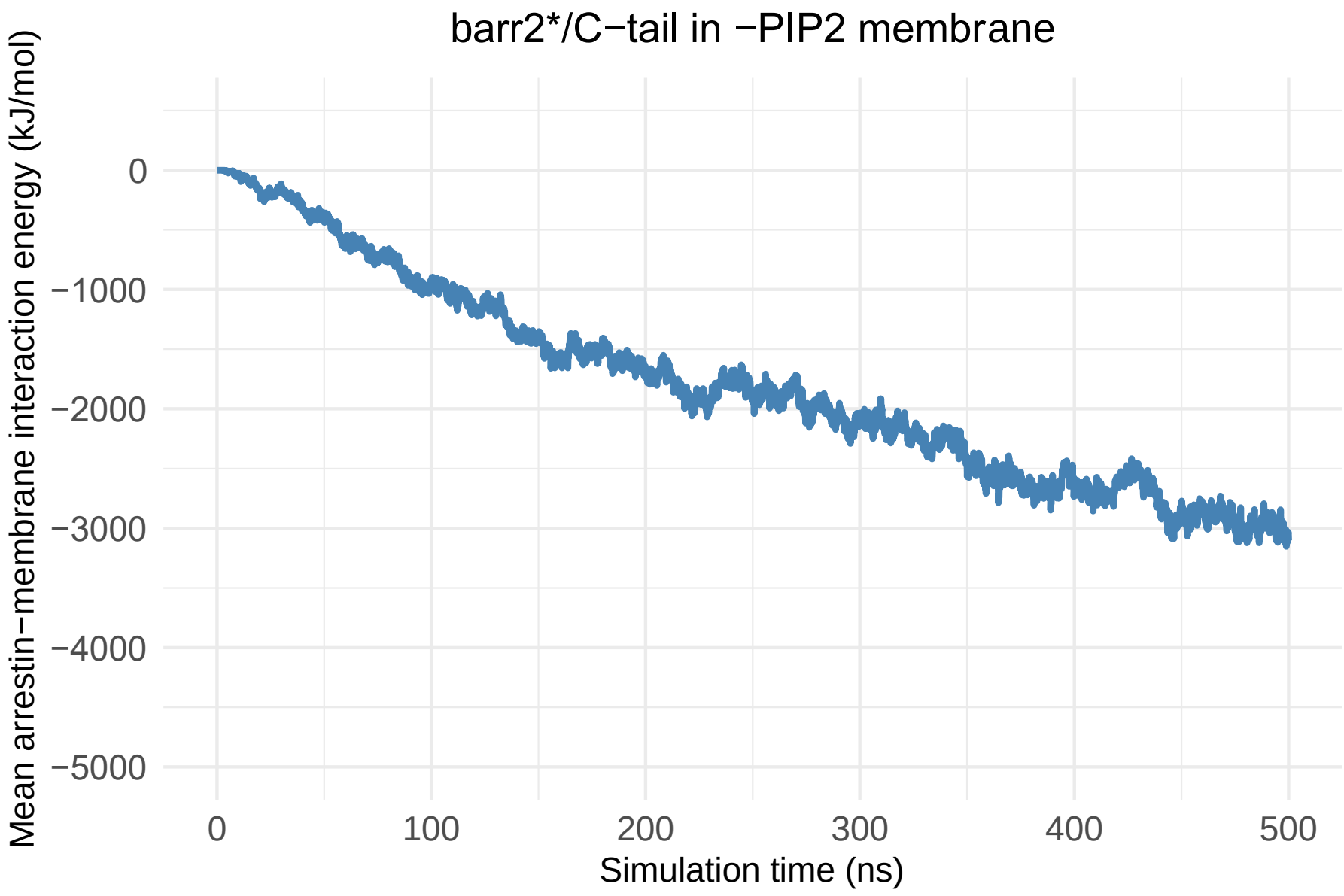

Supplementary Figure 36. Mean interaction energy of βarr2* with PIP_2-lacking membrane upon spontaneous binding. βarr2* is stabilized in the active state by a phosphorylated C-tail peptide (comprising β_2AR residues 342–362 and being phosphorylated on residues 355 and 356). The shaded area denotes the SD from 10 MD simulations.

Time-dependent analysis of all-atom MD simulations of membrane-embedded β$_2$AR (phosphorylated or nonphosphorylated) including prolonged C-tail, i.e. comprising residues 29–378, and being activated by adrenaline

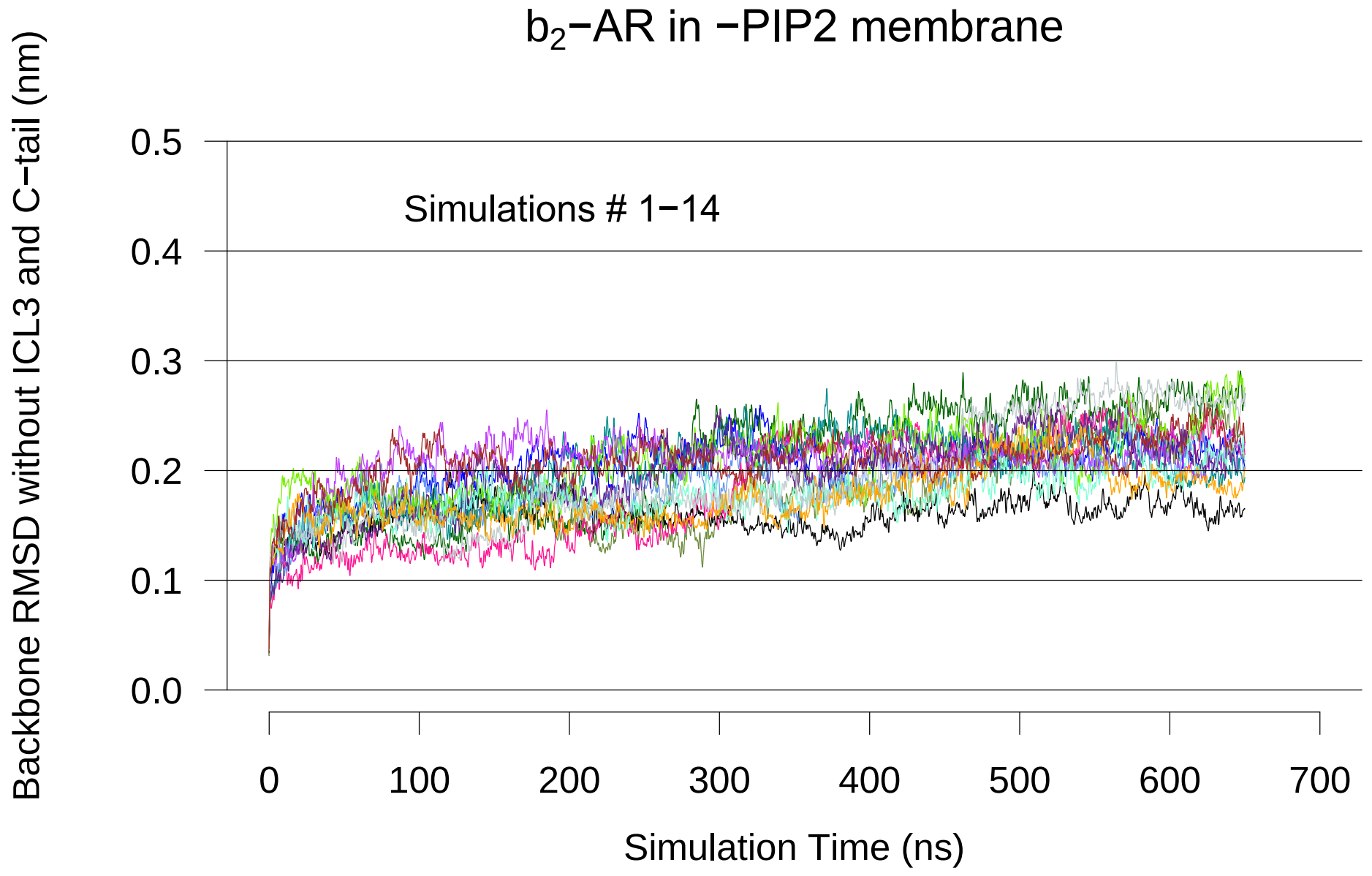

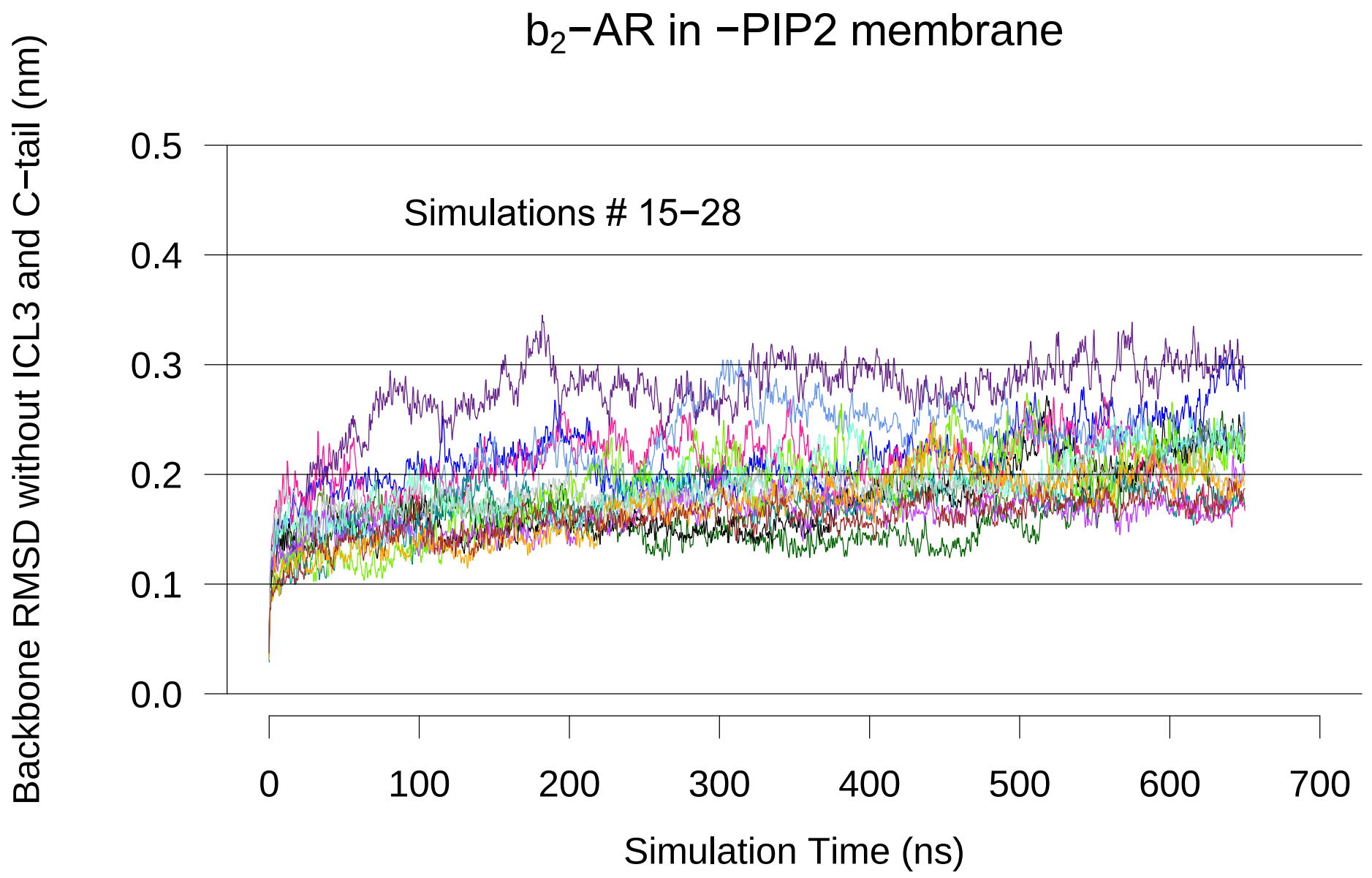

Supplementary Figure 41. Root mean square deviation (RMSD) over time of backbone atoms of nonphosphorylated β$_2$AR embedded in a membrane without PIP$_2$. The flexible ICL3 (residues 232–262) and C-tail (residues 342–378) were excluded from the analysis. The receptor was fitted on Cα atoms (without ICL3 and C-tail residues) prior to analysis. RMSD of MD simulations 1–14 is shown at the top and of MD simulations 15–28 at the bottom. Shown are 28 MD simulations.

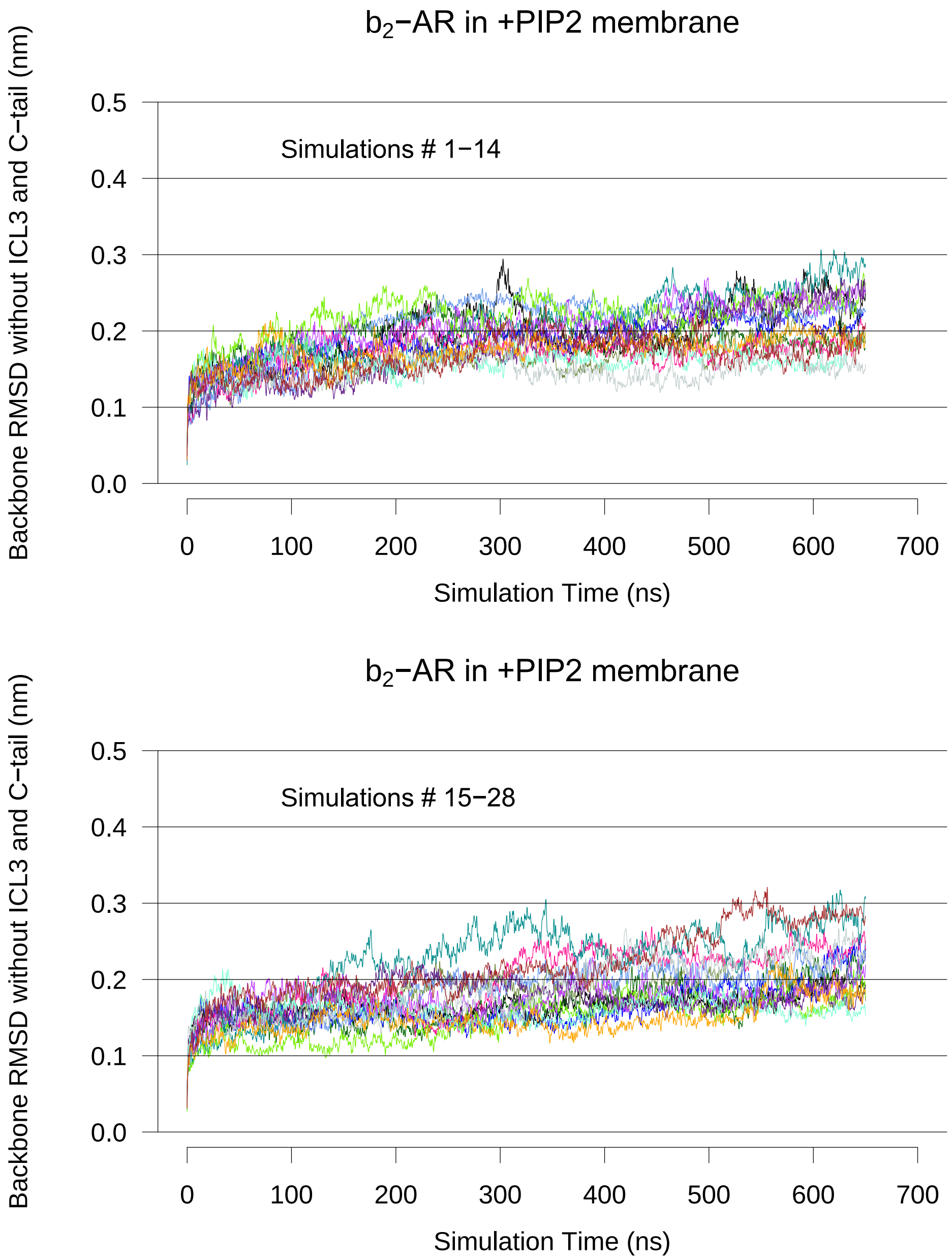

Supplementary Figure 42. Root mean square deviation (RMSD) over time of backbone atoms of nonphosphorylated β$_2$AR embedded in a membrane with PIP$_2$. The flexible ICL3 (residues 232-262) and C-tail (residues 342-378) were excluded from the analysis. The receptor was fitted on Cα atoms (without ICL3 and C-tail residues) prior to analysis. RMSD of MD simulations 1–14 is shown at the top and of MD simulations 15–28 at the bottom. Shown are 28 MD simulations.

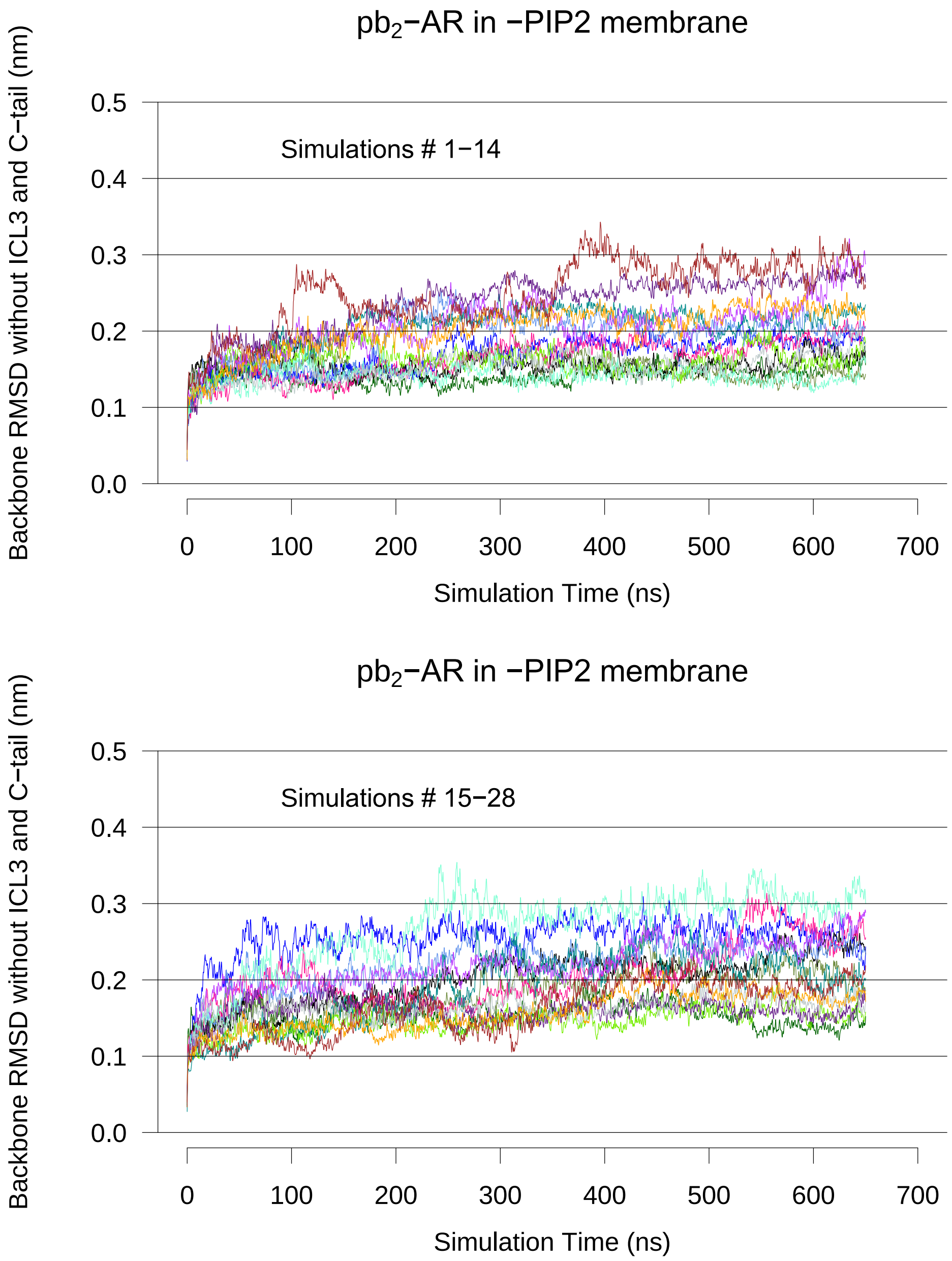

Supplementary Figure 43. Root mean square deviation (RMSD) over time of backbone atoms of phosphorylated β$_2$AR embedded in a membrane without PIP$_2$. The flexible ICL3 (residues 232–262) and C-tail (residues 342–378) were excluded from the analysis. The receptor was fitted on Cα atoms (without ICL3 and C-tail residues) prior to analysis. RMSD of MD simulations 1–14 is shown at the top and of MD simulations 15–28 at the bottom. Shown are 28 MD simulations.

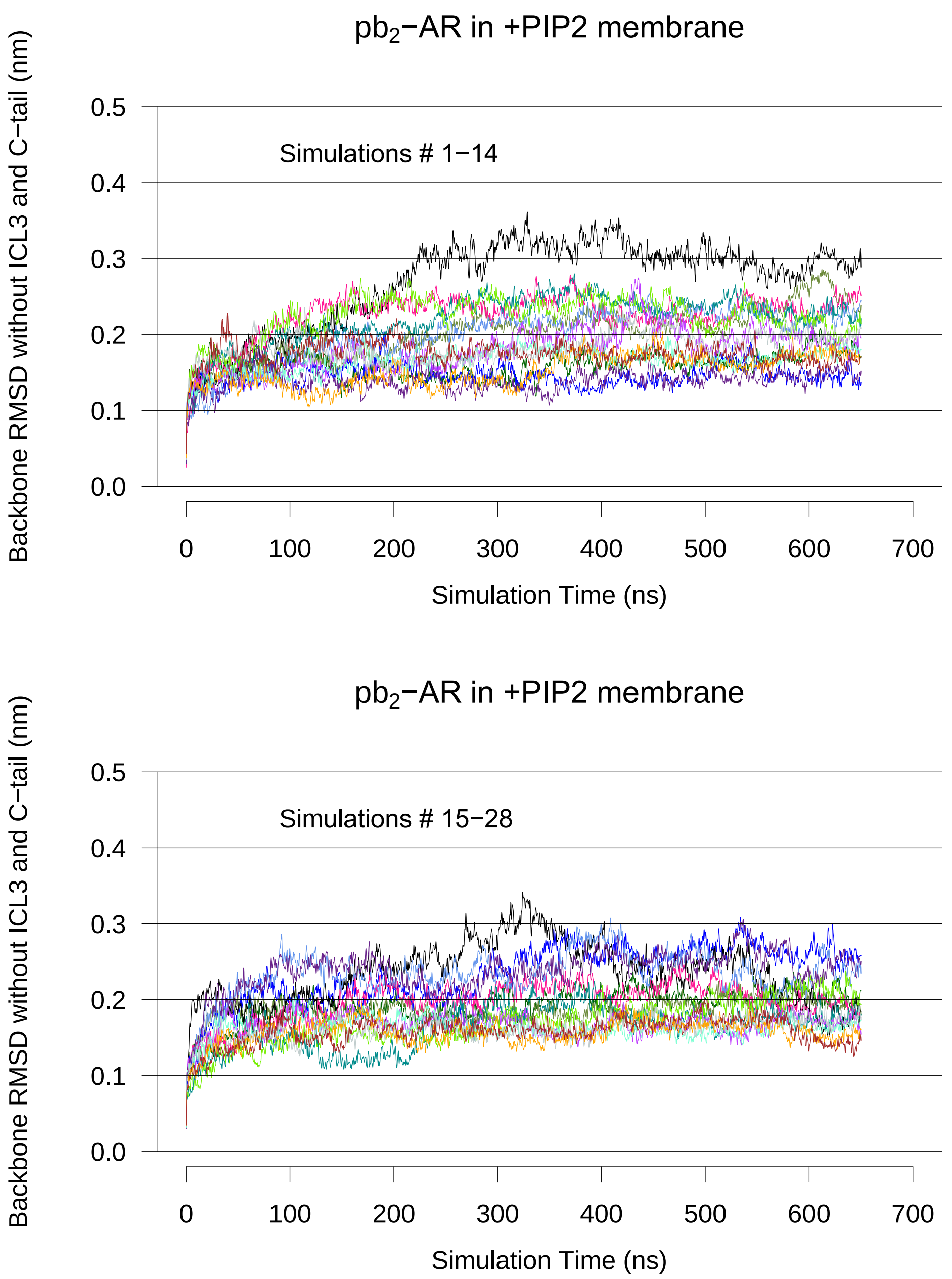

Supplementary Figure 44. Root mean square deviation (RMSD) over time of backbone atoms of phosphorylated β$_2$AR embedded in a membrane with PIP$_2$. The flexible ICL3 (residues 232–262) and C-tail (residues 342–378) were excluded from the analysis. The receptor was fitted on Cα atoms (without ICL3 and C-tail residues) prior to analysis. RMSD of MD simulations 1–14 is shown at the top and of MD simulations 15–28 at the bottom. Shown are 28 MD simulations.

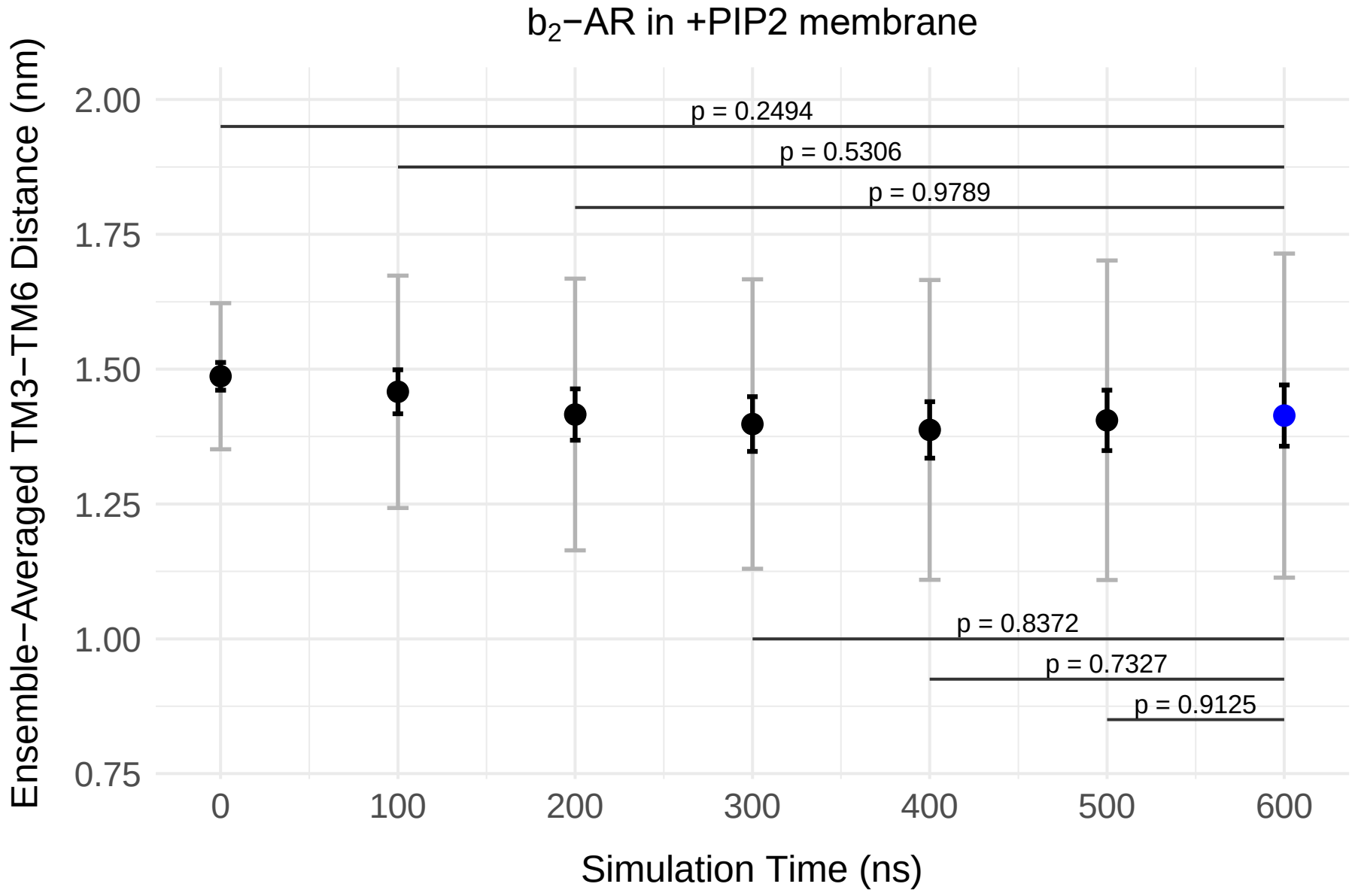

Supplementary Figure 45. TM3–TM6 distance averaged over 28 MD simulations in 100 ns time intervals for unphosphorylated β$_2$AR embedded in a membrane with PIP$_2$ (+PIP2). Black error bars indicate the SEM across all independent simulations, reflecting the uncertainty of the ensemble-averaged distance. Grey error bars represent the SD across all simulations within each block. Statistical significance between the final 550–650 ns block (highlighted in blue) and all other blocks was assessed using Welch's *t*-tests on per-simulation block averages (values shown in the figure). The value at 0 ns corresponds to the average distance at the initial time point**.**

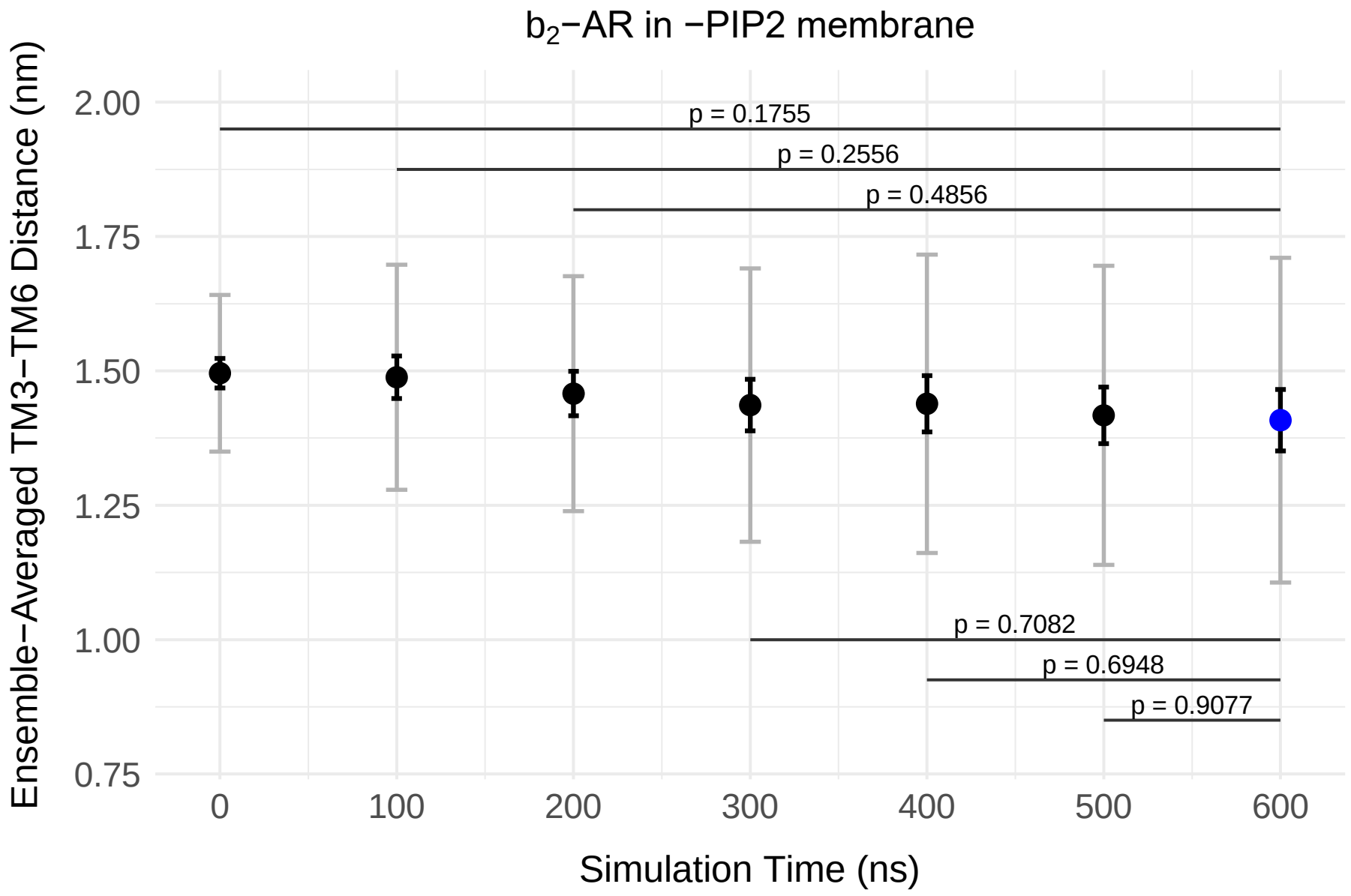

Supplementary Figure 46. TM3–TM6 distance averaged over 28 MD simulations in 100 ns time intervals for unphosphorylated β$_2$AR embedded in a membrane without PIP$_2$ (-PIP2). Black error bars indicate the SEM across all independent simulations, reflecting the uncertainty of the ensemble-averaged distance. Grey error bars represent the SD across all simulations within each block. Statistical significance between the final 550–650 ns block (highlighted in blue) and all other blocks was assessed using Welch's *t*-tests on per-simulation block averages (values shown in the figure). The value at 0 ns corresponds to the average distance at the initial time **point.**

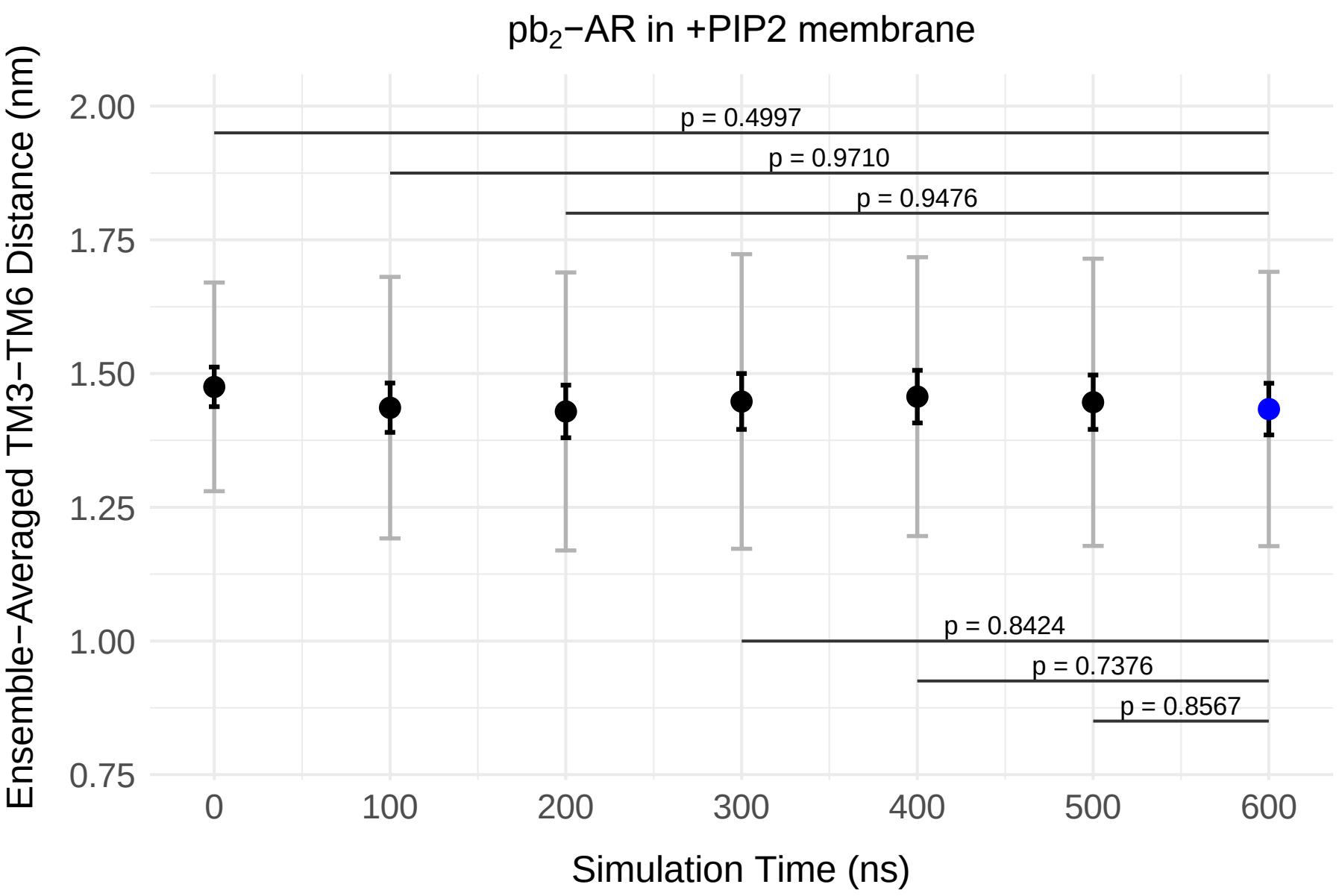

Supplementary Figure 47. TM3–TM6 distance averaged over 28 MD simulations in 100 ns time intervals for phosphorylated β$_2$AR embedded in a membrane with PIP$_2$ (+PIP2). Black error bars indicate the SEM across all independent simulations, reflecting the uncertainty of the ensemble-averaged distance. Grey error bars represent the SD across all simulations within each block. Statistical significance between the final 550–650 ns block (highlighted in blue) and all other blocks was assessed using Welch's t-tests on per-simulation block averages (values shown in the figure). The value at 0 ns corresponds to the average distance at the initial time point**.**

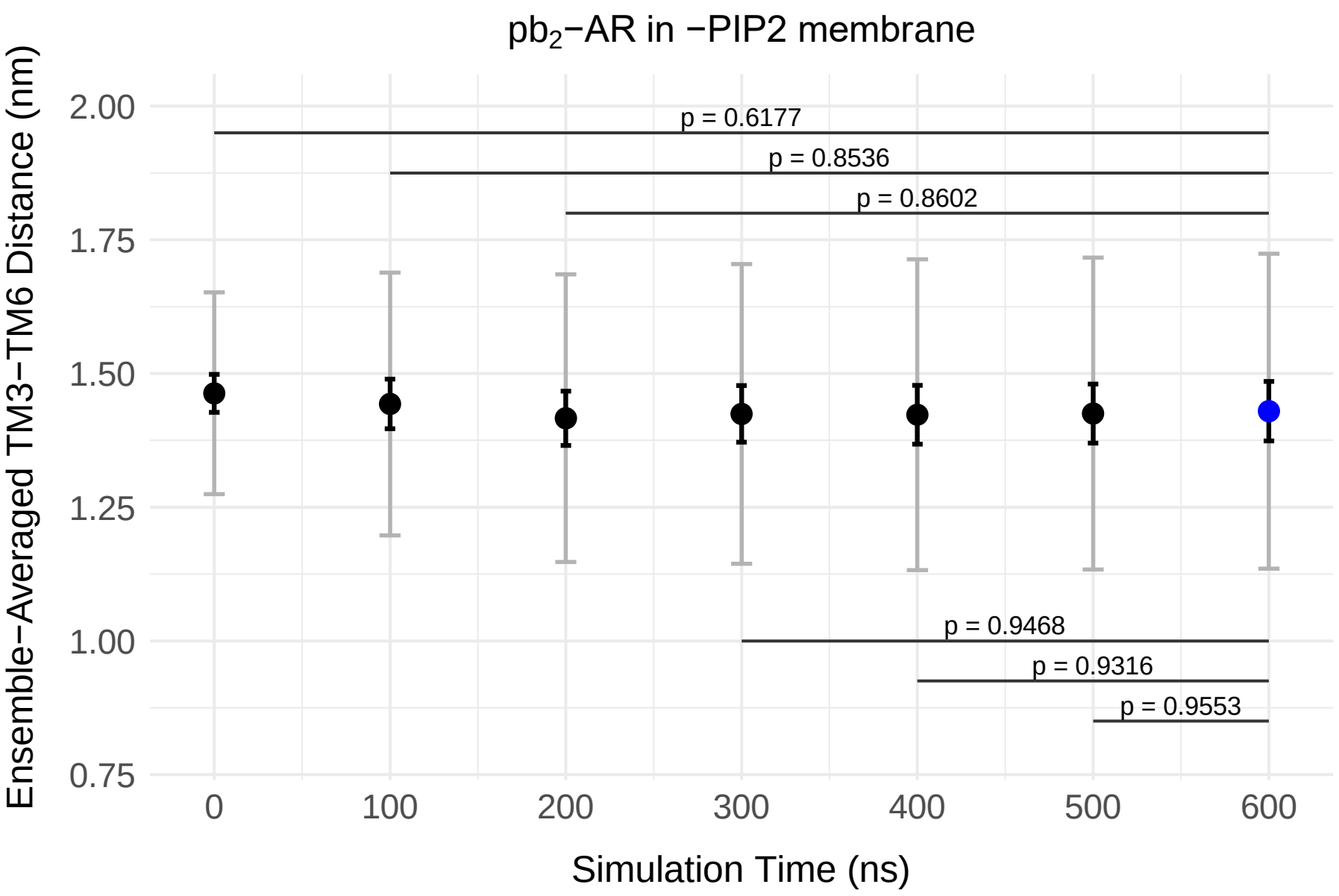

Supplementary Figure 48. TM3–TM6 distance averaged over 28 MD simulations in 100 ns time intervals for phosphorylated β$_2$AR embedded in a membrane without PIP$_2$ (-PIP2). Black error bars indicate the SEM across all independent simulations, reflecting the uncertainty of the ensemble-averaged distance. Grey error bars represent the SD across all simulations within each block. Statistical significance between the final 550–650 ns block (highlighted in blue) and all other blocks was assessed using Welch's *t*-tests on per-simulation block averages (values shown in the figure). The value at 0 ns corresponds to the average distance at the initial time point**.**

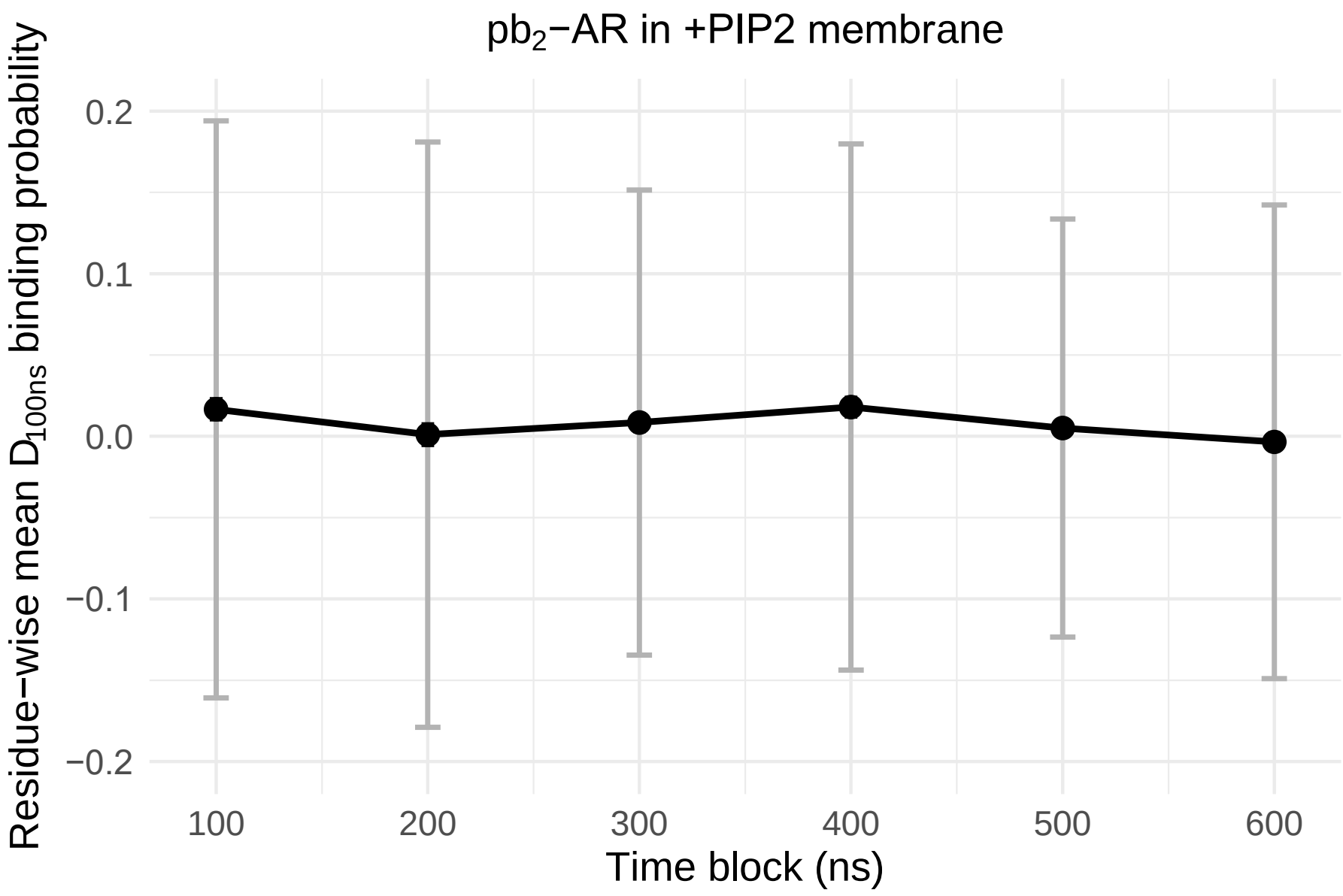

Supplementary Figure 49. ICL3 residue-wise membrane binding probability convergence over time for MD simulations of phosphorylated β_2AR embedded in a membrane with PIP_2. Mean changes in membrane binding probability (Δ100 ns) between consecutive 100 ns blocks were calculated for each ICL3 residue within each of the 28 MD simulations. The black line and points represent the mean Δ100 ns across residues and simulations, while error bars indicate the SD across residues and simulations.

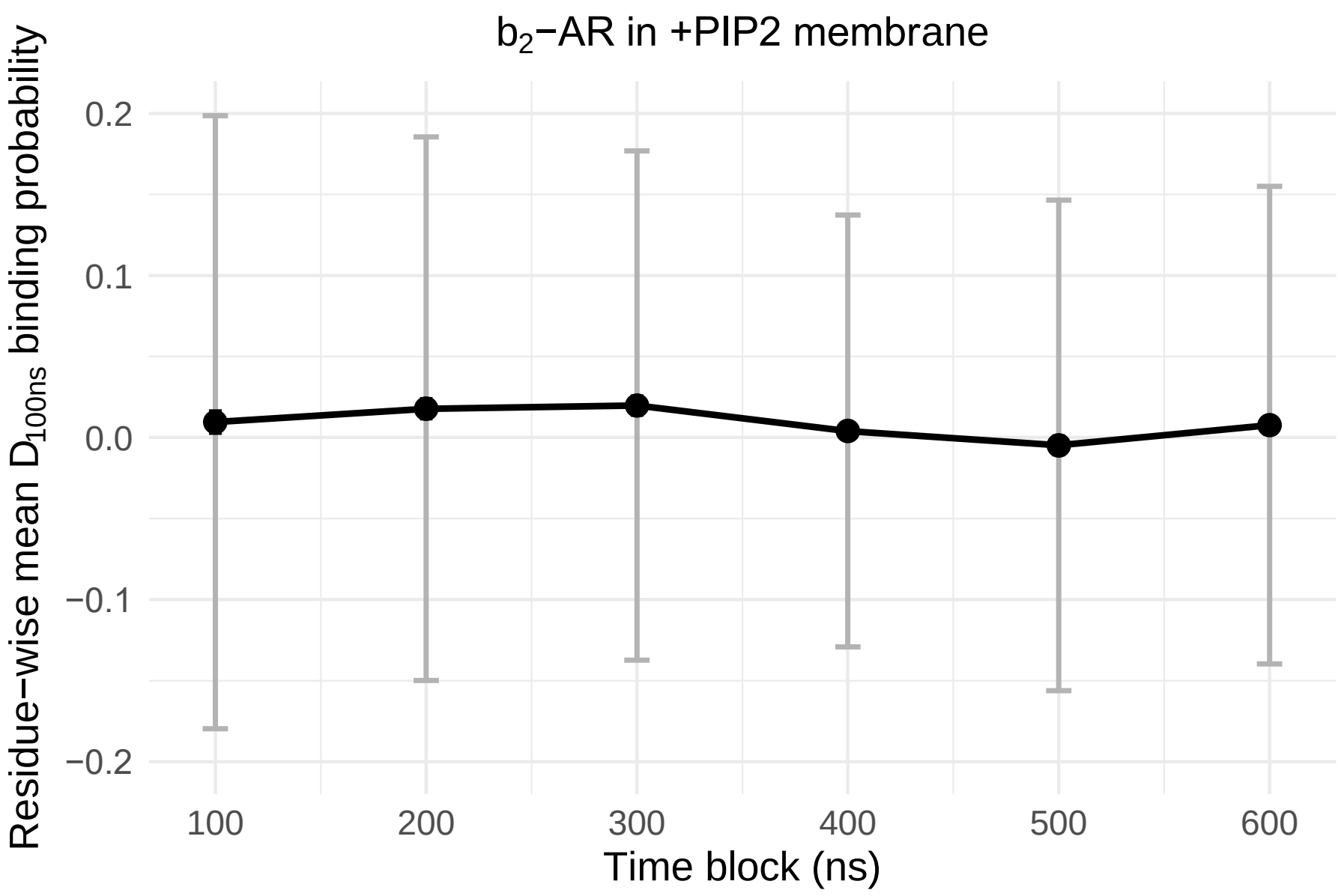

Supplementary Figure 50. ICL3 residue-wise membrane binding probability convergence over time for MD simulations of nonphosphorylated β_2AR embedded in a membrane with PIP_2. Mean changes in membrane binding probability (Δ100 ns) between consecutive 100 ns blocks were calculated for each ICL3 residue within each of the 28 MD simulations. The black line and points represent the mean Δ100 ns across residues and simulations, while the grey error bars indicate the SD across residues and simulations.

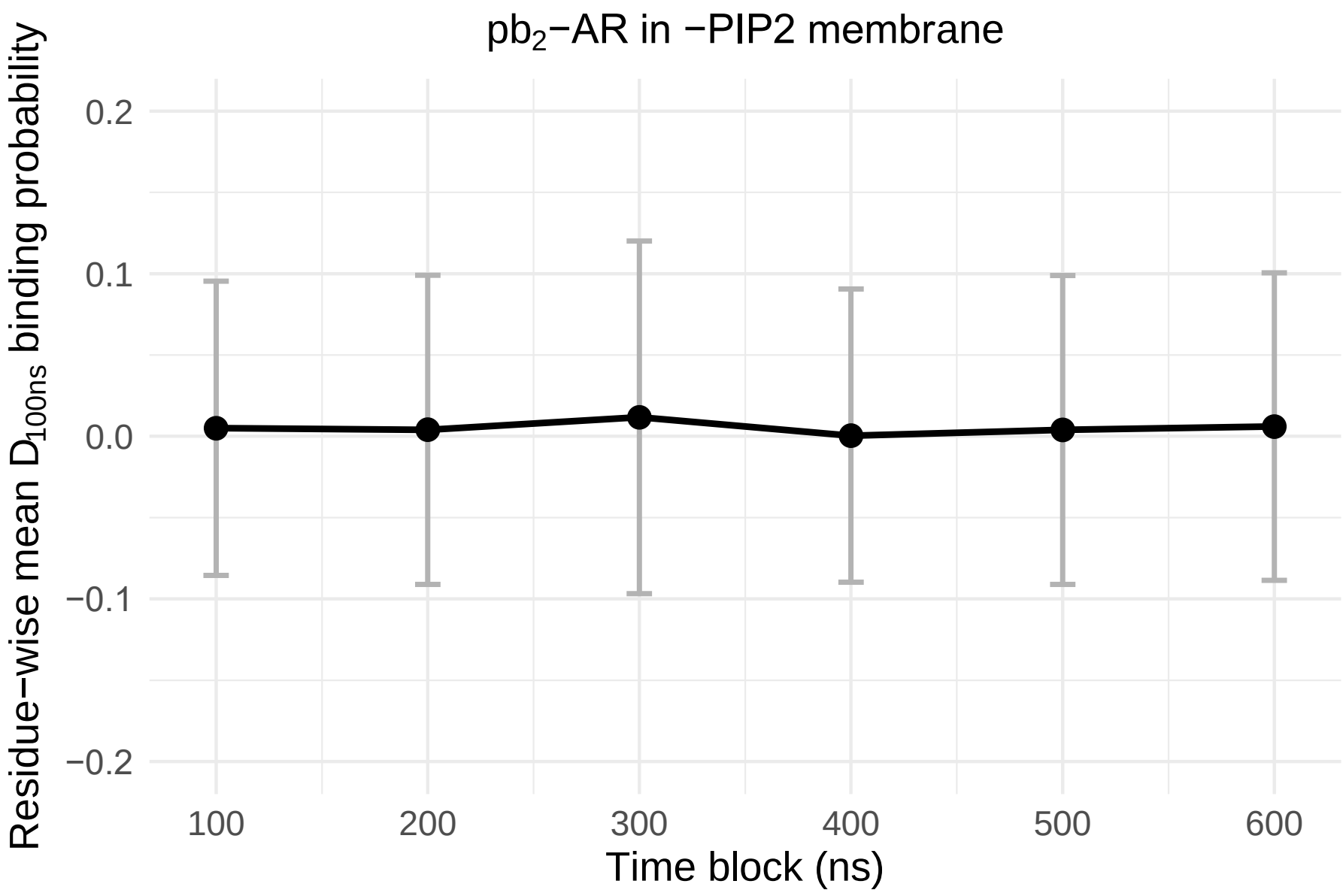

Supplementary Figure 51. ICL3 residue-wise membrane binding probability convergence over time for MD simulations of phosphorylated β$_2$AR embedded in a membrane without PIP$_2$. Mean changes in membrane binding probability (Δ100 ns) between consecutive 100 ns blocks were calculated for each ICL3 residue within each of the 28 MD simulations. The black line and points represent the mean Δ100 ns across residues and simulations, while the grey error bars indicate the SD across all residues and simulations.

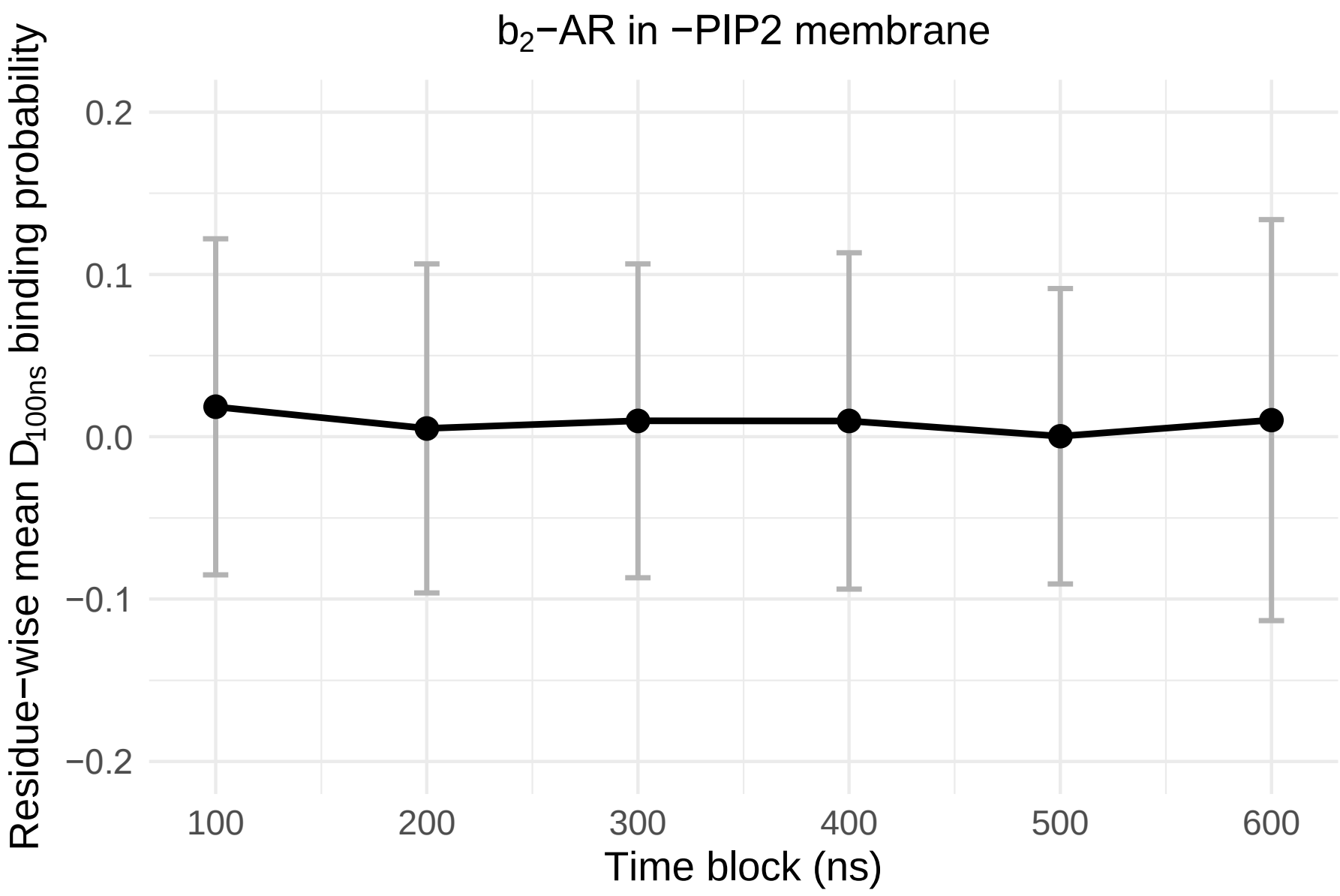

Supplementary Figure 52. ICL3 residue-wise membrane binding probability convergence over time for MD simulations of nonphosphorylated β$_2$AR embedded in a membrane without PIP$_2$. Mean changes in membrane binding probability (Δ100 ns) between consecutive 100 ns blocks were calculated for each ICL3 residue within each of the 28 MD simulations. The black line and points represent the mean Δ100 ns across residues and simulations, while the grey error bars indicate the SD across all residues and simulations.

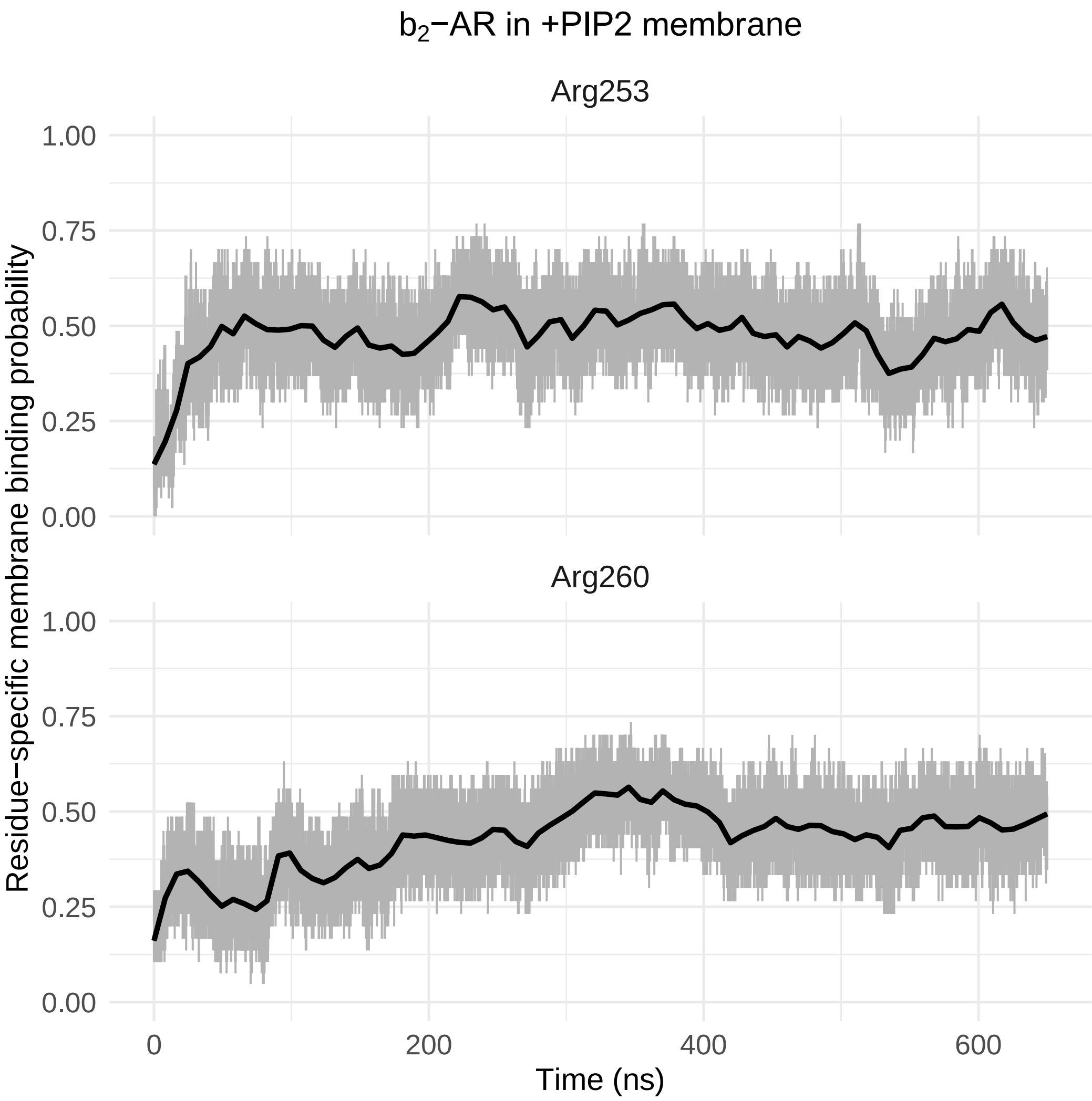

Supplementary Figure 53. ICL3 residue–specific membrane binding probabilities for MD simulations of nonphosphorylated β$_2$AR embedded in a membrane with PIP$_2$. Time-resolved membrane binding probabilities are exemplarily shown for two representative ICL3 residues, each averaged over 28 independent MD simulations. Black lines represent smoothed mean binding probabilities over time, while light grey error bars denote the SEM across all simulations.

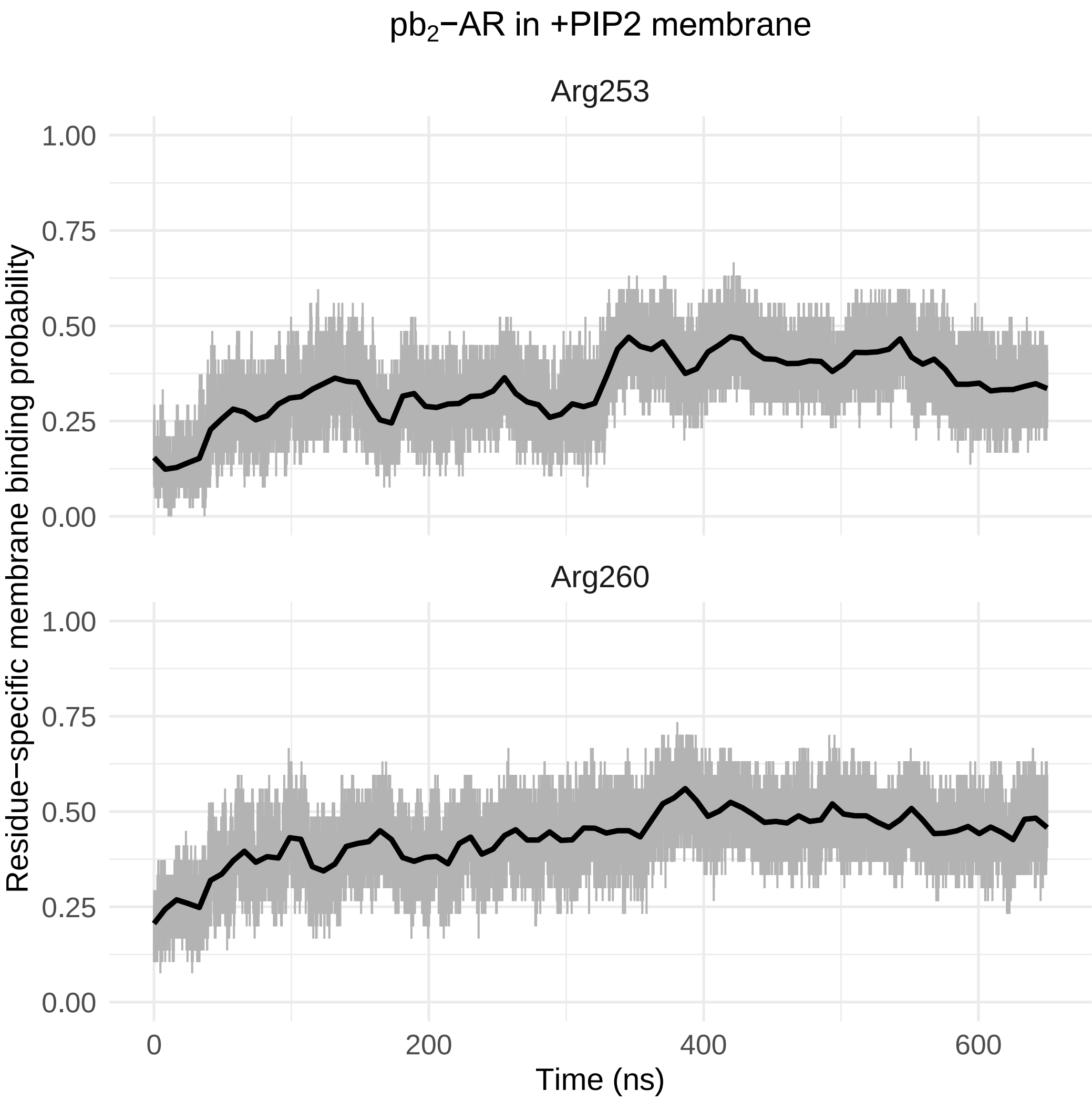

Supplementary Figure 54. ICL3 residue–specific membrane binding probabilities for MD simulations of phosphorylated β_2AR embedded in a membrane with PIP_2. Time-resolved membrane binding probabilities are exemplarily shown for two representative ICL3 residues, each averaged over 28 independent MD simulations. Black lines represent smoothed mean binding probabilities over time, while light grey error bars denote the SEM across all simulations.

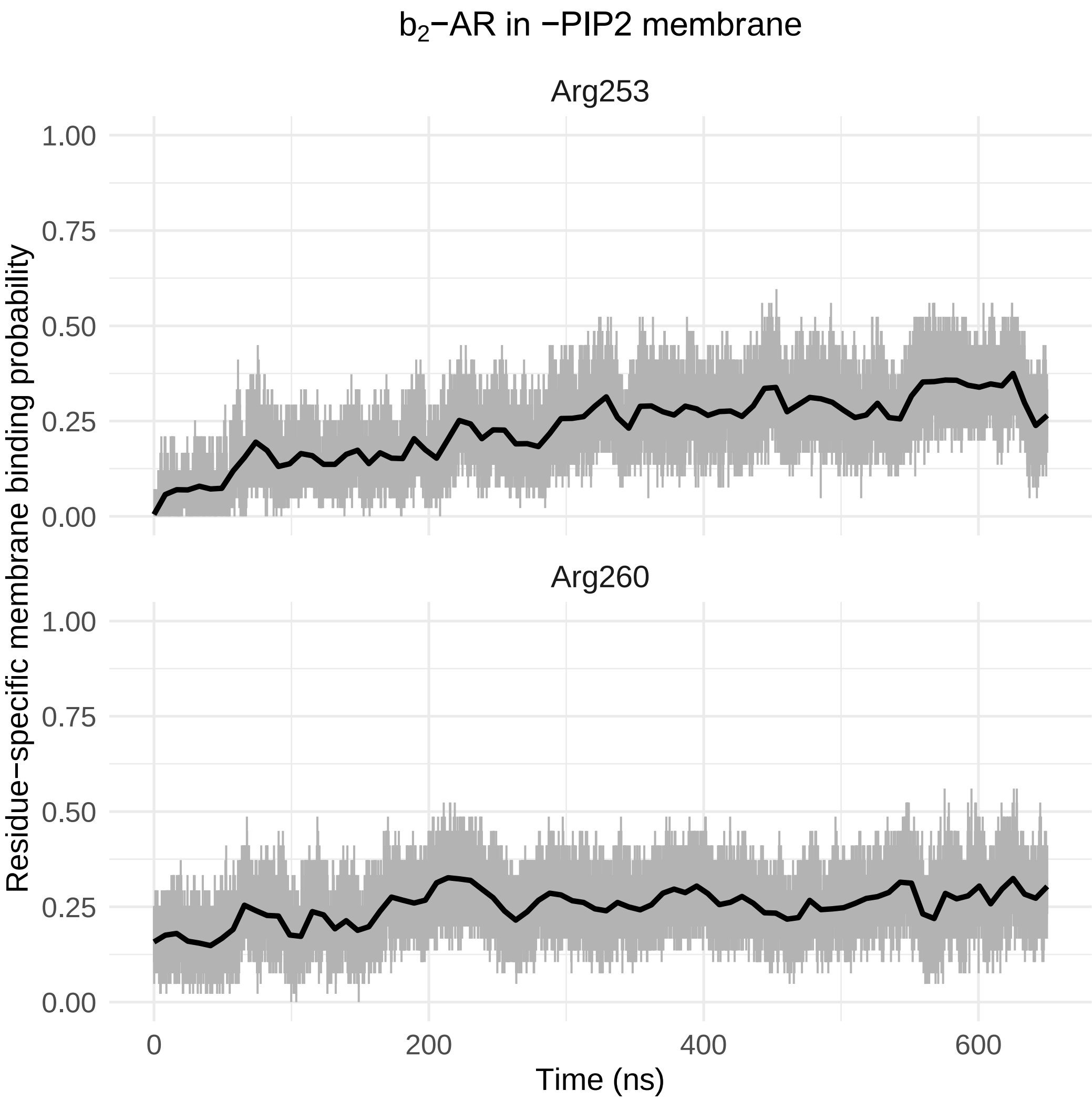

Supplementary Figure 55. ICL3 residue–specific membrane binding probabilities for MD simulations of nonphosphorylated β_2AR embedded in a membrane without PIP_2. Time-resolved membrane binding probabilities are exemplarily shown for two representative ICL3 residues, each averaged over 28 independent MD simulations. Black lines represent smoothed mean binding probabilities over time, while light grey error bars denote the SEM across all simulations.

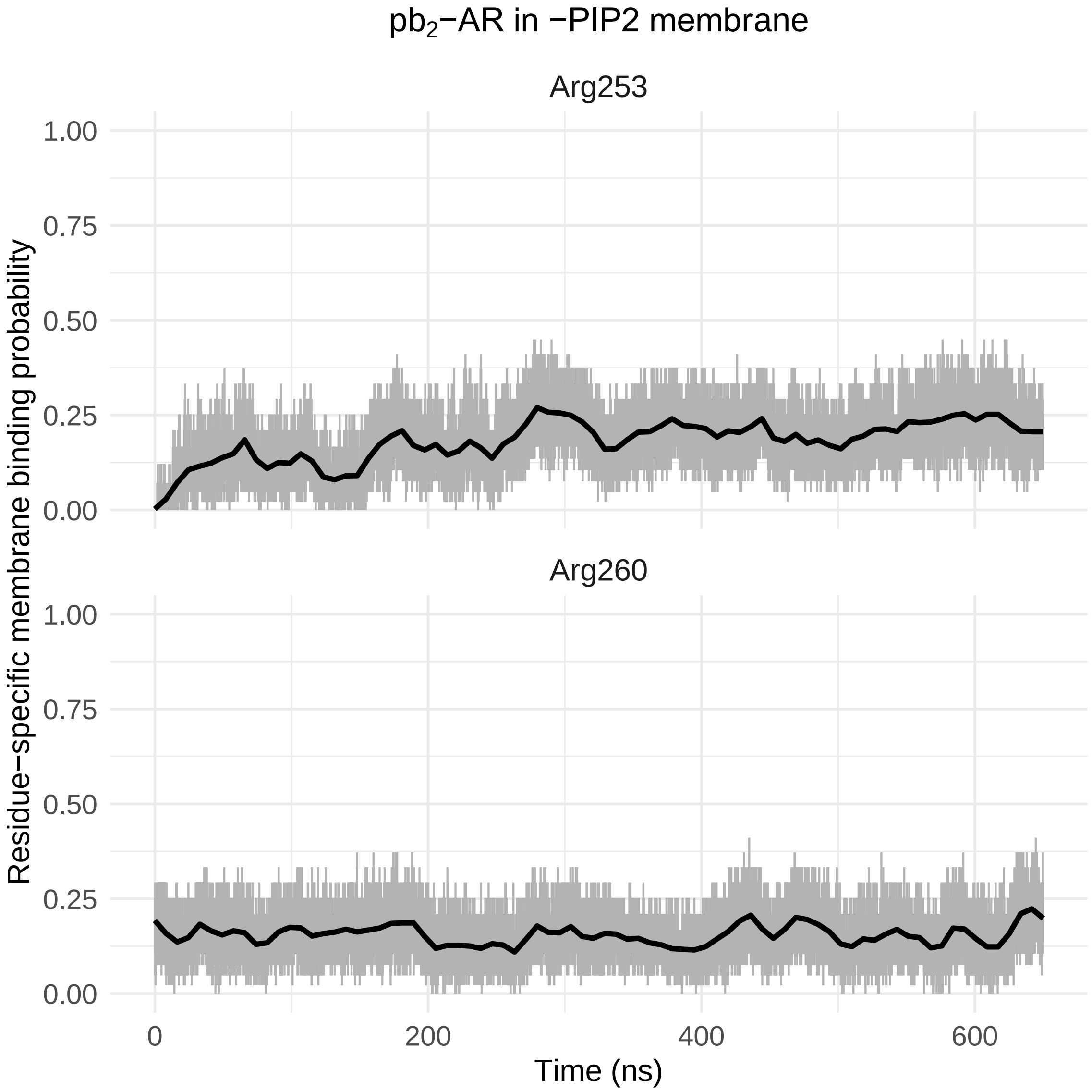

Supplementary Figure 56. ICL3 residue–specific membrane binding probabilities for MD simulations of phosphorylated β$_2$AR embedded in a membrane without PIP$_2$. Time-resolved membrane binding probabilities are exemplarily shown for two representative ICL3 residues, each averaged over 28 independent MD simulations. Black lines represent smoothed mean binding probabilities over time, while light grey error bars denote the SEM across all simulations.

Supplementary References

1 Dufrene, Y. F., Martinez-Martin, D., Medalsy, I., Alsteens, D. & Muller, D. J. Multiparametric imaging of biological systems by force-distance curve-based AFM. *Nat Methods* **10**, 847-854 (2013). https://doi.org/10.1038/nmeth.2602

2 Evans, E. & Ritchie, K. Dynamic strength of molecular adhesion bonds. *Biophys J* **72**, 1541-1555 (1997). https://doi.org/10.1016/S0006-3495(97)78802-7
