# Supplementary material for: Factors modulating the assembly of human β2-adrenergic receptor–β-arrestin complexes
Source: Nat Struct Mol Biol. 2026 Jul 31;33(8):1158–70. doi: 10.1038/s41594-026-01842-3 (PMC13468150; doi:10.1038/s41594-026-01842-3)
Supplement: Supplementary file 2 [file 41594_2026_1842_MOESM2_ESM.pdf]

nature portfolio

Corresponding author(s): Daniel J. Muller

Last updated by author(s): Jun 23, 2026

Reporting Summary

Nature Portfolio wishes to improve the reproducibility of the work that we publish. This form provides structure for consistency and transparency in reporting. For further information on Nature Portfolio policies, see our Editorial Policies and the Editorial Policy Checklist.

Statistics

For all statistical analyses, confirm that the following items are present in the figure legend, table legend, main text, or Methods section.

n/a	Confirmed	
☐	☒	The exact sample size (*n*) for each experimental group/condition, given as a discrete number and unit of measurement
☐	☒	A statement on whether measurements were taken from distinct samples or whether the same sample was measured repeatedly
☐	☒	The statistical test(s) used AND whether they are one- or two-sided<br>*Only common tests should be described solely by name; describe more complex techniques in the Methods section.*
☐	☒	A description of all covariates tested
☒	☐	A description of any assumptions or corrections, such as tests of normality and adjustment for multiple comparisons
☐	☒	A full description of the statistical parameters including central tendency (e.g. means) or other basic estimates (e.g. regression coefficient) AND variation (e.g. standard deviation) or associated estimates of uncertainty (e.g. confidence intervals)
☐	☒	For null hypothesis testing, the test statistic (e.g. *F*, *t*, *r*) with confidence intervals, effect sizes, degrees of freedom and *P* value noted<br>*Give P values as exact values whenever suitable.*
☒	☐	For Bayesian analysis, information on the choice of priors and Markov chain Monte Carlo settings
☒	☐	For hierarchical and complex designs, identification of the appropriate level for tests and full reporting of outcomes
☒	☐	Estimates of effect sizes (e.g. Cohen's *d*, Pearson's *r*), indicating how they were calculated

Our web collection on statistics for biologists contains articles on many of the points above.

Software and code

Policy information about availability of computer code

Data collection	Bruker Nanoscope (version 9.4R3), Burst-Analyzer (version 2.0), GROMACS (versions 2021 & 2023)
Data analysis	R (version 4.2.3), RStudio (version 2024.12.0+467), MATLAB (version 2018b), GraphPad Prism (versions 9 and newer), Origin (version 2019), PyMOL (version 2.5)

For manuscripts utilizing custom algorithms or software that are central to the research but not yet described in published literature, software must be made available to editors and reviewers. We strongly encourage code deposition in a community repository (e.g. GitHub). See the Nature Portfolio guidelines for submitting code & software for further information.

Data

Policy information about availability of data

All manuscripts must include a data availability statement. This statement should provide the following information, where applicable:

- Accession codes, unique identifiers, or web links for publicly available datasets
- A description of any restrictions on data availability
- For clinical datasets or third party data, please ensure that the statement adheres to our policy

The raw data generated in this study and used to generate the main figures, Extended Data and Supplementary Information are available from Universität Stuttgart at https://doi.org/10.18419/DARUS-5513. The source data for the Supplementary Information section ‘MD data analysis’ have also been deposited under the same DOI. Additional data and materials are available from the corresponding author upon reasonable request. Source data are provided with this paper.

Research involving human participants, their data, or biological material

Policy information about studies with human participants or human data. See also policy information about sex, gender (identity/presentation), and sexual orientation and race, ethnicity and racism.

Reporting on sex and gender	NA
Reporting on race, ethnicity, or other socially relevant groupings	NA
Population characteristics	NA
Recruitment	NA
Ethics oversight	NA

Note that full information on the approval of the study protocol must also be provided in the manuscript.

Field-specific reporting

Please select the one below that is the best fit for your research. If you are not sure, read the appropriate sections before making your selection.

☒ Life sciences ☐ Behavioural & social sciences ☐ Ecological, evolutionary & environmental sciences

For a reference copy of the document with all sections, see nature.com/documents/nr-reporting-summary-flat.pdf

Life sciences study design

All studies must disclose on these points even when the disclosure is negative.

Sample size	The sample size for experiments was determined by calculating the number of adhesion events or simulations. The SMFS samples were taken from at least three independent experiments (where one AFM tip functionalization constitutes one independent experiment), with each experiment investigating at least one new biological sample preparation, buffer solution, AFM cantilever, etc. Within each experiment several hundreds of force-distance curves were experimentally collected and analyzed.<br>No formal statistical power calculation was performed to predetermine sample sizes for molecular dynamics (MD) simulations. Instead, sample sizes were selected based on established practice in the field and computational feasibility. For each condition, at least eight independent MD replicas ($N \geq 8$) were generated using different initial models. This number of replicas is commonly used to ensure adequate sampling of conformational space and to assess reproducibility. Convergence and robustness were evaluated by comparing key observables (e.g., structural metrics, interaction frequencies) across replicas. This approach is consistent with current recommendations emphasizing ensemble-based MD simulations and reproducibility assessment rather than reliance on single trajectories.
Data exclusions	SMFS adhesion events weaker than five times the standard deviation of the baseline noise of the experiment (≈ 15 pN) were excluded from the analysis. Also, adhesion events between barr2 and b2AR which occurred closer than 3 nm to the tip-sample contact point (to avoid taking into account non-specific tip-sample interaction events) and further than the pre-defined distance of 20 nm (to avoid taking into account binding events longer than the stretching of the PEG-spacer such as occurring from macromolecular aggregates adhering to the cantilever tip) from the tip-sample contact point. Also force-distance curves containing multiple adhesion events were excluded as they likely originated from non-specific interactions. The pre-defined distance of 20 nm is determined based on the length of the PEG linker and the barr2 attached to the AFM tip.
Replication	Some attempts for SMFS experiments were unsuccessful due to failed membrane protein reconstitution or failed chemical modification of the AFM tip. To compensate for such unsuccessful experiments many experimental repetitions have been conducted. The adhesion forces analyzed orignate from at least three independent SMFS experiments at which the barr2 had been successfully chemically attached to the AFM tip.
Randomization	When multiple AFM-tip-sample interaction times were recorded from an experimental sample, the acquisition of contact times was randomized. SMFS experiments were repeated at least three independent times. Each of the biological samples characterized had been clearly labeled and biochemically characterized before of the experiments.
Blinding	Blinding during data collection does not alter the experimental outcome because all atomic force microscopy settings were pre-set with changes only in the AFM-tip-sample interaction time or cantilever velocity as indicated. A control of the data analysis by a blinded researcher from outside of the list of authors confirmed the outcome of the analysis.

Reporting for specific materials, systems and methods

We require information from authors about some types of materials, experimental systems and methods used in many studies. Here, indicate whether each material, system or method listed is relevant to your study. If you are not sure if a list item applies to your research, read the appropriate section before selecting a response.

Materials & experimental systems

n/a	Involved in the study
☐	☒ Antibodies
☐	☒ Eukaryotic cell lines
☒	☐ Palaeontology and archaeology
☒	☐ Animals and other organisms
☒	☐ Clinical data
☒	☐ Dual use research of concern
☒	☐ Plants

Methods

n/a	Involved in the study
☒	☐ ChIP-seq
☒	☐ Flow cytometry
☒	☐ MRI-based neuroimaging

Antibodies

Antibodies used	Nanobody 6B9 (Nb6B9) was expressed and purified as detailed in the Methods
Validation	The specific binding of the Nb689 was validated by detecting the binding to specific epitopes (BI-167107 activated b2AR) through fluorescence and force spectroscopy. Binding to non-specific eptiopes (ICI-118551 inhibited b2AR) was not observed. This is described in the section “Monitoring spontaneous binding events of barr2 to b2AR”. In addition, Nb6B9 hass previously been described in Ring, A.M. et al. Adrenaline-activated structure of beta2-adrenoceptor stabilized by an engineered nanobody. Nature 502, 575-579 (2013).

Eukaryotic cell lines

Policy information about cell lines and Sex and Gender in Research

Cell line source(s)	Sf9, Expression Systems, Cat 94-001F.
Authentication	Cell lines are maintained by the supplier. No additional authentication was performed by the authors of this study.
Mycoplasma contamination	Cell lines are tested by manufacturer for contamination, but were not further tested by the authors of this study.
Commonly misidentified lines (See ICLAC register)	No commonly misidentified lines were used in this study

Plants

Seed stocks	NA
Novel plant genotypes	NA
Authentication	NA
