# Supplementary material for: Factors modulating the assembly of human β2-adrenergic receptor–β-arrestin complexes
Source: Nat Struct Mol Biol. 2026 Jul 31;33(8):1158–70. doi: 10.1038/s41594-026-01842-3 (PMC13468150; doi:10.1038/s41594-026-01842-3)
Supplement: Supplementary file 3 [file 41594_2026_1842_MOESM3_ESM.pdf]

nature portfolio

Peer Review File

Factors modulating the assembly of human β2-adrenergic receptor–β-arrestin complexes

Corresponding Author: Professor Daniel Mueller

Version 0:

Decision Letter:

25th Apr 2025

Dear Dr. Müller,

I sincerely apologize for the delay in sharing our decision with you -- I am writing now on behalf of my colleague Dr Ciazynska, who is out of the office.

Thank you again for submitting your manuscript "Roles of ligand, phosphorylation, and membrane in assembling human β2-adrenergic receptor-β-arrestin complexes". I apologize again for the delay in responding, which resulted from the difficulty in obtaining suitable referee reports. Nevertheless, we now have comments (below) from the 3 reviewers who evaluated your paper. **Please note that Rev#1 and Rev#3 co-reviewed the manuscript, and so we are only sharing their comment once, under "Rev#1".** In light of those reports, we remain interested in your study and would like to see your response to the comments of the referees, in the form of a revised manuscript.

You will see that the reviewers found the data interesting but raised substantial concerns that undermine the strength of the novel conclusions, data, and interpretation. For reconsideration at the journal, we would expect you to address thoroughly all of the reviewers' questions with what the approach can detect (all reviewers) and the concerns about the strength of the interpretation, data analysis, and conclusions, providing additional experimentation to bolster the model, especially with regards to the impact of PIP2, as per Rev#1-3. Resolving conflicts with published work should also be a priority in revision. Lastly, please address the reviewers' minor and technical points, requests for discussion, and questions about statistical analyses.

Please do not hesitate to contact us directly if you would like to discuss these issues further. Please be sure to respond to all concerns of the referees in full in a point-by-point response and highlight all changes in the revised manuscript text file.

Our revision period is ~six months and we are committed to providing a fair and constructive peer-review process. If you cannot send the revision within ~6 months, please let us know. We will be happy to consider your revision as long as nothing similar has been accepted for publication at NSMB or published elsewhere. Should your manuscript be substantially delayed without notifying us in advance and your article is eventually published, the received date would be that of the revised, not the original, version.

As you already know, we put great emphasis on ensuring that the methods and statistics reported in our papers are correct and accurate. As such, if there are any changes that should be reported, please submit an updated version of the Reporting Summary along with your revision.

Reporting Summary:
https://www.nature.com/documents/nr-reporting-summary.pdf

Please note that the form is a dynamic ‘smart pdf’ and must therefore be downloaded and completed in Adobe Reader.

When submitting the revised version of your manuscript, please pay close attention to our href="https://www.nature.com/nature-portfolio/editorial-policies/image-integrity">Digital Image Integrity Guidelines. and to the following points below:

-- that unprocessed scans are clearly labelled and match the gels and western blots presented in figures.
-- that control panels for gels and western blots are appropriately described as loading on sample processing controls
-- all images in the paper are checked for duplication of panels and for splicing of gel lanes.

EXTENDED DATA FIGURES

When re-submitting your manuscript, please ensure that any supplementary figures and tables that are crucial to the manuscript's conclusions are converted into Extended Data figures and tables to increase visibility of these data. Extended Data figures and tables are online-only (present in the online PDF and full-text HTML versions of the paper), peer-reviewed display items that provide essential background to the article but are not included in the main article due to space constraints. A maximum of ten Extended Data display items (figures and tables) is permitted.

Finally, please ensure that you retain unprocessed data and metadata files after publication, ideally archiving data in perpetuity, as these may be requested during the peer review and production process or after publication if any issues arise.

If there are additional or modified structures presented in the final revision, please submit the corresponding PDB validation reports.

Please note that all key data shown in the main figures as cropped gels or blots should be presented in uncropped form, with molecular weight markers. These data can be aggregated into a single supplementary figure. While these data can be displayed in a relatively informal style, they must refer back to the relevant figures. These data should be submitted with the last revision, prior to acceptance, but you may want to start putting it together at this point.

SOURCE DATA: we urge authors to provide, in tabular form, the data underlying the graphical representations used in figures. This is to further increase transparency in data reporting, as detailed in this editorial (http://www.nature.com/nsmb/journal/v22/n10/full/nsmb.3110.html). Spreadsheets can be submitted in excel format. Only one (1) file per figure is permitted; thus, for multi-paneled figures, the source data for each panel should be clearly labeled in the Excel file; alternately the data can be provided as multiple, clearly labeled sheets in an Excel file. When submitting files, the title field should indicate which figure the source data pertains to. We encourage our authors to provide source data at the revision stage, so that they are part of the peer-review process.

While we encourage the use of color in preparing figures, please note that this will incur a charge to partially defray the cost of printing. Information about color charges can be found at http://www.nature.com/nsmb/authors/submit/index.html#costs

We require deposition of coordinates (and, in the case of crystal structures, structure factors) into the Protein Data Bank with the designation of immediate release upon publication (HPUB). Electron microscopy-derived density maps and coordinate data must be deposited in EMDB and released upon publication. Deposition and immediate release of NMR chemical shift assignments are highly encouraged. Deposition of deep sequencing and microarray data is mandatory, and the datasets must be released prior to or upon publication. To avoid delays in publication, dataset accession numbers must be supplied with the final accepted manuscript and appropriate release dates must be indicated at the galley proof stage. Please find the complete NRG policies on data availability at http://www.nature.com/authors/policies/availability.html.

Nature Structural & Molecular Biology is committed to improving transparency in authorship. As part of our efforts in this direction, we are now requesting that all authors identified as 'corresponding author' on published papers create and link their Open Researcher and Contributor Identifier (ORCID) with their account on the Manuscript Tracking System (MTS), prior to acceptance. This applies to primary research papers only. ORCID helps the scientific community achieve unambiguous attribution of all scholarly contributions. You can create and link your ORCID from the home page of the MTS by clicking on 'Modify my Springer Nature account'. For more information please visit please visit www.springernature.com/orcid.

Please use the link below to submit your revised manuscript and related files:

Link Redacted

Note: This URL links to your confidential home page and associated information about manuscripts you may have submitted, or that you are reviewing for us. If you wish to forward this email to co-authors, please delete the link to your homepage.

We look forward to seeing the revised manuscript and thank you for the opportunity to review your work. Once again I am very sorry the peer review process took so long.

Sincerely,

Melina Casadio, PhD
Locum Chief Editor, Nature Structural & Molecular Biology
ORCID ID: https://orcid.org/0000-0003-2389-2243

Referee expertise:

Referee #1, #3: GPCRs, arrestins

Referee #2: AFM

Reviewers' Comments:

Reviewer #1 (Remarks to the Author):

The manuscript, "Roles of ligand, phosphorylation, and membrane in assembling human β2-adrenergic 2 receptor-β-arrestin complexes" describes the use of dynamic atomic force spectroscopy (AFM)-based single molecule force spectroscopy (SMFS) and predictive atomistic molecular dynamics (MD) simulations to characterize β-arrestin2 interactions with the beta-2 adrenergic receptor (β2AR) and with membranes. The authors covalently tether purified β-arrestin2 to the tip of the AFM cantilever, which hovers over purified β2AR reconstituted in an artificial DOPC:DOPG:cholesterol lipid environment absorbed to a mica surface. This lipid environment is doped with or without phosphatidylinositol 4,5-bisphosphate (PIP2), a minor phospholipid involved in many cellular signaling pathways and also shown in prior reports to directly bind β-arrestins, regulating their interactions with receptors and ability to drive clathrin-mediated receptor endocytosis. The AFM tip is brought near the β2AR-embedded membranes, such that β-arrestin can interact with the receptor or membrane. A tip-sample interaction time of 21 ms or 121 ms is used, after which the cantilever is retracted at different speeds. From these measurements, the percentage of β-arrestin-receptor binding events is determined, and the resulting rupture force is used to extract their kinetic and thermodynamic characteristics. By strategically using full (BI-167107) and inverse (ICI-118551) β2AR agonists, the authors control whether β-arrestin2 can interact with the receptor core, as well as using β2AR in the absence or presence of GRK5-mediated phosphorylation to control receptor C-tail engagement with the phosphate sensing groove in β-arrestin2. Using these strategies, the authors are able to characterize the biophysical properties of the receptor tail only, core only, and tail+core complexes with β-arrestin2. Because β-arrestin2 has been reported to be preassociated with the plasma membrane, the authors use the same technique to quantify the dynamics of β-arrestin2 interactions with the absorbed membranes in the absence of embedded β2AR.

Using their approach, the authors provide further support of the existence of the receptor tail (or hanging conformation), core, and tail+core (or tight complex) complexes between β-arrestins and receptor as well as the interaction of β-arrestin with the membrane and PIP2 in the absence of receptor binding, all of which have been reported previously. What sets this work apart from the prior studies is that the authors present a deeper understanding of the kinetic and thermodynamic properties of these various complexes, as well as how these properties are affected in the absence and presence of PIP2. Arguably, the most novel and intriguing aspect of this work is the finding that PIP2 stabilizes the receptor-β-arrestin2 core interaction, but surprisingly hinders β-arrestin2 from concomitantly binding the phosphorylated receptor C-tail. While this finding and their other experiments shed light on how PIP2 regulates the stability of the β2AR-β-arrestin2 complex, how PIP2 exerts its effects is less clear. The computational findings suggest that PIP2 binding to β-arrestin itself is primarily responsible for these effects, but this is not tested experimentally with respect to the receptor-arrestin interaction as discussed below. Furthermore, there seems to be a bit of disconnect between the conclusion here and from previous work that PIP2 is essential for β-arrestin recruitment to class A receptors such as the β2AR studied here and the fact that tail+core interactions are observed and relatively stable in the complete absence of PIP2, as well as the finding here that in the presence of PIP2 the core interaction is stable, despite the fact that tail phosphorylation seems critical for in β-arrestin recruitment in cells. These apparent disconnects are not discussed, and doing so would strengthen the manuscript and its impact.

Major Comments

• Page 3, lines 138-139. "The results demonstrate that our experimental setup can detect and quantify the force, lifetime, and width of the free-energy valley from single binding events of βarr2 and β2AR." The authors call this technique "AFM-based single-molecule force spectroscopy (SMFS)," but based on how they prepare the samples, how can the authors know that they are probing single binding events between individual β2AR and βarr2 molecules? Indeed, on line 114 the authors comment that single or groups or receptors can be seen in the membrane. It seems likely that the receptors (white spots) that are sparsely distributed and protruding from the phospholipid membrane (Supplementary Figs. 1d-g)) represent a mix of individual and multiple aggregated receptors – this would seem to explain why some of these spots appear larger in diameter. Moreover, Supplementary Fig 2b, it appears that the bright GFP coincides with the larger spots, which would also suggest that we are seeing receptor aggregates, as the brightness seems substantially greater than the GFP for the smaller white spots. There is no discussion of how the results might be impacted by the availability of multiple receptors on the surface. Likewise, and perhaps most salient, in the schematic shown in Supplementary Fig. 3, it appears likely that multiple β-arrestin molecules could be attached to a single AFM tip. Do the authors have data suggesting that only one β-arrestin2 molecule can attach to the tip? If many β-arrestin molecules interact with a white spot representing possibly many receptors, can the force, lifetime, and width of the free-energy valleys quantified in this study be characterized as information related to a binding event between single β2AR and β-arrestin2 molecules? The sample data in Fig 1a could easily be interpreted as a relaxation to an intermediate force, followed by a second step, possibly with brief transitions to the intermediate step throughout the first phase. Clearly, as described in the methods, there is extensive filtering of the data that can only be appreciated by an expert in AFM, but these issues must be discussed.

• Lines 237-246, page 5. "Taken together, the results suggest that within ≈21 ms βarr2 can assemble a kinetically, mechanically, and thermodynamically stable complex with the phosphorylated C-tail of β2AR. Concurrent binding of βarr2 to the tail and core of β2AR or the core alone resulted in complexes with shorter lifetimes, narrower free-energy valleys, and lower free-energy barriers". It appears the authors are comparing the 21 ms interaction time data for the tail only complex data (Fig. 2c) to the core only (Fig. 2d) and tail+core (Fig. 2e) data. However, there is no statistical difference between the tail and core complex conditions (Supplementary Fig. 7a; p=0.16) as well as no statistical difference between the tail and

tail+core conditions (Supplementary Fig. 7b; p =0.1). This makes it difficult to conclude that the core only interaction is more or less stable than the tail only interaction at 21 ms interaction time; the same can also be said for the comparison between the tail only and tail+core data.

• Lines 237-246, page 5. "However, at 121 ms interaction time between βarr2 and β2AR the measurements reveal that stable binding to the active receptor core requires additional tens of ms and thus occurs after tail binding (bolding added here for emphasis; Supplementary Fig. 7g)." I also find this difficult to conclude, as the data for the core only complex at 21 ms compared to the longer 121 ms interaction time is not statistically different (Supplementary Fig. 7e; p=0.10). Thus, while the calculated parameters (koff, xβ, ΔG) shown in Fig 2d suggest that the core stabilizes at the longer interaction time relative to the shorter interaction time, the parent data from which they are derived are not statistically different, thus this weakens confidence in statements about how the core stability changes with an increase in interaction time between βarr2 and β2AR.

It is intriguing that there appears to be a slight destabilization of the tail only complex at longer interaction times compared to that at the shorter time (21 ms vs. 121 ms data is statistically significant, Supplementary Fig. 7d, p=0.0079), and at this longer time (121 ms), the tail only interaction is statistically less stable than the core-only interaction (Supplementary Fig. 7g, p=0.0045). I can see why the authors want to relate these statistically different trends to comparisons made between the tail-only and core-only at the shorter interaction time, but the later are not statistically different, and in my view, there is not enough confidence to relate these findings. Thus, the statement that core binding occurs after tail binding is not convincingly supported by these data, and it seems just as likely that either tail-only or core initial binding can both express to the more stable core-tail interaction with more time. In fact, this is what the authors concluded based on the 21 ms data: "As the parameters describing the β2AR-arr2 tail-core complex range between those measured for the tail and the core complexes, our data suggest that within the first 21 ms of engagement, βarr2 can interact with either the phosphorylated C-tail or the active core of β2AR, resulting in a mixed population of βarr2 β2AR complexes." How core could follow tail at 121 ms therefore is confusing.

Clearly PIP2 is not necessary for the stabilization of the tail+core interaction. How this relates to the previously reported essential role of PIP2 for β-arrestin recruitment is not discussed and seems puzzling in light of some of the authors' previous work.

• Figure 3. The authors show in this manuscript that the core only interaction is greatly stabilized after longer interaction times in the presence of PIP2. As mentioned above, this is an intriguing result. One would assume at the same, longer interaction times, that the tail-core complex, which also involves the core, would have the same, if not greater, stabilizing effect as the core only complex, given the additional contribution of the tail in the core+tail complex. The tail+core complex, however, is far less stable than the core-only complex in the presence of PIP2 under these same conditions. The authors speculate in the discussion "that tail-core complexes do not stabilize in the presence of PIP2 as a result of the effect PIP2 has on βarr2 with the membrane alone." They go on to connect these findings to their computational simulations of β-arrestin2, and how its simultaneous interaction with PIP2 and the membrane might hinder its ability to stabilize the tail+core complex. If tail interaction occurs first a substantial fraction of the time, then these results would predict that in the presence of PIP2 a large fraction of the receptor-β-arrestin complexes would be core only. This would seem to suggest that phosphorylation of the receptor tail would not be required for β-arrestin recruitment with PIP2 present, which would be surprising. I don't know of any evidence in the literature that would be consistent with such a prediction. This is not discussed.

• Many studies (e.g. Yen et al , Nature 2018; Song et al, Structure, 2019; Mukhaleva et al, iScicnce 2024) have shown that PIP2 stabilizes the active states of several GPCRs. Song et al. provides evidence that PIP2 enhances the association of mini-Gs, which binds the receptor core, with the A2a receptor. Given these studies, it appears plausible that PIP2 stabilization of the β2AR active states might also enhance β-arrestin's ability to bind the core of the receptor. Thus, while the authors exclusively focus here on PIP2 binding to β-arrestin and use their MD simulations as support, it is quite possible that PIP2 effects are being exerted at the level of the receptor alone, or at both receptor and β-arrestin. This is not considered in the manuscript. Prior studies have shown that mutations (K233Q, R237Q, and K251Q; 3Q mutant) of a PIP2 binding site mapped in β-arrestin2 dramatically reduces its ability to immunoprecipitate with PIP2-coated agarose beads, suggesting that this site is the major PIP2 binding region in β-arrestin. The authors have also previously reported that this mutant blunts β-arrestin2 recruitment to β2AR in cells (where PIP2 is present in the plasma membrane), consistent with PIP2-blunted β-arrestin2-3Q forming a less stable interaction with β2AR, perhaps because of a less stable core interaction. If the PIP2 effect is in fact exclusively mediated by PIP2 binding to β-arrestin, use of the β-arrestin-3Q mutant in the experiments shown in Fig. 3 (in the presence of PIP2) would be expected to reduce the stability and binding events of the core interaction observed at 121 ms and would provide experimental data to support that the core stabilization observed in the presence of PIP2 is indeed related to PIP2 binding by wild type β-arrestin. Additionally, the use of the β-arrestin2-3Q PIP2 blunted mutant in the tail+core experiment could also reveal if the destabilization observed in the presence of PIP2 is related to β-arrestin2's binding of this lipid, or if some other mechanism is at play (see below).
o At 121 ms interaction time, could the less stable tail+core complex compared to the substantially more stable core only complex in the presence of PIP2 also be interpreted as the phosphorylated tail playing an autoinhibitory role by blocking the core's ability to engage the receptor? Prior studies, some of which were performed in living cells in the presence of PIP2 in the plasma membrane, have shown that C-tail truncations of GPCRs, including β2AR, leads to enhanced β-arrestin binding. Could this mechanism also explain the observation presented in this manuscript?

• Supplementary Figure 6. I am very puzzled by these results. The phosphorylated V2R tail peptide has substantially higher affinity for β-arrestin than the β2AR tail, and the text says that the peptide was provided in excess. If provided in excess, I

would expect the peptide to fully outcompete the receptor for β-arrestin engagement, but it only blocks about 61% of binding, The residual binding is in fact similar in character as the authors note, but I find the overall result surprising. If the peptide was truly provided in excess, then this implies a second population of β-arrestin that prefers to bind receptor, which would seem surprising.

• Figure 5. As best I can tell, while almost all the binding probabilities shown in the manuscript are relative to tail+core complex at 21 ms, here they are expressed as relative to membrane association in the absence of PIP2 at 121 ms. This makes it impossible to compare between data sets. I would suggest making all the data consistent as it would be highly informative to know the likelihood of β-arrestin interaction with membranes relative to the presence of receptor. How rare/common are these events? Notably, very little β-arrestin is seen at the surface of live cells in the absence of receptor activation, but this figure by normalizing to 1 makes it seem that β-arrestin is highly bound without receptor activation, and even more so with V2Rpp activation and PIP2. This is confusing, because the mean binding probability of the control sample, inactivated (ICI-stimulated) and unphosphorylated β2AR embedded, in the same lipid environment (PIP2 condition) being used for the experiments in Fig. 5 in the absence of receptor (Supplementary Fig. 8B; 121 ms interaction time), is substantially lower, at near zero (0.16 ± 0.04 %, as reported in line 233, page 5) number of events. How do the direct membrane interactions compare in a relative measure without receptor? Were these control experiments also performed in the +PIP2 condition (see below)?
o In Figure 5a, the +PIP2 + V2Rpp (21 ms) condition is shown and interpreted as β-arrestin2 interacting more strongly with PIP2 when activated by V2Rpp. To put this result in context with the rest of the figure and to strengthen the interpretation, it would help to perform the same experiment with lipids not containing PIP2, essentially a PIP2 + V2Rpp condition for comparison. Is it possible that differences in the lipid environment (charge, etc.) not due to β-arrestin binding events might be influencing these AFM-based techniques experiments? Without all the controls it is hard to compare. Comparing this with the β-arrestin-3Q mutant with blunted PIP2 binding ability in the presence of +PIP2 could also be revealing in this context.
o Fig. 5b,c; do the authors show statistical comparison of the corresponding DFS data (in rupture force (pN) vs. loading rate (pN/s) plots like for other comparisons made in the manuscript) to determine if the differences observed are statistically different?
Minor Comments

• It is perfectly reasonable to conduct these powerful biophysical experiments as done, but there is no discussion of the fact that the membranes studied here are very different from a cellular plasma membrane with asymmetry of charge on both leaflets and different lipid composition. Just a simple reminder of this in the text would be useful for context.

• How do the authors know that the non GRK5 treated sample is completely devoid of receptor tail phosphorylation and wasn't phosphorylated to some extent prior to purification.

• Why do the authors use BI here and does this impact the findings? This should be discussed as what is most physiologically relevant is the epinephrine activated receptor, which in fact was used for the GRK5 induced phosphorylation.

• Why do the authors use GRK5 and not GRK2? Is there evidence that the phosphorylation bar code might lead to differences in the findings?

• Supplementary Figure 2b. Are these representative images? What is the N of these experiments?

• Page 2, lines 130-132. "The binding rate, i.e., binding events per total number of FT curves, was 2.0%, while in control experiments on membranes without β2AR it was 0.1%, indicating that the majority of detected events correspond to the spontaneous binding of βarr2 to β2AR." Where is this data shown? There is no figure reference after this sentence. Supplementary Figure 4 is related, but I don't see data/distributions of binding events showing the binding events are 2.0% for β2AR -containing samples and 0.1% for samples prepared in the absence of receptor. Perhaps the representative images shown in Supplementary Figure 4a,b are where such distributions are derived. The authors should show these distributions and associated statistical comparisons, and how many active imaging areas and independent replications were carried out to support their claim.

• Line 175: "Taken together, the experiments show that βarr2 binds the phosphorylated C-tail of β2AR rapidly, within millisececonds." I am not sure what is meant by milliseconds here. It would seem that all we can conclude is that some binding can take place within 16 ms when the βarr2 is appropriately positioned nearby. Again, the model system is powerful and appreciated, but βarr2 is normally diffusing in the cytoplasm, so we have to remember that in these experiments it is being tethered very close to the final binding position.

Reviewer #2 (Remarks to the Author):

The research on β-arrestins, conducted by a team of world leading experts, is pioneering in its detailed exploration of how these proteins complex with G protein-coupled receptors (GPCRs) to regulate signaling. β-arrestins, particularly β-arrestin1 and β-arrestin2, play crucial roles in desensitizing and internalizing GPCRs, which are essential for cellular responsiveness. This study advances our understanding by demonstrating how β-arrestins interact with various structural elements of GPCRs—such as the C-tail and core—and how these interactions impact signaling outcomes. The use of dynamic single-molecule force spectroscopy and molecular dynamics simulations provides profound insights into the mechanical and thermodynamic properties of β-arrestin-GPCR complexes. These findings reveal that β-arrestins can adopt multiple active

conformations depending on their interaction with the GPCR, leading to different signaling pathways. Moreover, the role of the lipid environment, particularly phosphoinositides, in modulating these interactions highlights a significant layer of complexity in GPCR regulation. This groundbreaking work has significant implications for drug development, offering potential for therapies that more precisely target GPCR signaling in diseases such as cancer and diabetes. The research sets a new standard in the field, combining sophisticated experimental techniques with a deep understanding of molecular interactions to unravel the complexities of cell signaling.

The height clamp experiments measuring the single binding events between barr2 and b2AR are fascinating and extremely interesting. To my opinion they show for the first time directly and in real time the on and off binding of arrestin to a GPCR. The current analysis of the height clamp data suggests single binding and unbinding events of the barr2-b2AR complex. This suggests that the complex can bind only in one step and that further binding steps cannot occur under externally applied force. Did the authors analyze whether the binding events detected in the height clamp mode show multiple binding or/and unbinding steps? How frequently were such binding steps detected if at all? How do these steps distribute in force/time? Is there any indication of multiple binding steps within this (un-)binding events?

The authors present an impressive amount of DFS experiments to characterize the various conditions of barr2-b2AR complex formation. However the authors should cite the pioneering work of the group in which they detected ligand binding to GPCRs. They find that the complexes can form via several consecutive steps. However, do these complexes also unbind via several steps? To answer this question the authors could analyze the rupture/ dissociation events of the complexes, and whether these events occur in single or multiple steps. Such analysis may be challenging, but rewarding in contributing towards the understanding how the complexes stabilize, destabilize and restablize through rebinding.

If I understand correctly, the authors applied the Kernel-Density Estimation to determine the most probable rupture force and loading rate. It would be nice to show the fits of the individual data sets and estimate the quality/robustness of the fits.

Alternative models for experimental data analysis. Did the authors test alternative models for data analysis? If there are suitable models, do they reveal similar insight?

The authors use the Freedman-Diaconis rule to bin their data, what was the rationale for selecting this rule? Are there suitable other rules that could be applied and how would they change the outcome of the analysis?

Fig. 2f,g,h. The data points in the whisker plots, do they represent single replicates? Was each replicate recorded with a new sample preparation and functionalized cantilever? Please specify.

Fig. 3d,e,f. The data points in the whisker plots, do they represent single replicates? Was each replicate recorded with a new sample preparation and functionalized cantilever? Please specify.

Fig. 4f. Please show data points wherever possible.

Fig. 5e,f. Please show data points wherever possible.

Reviewer #3: Reviewer #3 co-reviewed with Rev#1.

Version 1:

Decision Letter:

11th Dec 2025

Dear Dr. Mueller,

Thank you again for submitting your manuscript "Roles of ligand, phosphorylation, and membrane in assembling human β2-adrenergic receptor-β-arrestin complexes" to the journal.

Please accept my apologies for the length of time the manuscript has been out to re-review. Unfortunately, some of the reviewers were delayed in submitting their re-assessment.

You will see that there is an additional reviewer on the panel. Since more MD simulations were added in revision, we found it necessary to have a specific reviewer assess technical aspects of these. I am sorry this has been done at this late stage, but we ensure you that the reviewer was aware that the manuscript had been revised, and that they focused on technical points only. We also evaluated their comments carefully, to ensure that no new scientific points were raised at this point, to ensure a fair peer-review process.

Nevertheless, we now have comments (below) from the 4 reviewers who evaluated your paper. Please note that reviewers #1 and #3 co-reviewed the submission again, and their comments are redundant. In light of those reports, we remain

interested in your study and would like to see your response to the comments of the referees in the form of a revised manuscript.

You will see that while reviewers appreciate the results, they raise several concerns which will need to be addressed in a revision. While the reviewers appreciate the findings, which have been further strengthened in revision, you will see that reviewer #1 and #3, who co-reviewed the manuscript, have some remaining points which require clarification. Please make an effort to make the manuscript accessible to a broad audience. Likewise, reviewer #2 asks for clarifications regarding contact time estimations, as well as thermodynamics – please ensure these are addressed. We also insist that you provide measures requested by reviewer #4 in the supplementary information or extended data files – RMSD and PCA plots, as well as equilibration tests. We also ask that in line with comments of reviewers #1, #3 and #4 you tone down and provide caveats to any conclusions which cannot be fully supported by MD and other data at hand.

We appreciate the efforts to strengthen the manuscript, and usually avoid multiple rounds of revision, but will appreciate your efforts in addressing the remaining minor comments in one more round of revision.

Please be sure to address/respond to all concerns of the referees in full in a point-by-point response and highlight all changes in the revised manuscript text file. We expect to see your revised manuscript within 6 weeks. We are committed to providing a fair and constructive peer-review process; please do not hesitate to contact us if you would like to discuss the reviews or anticipate any delay with the resubmission. If you cannot send it within this time, please contact us to discuss an extension; we will still consider your revision provided that no similar work has been accepted for publication at NSMB or published elsewhere.

REPORTING AND DATA AVAILABILITY: Please provide updated files as follows with your revision. As you already know, we put great emphasis on ensuring that the methods and statistics reported in our papers are correct and accurate.

1. REPORTING SUMMARY: Please provide an updated file:

https://www.nature.com/documents/nr-reporting-summary.pdf

This form is a dynamic ‘smart pdf’ and must be downloaded and completed in Adobe Reader.

2. REPORTING OF STRUCTURAL DATA: If there are additional or modified structures in the revision, please submit the corresponding PDB validation reports with your other files, and use the figshare integration system to provide access to maps, half-maps, and models.

Manuscripts reporting new structures should contain a table summarizing structural and refinement statistics. Please include these tables for new cryo-EM or -ET (https://www.nature.com/documents/nr-tables-cryo-em.doc and modifying this file for ET), NMR (https://www.nature.com/documents/nr-tables-nmr.doc) and X-ray crystallography (https://www.nature.com/documents/nr-tables-xray.doc). To facilitate assessment of the quality of the structural data, a stereo image of a portion of the electron density map (for crystallography papers) or of the superimposed lowest energy structures (>10; for NMR papers) should be provided with the submitted manuscript. If the reported structure represents a novel overall fold, a stereo image of the entire structure (as a backbone trace) should also be provided. For cryo-EM structures, a representative micrograph showing individual particles should be provided in the figures alongside a processing workflow (please see our Editorial for more information: https://www.nature.com/articles/s41594-025-01567-9).

DATA DEPOSITION: We require deposition of coordinates (and, in the case of crystal structures, structure factors) into the Protein Data Bank with the designation of immediate release upon publication (HPUB). Electron microscopy-derived density maps and coordinate data must be deposited in EMDB and released upon publication. Deposition and immediate release of NMR chemical shift assignments are requested. To avoid delays in publication, dataset accession numbers must be supplied with the revised manuscript and appropriate release dates must be indicated at the galley proof stage.

3. REPORTING OF LIGHT MICROSCOPY DATA: For any revision that includes light microscopy data, we ask our authors to please include a completed light microscopy reporting table [https://www.nature.com/documents/Light_microscopy_reporting_table.xlsx] to ensure the methods are described thoroughly. The table will be available to reviewers and

ultimately published, should the manuscript be accepted at the journal. When submitting the revised version of your manuscript, please pay close attention to our href="https://www.nature.com/nature-portfolio/editorial-policies/image-integrity">Digital Image Integrity Guidelines.

4. CODE AND COMPUTATIONAL WORK: If newly developed, unpublished code was used in this work, it must be provided with the revision and access to it must be disclosed in the Code Availability Statement in the manuscript. Please also provide the completed software submission checklist. This form is a dynamic ‘smart pdf’ and must be downloaded and

completed in Adobe Reader, instead of opening it in a web browser: https://www.nature.com/documents/nr-software-policy.pdf

If molecular dynamics (MD) simulations were performed, please refer to this Editorial and provide a completed MD simulations checklist with your revision: https://www.nature.com/articles/s42003-023-04653-0

Lastly, if it applies, please complete and upload the completed machine learning checklist with your submission for re-review: https://www.nature.com/documents/machine-learning-checklist.pdf

5. SOURCE GEL IMAGES: Unprocessed scans should be provided for all gels and western blots presented in figures and should be clearly labelled with the figure number in the file title. The source gel images should be uncropped, unmodified images, with molecular weight markers. Uncropped, unmodified gel images from existing experimental repeats, in addition to the data shown in the figures, can also be presented in source data. Please provide these files as PDF with your revision.

Please ensure that all control panels for gels and western blots are appropriately described as loading or IP controls. Lastly, please ensure that all images in the paper are checked for duplication of panels and for splicing of gel lanes.

6. SOURCE NUMERICAL DATA: we urge authors to provide, in tabular form in excel files, the data underlying the graphical representations used in figures. This is to further increase transparency in data reporting, as detailed in this editorial (http://www.nature.com/nsmb/journal/v22/n10/full/nsmb.3110.html). Please provide one excel file per figure max, with one panel per tab. When submitting files, the title field should indicate which figure the source data pertains to.

7. DATA AVAILABILITY: this journal strongly supports public availability of data. All data used in accepted papers should be available via a public data repository, or alternatively, as Supplementary Information. If data can only be shared on request, please explain why in your Data Availability Statement and in the correspondence with your editor.

For some data types, deposition in a public repository is mandatory as detailed below:

https://www.nature.com/nature-research/editorial-policies/reporting-standards#availability-of-data

Finally, please ensure that you retain unprocessed data and metadata files after publication, ideally archiving data in perpetuity, as these may be requested during the peer review and production process or after publication if any issues arise.

EXTENDED DATA FIGURES

When re-submitting your manuscript, please ensure that any supplementary figures and tables that are crucial to the manuscript’s conclusions are converted into Extended Data figures and tables to increase visibility of these data. Extended Data figures and tables are online-only (present in the online PDF and full-text HTML versions of the paper), peer-reviewed display items that provide essential background to the article but are not included in the main article due to space constraints. A maximum of ten Extended Data display items (figures and tables) is permitted.

While we encourage the use of color in preparing figures, please note that this will incur a charge to partially defray the cost of printing. Information about color charges can be found at http://www.nature.com/nsmb/authors/submit/index.html#costs

Nature Structural & Molecular Biology is committed to improving transparency in authorship. As part of our efforts in this direction, we are now requesting that all authors identified as ‘corresponding author’ on published papers create and link their Open Researcher and Contributor Identifier (ORCID) with their account on the Manuscript Tracking System (MTS), prior to acceptance. This applies to primary research papers only. ORCID helps the scientific community achieve unambiguous attribution of all scholarly contributions. You can create and link your ORCID from the home page of the MTS by clicking on ‘Modify my Springer Nature account’. For more information please visit please visit www.springernature.com/orcid.

Please use the link below to submit your revised manuscript and related files:

Link Redacted

Note: This URL links to your confidential home page and associated information about manuscripts you may have submitted, or that you are reviewing for us. If you wish to forward this email to co-authors, please delete the link to your homepage.

We look forward to seeing the revised manuscript and thank you for the opportunity to review your work.

Sincerely,

Katarzyna Ciazynska, PhD
(she/her)
Senior Editor
Nature Structural & Molecular Biology
https://orcid.org/0000-0002-9899-2428

Referee expertise:

Referee #1, #3: GPCRs, arrestins

Referee #2: AFM

Referee #4: MD simulations

Reviewers' Comments:

Reviewer #1 (Remarks to the Author):

The authors have thoroughly addressed the concerns raised in our initial review. They performed several additional experiments that strengthen the key conclusions of the manuscript and clarify several points. The revisions demonstrate a careful and rigorous approach to addressing each comment, and the manuscript is significantly improved as a result.

We have the following minor points that we believe would further enhance the clarity of the manuscript.

• Page 7, Lines 312-328. The experiments shown in Extended Data Figure 5 all appear to have been conducted in the presence of PIP2, but this is not mentioned until line 329: “However, in the presence of PIP2 the β2AR-βarr2-3Q tail-core complex…”. The late introduction of this detail, combined with the use of “however,” at line 329 initially suggested to us that lines 312-328 were describing experiments performed in the absence of PIP2. We suggest that the authors state early in the paragraph that all experiments were carried out in the presence of PIP2 to avoid this confusion.

• Page 8, Lines 363-375. The presentation of simulation results in this paragraph is difficult to follow because the text alternates between conditions (± PIP2) without clear transitions. The authors conclude: “Taken together, our simulations show that PIP2 modulates the βarr2 interaction with receptor and membrane and orients the finger loop of βarr2 towards the membrane.” Our initial read of this was that the authors were inferring from the simulations that PIP2 increases the interaction with membrane based on the simulations, which we don’t see in the data (although we might have been confused by the different numbers of simulations for the different conditions), but we think they refer to the changes in orientation and not propensity to interact. Perhaps the language in this section could be made clearer for the reader.

• Figure 5a. We know this is a lot of work and don’t propose that the authors do more experiments, but the presentation is a bit confusing to us. Why is there only 121 ms data in the absence of PIP2 along with 21 ms for V2Rpp activation – but no 21 ms data without the phosphopeptide? In contrast, there is 21 ms data for wildtype βarr2 with and without V2Rpp. This prevents comparison of 21 ms data for βarr2-3Q with wildtype βarr2 under the same conditions, although as the authors note it is similar in binding probability to wildtype βarr2 at 121 ms. Also, V2Rpp clearly increases binding probability with or without PIP2. It would be nice to know if V2Rpp can also increase binding of βarr2-3Q. This point seems particularly important given the report that activated wildtype βarr2 can stay associated with the plasma membrane in a PIP2-dependent way after dissociating from receptor– here activation increased membrane binding doesn’t seem to be PIP2 dependent, which is an important point that we don’t think is adequately discussed. We suggest the authors restructure this presentation, explicitly stating which conclusions are supported and which questions remain unresolved.

Reviewer #2 (Remarks to the Author):

I would first like to congratulate the authors for the thorough revision of the manuscript. The work is of high quality, and the improvements made in response to the previous round of review have significantly strengthened the study.
I have only two minor comments that I believe would help further clarify the methodology and interpretation:
1. Contact time estimation
The manuscript reports an interaction time of 21 ms. Based on the experimental parameters provided (ramp size of 40 nm, cantilever stiffness of 0.07 N/m, and approach/retraction speeds of 1090 nm/s and 203 nm/s), the estimated contact time is closer to ~8 ms in the absence of a dwell period. It would be helpful if the authors could clarify how the value of 21 ms was obtained, and whether an additional dwell phase or a larger effective contact distance was involved.
2. Use of the term “thermodynamic”
The manuscript refers to a thermodynamic ΔG derived from koff using an Arrhenius-type relation. However, this ΔG

corresponds to the activation energy barrier for dissociation ($\Delta G^{\ddagger}$), which is a kinetic parameter. The thermodynamic binding free energy ΔG° between the bound and unbound states depends on both koff and kon. To avoid confusion, I recommend clarifying that the quantity measured here is related to the activation barrier rather than the equilibrium free-energy difference.

Reviewer #3 (Remarks to the Author):

The authors have thoroughly addressed the concerns raised in our initial review. They performed several additional experiments that strengthen the key conclusions of the manuscript and clarify several points. The revisions demonstrate a careful and rigorous approach to addressing each comment, and the manuscript is significantly improved as a result.
We have the following minor points that we believe would further enhance the clarity of the manuscript.

•Page 7, Lines 312-328. The experiments shown in Extended Data Figure 5 all appear to have been conducted in the presence of PIP2, but this is not mentioned until line 329: “However, in the presence of PIP2 the β2AR-βarr2-3Q tail-core complex…”. The late introduction of this detail, combined with the use of “however,” at line 329 initially suggested to us that lines 312-328 were describing experiments performed in the absence of PIP2. We suggest that the authors state early in the paragraph that all experiments were carried out in the presence of PIP2 to avoid this confusion.

•Page 8, Lines 363-375. The presentation of simulation results in this paragraph is difficult to follow because the text alternates between conditions (± PIP2) without clear transitions. The authors conclude: “Taken together, our simulations show that PIP2 modulates the βarr2 interaction with receptor and membrane and orients the finger loop of βarr2 towards the membrane.” Our initial read of this was that the authors were inferring from the simulations that PIP2 increases the interaction with membrane based on the simulations, which we don’t see in the data (although we might have been confused by the different numbers of simulations for the different conditions), but we think they refer to the changes in orientation and not propensity to interact. Perhaps the language in this section could be made clearer for the reader.

•Figure 5a. We know this is a lot of work and don’t propose that the authors do more experiments, but the presentation is a bit confusing to us. Why is there only 121 ms data in the absence of PIP2 along with 21 ms for V2Rpp activation – but no 21 ms data without the phosphopeptide? In contrast, there is 21 ms data for wildtype βarr2 with and without V2Rpp. This prevents comparison of 21 ms data for βarr2-3Q with wildtype βarr2 under the same conditions, although as the authors note it is similar in binding probability to wildtype βarr2 at 121 ms. Also, V2Rpp clearly increases binding probability with or without PIP2. It would be nice to know if V2Rpp can also increase binding of βarr2-3Q. This point seems particularly important given the report that activated wildtype βarr2 can stay associated with the plasma membrane in a PIP2-dependent way after dissociating from receptor– here activation increased membrane binding doesn’t seem to be PIP2 dependent, which is an important point that we don’t think is adequately discussed. We suggest the authors restructure this presentation, explicitly stating which conclusions are supported and which questions remain unresolved.

Reviewer #4 (Remarks to the Author):

As requested by the editor, I had a closer look at the Molecular Dynamics (MD) simulations reported in this manuscript. Overall, my impression is that the simulations have been set-up carefully following established procedures, and the starting structures have been modelled as accurately as possible, given that, unfortunately, no suitable experimental structure for the complex was available. Instead, the authors used available structures of complexes with other receptors as starting structures for their MD-simulations, combined with modelling and AlphaFold for missing residues.

While this approach *might* work, it needs to be justified that the modelled complex is sufficiently accurate, i.e., similar enough to the actual (unknown) structure to study, e.g., the reported interaction energies. To this end, the authors write in the methods part: “… literature data show that atomistic MD simulations can model equilibrium structures of proteins and their complexes if their initial structures are reasonable and sufficiently equilibrated”. Taken at face value, this is a tautology of course: If the initial structures are sufficiently equilibrated, these *are* equilibrium structures. I guess what the authors actually mean is that if the modelled complex is structurally not too dissimilar from the true (unknown) structure, there is a certain chance that during sufficiently long equilibration runs, the MD structure will converge towards the true one.

Of course, there is no guarantee, and nobody knows a priori what ‘sufficiently long’ means for a particular complex. Hence, this claim needs to be supported by evidence. My main issue here is that such evidence, such as RMSD-plots, PCA plots, or cosine content analyses, are not provided. To be clear, full equilibration can never be proven, but it would be important to see if the used structures at least ‘survive’ standard tests which prove *lack* convergence.

I do acknowledge that the structural changes seen in the simulations are intriguing, nicely complement the experiments with (if properly validated) new mechanistic insights, and worth reporting. A major concern here is, however, that only interaction energies were analysed, as opposed to actual free energies. In particular, the reported interaction energies miss the (enthalpic) interactions mediated by the surrounding water molecules, and completely miss all entropic contributions to free energies. As a result, claims such as “Overall, our MD simulations show that
membranes with and without PIP2 considerably contribute to the interaction energy stabilizing 2AR- arr2 tail, core, and tail-core complexes“ or “[…] atomistic molecular dynamics (MD)
simulations, we are able to characterize the […] thermodynamic properties of the assembled 2AR- arr2 complexes” (and

similar ones) clearly are overinterpretations and need to be properly tuned down.

Anticipating the authors' reply: I know that calculating the free energies that would actually be required to support the above claims is super-difficult to impossible, but that does not justify over-interpretation or insufficient evidence. In their favour, I do agree that their reported probabilities are interesting and *can* allow to derive free energies, but this needs to be done carefully and properly, and does not relieve the authors from sufficiently carefully interpreting their interaction energies.

Finally, the conclusion “Collectively, our simulations of 2AR in membranes with and without PIP2 show that at concentrations of PIP2 expected to be present in physiological membranes, it promotes ICL3-membrane interactions without inducing active conformations of 2AR.” seems to assume that any active conformation would actually have been formed within the (still) relatively short simulation time of a few microseconds. If in reality it takes longer (the experimental upper limit seems to be milliseconds), this conclusion is not valid. Hence, the authors need to provide evidence that *in reality* the respective conformational changes actually would occur at microseconds time scales, or the conclusions needs to be tuned down.

Version 2:

Decision Letter:

Our ref: NSMB-A50618B

14th Apr 2026

Dear Dr. Mueller,

Thank you for submitting your revised manuscript "Roles of ligand, phosphorylation, and membrane in assembling human β2-adrenergic receptor-β-arrestin complexes" (NSMB-A50618B). Please accept my sincere apologies for the delay in this decision. The manuscript has now been seen by two of the original referees and their comments are below. The reviewers find that the paper has improved in revision, and therefore we'll be happy in principle to publish it in Nature Structural & Molecular Biology, pending minor revisions to satisfy the referees' final requests and to comply with our editorial and formatting guidelines.

Here, we will specifically ask that you textually address the remaining comments of reviewer #4 regarding the interpretation of the MD data.

We are now performing detailed checks on your paper and will send you a checklist detailing our editorial and formatting requirements in about a week. Please do not upload the final materials and make any revisions until you receive this additional information from us.

Thank you again for your interest in Nature Structural & Molecular Biology Please do not hesitate to contact me if you have any questions.

Sincerely,

Katarzyna Ciazynska, PhD
(she/her)
Senior Editor
Nature Structural & Molecular Biology
https://orcid.org/0000-0002-9899-2428

Reviewer #2 (Remarks to the Author):

The authors fully addressed my previous comments.
Congrats for this nice work!

Reviewer #4 (Remarks to the Author):

My main initial concerns were over-interpretations and too strong conclusions drawn from the molecular dynamics simulations that were sufficiently supported by the provided results. In their revision, the authors now provide additional data that helped me and potential readers assess the equilibration of the simulations, which I think for the mostly qualitative conclusions drawn is good enough. Further, there were a few misleading or overly confident statements (e.g., regarding the interaction energies they 'sold' as free energies), which the authors have now carefully tuned down, and I think all these issues have been sufficiently addressed now.

The only uncertainty remaining is my first comment in the previous round, i.e., whether or not the structure model is accurate enough to draw these conclusions, even if tuned down. After all, it is conceivable that the model is so far off reality that all simulations are stuck in a wrong free energy minimum. Averaging over many trajectories, as advertised in the authors' reply,

may help but does not rule out this possibility. The only way to address this issue is to try and calculate observables that can be measured independently and compare. It would be ideal if the authors could provide such a sanity-check against experiment, but I would also be ok with a clear cautionary remark to this effect when discussing their model and its limitations.

Version 3:

Decision Letter:

9th Jun 2026

Dear Dr. Mueller,

We are now happy to accept your revised paper "Factors modulating the assembly of human β2-adrenergic receptor–β-arrestin complexes" for publication as an Article in Nature Structural & Molecular Biology.

Acceptance is conditional on the manuscript's not being published elsewhere and on there being no announcement of this work to the newspapers, magazines, radio or television until the publication date in Nature Structural & Molecular Biology.

Over the next few weeks, your paper will be copyedited to ensure that it conforms to Nature Structural & Molecular Biology style. Once your paper is typeset, you will receive an email with a link to choose the appropriate publishing options for your paper and our Author Services team will be in touch regarding any additional information that may be required.

After the grant of rights is completed, you will receive a link to your electronic proof via email with a request to make any corrections within 48 hours. If, when you receive your proof, you cannot meet this deadline, please inform us at rjsproduction@springernature.com immediately.

You will not receive your proofs until the publishing agreement has been received through our system.

Due to the importance of these deadlines, we ask that you please let us know now whether you will be difficult to contact over the next month. If this is the case, we ask you provide us with the contact information (email, phone and fax) of someone who will be able to check the proofs on your behalf, and who will be available to address any last-minute problems.

To assist our authors in disseminating their research to the broader community, our SharedIt initiative provides all co-authors with the ability to generate a unique shareable link that will allow anyone (with or without a subscription) to read the published article. Recipients of the link with a subscription will also be able to download and print the PDF.

As soon as your article is published, you can generate your shareable link by entering the DOI of your article here: http://authors.springernature.com/share. Corresponding authors will also receive an automated email with the shareable link

Note the policy of the journal on data deposition: http://www.nature.com/authors/policies/availability.html.

Your paper will be published online soon after we receive proof corrections and will appear in print in the next available issue. You can find out your date of online publication by contacting the production team shortly after sending your proof corrections.

You may wish to make your media relations office aware of your accepted publication, in case they consider it appropriate to organize some internal or external publicity. Once your paper has been scheduled you will receive an email confirming the publication details. This is normally 3-4 working days in advance of publication. If you need additional notice of the date and time of publication, please let the production team know when you receive the proof of your article to ensure there is sufficient time to coordinate. Further information on our embargo policies can be found here: https://www.nature.com/authors/policies/embargo.html

You can now use a single sign-on for all your accounts, view the status of all your manuscript submissions and reviews, access usage statistics for your published articles and download a record of your refereeing activity for the Nature journals.

If you have not already done so, we strongly recommend that you upload the step-by-step protocols used in this manuscript to the Protocol Exchange. Protocol Exchange is an open online resource that allows researchers to share their detailed experimental know-how. All uploaded protocols are made freely available, assigned DOIs for ease of citation and fully searchable through nature.com. Protocols can be linked to any publications in which they are used and will be linked to from your article. You can also establish a dedicated page to collect all your lab Protocols. By uploading your Protocols to Protocol Exchange, you are enabling researchers to more readily reproduce or adapt the methodology you use, as well as increasing the visibility of your protocols and papers. Upload your Protocols at www.nature.com/protocolexchange/. Further information can be found at www.nature.com/protocolexchange/about.

An online order form for reprints of your paper is available at https://www.nature.com/reprints/author-reprints.html. Please let your coauthors and your institutions' public affairs office know that they are also welcome to order reprints by this method.

Authors may need to take specific actions to achieve compliance with funder and institutional open access mandates. If your research is supported by a funder that requires immediate open access (e.g. according to Plan S principles or the NIH public access policy) then you should select the gold OA route, and we will direct you to the compliant route where possible. Because authors warrant under our subscription licensing terms that they haven't committed to licensing any version of their article under a licence inconsistent with the terms of our agreement – including the applicable embargo period – publication under the subscription model isn't suitable for authors whose funders require no embargo.

In approximately 10 business days you will receive an email with a link to choose the appropriate publishing options for your paper and our Author Services team will be in touch regarding any additional information that may be required.

You will not receive your proofs until the publishing agreement has been received through our system.

If you have any questions about our publishing options, costs, Open Access requirements, or our legal forms, please contact ASJournals@springernature.com

Sincerely,

Katarzyna Ciazynska, PhD
(she/her)
Senior Editor
Nature Structural & Molecular Biology
https://orcid.org/0000-0002-9899-2428

Click here if you would like to recommend Nature Structural & Molecular Biology to your librarian:
http://www.nature.com/subscriptions/recommend.html#forms

Open Access This Peer Review File is licensed under a Creative Commons Attribution 4.0 International License, which permits use, sharing, adaptation, distribution and reproduction in any medium or format, as long as you give appropriate credit to the original author(s) and the source, provide a link to the Creative Commons license, and indicate if changes were made.
In cases where reviewers are anonymous, credit should be given to 'Anonymous Referee' and the source.
The images or other third party material in this Peer Review File are included in the article's Creative Commons license, unless indicated otherwise in a credit line to the material. If material is not included in the article's Creative Commons

license and your intended use is not permitted by statutory regulation or exceeds the permitted use, you will need to obtain permission directly from the copyright holder.
To view a copy of this license, visit https://creativecommons.org/licenses/by/4.0/

NSMB-A50618 “Roles of ligand, phosphorylation, and membrane in assembling human β2-adrenergic receptor-β-arrestin complexes”

Point-by-point response to the comments of Reviewers #1 and #3

Reviewer 1 and 3: “*Roles of ligand, phosphorylation, and membrane in assembling human β2-adrenergic receptor-β-arrestin complexes*” describes the use of dynamic atomic force spectroscopy (AFM)-based single molecule force spectroscopy (SMFS) and predictive atomistic molecular dynamics (MD) simulations to characterize β-arrestin2 interactions with the beta-2 adrenergic receptor (β2AR) and with membranes. The authors covalently tether purified β-arrestin2 to the tip of the AFM cantilever, which hovers over purified β2AR reconstituted in an artificial DOPC:DOPG:cholesterol lipid environment absorbed to a mica surface. This lipid environment is doped with or without phosphatidylinositol 4,5-bisphosphate (PIP2), a minor phospholipid involved in many cellular signaling pathways and also shown in prior reports to directly bind β-arrestins, regulating their interactions with receptors and ability to drive clathrin-mediated receptor endocytosis. The AFM tip is brought near the β2AR-embedded membranes, such that β-arrestin can interact with the receptor or membrane. A tip-sample interaction time of 21 ms or 121 ms is used, after which the cantilever is retracted at different speeds. From these measurements, the percentage of β-arrestin-receptor binding events is determined, and the resulting rupture force is used to extract their kinetic and thermodynamic characteristics. By strategically using full (BI-167107) and inverse (ICI-118551) β2AR agonists, the authors control whether β-arrestin2 can interact with the receptor core, as well as using β2AR in the absence or presence of GRK5-mediated phosphorylation to control receptor C-tail engagement with the phosphate sensing groove in β-arrestin2. Using these strategies, the authors are able to characterize the biophysical properties of the receptor tail only, core only, and tail+core complexes with β-arrestin2. Because β-arrestin2 has been reported to be preassociated with the plasma membrane, the authors use the same technique to quantify the dynamics of β-arrestin2 interactions with the absorbed membranes in the absence of embedded β2AR.

Authors: We thank both reviewers for their detailed and constructive review, which acknowledges the value of our AFM-based single-molecule force spectroscopy approach combined with atomistic molecular dynamics (MD) simulations for characterizing the β-arrestin2 (βarr2) interactions with the β_2-adrenergic receptor (β_2AR) and with membranes. In the following, we address the reviewers’ questions point by point and explain in detail the new data we generated based on their suggestions.

Reviewer 1 and 3: Using their approach, the authors provide further support of the existence of the receptor tail (or hanging conformation), core, and tail+core (or tight complex) complexes between β-arrestins and receptor as well as the interaction of β-arrestin with the membrane and PIP2 in the absence of receptor binding, all of which have been reported previously. What sets this work apart from the prior studies is that the authors present a deeper understanding of the kinetic and thermodynamic properties of these various complexes, as well as how these properties are affected in the absence and presence of PIP2. Arguably, the most novel and intriguing aspect of this work is the finding that PIP2 stabilizes the

receptor-β-arrestin2 core interaction, but surprisingly hinders β-arrestin2 from concomitantly binding the phosphorylated receptor C-tail. While this finding and their other experiments shed light on how PIP2 regulates the stability of the β2AR-β-arrestin2 complex, how PIP2 exerts its effects is less clear. The computational findings suggest that PIP2 binding to β-arrestin itself is primarily responsible for these effects, but this is not tested experimentally with respect to the receptor-arrestin interaction as discussed below. Furthermore, there seems to be a bit of disconnect between the conclusion here and from previous work that PIP2 is essential for β-arrestin recruitment to class A receptors such as the β2AR studied here and the fact that tail+core interactions are observed and relatively stable in the complete absence of PIP2, as well as the finding here that in the presence of PIP2 the core interaction is stable, despite the fact that tail phosphorylation seems critical for in β-arrestin recruitment in cells. These apparent disconnects are not discussed, and doing so would strengthen the manuscript and its impact.

Authors: We thank both reviewers for highlighting how our manuscript advances the current understanding of receptor-β-arrestin complex assembly. Based on the recommendations of the reviewers, we decided to directly test the role of PIP_2 in βarr2 binding to $β_2$AR and to the membrane. To this end, we conducted single-molecule force microscopy measurements with βarr2 whose phosphatidylinositol 4,5-bisphosphate (PIP_2)-binding capability was blunted (βarr2 3Q mutant; βarr2-3Q)[1]. Further, we conducted MD simulations to characterize if and how PIP_2 affects the conformation and activity of $β_2$AR, which is not complexed with βarr2. For this purpose, we performed MD simulations of the $β_2$-adrenergic receptor ($β_2$AR) embedded in membranes with and without PIP_2. Finally, we conducted single-molecule force microscopy measurements with wild-type βarr2 (βarr2-WT) and βarr2-3Q to characterize their binding to membranes without $β_2$AR, which allowed us to delineate the effect PIP_2 and arrestin activation by a receptor C-tail-mimicking phosphopeptide have on βarr2-membrane interactions. The results of the additional experiments and simulations are detailed in our response to the reviewer questions following below.

As also recommended by the reviewers, we now connect our findings to previously published key publications as detailed in our response below. In short, in our revised discussion we highlight that our results are not in contradiction to previous findings, since the experimental conditions amongst the various studies differ greatly. Furthermore, we outline why the insights gained from our biophysical studies expand the ones made in the key publications by describing more native-like conditions.

Reviewer 1 and 3: Major comments: • Page 3, lines 138-139. “*The results demonstrate that our experimental setup can detect and quantify the force, lifetime, and width of the free-energy valley from single binding events of βarr2 and β2AR*.” The authors call this technique “*AFM-based single-molecule force spectroscopy (SMFS)*,” but based on how they prepare the samples, how can the authors know that they are probing single binding events between individual β2AR and βarr2 molecules? Indeed, on line 114 the authors comment that single or groups or receptors can be seen in the membrane. It seems likely that the receptors (white spots) that are sparsely distributed and protruding from the phospholipid membrane (Supplementary Figs. 1d-g)) represent a mix of individual and multiple aggregated receptors – this would seem to explain why some of these spots appear larger in

diameter. Moreover, Supplementary Fig 2b, it appears that the bright GFP coincides with the larger spots, which would also suggest that we are seeing receptor aggregates, as the brightness seems substantially greater than the GFP for the smaller white spots. There is no discussion of how the results might be impacted by the availability of multiple receptors on the surface. Likewise, and perhaps most salient, in the schematic shown in Supplementary Fig. 3, it appears likely that multiple β-arrestin molecules could be attached to a single AFM tip. Do the authors have data suggesting that only one β-arrestin2 molecule can attach to the tip? If many β-arrestin molecules interact with a white spot representing possibly many receptors, can the force, lifetime, and width of the free-energy valleys quantified in this study be characterized as information related to a binding event between single β2AR and β-arrestin2 molecules? The sample data in Fig 1a could easily be interpreted as a relaxation to an intermediate force, followed by a second step, possibly with brief transitions to the intermediate step throughout the first phase. Clearly, as described in the methods, there is extensive filtering of the data that can only be appreciated by an expert in AFM, but these issues must be discussed.

Authors: The reviewers correctly state that Supplementary Figs. 1d-g show single as well as groups of receptors, as described in the Results, section '*Monitoring spontaneous binding events of βarr2 to $β_2AR$*'. The same applies for Supplementary Fig. 2b, in which we co-localized $β_2AR$ imaged by AFM and confocal microscopy. To facilitate this co-localization we used the fluorescently labelled nanobody Nb6B9, which binds in a one-to-one ratio to the intracellular surface of active $β_2AR$[2]. Therefore, groups of receptors light up more brightly compared to single receptors in our confocal microscopy images. While the overall concentration of $β_2AR$ is low in our proteoliposomes (Supplementary Fig. 1 and 2), detecting the interaction force between single βarr2 and $β_2AR$ has been made possible through choosing the appropriate cantilever and functionalization protocol[3]. To probe the interaction between one βarr2 covalently tethered to the cantilever and one $β_2AR$ embedded in the supported membrane bilayer, we followed the well-established steps as outlined in the following:

1) We used an AFM cantilever with a sharp tip having a radius of 8 nm (Fig. R1a, Methods). The tip radius therefore had similar size as βarr2 (measured along its longitudinal axis). The probability to chemically tether more than one βarr2 to the apex of the cantilever tip was hence reduced due to steric hindrance.
2) To chemically tether βarr2 to the cantilever tip, the cantilever was first functionalized with PEG_{27}-linkers. Subsequently, to ensure that only one or a few βarr2 molecules can covalently tether to the very end of the cantilever tip, βarr2 was covalently attached to PEG_{27}-linkers using a βarr2 concentration much below that of the PEG_{27}-linker (Supplementary Fig. 3). To this end, the concentrations of PEG_{27}-linker and βarr2 are controlled during the tip functionalization (Methods).
3) If indeed more than one βarr2 binds to the cantilever tip, the βarr2 molecules would be spatially separated due to the small radius of the tip apex. Consequently, one βarr2 molecule would be expected to attach closer to the tip apex than the other βarr2. As during our AFM measurements, we record not only force-time (FT) but also force-distance (FD) curves, the two βarr2 molecules would result in the detection of two force peaks which are separated in distance in one FD curve (Fig. R1b). It is important to note that this would be

only the case if both βarr2 molecules simultaneously bind to two β_2AR molecules, within the short interaction times tested experimentally. As outlined above, to experimentally disfavor such a scenario, we used very low concentrations of β_2AR in the membrane and very low concentrations of βarr2 attached to the tip apex. Furthermore, in our analysis we only considered force curves showing single rupture events, which represented the vast majority of force curves recorded.

For a better understanding of these points, we improved the brief summary and the data selection criteria in the Methods section '*Functionalization of cantilever tip*' of the revised manuscript.

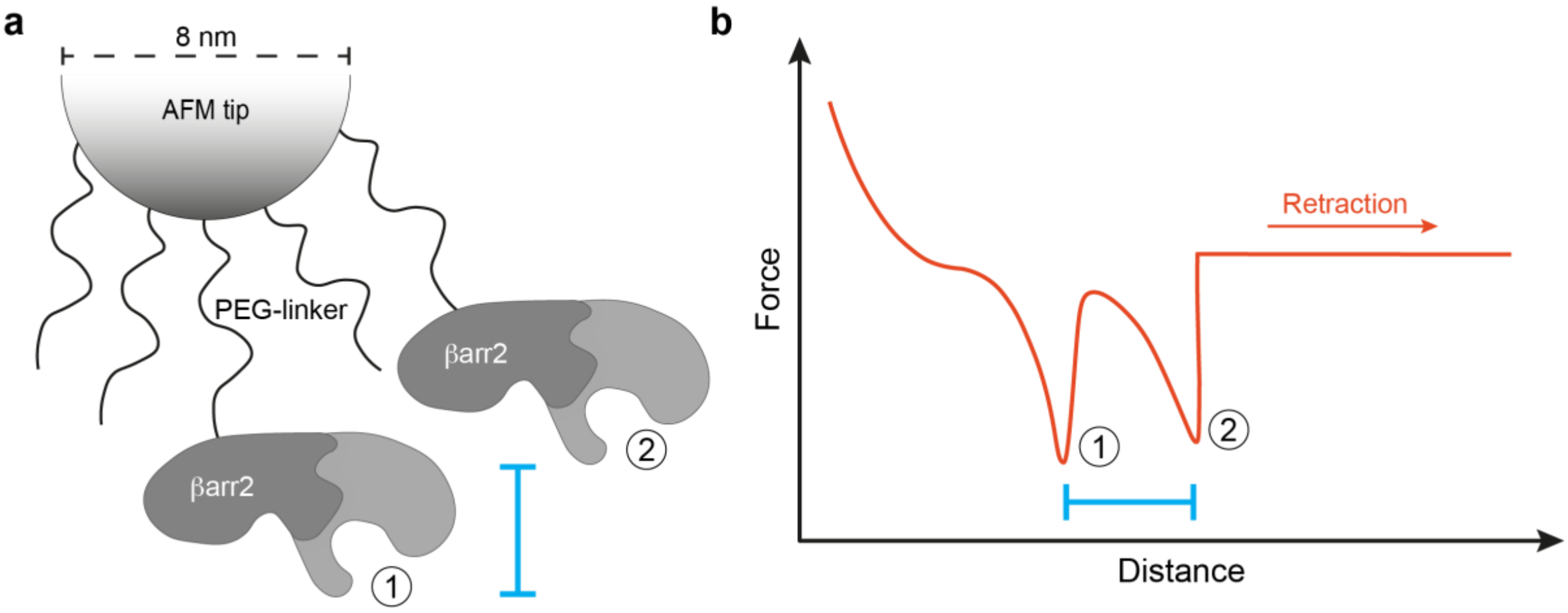

Figure R1. Underlying principle of detecting single-molecule interactions with AFM. a, Cartoon of a functionalized cantilever having a nominal tip diameter of 8 nm (Methods) to which two βarr2 molecules are covalently tethered *via* PEG_{27}-linkers (see also Fig. 1a and Supplementary Fig. 3). Due to their different attachment sites at the cantilever tip, the two βarr2 molecules are vertically separated by the distance marked in blue. **b**, Force-distance (FD) curve showing the unbinding of the two βarr2 molecules exemplified in (**a**) from a thought ligand. First, the βarr2 at the very apex of the tip (1) unbinds during the retraction of the cantilever, followed by the spatially separated second βarr2 (2), resulting in the recording of two force peaks, which are separated in distance.

The reviewers further refer to potential multiple binding steps that may occur within an unbinding event in our height clamp measurements. To answer this question, we calculated the variance of the experimentally measured (un-)binding force by considering single (un-)binding steps and multiple (un-)binding steps (Fig. R2a). If the variance of the force of multiple (un-)binding steps is smaller than the variance of only one (un-)binding step, there may indeed be multiple binding and unbinding steps within one (un-)binding event. However, we found that the difference in variance between multiple (un-)binding steps and a single (un-)binding step is larger than 10 only in 10 binding events, which is ≈ 6% of all 165 (un-)binding events detected in Fig. 1. In Fig. R2b we show the three binding events with the largest difference in variance. Despite the lower variance when considering multiple (un-)binding steps, it is difficult to distinguish any possible substep from noise or drift within an binding event. Considering also the small number of FT curves that potentially have detected multiple binding and unbinding steps, we do not find any statistically significant indication of such substeps within the (un-)binding events. As also reviewer 2 had comments on the detection of single and multiple (un-)binding events and the data analysis, we have included this analysis and controls in our revised manuscript to show that we indeed majorly detected and analyzed single (un-)binding events (see Results, section

'*Monitoring spontaneous binding events of βarr2 to β_2AR'* and new Supplementary Fig. 4).

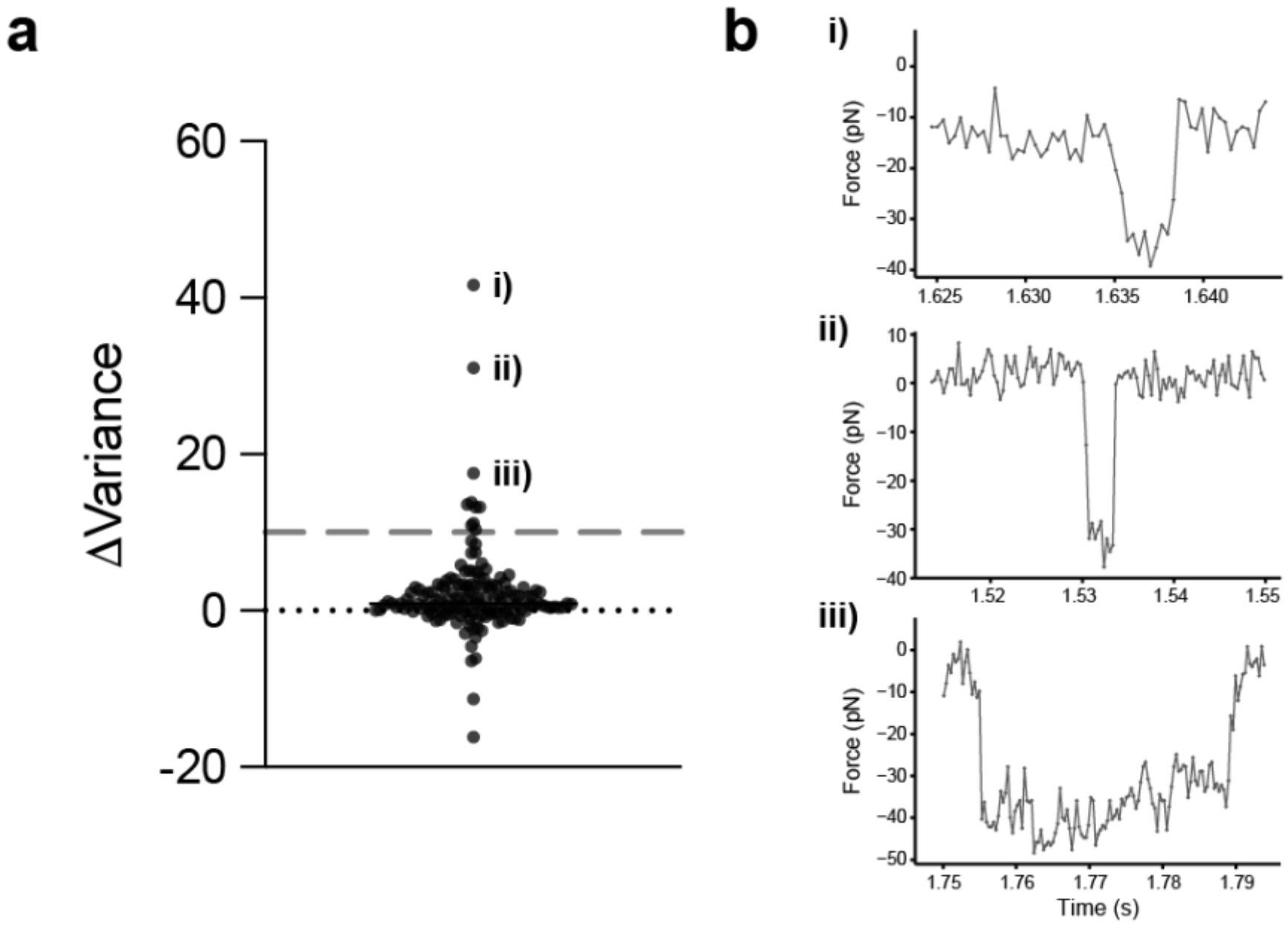

Figure R2. Analysis of multiple binding events of β$_2$AR and βarr2 and force steps in height clamp measurements. a, Difference of the variance of the force recorded in binding events of β$_2$AR and βarr2, assuming one or multiple force steps within one event. Force data were acquired using the height clamp setup described in Fig. 1. The dotted line marks no difference in variance, and the dashed line marks a difference in variance of 10. **b**, Examples of single binding events detected in force-time (FT) curves using the height clamp setup. i), ii), and iii) show single binding events with the largest difference in variance, i.e., the highest probability of multiple force steps. The data analyzed were taken from height clamp measurements presented in Fig. 1.

Reviewer 1 and 3: • Lines 237-246, page 5. "*Taken together, the results suggest that within ≈21 ms βarr2 can assemble a kinetically, mechanically, and thermodynamically stable complex with the phosphorylated C-tail of β2AR. Concurrent binding of βarr2 to the tail and core of β2AR or the core alone resulted in complexes with shorter lifetimes, narrower free-energy valleys, and lower free-energy barriers*". It appears the authors are comparing the 21 ms interaction time data for the tail only complex data (Fig. 2c) to the core only (Fig. 2d) and tail+core (Fig. 2e) data. However, there is no statistical difference between the tail and core complex conditions (Supplementary Fig. 7a; p=0.16) as well as no statistical difference between the tail and tail+core conditions (Supplementary Fig. 7b; p =0.1). This makes it difficult to conclude that the core only interaction is more or less stable than the tail only interaction at 21 ms interaction time; the same can also be said for the comparison between the tail only and tail+core data.

Authors: We apologize for having created this confusion. Before the lines cited by the reviewers we write in the Results in section '*Initial β_2AR-βarr2 complexes assemble within tens of milliseconds*' that "*Statistically, the parameters measured for tail and core complexes were not different (Supplementary Fig. 7a). Finally, we used BI-activated and phosphorylated β_2AR to characterize the β_2AR-βarr2 tail-core complex, whose values k_{off} of 6.9 ± 3.9 s^{-1}, x_β of 0.37 ± 0.05 nm, and ΔG of 9.6 ± 0.6 k_BT were considerably different from the core complexes but similar to tail complexes (Fig. 2e,*

Supplementary Fig. 7b,c).". The reviewers correctly point out that our conclusion that core and tail-core complexes are less stable than tail complexes is inaccurate. We therefore revised our conclusion in the Results section '*Fully engaged β$_2$AR-βarr2 complexes take about hundred milliseconds to assemble*' to correctly state that the kinetic and thermodynamic properties of core and tail-core complexes are similar to tail complexes at 21 ms. We have further revised the section to avoid confusion on which complexes are compared.

Reviewer 1 and 3: • Lines 237-246, page 5. "*However, at 121 ms interaction time between βarr2 and β2AR the measurements reveal that stable binding to the active receptor core requires additional tens of ms and thus occurs after tail binding (bolding added here for emphasis; Supplementary Fig. 7g)*." I also find this difficult to conclude, as the data for the core only complex at 21 ms compared to the longer 121 ms interaction time is not statistically different (Supplementary Fig. 7e; p=0.10). Thus, while the calculated parameters (koff, xβ, ΔG) shown in Fig 2d suggest that the core stabilizes at the longer interaction time relative to the shorter interaction time, the parent data from which they are derived are not statistically different, thus this weakens confidence in statements about how the core stability changes with an increase in interaction time between βarr2 and β2AR.

Authors: The reviewers correctly point out that our conclusion is not based on statistically significant differences between core complexes at 21 and 121 ms. We apologize for the mistake. Accordingly, we rephrased the Results section '*Fully engaged β$_2$AR-βarr2 complexes take about hundred milliseconds to assemble*' to state that the core complex only marginally stabilizes. In addition, we highlight that only the tail complex and the tail-core complex considerably change with increased interaction time. We have also revised the conclusion in the same section to state that the core complex only marginally changes its properties and that it is mostly the tail complex that changes with increased interaction time (see revised manuscript, Results section '*Fully engaged β$_2$AR-βarr2 complexes take about hundred milliseconds to assemble*).

Reviewer 1 and 3: It is intriguing that there appears to be a slight destabilization of the tail only complex at longer interaction times compared to that at the shorter time (21 ms vs. 121 ms data is statistically significant, Supplementary Fig. 7d, p=0.0079), and at this longer time (121 ms), the tail only interaction is statistically less stable than the core-only interaction (Supplementary Fig. 7g, p=0.0045). I can see why the authors want to relate these statistically different trends to comparisons made between the tail-only and core-only at the shorter interaction time, but the later are not statistically different, and in my view, there is not enough confidence to relate these findings. Thus, the statement that core binding occurs after tail binding is not convincingly supported by these data, and it seems just as likely that either tail-only or core initial binding can both express to the more stable core-tail interaction with more time. In fact, this is what the authors concluded based on the 21 ms data: "*As the parameters describing the β2AR-arr2 tail-core complex range between those measured for the tail and the core complexes, our data suggest that within the first 21 ms of engagement, βarr2 can interact with either the phosphorylated C-tail or the active core of β2AR, resulting in a mixed population of βarr2 β2AR complexes*." How core could follow tail at 121 ms therefore is confusing.

Authors: The reviewers raise a valid concern. In response to their comment we revised our statement regarding the sequence of events and only state that the tail complex reduces stability with increasing interaction time, which makes the tail complex less stable than the core complex. Statements regarding the sequence of events were removed.

Reviewer 1 and 3: Clearly PIP2 is not necessary for the stabilization of the tail+core interaction. How this relates to the previously reported essential role of PIP2 for β-arrestin recruitment is not discussed and seems puzzling in light of some of the authors' previous work.

Authors: We apologize for not having discussed the seeming discrepancy between our findings and previous literature. The reviewers correctly comment that in a previous publication[4] by some authors of our manuscript the data presented therein suggest that the fully engaged GPCR-arrestin tail-core complex stabilizes in the presence of PIP_2. These results were obtained with detergent-solubilized neurotensin type 1 receptor (NTSR1) and βarr1. PIP_2 was added in a soluble form. βarr1 binding to NTSR1 was measured by monitoring the change in fluorescence of the environmentally sensitive bimane fluorophore that had been introduced in the βarr1 finger loop. The key difference in the experimental setup compared to the experiments presented in our manuscript is that complex assembly of NTSR1 with βarr1 was measured in solution whereas here we measure the complex assembly of β_2AR with βarr2 with the receptor embedded in a complex phospholipid bilayer.

The data presented in our manuscript, which is further supported by additional data acquired during the revision process (see below), conclusively show that the β_2AR-βarr2 complex assembly is considerably affected by the membrane composition and the ability of βarr2 to bind phospholipids in the membrane. PIP_2 restricts the orientation of βarr2 relative to β_2AR (Fig. 4a-e) because βarr2-membrane interactions are strengthened in the presence of PIP_2 and upon activation of βarr2 (Fig. 5). As a result, the β_2AR-βarr2 tail-core complex does not stabilize (Fig. 3c,f). However, if βarr2-membrane interactions are weakened, *e.g.* due to the absence of PIP_2 or abrogation of the ability of βarr2 to bind PIP_2, the orientational freedom of βarr2 relative to β_2AR increases (Fig. 4a-e) and the β_2AR-βarr2 tail-core complex stabilizes (Fig. 2e,h). Since in our previous study[4] NTSR1 was not reconstituted into a phospholipid bilayer, βarr1 could not interact with a membrane and thus could fully engage the receptor, forming a stable NTSR1-βarr1 tail-core complex.

In our experimental approach, βarr2 is brought into close proximity of the membrane, which allows us to measure βarr2 binding to β_2AR but not the recruitment of βarr2 freely diffusing from the cytosol or of membrane pre-associated βarr2 freely diffusing to β_2AR. Hence, our observation that a stable β_2AR-βarr2 tail-core complex forms only in the absence of PIP_2 does not necessarily invalidate the finding that PIP_2 is essential for βarr2 recruitment. In fact, our βarr2-membrane interaction studies suggest that PIP_2 is necessary to stabilize βarr2 at the membrane (Fig. 5), which was previously shown to be important for the recruitment of β-arrestins to GPCRs[5] as well as for the ability of β-arrestins to translocate to clathrin-coated structures and promote receptor internalization[6]. Furthermore, the presence of PIP_2 clearly stabilizes interactions of βarr2 with the β_2AR core (Supplementary Fig. 8l,o). Together, these findings suggest

that in the cellular context βarr2-β_2AR complexes require PIP_2 to increase their lifetime to a level that is sufficient for functional responses.

Taken together, our AFM-based single-molecule force spectroscopy approach allows us to study β_2AR-βarr2 complexes at great mechanistic detail in a more native environment, compared to studies presented before. Our results and the literature discussed above, however, are not contradictory to each other. We thank the reviewers for their comments that led us to improve the Discussion of our manuscript, in which we include the important points raised above.

Reviewer 1 and 3: • Figure 3. The authors show in this manuscript that the core only interaction is greatly stabilized after longer interaction times in the presence of PIP2. As mentioned above, this is an intriguing result. One would assume at the same, longer interaction times, that the tail-core complex, which also involves the core, would have the same, if not greater, stabilizing effect as the core only complex, given the additional contribution of the tail in the core+tail complex. The tail+core complex, however, is far less stable than the core-only complex in the presence of PIP2 under these same conditions. The authors speculate in the discussion "*that tail-core complexes do not stabilize in the presence of PIP2 as a result of the effect PIP2 has on βarr2 with the membrane alone*." They go on to connect these findings to their computational simulations of β-arrestin2, and how its simultaneous interaction with PIP2 and the membrane might hinder its ability to stabilize the tail+core complex. If tail interaction occurs first a substantial fraction of the time, then these results would predict that in the presence of PIP2 a large fraction of the receptor-β-arrestin complexes would be core only. This would seem to suggest that phosphorylation of the receptor tail would not be required for β-arrestin recruitment with PIP2 present, which would be surprising. I don't know of any evidence in the literature that would be consistent with such a prediction. This is not discussed.

Authors: We thank the reviewers for their concise summary of one of our major findings but do not fully agree with the conclusion they draw from it. Since βarr2 is stabilized at the membrane in the presence of PIP_2 (Fig. 5) and stable β_2AR tail binding occurs rapidly and more frequently than core binding (as our binding probability experiments in the presence of PIP_2 suggest, Fig. 3f,g), activation of βarr2 upon β_2AR tail binding will further promote membrane association of βarr2 (see Results of V2Rpp-activated βarr2 binding to PIP_2-containing membranes, Fig. 5a). As the reviewers correctly state, we hypothesize that increased membrane association of βarr2 may be the reason that we do not observe any stabilization of the β_2AR-βarr2 tail-core complex in the presence of PIP_2 in our experimental setup. In turn, fast and frequent tail binding and subsequent stabilization of activated βarr2 at the PIP_2-containing membrane would imply that in the presence of β_2AR C-tail phosphorylation, mostly tail complexes and not core complexes as suggested by the reviewers would be formed. If β_2AR is not phosphorylated, we observe that βarr2 can still bind to the active receptor core by forming a stable β_2AR-βarr2 core complex in the presence of PIP_2 (Fig. 3e). In fact, Eichel and colleagues[6] demonstrated that tail-truncated β_2AR mutants stimulated with isoproterenol robustly recruit βarr2, which subsequently accumulate in clathrin-coated structures. This accumulation of βarr2 in clathrin-coated structures was dependent on ligand activation of β_2AR and direct binding of βarr2 to the β_2AR core. This finding goes hand-in-hand with a study by Latorraca *et al*.[7] showing that the GPCR core can

induce active conformations in arrestin in the absence of a phosphorylated receptor C-tail. A tail-truncated β2AR mutant used in the study by Eichel *et al.* was also shown to recruit βarr2 even in GPCR kinase (GRK) knock-out cells[8]. Furthermore, a study on the angiotensin II type-1 receptor (AT1R) demonstrated that βarr2 can engage the AT1R core even in the absence of tail phosphorylation[9]. Also the leukotriene B_4 receptor 1 was found to recruit β-arrestin in a phosphorylation-independent manner[10]. In a very recent publication by Gomes, Di Michele and colleagues[11] the authors suggest that PIP_2 enables full-length βarr1 to bind to the active core of the ghrelin receptor in the absence of receptor phosphorylation. Taking together these findings, there is ample evidence that receptor C-tail phosphorylation is not required for stable arrestin recruitment and activation. Our results strongly suggest that full-length βarr2 strongly binds to unphosphorylated full-length β_2AR if PIP_2 is present in the membrane, which represents an alternative pathway of βarr2 recruitment to β_2AR.

We thank both reviewers for their comments, which led us to discuss our findings and how they correlate with above mentioned literature more thoroughly in the revised Discussion of our manuscript.

Reviewer 1 and 3: • Many studies (e.g. Yen et al , Nature 2018; Song et al, Structure, 2019; Mukhaleva et al, iScicnce 2024) have shown that PIP2 stabilizes the active states of several GPCRs. Song et al. provides evidence that PIP2 enhances the association of mini-Gs, which binds the receptor core, with the A2a receptor. Given these studies, it appears plausible that PIP2 stabilization of the β2AR active states might also enhance β-arrestin's ability to bind the core of the receptor. Thus, while the authors exclusively focus here on PIP2 binding to β-arrestin and use their MD simulations as support, it is quite possible that PIP2 effects are being exerted at the level of the receptor alone, or at both receptor and β-arrestin. This is not considered in the manuscript. Prior studies have shown that mutations (K233Q, R237Q, and K251Q; 3Q mutant) of a PIP2 binding site mapped in β-arrestin2 dramatically reduces its ability to immunoprecipitate with PIP2-coated agarose beads, suggesting that this site is the major PIP2 binding region in β-arrestin. The authors have also previously reported that this mutant blunts β-arrestin2 recruitment to β2AR in cells (where PIP2 is present in the plasma membrane), consistent with PIP2-blunted β-arrestin2-3Q forming a less stable interaction with β2AR, perhaps because of a less stable core interaction. If the PIP2 effect is in fact exclusively mediated by PIP2 binding to β-arrestin, use of the β-arrestin-3Q mutant in the experiments shown in Fig. 3 (in the presence of PIP2) would be expected to reduce the stability and binding events of the core interaction observed at 121 ms and would provide experimental data to support that the core stabilization observed in the presence of PIP2 is indeed related to PIP2 binding by wild type β-arrestin. Additionally, the use of the β-arrestin2-3Q PIP2 blunted mutant in the tail+core experiment could also reveal if the destabilization observed in the presence of PIP2 is related to β-arrestin2's binding of this lipid, or if some other mechanism is at play (see below).

Authors: We thank both reviewers for addressing the possibility of PIP_2 affecting not only how βarr2 interacts with the membrane and the receptor but also how the receptor itself may be affected by the presence of this anionic lipid. We apologize for this shortcoming of our manuscript. To investigate whether we could observe activating

effects of PIP_2 on β_2AR similar to the studies mentioned by the reviewers[12-14], we conducted 4 x 28 atomistic molecular dynamics (MD) simulations, 650 ns each, of agonist-bound unphosphorylated and phosphorylated β_2AR in membranes of the same lipid composition as used in our manuscript, *i.e.* DOPC:DOPG:cholesterol and DOPC:DOPG:cholesterol:PIP_2 (Fig. R3). Indeed, the simulations show that in the presence of PIP_2 the intracellular loop 3 (ICL3) of β_2AR binds more frequently to phospholipids of the membrane (Fig. R3a). This is very much in agreement with the findings of Mukhaleva *et al.*[12] suggesting that PIP_2 specifically draws ICL3 to the membrane promoting an open conformation of the β_2AR core. As suggested by the reviewers, enhanced ICL3 interaction with PIP_2 in the membrane could indeed promote the binding of βarr2 to the core of β_2AR, which we observed in our DFS experiments (Fig. 3). To unveil the possible impact of enhanced ICL3-membrane binding on the active state of β_2AR, the distance between transmembrane helix 3 (TM3) and TM6 was measured (Fig. R3b). The analysis shows no significant differences between the distributions of TM3-TM6 distances in the four simulated conditions. As the simulation time of 650 ns should be sufficient[12] to capture the influence of ICL3-membrane binding on the TM3-TM6 distance and because the ensemble of the simulations performed is extensive (n = 28 for each condition, initiated from eight different conformations), these results suggest that under the here studied conditions, PIP_2 itself does not influence receptor opening characterized by the distance of TM3 and TM6. Yet, it is interesting to note that in our study we used 3 mol% of PIP_2, while Mukhaleva *et al.*[12] used 10 mol% PIP_2, which is far higher than the physiological concentration[15,16]. This increased PIP_2 concentration could explain the difference in the receptor activation state. We included above results in the new Results section '*PIP_2 attracts ICL3 of β_2AR to membrane but does not promote β_2AR activation*' and the Discussion of our manuscript.

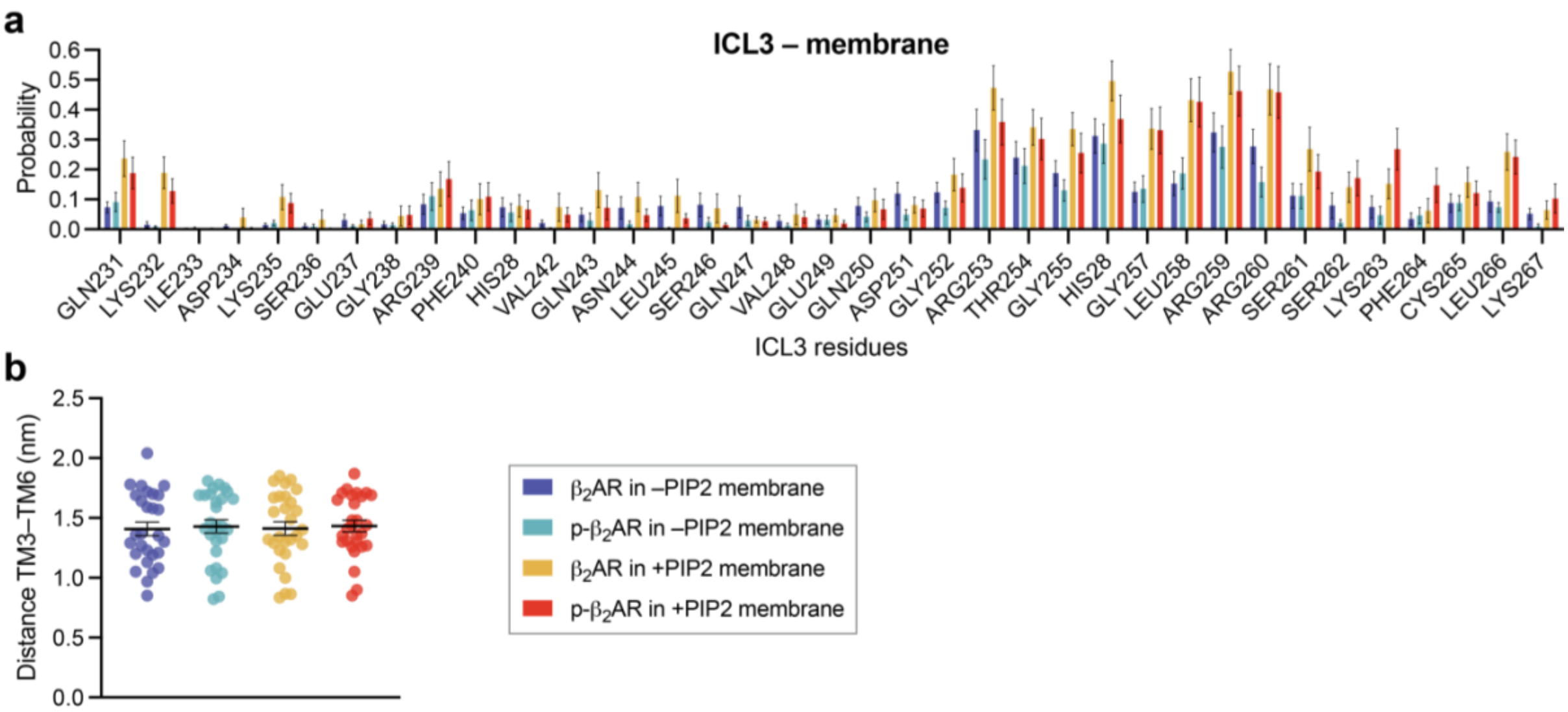

Figure R3, included in the revised manuscript as Extended Data Fig. 6. MD simulations of unphosphorylated and phosphorylated β_2AR in membranes without and with PIP_2. a, Interaction probability of β_2AR ICL3 residues with phospholipids of the membrane when β_2AR is unphosphorylated (blue) or phosphorylated (p-β_2AR; light blue) and embedded in a membrane without PIP_2 (–PIP_2) and unphosphorylated (orange) or phosphorylated (red) in a membrane with PIP_2 (+PIP_2). Bar graphs show the mean ± SEM of 28 simulations for every condition. β_2AR was equilibrated for 550 ns and the mean (interaction) probability was calculated for the following 100 ns of simulation. **b**, Distance between transmembrane helix 3 (TM3) and TM6 of β_2AR simulated in (**a**). Data points represent 28 simulations for each condition, as in (**a**). Bars indicate mean ± SEM.

The reviewers further note that mutation of three residues (K233Q, R237Q, and K251Q) on the C-lobe of βarr2 (the so-called 3Q mutant) have been shown to blunt the ability of βarr2 to bind PIP_2. They rightfully suggest to corroborate our experimental results obtained with wild-type βarr2 (βarr2-WT) with the βarr2 3Q mutant (βarr2-3Q) to test whether abrogation of the PIP_2-binding capability of βarr2 ***i)*** dampens the observed stabilization of core complexes and ***ii)*** promotes stabilization of tail-core complexes at 121 ms interaction time in membranes containing PIP_2. To address this comment, we conducted additional DFS experiments at 121 ms interaction time with βarr2-3Q tethered to the cantilever and activated, unphosphorylated β_2AR (core complex) or activated, GRK5-phosphorylated β_2AR (tail-core complex) in the presence of PIP_2 (Fig. R4a). We found that βarr2-3Q forms a complex with BI-activated, unphosphorylated β_2AR (forming a β_2AR-βarr2-3Q core complex) with a transition rate k_{off} of 1.5 ± 2.6 s^{-1}, a width of the free-energy valley x_β of 0.49 ± 0.14 nm, and a free-energy barrier ΔG of 11.1 ± 1.7 k_BT (Fig. R4b). Compared to the β_2AR-βarr2 core complex measured with βarr2-WT under the same conditions, k_{off} drastically increased and x_β decreased, correlating with a destabilisation of the β_2AR-βarr2-3Q complex (Fig. R4b). The binding probabilities did not significantly differ. Since the Bell-Evans fits of the DFS data obtained with βarr2-3Q and βarr2-WT in core complexes were not significantly different (Fig. R4c), we also compared the rupture forces of these two datasets (Fig. R4d). The rupture forces required to separate β_2AR-βarr2-3Q core complexes were for five out of six retraction speeds significantly lower than for β_2AR-βarr2-WT core complexes, suggesting that βarr2-3Q engages the core of β_2AR less stably than βarr2-WT. These findings corroborate the reviewer's hypothesis that βarr2-3Q forms a less stable core complex in the presence of PIP_2. Hence, this new set of data provides experimental proof that the stabilization of the core complex in the presence of PIP_2 depends on ability of βarr2-WT to bind PIP_2.

For the β_2AR-βarr2-3Q tail-core complex, we experimentally measured a k_{off} of 2.0 ± 0.9 s^{-1}, x_β of 0.41 ± 0.03 nm, and ΔG of 10.8 ± 0.4 k_BT (Fig. R4e). Thus, with βarr2-3Q the tail-core complex in the presence of PIP_2 increased stability compared to the tail-core complex with βarr2-WT (Fig. R4f,g). The binding probability of the β_2AR-βarr2-3Q tail-core complex again did not change significantly. Consequently, the ability of βarr2-WT to bind PIP_2 prevents the tail-core complex to stabilize if PIP_2 is in the membrane. In addition, the fact that the kinetic and thermodynamic properties of β_2AR-βarr2-3Q core and β_2AR-βarr2-3Q tail-core complexes are no longer distinguishable from each other underlines that losing the ability to bind PIP_2 considerably changes complex assembly (Fig. R4h). This finding is in stark contrast to β_2AR-βarr2-WT core and β_2AR-βarr2-WT tail-core complexes, which show clear differences from each other (Supplementary Fig. 8i).

Together with our original results, the additional experiments provide compelling evidence that PIP_2 plays a major role in modulating the complex assembly of β_2AR with βarr2 by affecting the membrane interaction of βarr2 and ICL3 of β_2AR. We thank the reviewers for their comments, which led us to conduct these additional experiments. The new experimental data have been included and discussed in our revised manuscript to further strengthen our mechanistic insights (see revised Results,

section ‘*PIP₂ promotes βarr2 binding to transmembrane core of β₂AR*’ and Discussion).

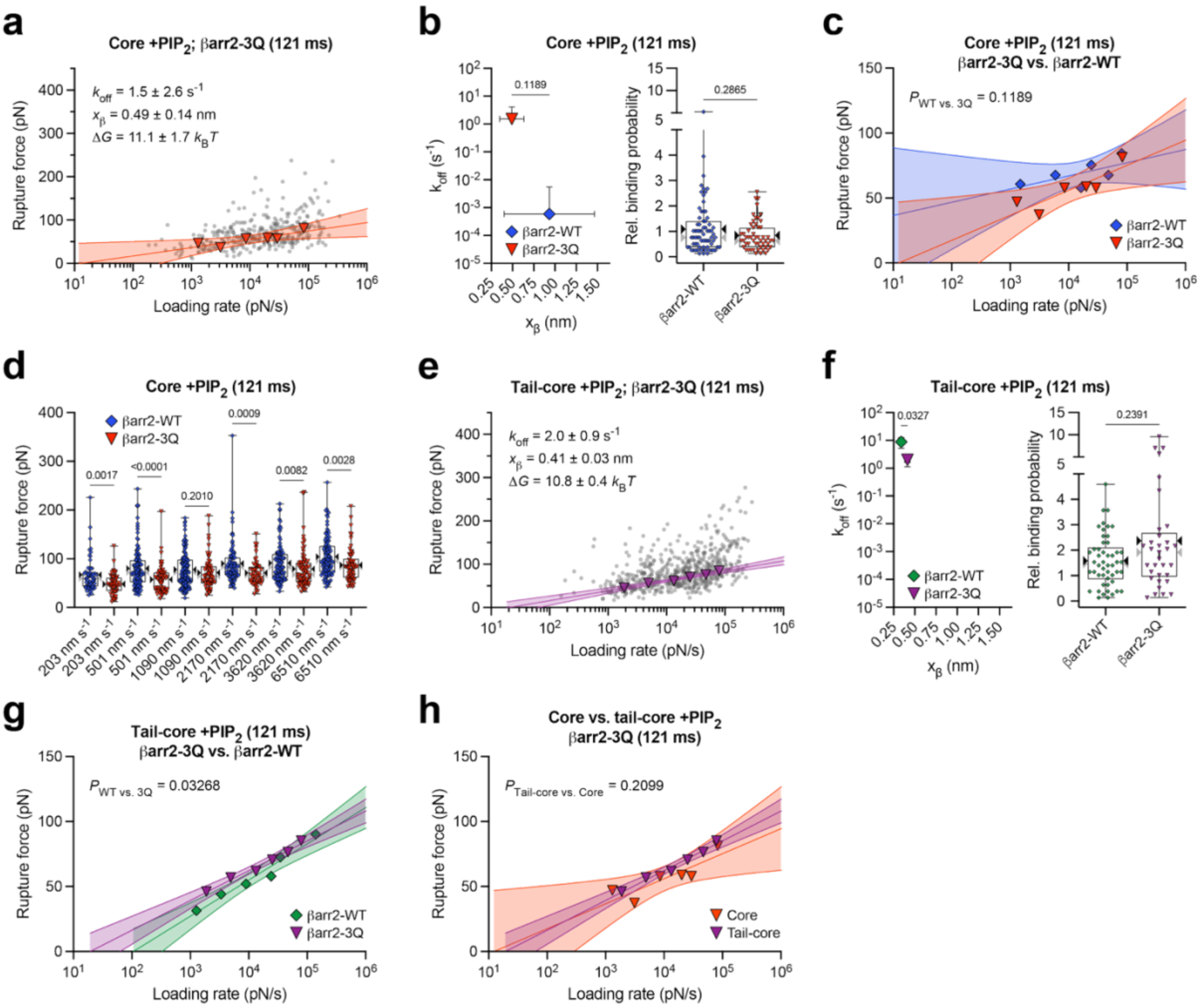

Figure R4, included in the revised manuscript as Extended Data Fig. 5. DFS measurements of core and tail-core complexes of βarr2-3Q mutant with β₂AR in membranes with PIP₂ at 121 ms interaction time. a, DFS data of β₂AR-βarr2-3Q core complexes in the presence of PIP₂ at 121 ms interaction time from 53 replicates recorded with 13 different AFM cantilevers from 4 independent functionalizations. DFS data are binned (red triangles) for each retraction speed (Methods). The Bell-Evans model[17] fit to the binned data is shown in red as mean (line) ± 95% CI (shaded area). **b**, Comparison of k_{off}, $x_β$, and binding probabilities of β₂AR-βarr2-3Q (red triangles) and β₂AR-βarr2-WT (blue diamond) core complexes at 121 ms in the presence of PIP₂. k_{off} and $x_β$ values are shown as mean ± SD. *P* values comparing k_{off} and $x_β$ values are calculated by applying the extra sum-of-squares F test to Bell-Evans fits of the DFS data shown in (**c**). Colors and symbols as in (**b**). **d**, Comparison of rupture forces recorded at different retraction speeds of β₂AR-βarr2-3Q (red triangles) and β₂AR-βarr2-WT (blue diamond) core complexes at 121 ms in the presence of PIP₂. **e**, DFS data of β₂AR-βarr2-3Q tail-core complexes in the presence of PIP₂ at 121 ms interaction time from 33 replicates recorded with 6 different AFM cantilevers from 3 independent functionalizations. DFS data are binned (purple triangles) for each retraction speed (Methods). The Bell-Evans model[17] fit to the binned data is shown in purple as mean (line) ± 95% CI (shaded area). **f**, Comparison of k_{off}, $x_β$, and binding probabilities of β₂AR-βarr2-3Q (purple triangles) and β₂AR-βarr2-WT (green diamond) tail-core complexes at 121 ms in the presence of PIP₂. k_{off} and $x_β$ values are shown as mean ± SD. *P* values comparing k_{off} and $x_β$ values are calculated by applying the extra sum-of-squares F test to Bell-Evans fits of the DFS data shown in (**g**). **g**, Comparison of Bell-Evans fits of the DFS data of β₂AR-βarr2-3Q tail-core (purple triangles) and β₂AR-βarr2-WT tail-core (green diamonds) complexes at 121 ms. *P* values are calculated by applying the extra sum-of-squares F test. **h**, Comparison of Bell-Evans fits to the DFS data of β₂AR-βarr2-3Q core (red) and tail-core (purple) complexes. *P* values are calculated by applying the extra

sum-of-squares F test. Binding probabilities and rupture forces in (**b**), (**d**), and (**f**) are shown as boxplots where boxes mark the 25th to 75th percentiles, whiskers show minimum and maximum values, and median (grey arrows) and mean (black arrows) are indicated. Binding probability data are normalized to the mean of the tail-core complex at 21 ms in the absence of PIP_2 (Fig. 2h). *P* values comparing binding probabilities and rupture forces in (**b**), (**d**), and (**f**) are obtained from Mann-Whitney tests. Data collected with βarr2-WT are taken from Fig. 3b,e and Fig. 3c,f for the core and tail-core complex, respectively.

Reviewer 1 and 3: At 121 ms interaction time, could the less stable tail+core complex compared to the substantially more stable core only complex in the presence of PIP2 also be interpreted as the phosphorylated tail playing an autoinhibitory role by blocking the core's ability to engage the receptor? Prior studies, some of which were performed in living cells in the presence of PIP2 in the plasma membrane, have shown that C-tail truncations of GPCRs, including β2AR, leads to enhanced β-arrestin binding. Could this mechanism also explain the observation presented in this manuscript?

Authors: We thank the reviewers for this interesting hypothesis. Indeed, the β_2AR C-tail has been proposed to have an autoinhibitory role affecting coupling to βarr2[8] and the Gs protein[18]. To address this possibility, we conducted MD simulations of agonist-bound unphosphorylated and phosphorylated β_2AR in membranes without and with PIP_2. We tracked the interaction of the β_2AR C-tail with elements of β_2AR and the membrane (Fig. R5). First, by visual inspection of the simulations, we observed that under all four conditions the receptor core can be open, partially open, or occluded (Fig. R5a). Next, we analyzed the behaviour of the C-tail in more detail (Fig. R5b-e). We calculated the probability of a free C-tail, *i.e.* no interaction with phospholipids of the membrane or other β_2AR residues and found no difference between phosphorylated and unphosphorylated β_2AR in membranes without and with PIP_2 (Fig. R5b). Moreover, there were no marked differences with respect to the interaction probability of the C-tail with phospholipids of the membrane (Fig. R5c). Interestingly, C-tail interaction with ICL2 became substantially more probable if β_2AR was phosphorylated and PIP_2 was present (Fig. R5d). The interaction probability increased by 10–20% for 10 C-tail residues. Although the increased ICL2 binding by the C-tail can occlude the β_2AR core, making it less accessible to βarr2, the probability of this binding is low. C-tail–ICL3 interactions appeared to be slightly increased if the C-tail was phosphorylated but were not altered by the presence of PIP_2 (Fig. R5e).

Taken together, the new MD simulations provide some evidence for a model in which the β_2AR C-tail is more prone to occlude the β_2AR core when phosphorylated and PIP_2 is present. Such an autoinhibitory role of the phosphorylated β_2AR C-tail could contribute to the lack of stabilization of β_2AR-βarr2 tail-core complexes in the presence of PIP_2. Moreover, our DFS data show that ***i)*** there is no reduction in binding probability for the β_2AR-βarr2 tail-core complex and ***ii)*** the β_2AR-βarr2 tail-core complex is indistinguishable from the β_2AR-βarr2 tail complex. Thus, if β_2AR is phosphorylated, βarr2 can still bind to the receptor C-tail. Furthermore, if occlusion of the receptor core were to prohibit tail-core strengthening, we would expect C-tail binding by βarr2 to make the core more accessible. In that case, stabilization of the β_2AR-βarr2 tail-core complex might take longer to occur, but even at 521 ms interaction time we do not observe any stabilization (Extended Data Fig. 4). Thus, since the β_2AR C-tail is accessible for a tail complex with βarr2, we conclude that the major factor preventing

the β_2AR-βarr2 tail-core complex from stabilizing remains the strengthening of βarr2-membrane interactions in the presence of PIP_2 and upon activation of βarr2. We thank the reviewers for their comment that has led us to conduct the additional simulations and data analysis. We have included the above results in the revised manuscript (see Results, section '*PIP_2 attracts ICL3 of β_2AR to membrane but does not promote β_2AR activation*', new Extended Data Fig. 6, and Discussion).

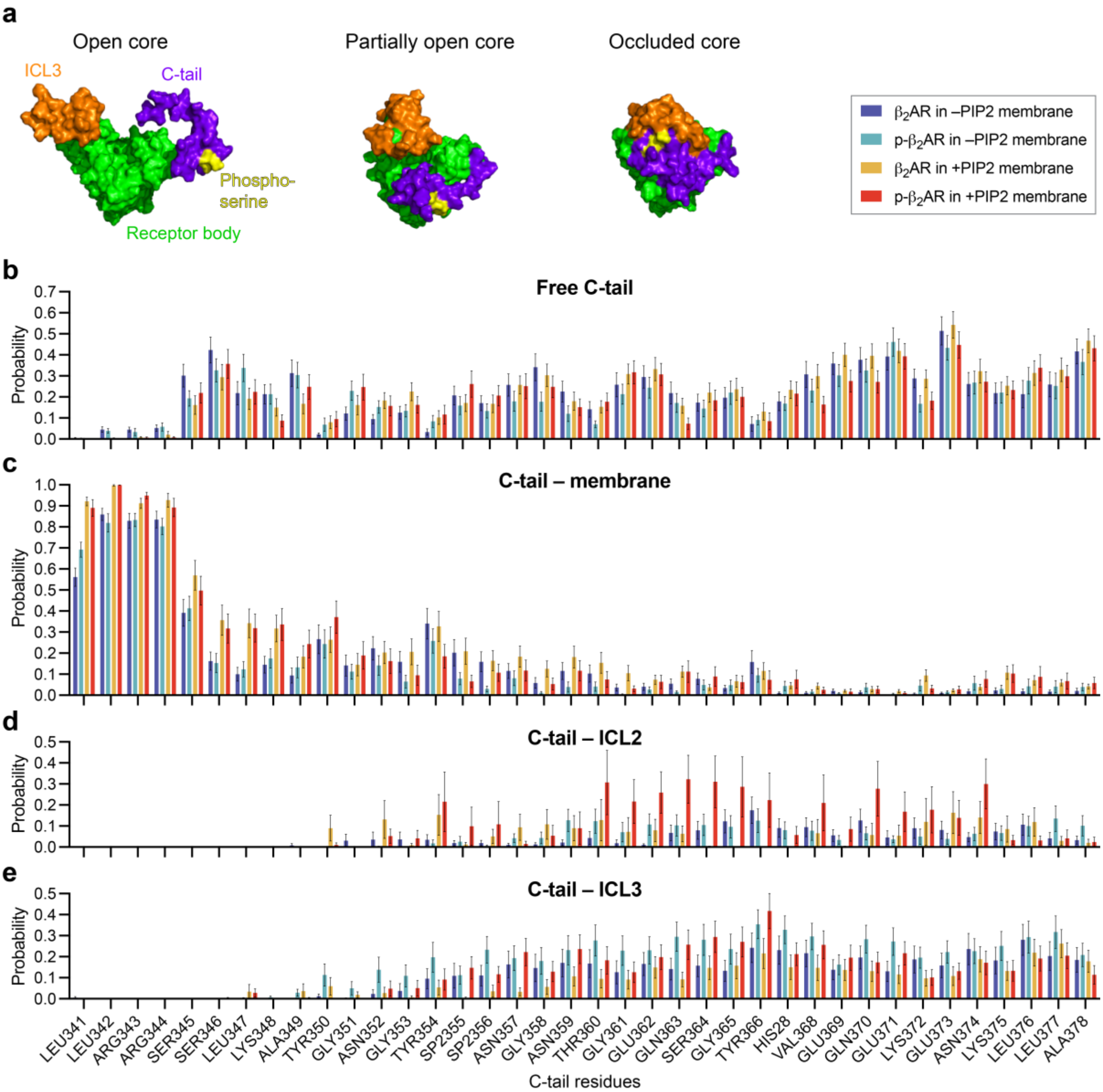

Figure R5, included in the revised manuscript as new Extended Data Fig. 6. Accessibility of the β_2AR core and interactions of the β_2AR C-tail in MD simulations of the activated, unphosphorylated and phosphorylated β_2AR in membranes without and with PIP_2. a, Molecular surfaces exemplifying β_2AR with an open, partially open, and occluded core. Receptor body coloured green, ICL3 orange, C-tail purple, and phosphoserines on C-tail yellow. **b**, Probability of β_2AR C-tail residues being free, *i.e.*, not interacting with any lipids or other receptor residues. Zero probability indicates that a residue is always interacting with lipids or other receptor residues. **c-e**, Interaction probability of (**c**) β_2AR C-tail residues with phospholipids of the membrane, (**d**) β_2AR C-tail residues with the intracellular loop 2 (ICL2), and (**e**) β_2AR C-tail residues with ICL3. Bar graphs in (**b-e**) show the mean ± SEM of 28 simulations for every condition. β_2AR was equilibrated for 550 ns and the mean (interaction) probability was calculated for the following 100 ns of simulation. Results of β_2AR embedded in a membrane without PIP_2 (–PIP_2) are shown in dark blue for unphosphorylated β_2AR and in light blue

for phosphorylated β_2AR (p-β_2AR). For β_2AR embedded in a membrane with PIP_2 (+PIP_2), results are shown in orange for unphosphorylated β_2AR and red for p-β_2AR.

Reviewer 1 and 3: •Supplementary Figure 6. I am very puzzled by these results. The phosphorylated V2R tail peptide has substantially higher affinity for β-arrestin than the β2AR tail, and the text says that the peptide was provided in excess. If provided in excess, I would expect the peptide to fully outcompete the receptor for β-arrestin engagement, but it only blocks about 61% of binding, The residual binding is in fact similar in character as the authors note, but I find the overall result surprising. If the peptide was truly provided in excess, then this implies a second population of β-arrestin that prefers to bind receptor, which would seem surprising.

Authors: We apologize for the confusion. We expanded the experimental characterization of βarr2 on phospholipid membranes without β_2AR (empty liposomes), as suggested by the reviewers. In the context of the competition binding assay shown in Supplementary Fig. 6 (now Extended Data Fig. 1), our binding probability measurements of V2Rpp-activated βarr2 on phospholipid membranes without PIP_2 shed light on the nature of the 40% of interactions that remained after competition with V2Rpp (Fig. R6). The β_2AR-βarr2 tail complex in the absence of PIP_2 at 21 ms interaction time shows a binding probability of 0.43 ± 0.06 (mean ± SEM; relative to tail-core –PIP_2, 21 ms; Fig. 2f). In the competition binding assay in Extended Data Fig. 1, we inhibited the formation of the β_2AR-βarr2 tail complex by blocking the β_2AR C-tail binding site of βarr2 through addition of V2Rpp. As correctly described by the reviewers, V2Rpp blocks ≈ 60% of binding. Our binding probability measurements show that βarr2 has a binding probability to the lipid membrane without any receptor at 21 ms interaction time of 0.17 ± 0.03 (relative to β_2AR-βarr2 tail-core –PIP_2, 21 ms) in the presence of the same V2Rpp concentration as we used for the competition binding assay (Fig. R6). This binding to the lipid membrane amounts to ≈ 40% of the binding probability of above-mentioned β_2AR-βarr2 tail complex. Hence, the V2Rpp concentration we used was sufficient to prevent βarr2 from binding to the phosphorylated C-tail of β_2AR, but also increased βarr2 binding to the membrane surrounding the receptors. Thanks to the additional binding probability data (Fig. R6), we can now clearly attribute the remaining ≈ 40% of binding in the competition assay of Extended Data Fig. 1 to βarr2-membrane interactions. We have revised the manuscript and included the new experimental data to avoid confusion of the reader (see Results, section '*βarr2 binds the phosphorylated C-tail of β_2AR in milliseconds*' and '*PIP_2 binding and βarr2 activation stabilizes βarr2 on membranes*').

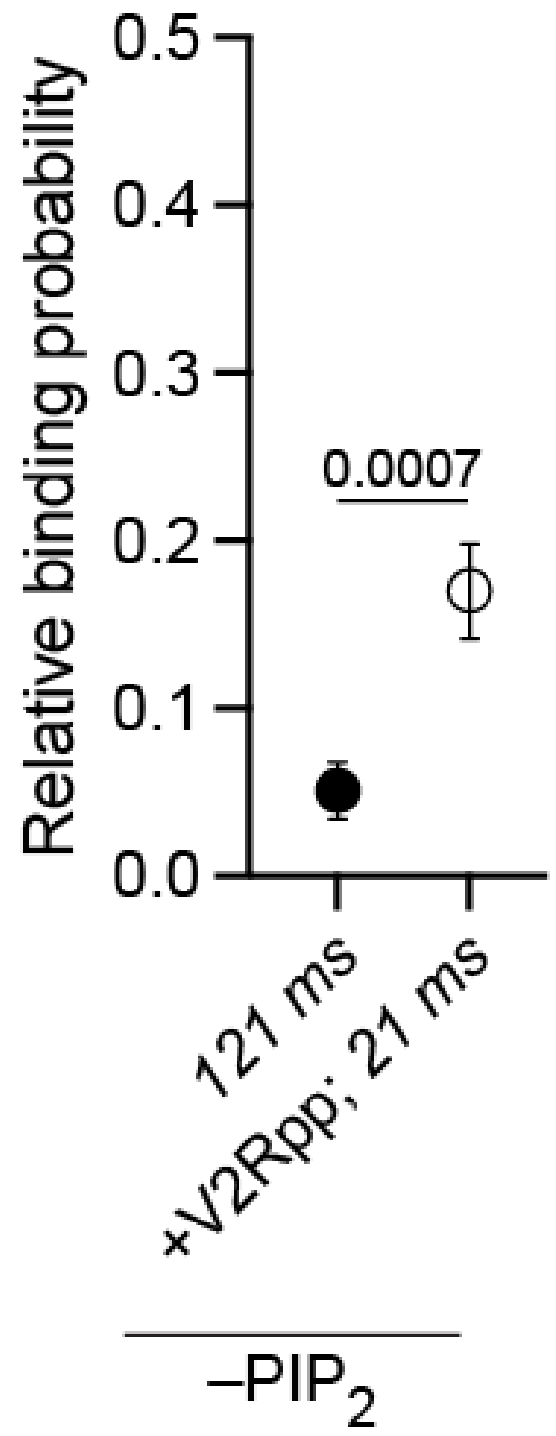

Figure R6, excerpt of Fig. R7 and included in the revised manuscript as part of revised Fig. 5a. Binding probability of βarr2–membrane interactions measured with DFS. Binding probability as measured using our AFM-based single-molecule force spectroscopy setup of βarr2 wild-type (βarr2-WT) with and without the activating phosphopeptide mimicking the phosphorylated C-tail of the vasopressin 2 receptor (V2Rpp) on membranes without PIP_2 (–PIP_2). Shown are the binding probability of βarr2-WT at 121 ms interaction time without V2Rpp (black circle; 40 replicates with 14 cantilevers from 3 independent functionalizations) and with V2Rpp (empty circle; 66 replicates, 22 cantilevers, 3 independent functionalizations) at 21 ms. Binding probabilities, which are given as mean ± SEM, have been normalized to the binding probability of the $β_2$AR-βarr2 tail-core complex (–PIP_2) at 21 ms (Fig. 2h). *P* values are obtained from Mann-Whitney tests. Data without V2Rpp are taken from Fig. 5a.

Reviewer 1 and 3: • Figure 5. As best I can tell, while almost all the binding probabilities shown in the manuscript are relative to tail+core complex at 21 ms, here they are expressed as relative to membrane association in the absence of PIP2 at 121 ms. This makes it impossible to compare between data sets. I would suggest making all the data consistent as it would be highly informative to know the likelihood of β-arrestin interaction with membranes relative to the presence of receptor. How rare/common are these events? Notably, very little β-arrestin is seen at the surface of live cells in the absence of receptor activation, but this figure by normalizing to 1 makes it seem that β-arrestin is highly bound without receptor activation, and even more so with V2Rpp activation and PIP2. This is confusing, because the mean binding probability of the control sample, inactivated (ICI-stimulated) and unphosphorylated β2AR embedded, in the same lipid environment (–PIP2 condition) being used for the experiments in Fig. 5 in the absence of receptor (Supplementary Fig. 8B; 121 ms interaction time), is substantially lower, at near zero (0.16 ± 0.04 %, as reported in line 233, page 5) number of events. How do the direct membrane interactions compare in a relative measure without receptor? Were these control experiments also performed in the +PIP2 condition (see below)?

Authors: The reviewer correctly points out that while all binding probabilities are given relative to the $β_2$AR-βarr2 tail-core complex –PIP_2 (21 ms), in Fig. 5a they are given relative to empty phospholipid membranes –PIP_2 (121 ms). We apologize for this

inconsistency. We revised Fig. 5a so that binding probabilities are now given relative to β_2AR-βarr2 tail-core complex –PIP_2 (21 ms) and thus comparable among all datasets (Fig. R7). The binding probabilities of βarr2 on phospholipid membranes without β_2AR are well below the binding probabilities of βarr2 with β_2AR. The two β_2AR-βarr2 complexes with the lowest binding probabilities are the tail complex –PIP_2 at 21 ms with a relative binding probability of 0.43 ± 0.06 (mean ± SEM) and the core complex –PIP_2 at 21 ms with a relative binding probability of 0.46 ± 0.04. In comparison, the binding probability of βarr2 on lipid membranes –PIP_2 without β_2AR and V2Rpp (black circle in Fig. R7) is 0.05 ± 0.02, corresponding to an almost 90% reduction in binding when β_2AR is absent. Also, in the presence of PIP_2 the binding probability of βarr2 to lipid membranes without β_2AR is 0.14 ± 0.03 compared to the binding probability of 0.47 ± 0.03 of the β_2AR-βarr2 core complex at 21 ms. The marked reduction in binding probability corresponds well with the reviewer's statement that in live cells only very few βarr2 molecules are observed at the plasma membrane when GPCRs are not activated. Our data are thus in good agreement with such observations and suggest that inactive βarr2 only rarely binds to the membrane. However, activation of βarr2 clearly increases the probability of membrane association. We have revised our manuscript to more clearly describe this context (see revised Results, section '*PIP_2 binding and βarr2 activation stabilizes βarr2 on membranes'* and/or Discussion).

The reviewer also refers to the control measurements of the binding probability of βarr2 to inactivated, unphosphorylated β_2AR in lipid membranes without PIP_2, which are presented in Extended Data Fig. 2. In these measurements, the binding probability of 0.16 ± 0.04 at 121 ms was also substantially reduced compared to conditions in which β_2AR can assemble a complex with βarr2. Compared to lipid membranes without any β_2AR measured under the same conditions, which show a binding probability of 0.051 ± 0.02, the binding probability slightly elevated. While we cannot fully explain this difference, one factor could be a slight increase in unspecific binding (as mentioned in the Results, section '*Fully engaged β_2AR-βarr2 complexes take about hundred milliseconds to assemble*'). While the measurements of the binding probability of βarr2 without β_2AR were carried out in membranes with and without PIP_2, the measurements with inactivated, unphosphorylated β_2AR were only done without PIP_2.

Please note that the other conditions shown in Fig. R7 are discussed in the next question of the reviewers.

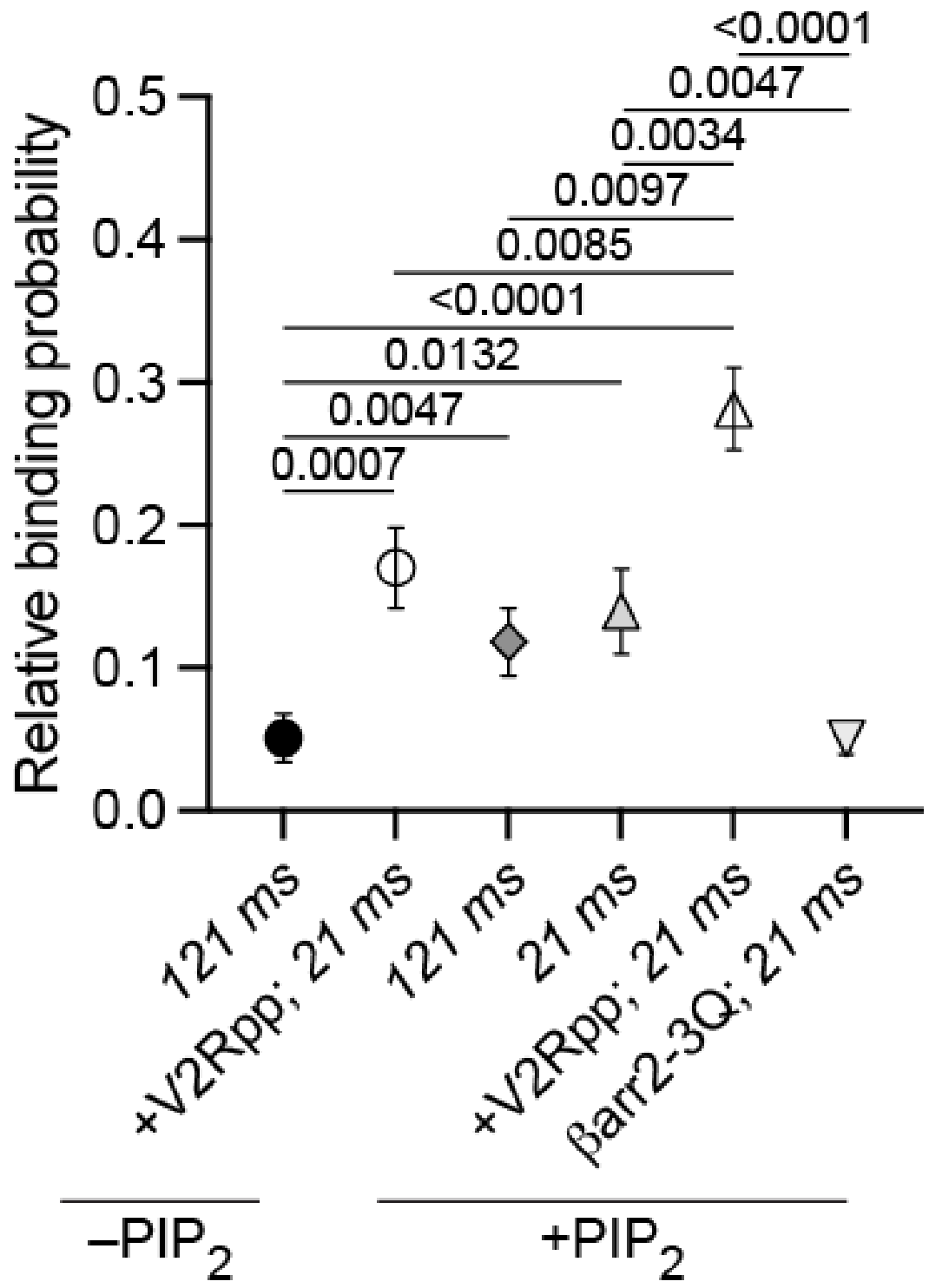

Figure R7, included in the revised manuscript as revised Fig. 5a. Binding probabilities of βarr2–membrane interactions measured with DFS. Binding probabilities as measured using our single-molecule force spectroscopy setup of βarr2 wild-type (βarr2-WT) with and without the activating phosphopeptide mimicking the phosphorylated C-tail of the vasopressin 2 receptor (V2Rpp) and of βarr2 3Q mutant (βarr2-3Q) without V2Rpp. Binding probability of βarr2-WT with lipid membranes without PIP_2 ($–PIP_2$) at 121 ms interaction time without V2Rpp (black circle; 40 replicates with 14 cantilevers from 3 independent functionalizations) and with V2Rpp at 21 ms (empty circle; 66 replicates, 22 cantilevers, 3 independent functionalizations); binding probability of βarr2-WT with lipid membranes with PIP_2 ($+PIP_2$) at 121 ms (diamond; 28 replicates, 8 cantilevers, 4 independent functionalizations) and 21 ms (grey up-triangle; 43 replicates, 13 cantilevers, 4 independent functionalizations) without V2Rpp and at 21 ms with V2Rpp (empty up-triangle; 33 replicates, 9 cantilevers, 4 independent functionalizations); binding probability of βarr2-3Q with lipid membranes $+PIP_2$ at 21 ms (down-triangle; 73 replicates, 24 cantilevers, 3 independent functionalizations). Binding probabilities are shown as mean ± SEM and are normalized to the binding probability of the β_2AR-βarr2 tail-core complex ($–PIP_2$) at 21 ms (Fig. 2h). *P* values are obtained from Mann-Whitney tests.

Reviewer 1 and 3: • In Figure 5a, the +PIP2 + V2Rpp (21 ms) condition is shown and interpreted as β-arrestin2 interacting more strongly with PIP2 when activated by V2Rpp. To put this result in context with the rest of the figure and to strengthen the interpretation, it would help to perform the same experiment with lipids not containing PIP2, essentially a –PIP2 + V2Rpp condition for comparison. Is it possible that differences in the lipid environment (charge, etc.) not due to β-arrestin binding events might be influencing these AFM-based techniques experiments? Without all the controls it is hard to compare. Comparing this with the β-arrestin-3Q mutant with blunted PIP2 binding ability in the presence of +PIP2 could also be revealing in this context.

Authors: As suggested by the reviewer, we conducted new binding probability measurements with βarr2-WT activated by V2Rpp on membranes without PIP_2 and with βarr2-3Q on membranes with PIP_2, which were included to Fig. 5 (see also Fig. R7), to strengthen our conclusions. The complete set of binding probability measurements probing βarr2-membrane interactions with βarr2-WT in an inactive and

active state, both in the absence and presence of PIP_2 now corroborates our MD simulations of the same conditions (Fig. 5b). We find that both activation of βarr2-WT as well as the presence of PIP_2 increase and strengthen βarr2-membrane binding independently. While activation alone leads to a ≈ 3-fold increase in membrane binding, addition of PIP_2 to the membrane increases membrane binding ≈ 2-fold (Fig. R7). Combining both βarr2-WT activation and addition of PIP_2 further potentiates membrane binding of βarr2. Probing the membrane binding ability of the well-characterized βarr2-3Q mutant[1] in the presence of PIP_2 reveals that βarr2-3Q behaves like βarr2-WT in the absence of PIP_2. Thus, βarr2-3Q is unable to sense PIP_2 and consequently cannot stabilize its interaction with the membrane.

Since our MD simulations show the same trend as our experimental data, and our binding probability assay shows that the βarr2-3Q mutant is insensitive to PIP_2 in membranes without β_2AR, we are confident that our results are not affected by the experimental setup of the AFM approach. We have included this information in the revised manuscript, section '*PIP2 binding and βarr2 activation stabilizes βarr2 on membranes*'.

Reviewer 1 and 3: • Fig. 5b,c; do the authors show statistical comparison of the corresponding DFS data (in rupture force (pN) vs. loading rate (pN/s) plots like for other comparisons made in the manuscript) to determine if the differences observed are statistically different?

Authors: The results of the additional binding probability measurements on lipid membranes without β_2AR (Fig. 5 and Fig. R7) further strengthen our findings that both the presence of PIP_2 in the membrane as well as βarr2 activation independently and synergistically promote βarr2-membrane association. Furthermore, as the binding probabilities of βarr2 on lipid membranes without β_2AR are extremely low (Fig. 5a), it is difficult to statistically compare their thermodynamic and kinetic parameters. Therefore, we focused our conclusions on the expanded binding probability data (Fig. 5a), included the new βarr2 +V2Rpp +PIP_2 condition (new Supplementary Fig. 10), and show the respective transition rates and heights of the free-energy barrier separating the bound from the unbound state in our summarizing model (Fig. 6).

Reviewer 1 and 3: • It is perfectly reasonable to conduct these powerful biophysical experiments as done, but there is no discussion of the fact that the membranes studied here are very different from a cellular plasma membrane with asymmetry of charge on both leaflets and different lipid composition. Just a simple reminder of this in the text would be useful for context.

Authors: We agree and included a reminder of the reduced complexity of our artificial membranes in the revised manuscript (see Results, section '*Monitoring spontaneous binding events of βarr2 to β2AR*').

Reviewer 1 and 3: • How do the authors know that the non GRK5 treated sample is completely devoid of receptor tail phosphorylation and wasn't phosphorylated to some extent prior to purification.

Authors: This is a valid concern that we addressed by introducing an additional control in which we pre-treated unphosphorylated β2AR with lambda protein phosphatase (λPP) (Fig. R8). LPP treatment was used to remove any basal phosphorylation that could have been introduced during receptor expression in insect cells (see Methods). We then performed dynamic force spectroscopy (DFS) on λPP-treated, BI-activated β2AR in the presence of PIP_2 at 21 ms interaction time, equivalent to the core +PIP_2 condition shown in Fig. 3b, top graph. We found that the kinetic, thermodynamic, and mechanical parameters as well as the binding probability are indistinguishable from β2AR-βarr2 core complexes measured in the presence of PIP_2 at 21 ms. Thus, we can confirm that any basal phosphorylation that may have been present after expression, purification, and reconstitution of β2AR does not affect our core complex measurements and that tail phosphorylations are absent in our unphosphorylated sample. We have included this control as Extended Data Fig. 3 in the revised manuscript (see Results, section '*PIP_2 promotes βarr2 binding to transmembrane core of β2AR*' and Methods, section '*Sample preparation for SMFS*').

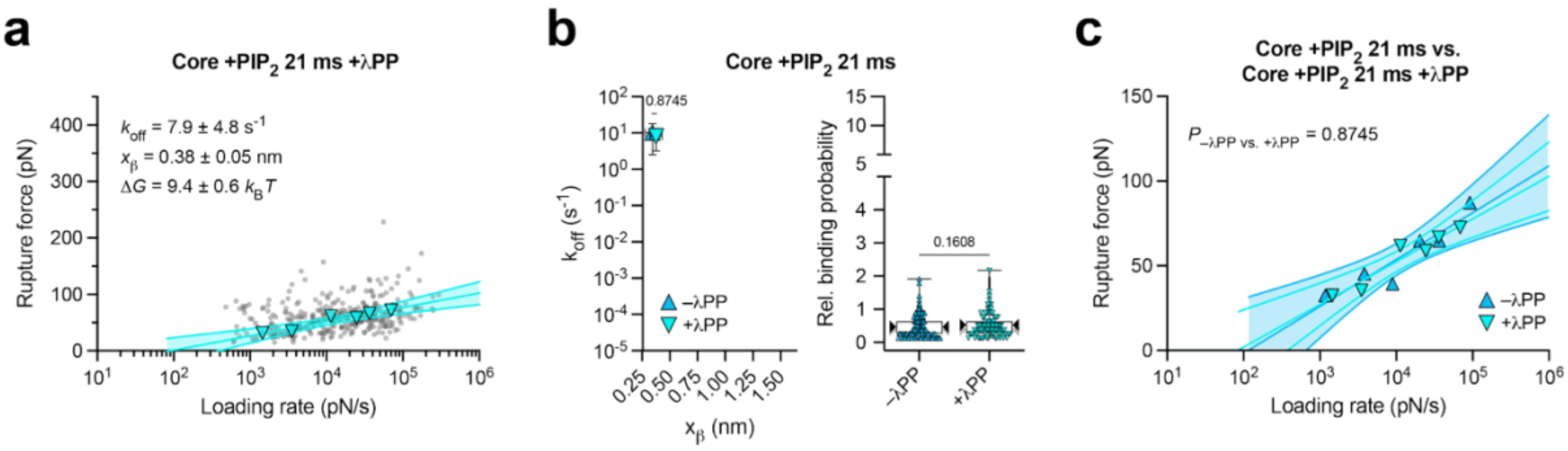

Figure R8, included in the revised manuscript as Extended Data Fig. 3. Lambda protein phosphatase-treatment of β2AR does not affect binding of βarr2 to the β2AR core. a, DFS data characterizing the binding of βarr2 to lambda protein phosphatase (λPP)-treated, BI-activated β2AR at ≈ 21 ms interaction time in the presence of PIP_2. Single (un-)binding events are shown as grey dots. DFS data are binned (down-triangles) for each retraction speed with the most probable loading rate and rupture force obtained by kernel density estimation (Methods). The Bell-Evans model[2] fit to the binned data is shown in cyan as mean (line) ± 95% CI (shaded area). DFS data represent 87 replicates obtained with 18 different cantilevers from 3 independent functionalizations. k_{off}, x_{β}, and ΔG values are given as mean ± SD. **b**, Comparison of k_{off} values, x_{β} values, and binding probabilities from β2AR-βarr2 core complexes measured in the presence of PIP_2 at ≈ 21 ms with λPP-treated (cyan down-triangle) and untreated (light-blue up-triangle) β2AR. Data is shown as mean with error bars showing SD. *P* values comparing k_{off} and x_{β} values are calculated by applying the extra sum-of-squares F test to Bell-Evans fit to the DFS data (shown in **c**). Binding probabilities are shown as boxplots where boxes mark the 25th to 75th percentiles, whiskers show minimum and maximum values, and median and mean are indicated by grey and black arrows, respectively. Data points represent the probability of βarr2 binding to the β2AR in every replicate. Binding probability data are normalized to the mean of the tail-core complex at ≈ 21 ms interaction time in the absence of PIP_2. *P* values comparing binding probabilities are obtained from Mann-Whitney tests. **c**, Statistical comparison of DFS data obtained from core complexes ± LPP in the presence of PIP2 at ≈ 21 ms. Coloring and symbols as in **b**. *P* values comparing the two conditions are calculated by applying the extra sum-of-squares F test to the Bell-Evans fit, shown as lines ± 95% CI (shaded area), of the binned DFS data. Data of core –λPP in **b** and **c** were taken from Fig. 3b,e.

Reviewer 1 and 3: • Why do the authors use BI here and does this impact the findings? This should be discussed as what is most physiologically relevant is the epinephrine activated receptor, which in fact was used for the GRK5 induced phosphorylation.

Authors: We used BI in our experiments because, as opposed to epinephrine, BI is a full agonist with pM-affinity and a very long residence time on β_2AR that induces maximal activation of β_2AR[19]. Furthermore, BI is chemically very stable compared to epinephrine, which is prone to oxidation and requires addition of an antioxidant (ascorbic acid) to the buffer solution. In initial tests we observed some interference with our single-molecule force spectroscopy measurements upon addition of ascorbic acid. And since β_2AR-βarr2 interactions, especially in core complexes, are very rare in our experiments, we needed a potent and stable agonist to shift as many β_2AR molecules as possible towards an active conformation. Hence, BI is the ideal agonist in our experimental setup. However, we acknowledge that BI is a synthetic ligand and therefore not the most physiologically relevant. We included a short description of the selection criteria in the manuscript (see Results, section '*Monitoring spontaneous binding events of βarr2 to β_2AR*').

Reviewer 1: • Supplementary Figure 2b. Are these representative images? What is the N of these experiments?

Authors: We apologize for the confusion created and adapted the legend of Supplementary Fig. 2b. AFM topographs and confocal microscopy images are representative of three independent replicates of each condition.

Reviewer 1 and 3: • Page 2, lines 130-132. "*The binding rate, i.e., binding events per total number of FT curves, was 2.0%, while in control experiments on membranes without β2AR it was 0.1%, indicating that the majority of detected events correspond to the spontaneous binding of βarr2 to β2AR*." Where is this data shown? There is no figure reference after this sentence. Supplementary Figure 4 is related, but I don't see data/distributions of binding events showing the binding events are 2.0% for β2AR-containing samples and 0.1% for samples prepared in the absence of receptor. Perhaps the representative images shown in Supplementary Figure 4a,b are where such distributions are derived. The authors should show these distributions and associated statistical comparisons, and how many active imaging areas and independent replications were carried out to support their claim.

Authors: Thank you for pointing out this inaccuracy. In fact, the so-called height-clamp measurements the reviewer refers to are conducted in a non-imaging mode of the AFM. In other words, the AFM only collects force-time (FT) curves but no topographical information during this measurement. To locate receptors in supported membranes, we first imaged the sample surface with the AFM cantilever and then performed height-clamp measurements in areas where we had located receptors. In total, we collected 8305 FT curves with 21 cantilevers from 5 independent functionalizations on membranes containing β_2AR. In those 8305 FT curves, we observed 165 β_2AR-βarr2 (un-)binding events (≈ 2%). On membranes without β_2AR, we collected 3038 FT curves with 13 cantilevers from 5 independent functionalizations and observed 3 adhesive events (≈ 0.1%). We have plotted the data distributions in Fig. R9 and compared them by applying a Mann-Whitney test. This statistical comparison yields a P value < 0.0001.

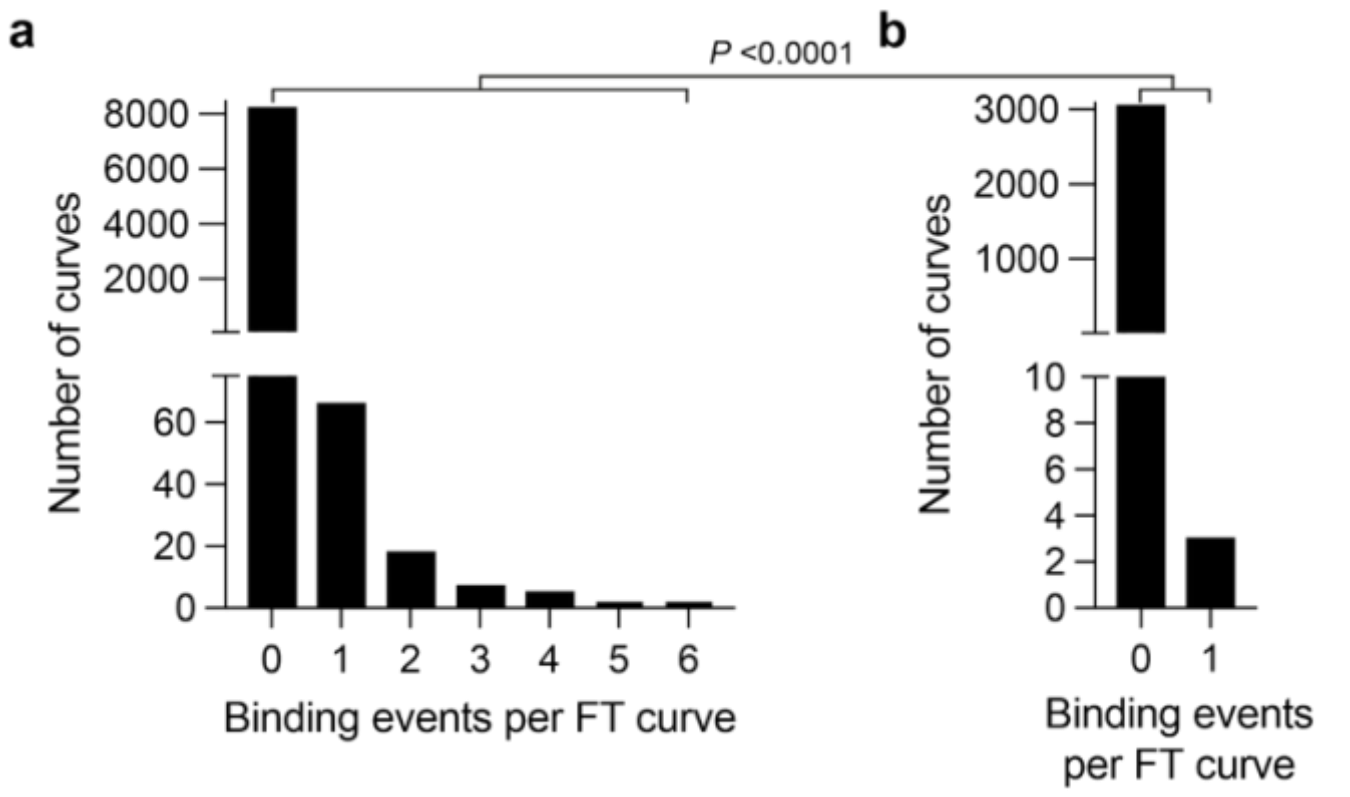

Figure R9, included in the revised manuscript as Supplementary Fig. 4a,b. Analysis of multiple binding events in height clamp measurements. a and **b**, Number of individual binding events detected per force-time (FT) curve in (**a**) lipid membranes containing β$_2$AR and (**b**) lipid membranes without β$_2$AR. *P* value comparing both distributions shown in **a** and **b** is obtained from a Mann-Whitney test. The data analyzed were taken from Fig. 1.

We have included the data distributions of above-mentioned experiments in the revised manuscript, in which we also explain these experiments and differences in greater detail (see Results, section '*Monitoring spontaneous binding events of βarr2 to β$_2$AR*', and new in Supplementary Fig. 4).

Reviewer 1 and 3: • Line 175: "*Taken together, the experiments show that βarr2 binds the phosphorylated C-tail of β2AR rapidly, within milliseconds*." I am not sure what is meant by milliseconds here. It would seem that all we can conclude is that some binding can take place within 16 ms when the βarr2 is appropriately positioned nearby. Again, the model system is powerful and appreciated, but βarr2 is normally diffusing in the cytoplasm, so we have to remember that in these experiments it is being tethered very close to the final binding position.

Authors: We agree with the reviewer that, as mentioned above, we cannot measure the recruitment of βarr2 from the cytosol or from a membrane-associated state to β$_2$AR. Therefore, we have refrained from using the term recruitment in our manuscript and use the term 'binding' instead to refer to arrestin engaging the receptor tail or core. However, our measurements can reveal that once βarr2 is in close proximity (≈ 4 – 8 nm) to β$_2$AR it can bind within milliseconds. Accordingly, we have revised the cited sentence, now explicitly stating that the time scale measured in our experiments refers to the binding between βarr2 and β$_2$AR being brought into close proximity of each other.

NSMB-A50618 “Roles of ligand, phosphorylation, and membrane in assembling human β2-adrenergic receptor-β-arrestin complexes” NSMB-A50618

Point-by-point response to the comments of Reviewer #2

Reviewer 2: The research on β-arrestins, conducted by a team of world leading experts, is pioneering in its detailed exploration of how these proteins complex with G protein-coupled receptors (GPCRs) to regulate signaling. β-arrestins, particularly β-arrestin1 and β-arrestin2, play crucial roles in desensitizing and internalizing GPCRs, which are essential for cellular responsiveness. This study advances our understanding by demonstrating how β-arrestins interact with various structural elements of GPCRs—such as the C-tail and core—and how these interactions impact signaling outcomes. The use of dynamic single-molecule force spectroscopy and molecular dynamics simulations provides profound insights into the mechanical and thermodynamic properties of β-arrestin-GPCR complexes. These findings reveal that β-arrestins can adopt multiple active conformations depending on their interaction with the GPCR, leading to different signaling pathways. Moreover, the role of the lipid environment, particularly phosphoinositides, in modulating these interactions highlights a significant layer of complexity in GPCR regulation. This groundbreaking work has significant implications for drug development, offering potential for therapies that more precisely target GPCR signaling in diseases such as cancer and diabetes. The research sets a new standard in the field, combining sophisticated experimental techniques with a deep understanding of molecular interactions to unravel the complexities of cell signaling.

Authors: We thank the reviewer for the thorough review and the encouraging and constructive comments. In the following, we explain point-by-point how we addressed each specific comment of the reviewer.

Reviewer 2: The height clamp experiments measuring the single binding events between barr2 and b2AR are fascinating and extremely interesting. To my opinion they show for the first time directly and in real time the on and off binding of arrestin to a GPCR. The current analysis of the height clamp data suggests single binding and unbinding events of the barr2-b2AR complex. This suggests that the complex can bind only in one step and that further binding steps cannot occur under externally applied force. Did the authors analyze whether the binding events detected in the height clamp mode show multiple binding or/and unbinding steps? How frequently were such binding steps detected if at all? How do these steps distribute in force/time? Is there any indication of multiple binding steps within this (un-)binding events?

Authors: Thank you for your encouraging comments regarding our height clamp experiments and for requesting an in-depth analysis of our height clamp data characterizing the single binding events between βarr2 and β_2AR. In Fig. R10 we provide further analysis of single and multiple (un-)binding events detected in single force-time (FT) curves on phospholipid membranes with and without β_2AR. The analysis shows that multiple binding events only occur in the presence of β_2AR (Fig. R10a,b). These multiple binding events occur less frequently (34% of binding events) than single events. In Fig. R10c we analyzed the time Δt between consecutive binding events. Most multiple binding events follow each other within 100 ms. To further

investigate whether rapid unbinding and rebinding of βarr2 could lead to prolonged or stronger rebinding to β_2AR, we calculated the difference in the duration $\Delta\Delta t$ and binding force $\Delta\Delta F$ between consecutive binding events (Fig. R10d,e). We compared $\Delta\Delta t$ and $\Delta\Delta F$ of events following each other rapidly within the lifetime of the β_2AR-βarr2 complex obtained from our height clamp experiments (< 50 ms) to $\Delta\Delta t$ and $\Delta\Delta F$ of binding events that are spaced by ≥ 50 ms, which is more than the lifetime of the β_2AR-βarr2 complex. The analysis revealed no statistical difference in $\Delta\Delta t$ or $\Delta\Delta F$ between both groups. Therefore, we conclude that ***i)*** βarr2 binds and unbinds frequently to and from β_2AR in our height clamp measurements and that ***ii)*** the binding events, even if they follow each other rapidly, are independent and do not affect each other.

Next, we addressed whether there are multiple (un-)binding steps within a single binding event. To this end, we calculated the variance in force both considering a single (un-)binding step and multiple (un-)binding steps (Fig. R10f). If the variance in force considering multiple (un-)binding steps was smaller than the variance with only one (un-)binding step, there may indeed occur multiple binding and unbinding steps within one binding event. However, we found that the difference in variance between multiple (un-)binding steps and a single (un-)binding step is larger than 10 only in 10 binding events, which is ≈ 6% of all 165 binding events detected. In Fig. R10g we show the three binding events with the largest difference in variance. Despite the lower variance when considering multiple (un-)binding steps, it is difficult to distinguish any possible substep from noise or drift within an binding event. Considering also the small number of FT curves that potentially have detected multiple binding and unbinding steps, we do not find any statistically significant indication of such substeps within the binding events.
We have included above results in our revised manuscript (see Results, section '*Monitoring spontaneous binding events of βarr2 to β_2AR*', and new in Supplementary Fig. 4).

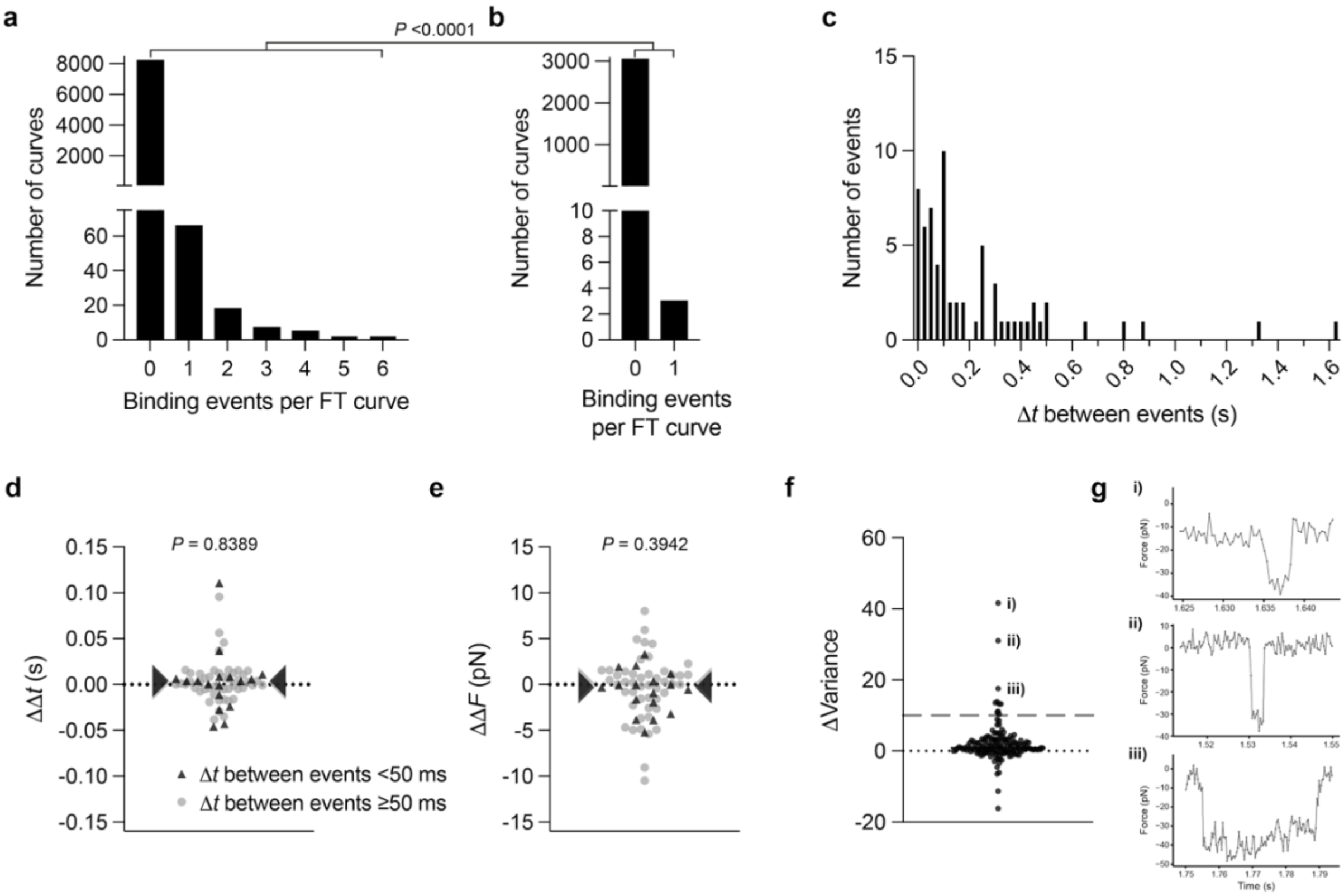

Figure R10, included in the revised manuscript as Supplementary Fig. 4. Analysis of multiple binding events and force steps in height clamp measurements. a and **b**, Number of individual binding events detected per force-time (FT) curve in (**a**) phospholipid membranes containing β_2AR and (**b**) phospholipid membranes without β_2AR. *P* value comparing both distributions shown in **a** and **b** is obtained by applying a Mann-Whitney test. **c**, Distribution of the time Δt between consecutive binding events of the same FT curve. **d** and **e**, Comparison of the difference in binding event duration $\Delta\Delta t$ (**d**) and binding force $\Delta\Delta F$ (**e**) between two consecutive events separated by < 50 ms (black triangles) and ≥ 50 ms (grey circles). $\Delta\Delta t$ and $\Delta\Delta F$ were calculated by subtracting the duration/change in force of the preceding binding event from the duration/change in force of the following event. *P* values comparing the datasets with Δt between events < 50 ms and ≥ 50 ms in (**d**) and (**e**) are obtained from Mann-Whitney tests. **f**, Difference of the variance of the force of a binding event if a single force step was assumed and if multiple force steps were assumed. The dotted line marks no difference in variance and the dashed line marks a difference in variance of 10. **g**, Examples of single binding events detected in FT curves. *i)*, *ii)*, and *iii)* show the single events with the largest difference in variance, *i.e.*, the highest probability of multiple force steps. The data analyzed were taken from Fig. 1.

Reviewer 2: The authors present an impressive amount of DFS experiments to characterize the various conditions of barr2-b2AR complex formation. However the authors should cite the pioneering work of the group in which they detected ligand binding to GPCRs. They find that the complexes can form via several consecutive steps. However, do these complexes also unbind via several steps? To answer this question the authors could analyze the rupture/ dissociation events of the complexes, and whether these events occur in single or multiple steps. Such analysis may be challenging, but rewarding in contributing towards the understanding how the complexes stabilize, destabilize and restablize through rebinding.

Authors: Thank you for your supportive comment and for recommending to cite some of our work related to our manuscript. We have added the sentence "*AFM has proven to be a valuable tool for characterizing the dynamic binding of GPCR-ligand complexes*

on the single-molecule level[20,21].” in the first paragraph of our discussion and cite the work referred to.

The reviewer further recommends analyzing the rupture/dissociation events of β2AR-βarr2 complexes, to determine whether these (un-)binding events occur in single or multiple steps. We followed the reviewer’s suggestion and further analyzed our dynamic force spectroscopy (DFS) data to identify and characterize the potential substeps that can occur upon mechanically separating β2AR-βarr2 complexes (Fig. R11). To determine whether we observe multiple steps (substeps) in the (un-)binding events in our DFS data, we visually inspected all force-distance (FD) curves recorded at the two slowest retraction speeds of 203 and 501 nm s^{-1} (Fig. R11a). On average, only 6.9% of the 2104 FD curves contained (un-)binding events showing substeps. Examples of such FD curves are given for each β2AR-βarr2 complex (Fig. R11b-d). Considering that the probability of detecting an (un-)binding event of β2AR-βarr2 in our DFS measurements is extremely low (*e.g.*, 0.24% of all FD curves collected with the β2AR-βarr2 tail-core complex in the presence of PIP_2 at 121 ms interaction time) and that on average only a low percentage of FD curves show (un-)binding events that may show substeps, we can unfortunately not provide any statistically reasonable analysis of these substeps.

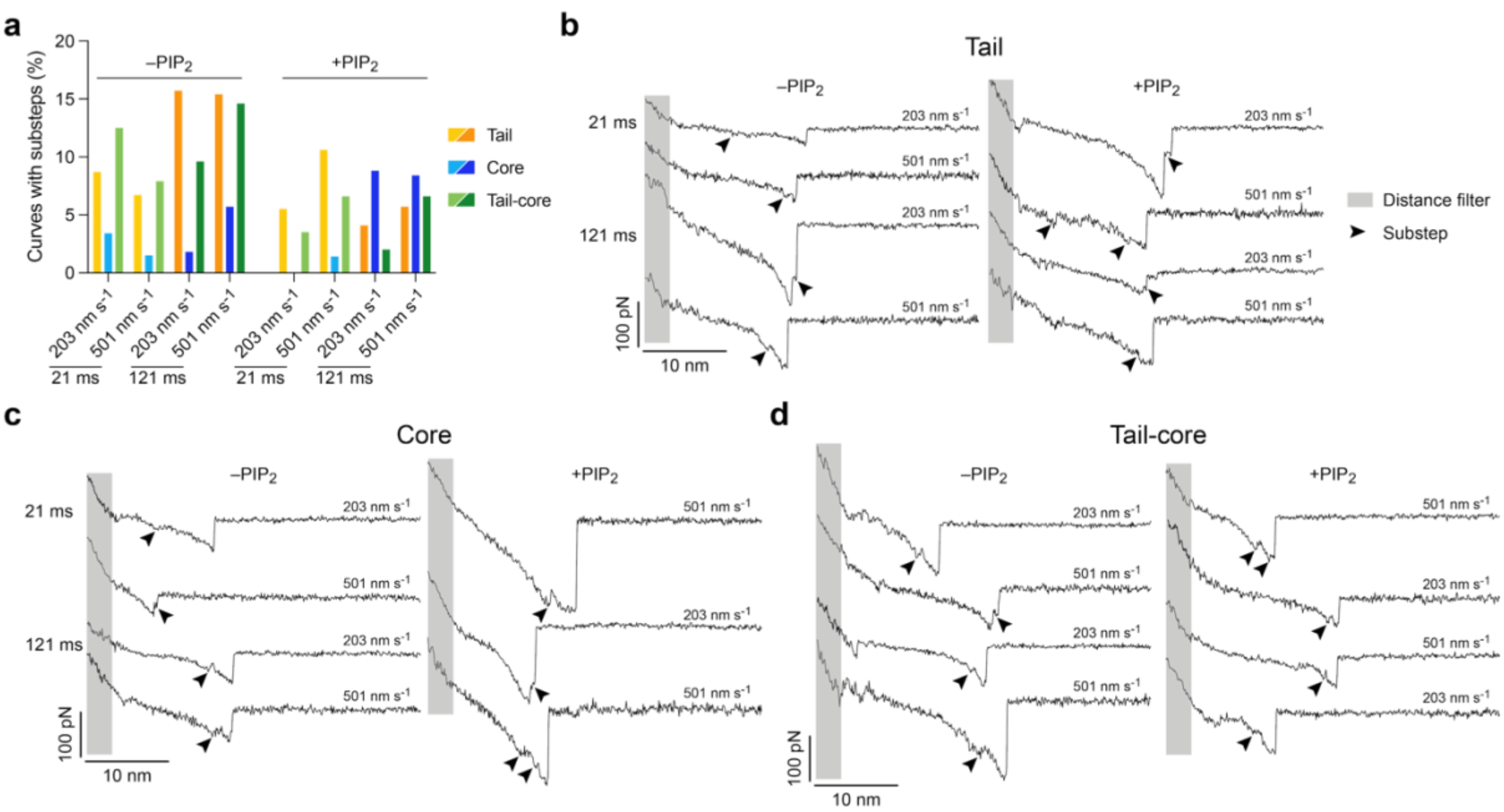

Figure R11. Analysis of substeps of (un-)binding events in retraction force-distance (FD) curves recorded in DFS experiments. a, Bar graph showing the percentage of FD curves resulting from a β2AR-βarr2 (un-)binding event containing substeps. Datasets are taken at retraction speeds of 203 or 501 nm s^{-1}, AFM tip–sample interaction time of 21 and 121 ms, and the membranes embedding β2AR (without PIP_2 and with PIP_2). **b-d**, Example FD curves with one or multiple substeps (black arrows) of β2AR-βarr2 (**b**) tail, (**c**) core, and (**d**) tail-core complexes. The grey area marks the distance filter within which no events are considered (see Methods, section ‘*Analysis of FT and FD curves*’). Data were taken from Fig. 2 and 3.

Reviewer 2: If I understand correctly, the authors applied the Kernel-Density Estimation to determine the most probable rupture force and loading rate. It would be nice to show the fits of the individual data sets and estimate the quality/robustness of the fits.

Authors: We indeed applied the kernel density estimation (KDE) to determine the most probable rupture force and loading rate. We applied KDE because it does not assume an underlying distribution of the data and therefore is less biased compared to other methods, which could be used for such estimations. We have now included a new Supplementary Fig. 11 (shown here as Fig. R12) showing the individual data sets and the corresponding KDEs for both the rupture force and the loading rate. Comparison of the KDE fit with the underlying distribution of the experimental data shows that all fits are robust and accurately capture the features of the data distribution.

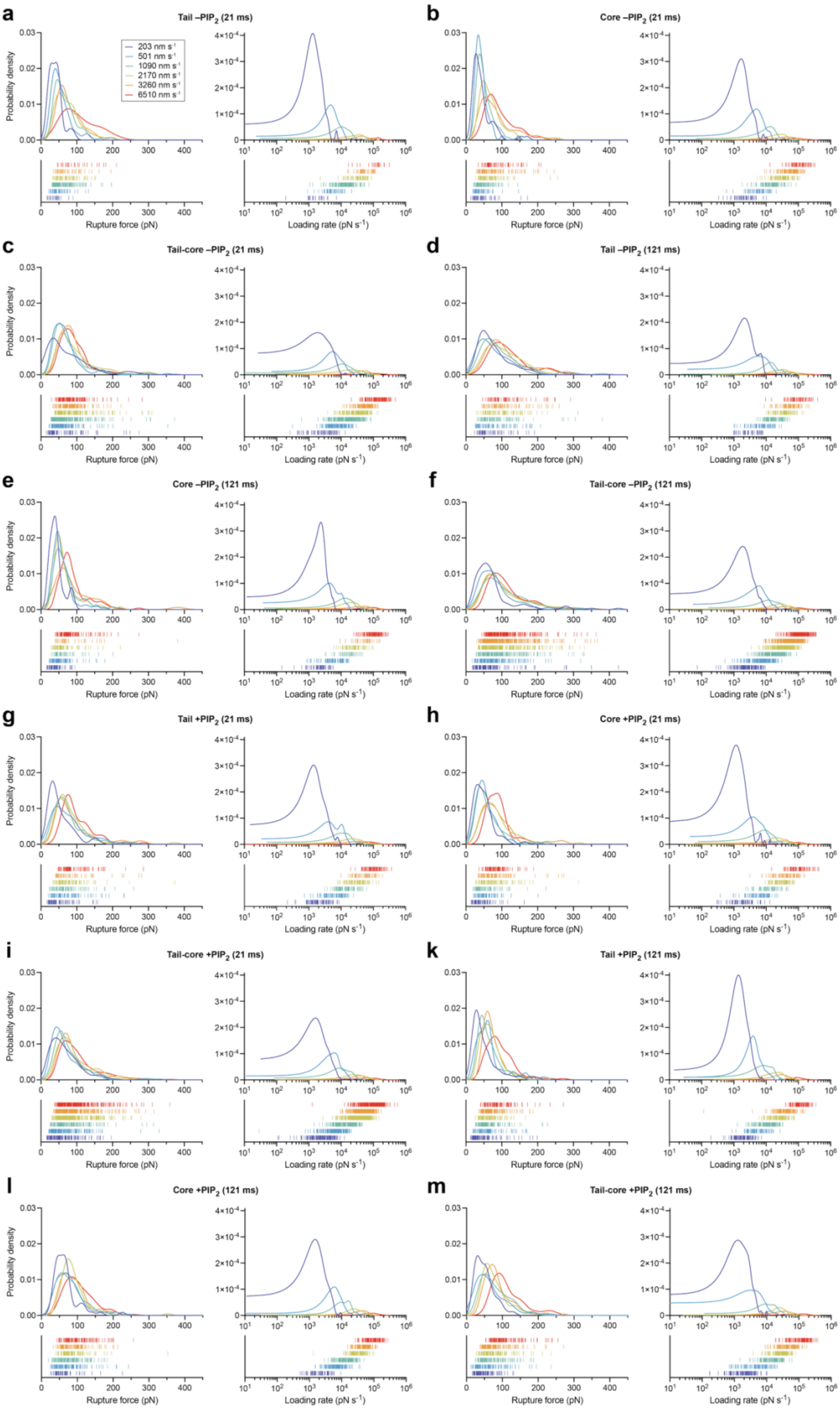
a
Tail –PIP2 (21 ms)
b
Core –PIP2 (21 ms)
c
Tail-core –PIP2 (21 ms)
d
Tail –PIP2 (121 ms)
e
Core –PIP2 (121 ms)
f
Tail-core –PIP2 (121 ms)
g
Tail +PIP2 (21 ms)
h
Core +PIP2 (21 ms)
i
Tail-core +PIP2 (21 ms)
k
Tail +PIP2 (121 ms)
l
Core +PIP2 (121 ms)
m
Tail-core +PIP2 (121 ms)
203 nm s-1
501 nm s-1
1090 nm s-1
2170 nm s-1
3260 nm s-1
6510 nm s-1
Probability density
Rupture force (pN)
Loading rate (pN s-1)

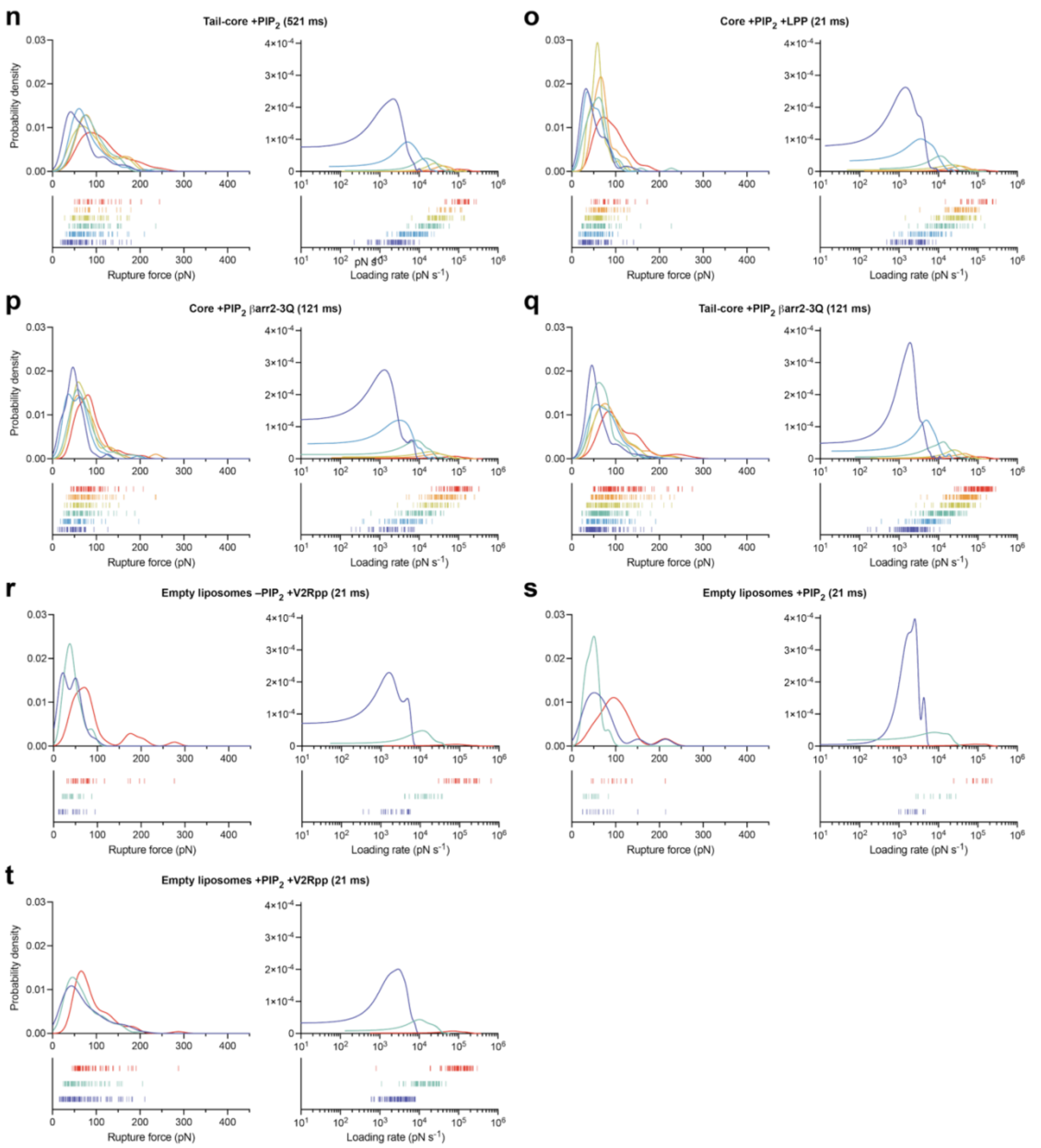

Figure R12, included in the revised manuscript as Supplementary Fig. 11. Kernel density estimation (KDE) of DFS datasets. a-f, KDEs of β2AR-βarr2 complexes measured in the absence of PIP_2 ($–PIP_2$) at 21 ms for (**a**) tail, (**b**) core, and (**c**) tail-core complexes and at 121 ms for (**d**) tail, (**e**) core, and (**f**) tail-core complexes. **g-q**, KDEs of β2AR-βarr2 complexes measured in the presence of PIP_2 ($+PIP_2$) at 21 ms for (**g**) tail, (**h**) core, and (**i**) tail-core complexes, at 121 ms for (**k**) tail, (**l**) core, and (**m**) tail-core complexes, at 521 ms for (**n**) the tail-core complex, and at 21 ms for the core complex treated with lambda protein phosphatase (+LPP). KDEs measured with βarr2-3Q at 121 ms for (**o**) core and (**p**) tail-core complexes. The top graphs show kernel density estimations (KDEs), while the bottom graphs show the distribution of rupture force (left) and loading rate (right) separated by the retraction speed. DFS data were taken from Fig. 2 and 3, Extended Data Fig. 3, 4, and 5, and Supplementary Fig. 10.

Reviewer 2: Alternative models for experimental data analysis. Did the authors test alternative models for data analysis? If there are suitable models, do they reveal similar insight?

Authors: There are alternative models to analyze DFS data. In addition to the Bell-Evans model[17], which we used in this study, there are, for instance, the Friddle-Noy-

De Yoreo model[22] and the Dudko-Hummer-Szabo model[23]. While the latter two models also take into account the rebinding of complexes during the separation process or different shapes of the free-energy landscape, they require detailed knowledge of the force transducer system, *i.e.,* the force extension behavior of the cantilever and of the flexible PEG_{27}-linker that covalently tethers the molecule of interest to the tip of the cantilever[3]. As demonstrated earlier by Alsteens *et al.*[20], the effective spring constant of a force transducer system can be approximated for a relatively simple system that, for example, uses a short linear peptide as the tethered molecule of interest. However, this approximation becomes problematic if the molecule of interest tethered to the cantilever is a large, complex protein like βarr2 and can introduce considerable uncertainties in the effective spring constant. Thus, we considered the Bell-Evans model to be the most robust model for analyzing our DFS data and comparing the thermodynamic and kinetic parameters of the various experimental conditions investigated. Below we show that the Bell-Evans model indeed proves to fit our DFS data robustly, resulting in parameters that are comparable to those previously published in literature[24-26] as discussed in the Results section '*Fully engaged* β_2*AR-βarr2 complexes take about hundred milliseconds to assemble*' (Fig. R13). Nevertheless, we tried to fit our DFS data also with the Friddle-Noy-De Yoreo model but observed that it failed to properly fit. Thus, we can be confident that the Bell-Evans model provides a good fitting to our DFS data.

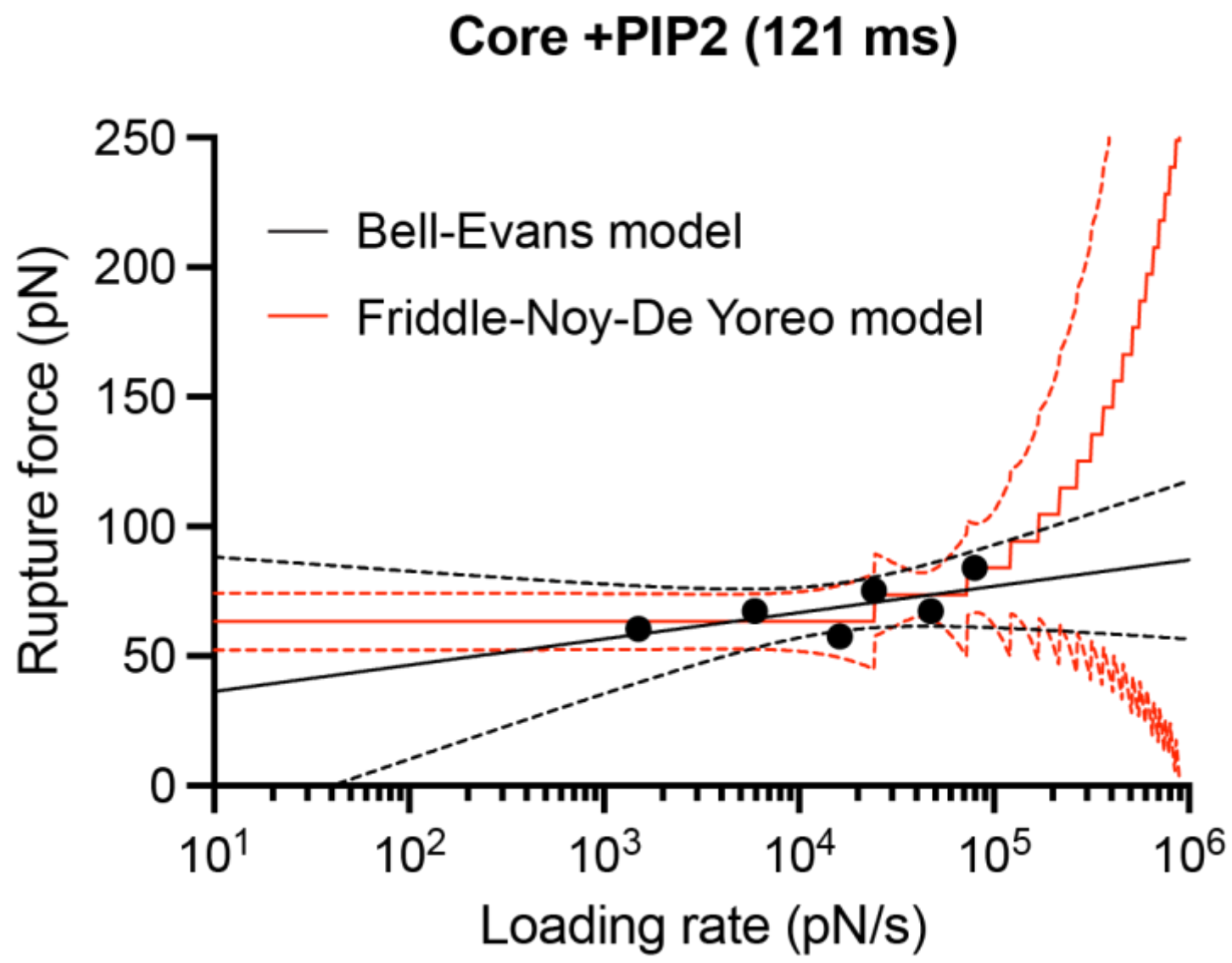

Figure R13. Comparison of the Bell-Evans model and the Friddle-Noy-De Yoreo model to fitting the DFS data. The Bell-Evans model (black) robustly fits the binned DFS data (circles) of β_2AR-βarr2 core complexes in the presence of PIP_2 at 121 ms interaction time while the Friddle-Noy-De Yore model (red) breaks down. Solid lines represent fits and dashed lines denote ±95% confidence intervals. DFS data was taken from Fig. 3b.

Reviewer 2: The authors use the Freedman-Diaconis rule to bin their data, what was the rationale for selecting this rule? Are there suitable other rules that could be applied and how would they change the outcome of the analysis?

Authors: We have chosen the Freedman-Diaconis rule[27] because it does not assume any particular distribution of the unbinned height clamp data, unlike, for instance, the Scott's choice[28]. As the Freedman-Diaconis rule results in a relatively small bin width, it accurately represents the distribution of our unbinned height clamp data.

Nevertheless, we tested three other methods to determine the number of bins, namely the square root method (number of bins = square root of the number of data points rounded to the next integer), the Sturges' formula[29], and the Scott's choice[28] (Fig. R14). Each of the three models resulted in the exact same parameters and thus did not change the outcome of the data analysis.

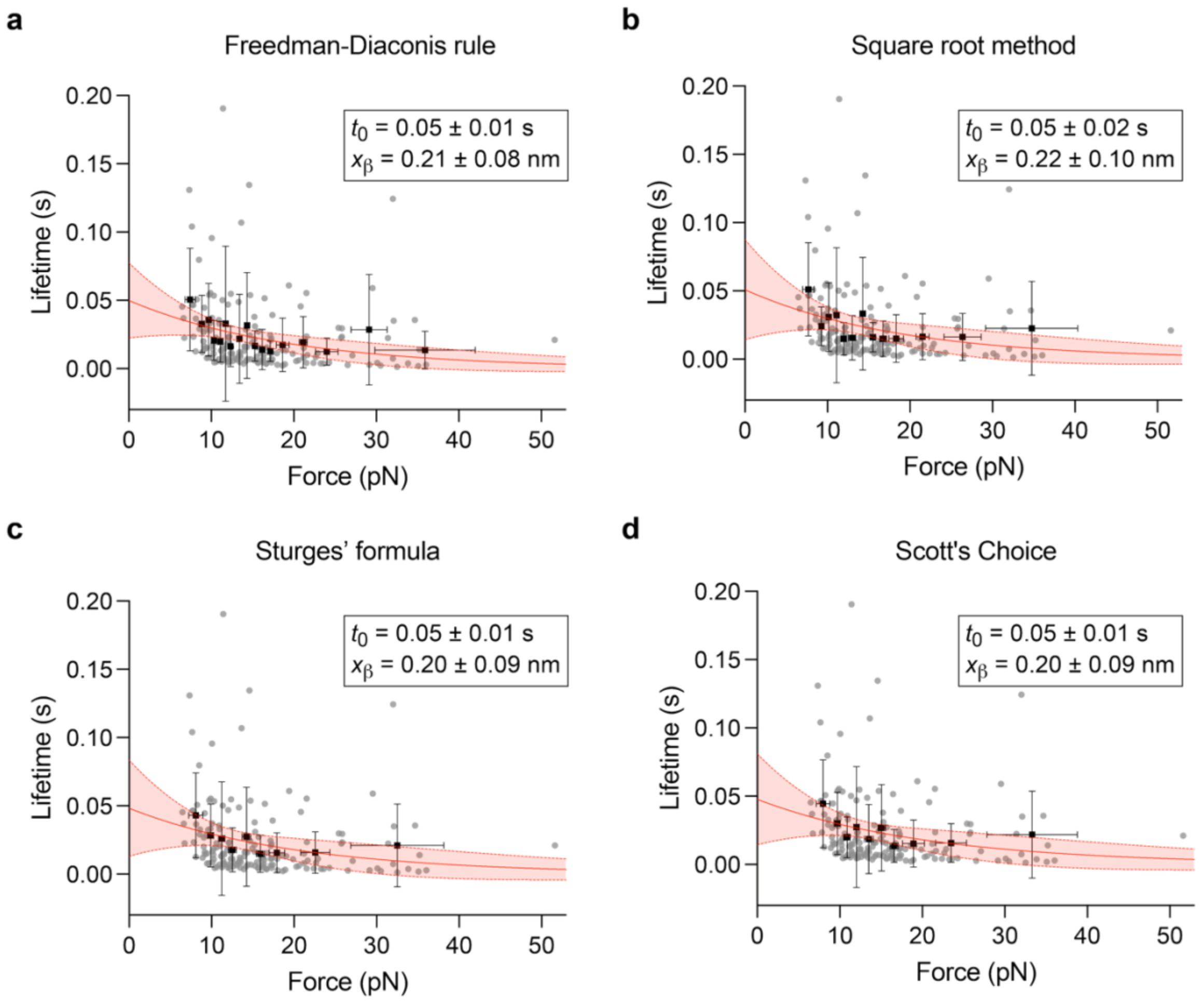

Figure R14. Comparison of binning methods applied to the height clamp data. Height clamp data (grey points) were binned (black squares) using the (**a**) Freedman-Diaconis rule, (**b**) square root method, (**c**) Sturges' formula, and (**d**) Scott's Choice. Insets show the parameters t_0 and x_β obtained from fitting the binned data with the Bell model (red line) ± 95% confidence interval (dashed line).

Reviewer 2: Fig. 2f,g,h. The data points in the whisker plots, do they represent single replicates? Was each replicate recorded with a new sample preparation and functionalized cantilever? Please specify.

Authors: Thank you. The data points in the whisker plots do represent single replicates. A replicate is defined as a force-volume scan in which the individual FD curves are recorded at a defined retraction speed. We have revised the legend of Fig. 2 to include the number of replicates, cantilevers, and independent functionalizations for each experimental condition.

Reviewer 2: Fig. 3d,e,f. The data points in the whisker plots, do they represent single replicates? Was each replicate recorded with a new sample preparation and functionalized cantilever? Please specify.

Authors: Thank you. The data points in the whisker plots represent single replicates. A replicate is defined as a force-volume scan recorded with a defined retraction speed. We revised the legend of Fig. 3 to include the number of replicates, cantilevers, and independent functionalizations for each condition.

Reviewer 2: Fig. 4f. Please show data points wherever possible.

Authors: Thank you, we have revised Fig. 4f, now showing all data points. The revised Fig. 4f is shown below.

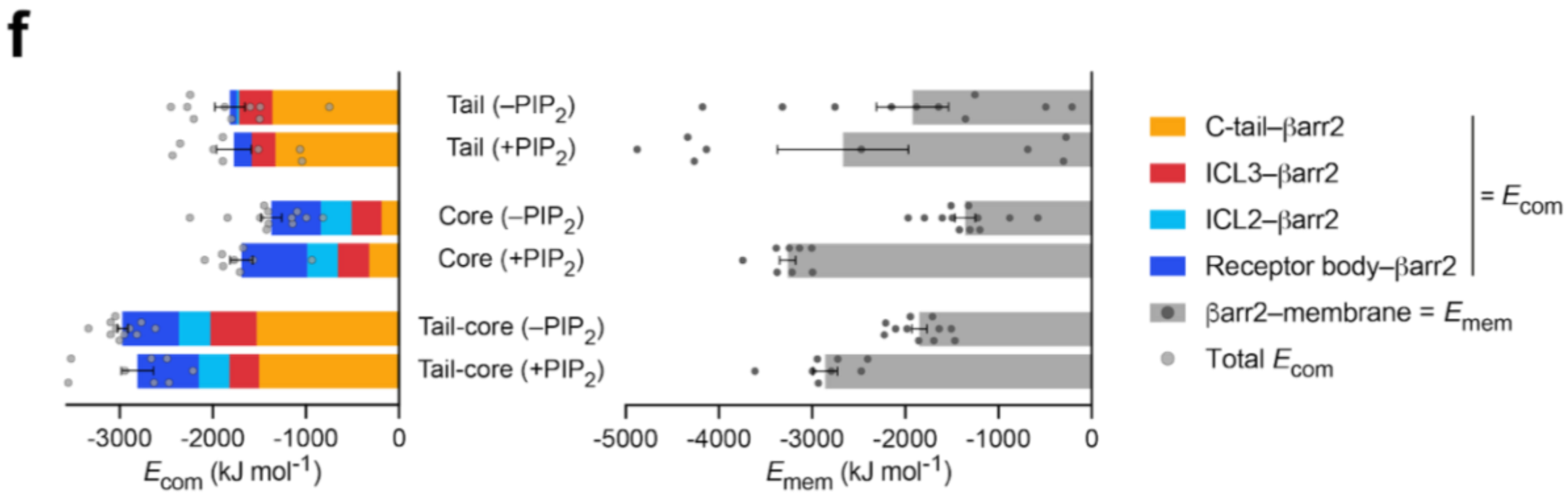

Reviewer 2: Fig. 5e,f. Please show data points wherever possible.

Authors: Thank you, we have revised Fig. 5e,f (now Fig. 5c,d) to show all data points. The revised Fig 5c,d is shown below.

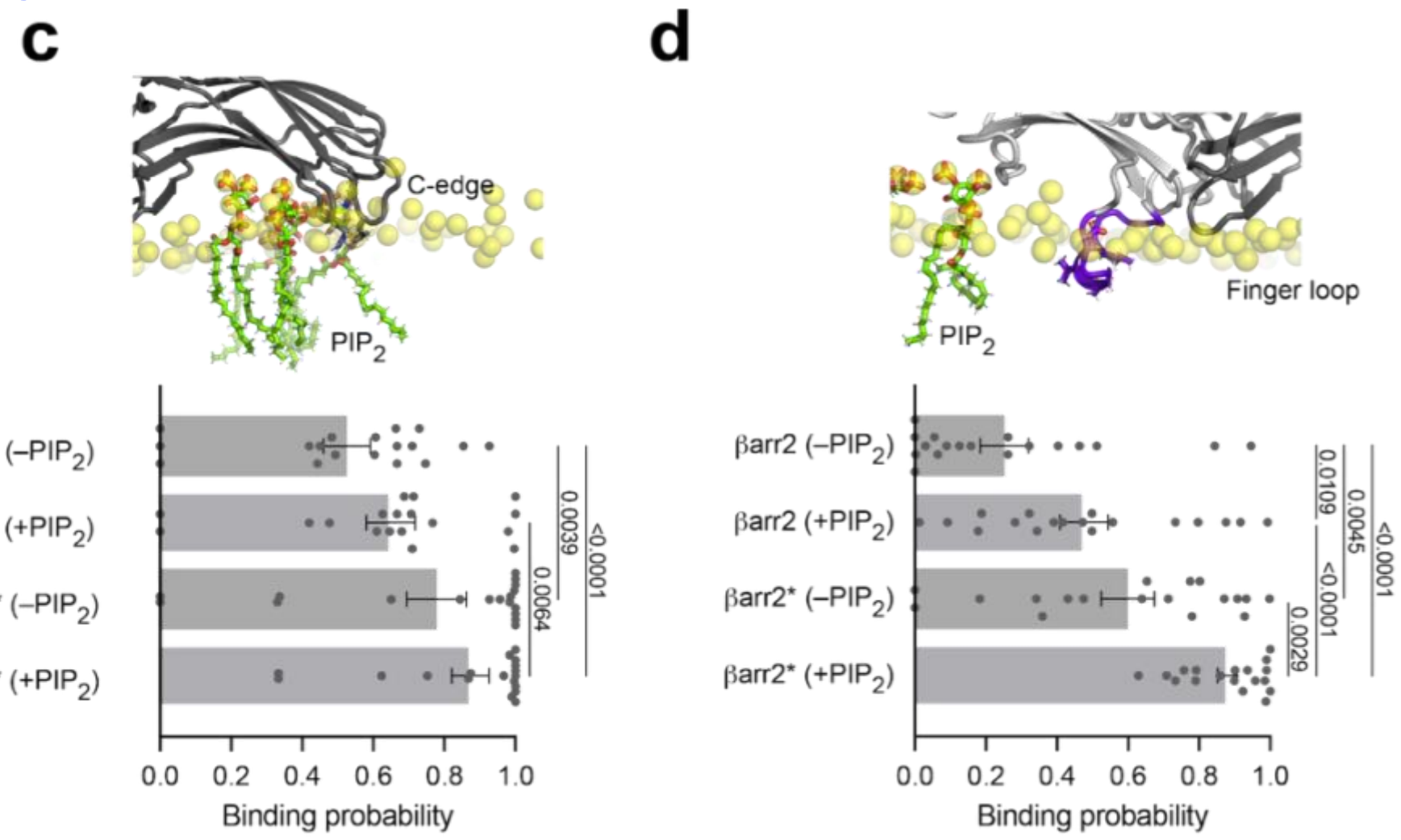

NSMB-A50618 “Roles of ligand, phosphorylation, and membrane in assembling human β2-adrenergic receptor-β-arrestin complexes

Point-by-point response to the comments of Reviewer #3

Reviewer #3 co-reviewed with Reviewer #1

Authors: Thank you for your encouraging and constructive co-reviewing with Reviewer 1, which guided us to hone our manuscript. In our response to the comments of Reviewer 1 we describe point-by-point how we have addressed each of the reviewer comments and suggestions.

References

1 Gaidarov, I., Krupnick, J. G., Falck, J. R., Benovic, J. L. & Keen, J. H. Arrestin function in G protein-coupled receptor endocytosis requires phosphoinositide binding. *EMBO J* **18**, 871-881 (1999). https://doi.org/10.1093/emboj/18.4.871

2 Ring, A. M. *et al.* Adrenaline-activated structure of beta2-adrenoceptor stabilized by an engineered nanobody. *Nature* **502**, 575-579 (2013). https://doi.org/10.1038/nature12572

3 Muller, D. J. *et al.* Atomic Force Microscopy-Based Force Spectroscopy and Multiparametric Imaging of Biomolecular and Cellular Systems. *Chem Rev* **121**, 11701-11725 (2021). https://doi.org/10.1021/acs.chemrev.0c00617

4 Janetzko, J. *et al.* Membrane phosphoinositides regulate GPCR-beta-arrestin complex assembly and dynamics. *Cell* **185**, 4560-4573 e4519 (2022). https://doi.org/10.1016/j.cell.2022.10.018

5 Grimes, J. *et al.* Plasma membrane preassociation drives beta-arrestin coupling to receptors and activation. *Cell* **186**, 2238-2255 e2220 (2023). https://doi.org/10.1016/j.cell.2023.04.018

6 Eichel, K. *et al.* Catalytic activation of beta-arrestin by GPCRs. *Nature* **557**, 381-386 (2018). https://doi.org/10.1038/s41586-018-0079-1

7 Latorraca, N. R. *et al.* Molecular mechanism of GPCR-mediated arrestin activation. *Nature* **557**, 452-456 (2018). https://doi.org/10.1038/s41586-018-0077-3

8 Asher, W. B. *et al.* GPCR-mediated beta-arrestin activation deconvoluted with single-molecule precision. *Cell* **185**, 1661-1675 e1616 (2022). https://doi.org/10.1016/j.cell.2022.03.042

9 Kawakami, K. *et al.* Heterotrimeric Gq proteins act as a switch for GRK5/6 selectivity underlying beta-arrestin transducer bias. *Nat Commun* **13**, 487 (2022). https://doi.org/10.1038/s41467-022-28056-7

10 Jala, V. R., Shao, W. H. & Haribabu, B. Phosphorylation-independent beta-arrestin translocation and internalization of leukotriene B4 receptors. *J Biol Chem* **280**, 4880-4887 (2005). https://doi.org/10.1074/jbc.M409821200

11 Gomes, A. A. S. *et al.* Lipids modulate the dynamics of GPCR:beta-arrestin interaction. *Nat Commun* **16**, 4982 (2025). https://doi.org/10.1038/s41467-025-59842-8

12 Mukhaleva, E. *et al.* Cellular lipids regulate the conformational ensembles of the disordered intracellular loop 3 in beta2-adrenergic receptor. *iScience* **27**, 110086 (2024). https://doi.org/10.1016/j.isci.2024.110086

13 Song, W., Yen, H. Y., Robinson, C. V. & Sansom, M. S. P. State-dependent Lipid Interactions with the A2a Receptor Revealed by MD Simulations Using In Vivo-Mimetic Membranes. *Structure* **27**, 392-403 e393 (2019). https://doi.org/10.1016/j.str.2018.10.024

14 Yen, H. Y. *et al.* PtdIns(4,5)P(2) stabilizes active states of GPCRs and enhances selectivity of G-protein coupling. *Nature* **559**, 423-427 (2018). https://doi.org/10.1038/s41586-018-0325-6

15 Balla, T. Phosphoinositides: tiny lipids with giant impact on cell regulation. *Physiol Rev* **93**, 1019-1137 (2013). https://doi.org/10.1152/physrev.00028.2012

16 Nasuhoglu, C. *et al.* Nonradioactive analysis of phosphatidylinositides and other anionic phospholipids by anion-exchange high-performance liquid chromatography with suppressed conductivity detection. *Anal Biochem* **301**, 243-254 (2002). https://doi.org/10.1006/abio.2001.5489

17 Evans, E. & Ritchie, K. Dynamic strength of molecular adhesion bonds. *Biophys J* **72**, 1541-1555 (1997).
18 Heng, J. *et al.* Function and dynamics of the intrinsically disordered carboxyl terminus of beta2 adrenergic receptor. *Nat Commun* **14**, 2005 (2023). https://doi.org/10.1038/s41467-023-37233-1
19 Rasmussen, S. G. *et al.* Structure of a nanobody-stabilized active state of the beta(2) adrenoceptor. *Nature* **469**, 175-180 (2011). https://doi.org/10.1038/nature09648
20 Alsteens, D. *et al.* Imaging G protein-coupled receptors while quantifying their ligand-binding free-energy landscape. *Nat Methods* **12**, 845-851 (2015). https://doi.org/10.1038/nmeth.3479
21 Dufrene, Y. F., Martinez-Martin, D., Medalsy, I., Alsteens, D. & Muller, D. J. Multiparametric imaging of biological systems by force-distance curve-based AFM. *Nat Methods* **10**, 847-854 (2013). https://doi.org/10.1038/nmeth.2602
22 Friddle, R. W., Noy, A. & De Yoreo, J. J. Interpreting the widespread nonlinear force spectra of intermolecular bonds. *Proc Natl Acad Sci U S A* **109**, 13573-13578 (2012). https://doi.org/10.1073/pnas.1202946109
23 Dudko, O. K., Hummer, G. & Szabo, A. Theory, analysis, and interpretation of single-molecule force spectroscopy experiments. *Proc Natl Acad Sci U S A* **105**, 15755-15760 (2008). https://doi.org/10.1073/pnas.0806085105
24 Mosslein, N., Pohle, L. G., Fuss, A., Bunemann, M. & Krasel, C. Residency time of agonists does not affect the stability of GPCR-arrestin complexes. *Br J Pharmacol* **179**, 4107-4116 (2022). https://doi.org/10.1111/bph.15846
25 Nuber, S. *et al.* beta-Arrestin biosensors reveal a rapid, receptor-dependent activation/deactivation cycle. *Nature* **531**, 661-664 (2016). https://doi.org/10.1038/nature17198
26 Zindel, D. *et al.* Engineered hyperphosphorylation of the beta2-adrenoceptor prolongs arrestin-3 binding and induces arrestin internalization. *Mol Pharmacol* **87**, 349-362 (2015). https://doi.org/10.1124/mol.114.095422
27 Freedman, D. & Diaconis, P. On the histogram as a density estimator:L2 theory. *Zeitschrift für Wahrscheinlichkeitstheorie und Verwandte Gebiete* **57**, 453-476 (1981). https://doi.org/10.1007/BF01025868
28 Scott, D. W. On optimal and data-based histograms. *Biometrika* **66**, 605-610 (1979). https://doi.org/10.1093/biomet/66.3.605
29 Sturges, H. A. The Choice of a Class Interval. *Journal of the American Statistical Association* **21**, 65-66 (1926). https://doi.org/10.1080/01621459.1926.10502161

NSMB-A50618A “Roles of ligand, phosphorylation, and membrane in assembling human β2-adrenergic receptor-β-arrestin complexes”

Point-by-point response to the comments of Reviewers #1 and #3

Reviewer 1 and 3: The authors have thoroughly addressed the concerns raised in our initial review. They performed several additional experiments that strengthen the key conclusions of the manuscript and clarify several points. The revisions demonstrate a careful and rigorous approach to addressing each comment, and the manuscript is significantly improved as a result.

Authors: We thank both reviewers for their thorough review and their encouraging and constructive comments, which guided us to improve our manuscript. In the following, we explain point-by-point how we addressed each remaining comment of the reviewers.

Reviewer 1 and 3: We have the following minor points that we believe would further enhance the clarity of the manuscript.

• Page 7, Lines 312-328. The experiments shown in Extended Data Figure 5 all appear to have been conducted in the presence of PIP2, but this is not mentioned until line 329: “However, in the presence of PIP2 the β2AR-βarr2-3Q tail-core complex…”. The late introduction of this detail, combined with the use of “however,” at line 329 initially suggested to us that lines 312-328 were describing experiments performed in the absence of PIP2. We suggest that the authors state early in the paragraph that all experiments were carried out in the presence of PIP2 to avoid this confusion.

Authors: We apologize for the confusion created. Following the reviewers’ comment we now clearly state earlier in the paragraph that all experiments were carried out in the presence of PIP_2 (see revised Manuscript, Results section ‘*PIP_2 promotes βarr2 binding to transmembrane core of β_2AR*’). We also omitted the use of ‘however’ to avoid confusion of the reader.

Reviewer 1 and 3: • Page 8, Lines 363-375. The presentation of simulation results in this paragraph is difficult to follow because the text alternates between conditions (± PIP2) without clear transitions. The authors conclude: “Taken together, our simulations show that PIP2 modulates the βarr2 interaction with receptor and membrane and orients the finger loop of βarr2 towards the membrane.” Our initial read of this was that the authors were inferring from the simulations that PIP2 increases the interaction with membrane based on the simulations, which we don’t see in the data (although we might have been confused by the different numbers of simulations for the different conditions), but we think they refer to the changes in orientation and not propensity to interact. Perhaps the language in this section could be made clearer for the reader.

Authors: We apologize for the confusion created. We have revised the language in the section and restructured the paragraph to more clearly explain which conditions are being discussed (see revised Manuscript).

Reviewer 1 and 3: • Figure 5a. We know this is a lot of work and don't propose that the authors do more experiments, but the presentation is a bit confusing to us. Why is there only 121 ms data in the absence of PIP2 along with 21 ms for V2Rpp activation – but no 21 ms data without the phosphopeptide? In contrast, there is 21 ms data for wildtype βarr2 with and without V2Rpp. This prevents comparison of 21 ms data for βarr2-3Q with wildtype βarr2 under the same conditions, although as the authors note it is similar in binding probability to wildtype βarr2 at 121 ms. Also, V2Rpp clearly increases binding probability with or without PIP2. It would be nice to know if V2Rpp can also increase binding of βarr2-3Q. This point seems particularly important given the report that activated wildtype βarr2 can stay associated with the plasma membrane in a PIP2-dependent way after dissociating from receptor– here activation increased membrane binding doesn't seem to be PIP2 dependent, which is an important point that we don't think is adequately discussed. We suggest the authors restructure this presentation, explicitly stating which conclusions are supported and which questions remain unresolved.

Authors: We again apologize for the confusion. In the revised manuscript we have restructured the presentation of these results and explicitly state which conclusions are supported by the experimental data (see revised Manuscript). Regarding the reviewers' question whether V2Rpp can increase binding of βarr2-3Q, we previously showed that V2Rpp has a similar effect on βarr2-3Q and βarr2-WT activation[1]. Thus, we expect V2Rpp to activate βarr2-3Q and thus promote membrane interactions, albeit to a lesser extent than with βarr2-WT, which in addition can also bind PIP_2.

Further, we would like to point out that while it is possible to increase the binding of βarr2 to the membrane in the absence of PIP_2 through activating βarr2, the stabilizing effect of PIP_2 is additive (Fig. 5). Activation of βarr2 alone results in a binding probability to the membrane, which is well below that of βarr2 complexed with β_2AR (Fig. 2, 3, and 5). Only the combination of βarr2 activation and PIP_2 allows βarr2 to establish membrane interactions, which strengths are in the range of those established with β_2AR (Fig. 2, 3, and 5). Thus, after engaging β_2AR, PIP_2 is required to stabilize active βarr2 at the membrane. The requirement of PIP_2 for receptor-independent βarr2 translocation at the plasma membrane to clathrin-coated structures was shown by Eichel and colleagues[2]. Lastly, PIP_2 was also shown to transiently increase the proportion of βarr in active-like states, underlining the importance and necessity of PIP_2[3].

Point-by-point response to the comments of Reviewer #2

Reviewer 2: I would first like to congratulate the authors for the thorough revision of the manuscript. The work is of high quality, and the improvements made in response to the previous round of review have significantly strengthened the study.

Authors: We thank the reviewer for the thorough review and the encouraging and constructive comments, which guided us to further improve our revised manuscript. In the following, we explain point-by-point how we addressed each specific comment of the reviewer.

Reviewer 2: I have only two minor comments that I believe would help further clarify the methodology and interpretation:
1. Contact time estimation
The manuscript reports an interaction time of 21 ms. Based on the experimental parameters provided (ramp size of 40 nm, cantilever stiffness of 0.07 N/m, and approach/retraction speeds of 1090 nm/s and 203 nm/s), the estimated contact time is closer to ~8 ms in the absence of a dwell period. It would be helpful if the authors could clarify how the value of 21 ms was obtained, and whether an additional dwell phase or a larger effective contact distance was involved.

Authors: Thank you. In our revised manuscript (Methods, section ‘*Analysis of FT and FD curves*’) we now detail how we have estimated the tip-sample contact time of ≈ 21 ms. We kindly note that in our SMFS experiments we always approached the AFM tip to the sample at the same speed of 1090 nm s^{-1}, while we retracted the AFM tip from the sample with 203 nm s^{-1}, 501 nm s^{-1}, 1090 nm s^{-1}, 2170 nm s^{-1}, 3260 nm s^{-1}, or 6510 nm s^{-1} (see methods, section ‘*Single-molecule force spectroscopy (SMFS)*’). To estimate the tip-sample contact time, we extracted the time from the first to the last tip-sample contact from the FT curves recorded under the experimental conditions described. This procedure was repeated for each of the retraction speeds applied in our experiments with three different cantilevers. The experimental measurement of the contact times showed that they differed slightly between the different retraction times, but because of the constant approach speed and the dwell time of the instrument, the contact time did not differ as largely as assumed by the reviewer. As a control whether these differences of a few ms would affect our measurements, we measured for each retraction speed the binding probability of βarr2 to β_2AR (Fig. R1). These binding probabilities did not differ significantly. Thus, to simplify the interpretability of our experimental data, we used the average tip-sample contact time of ≈ 21 ms as determined across all retraction speeds.

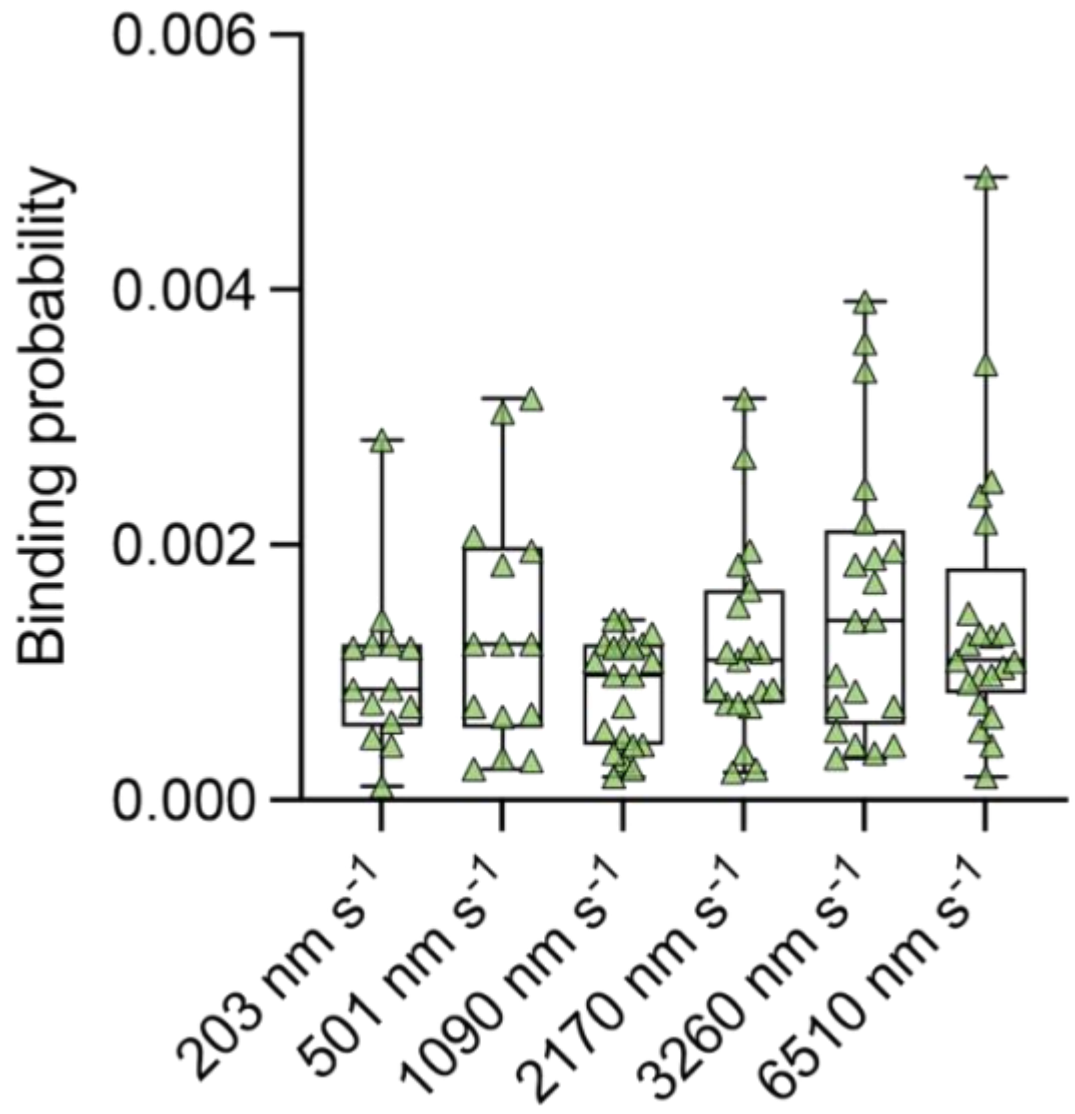

Tukey's multiple comparisons test	Adjusted P Value
203 nm s^{-1} vs. 501 nm s^{-1}	0.9109
203 nm s^{-1} vs. 1090 nm s^{-1}	0.9976
203 nm s^{-1} vs. 2170 nm s^{-1}	0.9798
203 nm s^{-1} vs. 3260 nm s^{-1}	0.4493
203 nm s^{-1} vs. 6510 nm s^{-1}	0.6462
501 nm s^{-1} vs. 1090 nm s^{-1}	0.6224
501 nm s^{-1} vs. 2170 nm s^{-1}	0.9990
501 nm s^{-1} vs. 3260 nm s^{-1}	0.9779
501 nm s^{-1} vs. 6510 nm s^{-1}	0.9984
1090 nm s^{-1} vs. 2170 nm s^{-1}	0.7919
1090 nm s^{-1} vs. 3260 nm s^{-1}	0.1215
1090 nm s^{-1} vs. 6510 nm s^{-1}	0.2403
2170 nm s^{-1} vs. 3260 nm s^{-1}	0.8318
2170 nm s^{-1} vs. 6510 nm s^{-1}	0.9524
3260 nm s^{-1} vs. 6510 nm s^{-1}	0.9992

Figure R1. Comparison of binding probabilities across retraction speeds. Binding probabilities measured between βarr2 and phosphorylated, BI-activated β_2AR embedded in a membrane containing PIP_2 were determined for every cantilever retraction speed. Binding probabilities are shown as boxplots where boxes mark the 25th to 75th percentiles, whiskers show minimum and maximum values, and the median (black bar) is indicated. Data points represent individual experimental replicates. An ordinary one-way ANOVA with Tukey's multiple comparisons test was performed with GraphPad Prism. The results of Tukey's multiple comparisons test are shown on the right.

Reviewer 2: 2. Use of the term "thermodynamic"
The manuscript refers to a thermodynamic ΔG derived from koff using an Arrhenius-type relation. However, this ΔG corresponds to the activation energy barrier for dissociation (ΔG‡), which is a kinetic parameter. The thermodynamic binding free energy ΔG° between the bound and unbound states depends on both koff and kon. To avoid confusion, I recommend clarifying that the quantity measured here is related to the activation barrier rather than the equilibrium free-energy difference.

Authors: Thank you kindly. We apologize for the confusion created and now clearly state that from the single molecule force-spectroscopy measurements presented in our manuscript we extract the kinetic or activation free-energy barrier $\Delta G^{‡}$. Accordingly, we have revised the manuscript to remove any confusion (see revised manuscript including figures and legends, extended data, and supplementary information).

Point-by-point response to the comments of Reviewer #3

Reviewer #3 co-reviewed with Reviewer #1

Authors: Thank you for your encouraging and constructive co-reviewing with Reviewer 1, which guided us to hone our manuscript. In our response to the comments of Reviewer 1 we describe point-by-point how we have addressed each of the remaining reviewer comments and suggestions.

Point-by-point response to the comments of Reviewer #4

Reviewer 4: As requested by the editor, I had a closer look at the Molecular Dynamics (MD) simulations reported in this manuscript. Overall, my impression is that the simulations have been set-up carefully following established procedures, and the starting structures have been modelled as accurately as possible, given that, unfortunately, no suitable experimental structure for the complex was available. Instead, the authors used available structures of complexes with other receptors as starting structures for their MD-simulations, combined with modelling and AlphaFold for missing residues.

Authors: Thank you for your encouraging and supporting comments. In the following, we explain point-by-point how we addressed each specific comment of the reviewer.

Reviewer 4: While this approach *might* work, it needs to be justified that the modelled complex is sufficiently accurate, i.e., similar enough to the actual (unknown) structure to study, e.g., the reported interaction energies. To this end, the authors write in the methods part: "… literature data show that atomistic MD simulations can model equilibrium structures of proteins and their complexes if their initial structures are reasonable and sufficiently equilibrated". Taken at face value, this is a tautology of course: If the initial structures are sufficiently equilibrated, these *are* equilibrium structures. I guess what the authors actually mean is that if the modelled complex is structurally not too dissimilar from the true (unknown) structure, there is a certain chance that during sufficiently long equilibration runs, the MD structure will converge towards the true one.

Authors: We sincerely thank the reviewer and agree, that the sentence cited may read like every single MD simulation we have performed represents an equilibrium structure. The reorientation of arrestin relative to the receptor is indeed a relatively slow process, as the PCA analysis (included into the new "Supplementary Data" file, Supplementary Data Fig. 11–14) shows. Already in the previously submitted version of our manuscript we have therefore abstained from interpreting single MD simulations and always simulated and analyzed ensembles of MD simulations. If applicable, these ensembles were compared to ensembles from MD simulations conducted under different conditions (*e.g.*, MD simulations of arrestin-receptor complexes in membranes without and with PIP2 or of unphosphorylated and phosphorylated β_2AR). We have removed the potentially misleading sentence from the manuscript and instead explain our ensemble-based approach as the following (see revised Methods, section '*Molecular dynamics (MD) simulations*', subsection '*Complex preparation*':

"To date, no crystal structure of β_2AR in complex with βarr2 has been resolved. In the absence of experimentally determined structures of β_2AR-βarr2 complexes atomistic MD simulations can provide mechanistic insight[4-6] through sampling ensembles of receptor–arrestin conformations, provided that multiple independent replicas are

generated and analyzed comparatively. Under this framework, conclusions can be drawn from differences between conformational ensembles rather than from the interpretation of individual trajectories[7]. Following this ensemble-based strategy, we built 8 models for each β2AR-βarr2 tail, core, and tail-core complex and simulated each system for >1 µs using all-atom MD simulations to obtain conformational ensembles. All simulations are listed in Supplementary Table 1."

Reviewer 4: Of course, there is no guarantee, and nobody knows a priori what 'sufficiently long' means for a particular complex. Hence, this claim needs to be supported by evidence. My main issue here is that such evidence, such as RMSD-plots, PCA plots, or cosine content analyses, are not provided. To be clear, full equilibration can never be proven, but it would be important to see if the used structures at least 'survive' standard tests which prove *lack* convergence.

Authors: Thank you we agree. As stated in our previous answer, we have removed the sentence containing "*sufficiently equilibrated*" from the revised manuscript. Also, in our previous version of the manuscript, we have used the term "equilibration" for our simulations, which could have been confusing. We have replaced this potentially confusing term. Furthermore, please see the Supplementary Data Figures in the new "Supplementary Data" file for plots containing time-resolved simulation-wise RMSD analysis, PCA analysis, as well as the arrestin C-edge and fingerloop binding to the membrane and the arrestin average interaction energy with the membrane for arrestin/membrane systems only. Additionally, the time courses of TM3–TM6 distances for β2AR only systems is included along with analysis of ICL3 residue-wise membrane binding (convergence in 100 ns blocks) and exemplary ensemble-average time courses of membrane interaction probability for two ICL3 residues exhibiting different membrane binding probability under different conditions (receptor phosphorylation and without/with PIP2 in the membrane).

Reviewer 4: I do acknowledge that the structural changes seen in the simulations are intriguing, nicely complement the experiments with (if properly validated) new mechanistic insights, and worth reporting. A major concern here is, however, that only interaction energies were analysed, as opposed to actual free energies. In particular, the reported interaction energies miss the (enthalpic) interactions mediated by the surrounding water molecules, and completely miss all entropic contributions to free energies. As a result, claims such as "Overall, our MD simulations show that membranes with and without PIP2 considerably contribute to the interaction energy stabilizing β2AR-βarr2 tail, core, and tail-core complexes" or "[…] atomistic molecular dynamics (MD) simulations, we are able to characterize the […] thermodynamic properties of the assembled β2AR-βarr2 complexes" (and similar ones) clearly are overinterpretations and need to be properly tuned down.

Authors: Thank you. We agree that interaction energies are not free energies. We have added following statement to our interpretation (see revised Results, section '*Interaction energies reveal PIP_2-dependent membrane stabilization of β2AR-βarr2 complexes*'):

“Absolute interaction energies derived from MD trajectories provide qualitative, force-field–dependent measures of intermolecular stabilization[8] and should not be interpreted as binding free energies. With this limitation in mind, our MD simulations indicate that membranes both without and with PIP_2 substantially contribute to the interaction energy stabilizing the β2AR-βarr2 tail, core, and tail-core complexes. Notably, PIP_2 selectively amplifies the stabilizing effect of the membrane on β2AR-βarr2 complexes relative to other anionic lipids."

Reviewer 4: … or “[…] atomistic molecular dynamics (MD) simulations, we are able to characterize the […] thermodynamic properties of the assembled β2AR-βarr2 complexes” (and similar ones) clearly are overinterpretations and need to be properly tuned down.

Authors: We apologize for the confusion. The word thermodynamic has been removed. Moreover, the characterization of the properties of the assembled β2AR-βarr2 complexes in this sentence related to the experimental data and not to the simulation data. We have revised the section to avoid confusion of the reader.

Reviewer 4: Anticipating the authors’ reply: I know that calculating the free energies that would actually be required to support the above claims is super-difficult to impossible, but that does not justify over-interpretation or insufficient evidence. In their favour, I do agree that their reported probabilities are interesting and *can* allow to derive free energies, but this needs to be done carefully and properly, and does not relieve the authors from sufficiently carefully interpreting their interaction energies.

Authors: Thank you for your understanding that it is not feasible for us to estimate free-energies of the complexes from MD simulations. As stated in our previous response, we have carefully revised the wording and description of our MD results to avoid any overinterpretation of the interaction energies derived (see revised Results section *‘Interaction energies reveal PIP_2-dependent membrane stabilization of β2AR-βarr2 complexes’*):

“Absolute interaction energies derived from MD trajectories provide qualitative, force-field–dependent measures of intermolecular stabilization[8] and should not be interpreted as binding free energies. With this limitation in mind, our MD simulations indicate that membranes both without and with PIP_2 substantially contribute to the interaction energy stabilizing the β2AR-βarr2 tail, core, and tail-core complexes. Notably, PIP_2 selectively amplifies the stabilizing effect of the membrane on β2AR-βarr2 complexes relative to other anionic lipids."

Notably, also in other parts of the manuscript, we never interpret absolute interaction energies, but always relate them to each other.

Reviewer 4: Finally, the conclusion “Collectively, our simulations of β2AR in membranes with and without PIP2 show that at concentrations of PIP2 expected to be present in physiological membranes, it promotes ICL3-membrane interactions

without inducing active conformations of β2AR." seems to assume that any active conformation would actually have been formed within the (still) relatively short simulation time of a few microseconds. If in reality it takes longer (the experimental upper limit seems to be milliseconds), this conclusion is not valid. Hence, the authors need to provide evidence that *in reality* the respective conformational changes actually would occur at microseconds time scales, or the conclusions needs to be tuned down.

Authors: We apologize for this unclear statement, we initiated the MD simulations from the diverse active states of β2AR and we measured whether the condition (C-tail phosphorylation or membrane with PIP_2) change the TM3–TM6 distance in the ensemble of 28 independent simulations initiated from β2AR in 8 different active states. The revised sentence included in the revised Results, section '*PIP_2 attracts ICL3 of β_2AR to membrane but does not promote β_2AR activation'* reads now:

"Collectively, our simulations of β_2AR, embedded in membranes without and with PIP_2, indicate that PIP_2 concentrations found in physiological membranes promote ICL3-membrane interactions without altering the active conformation of β_2AR within simulation times that are sufficient to observe TM3-TM6 distance changes. For the phosphorylated β_2AR C-tail, the C-tail interactions with ICL3 and, in the presence of PIP_2, with ICL2 slightly increase."

References

1 Pacheco, J. *et al.* Fast-diffusing receptor collisions with slow-diffusing peptide ligand assemble the ternary parathyroid hormone-GPCR-arrestin complex. *Nat Commun* **15**, 10499 (2024). https://doi.org/10.1038/s41467-024-54772-3

2 Eichel, K. *et al.* Catalytic activation of beta-arrestin by GPCRs. *Nature* **557**, 381-386 (2018). https://doi.org/10.1038/s41586-018-0079-1

3 Janetzko, J. *et al.* Membrane phosphoinositides regulate GPCR-beta-arrestin complex assembly and dynamics. *Cell* **185**, 4560-4573 e4519 (2022). https://doi.org/10.1016/j.cell.2022.10.018

4 Hempel, T. *et al.* Molecular mechanism of inhibiting the SARS-CoV-2 cell entry facilitator TMPRSS2 with camostat and nafamostat. *Chem Sci* **12**, 983-992 (2021). https://doi.org/10.1039/D0SC05064D

5 Lindorff-Larsen, K. *et al.* Systematic Validation of Protein Force Fields against Experimental Data. *PLOS ONE* **7**, e32131 (2012). https://doi.org/10.1371/journal.pone.0032131

6 Plattner, N., Doerr, S., De Fabritiis, G. & Noé, F. Complete protein–protein association kinetics in atomic detail revealed by molecular dynamics simulations and Markov modelling. *Nat Chem* **9**, 1005-1011 (2017). https://doi.org/10.1038/nchem.2785

7 Dror, R. O. *et al.* Activation mechanism of the beta2-adrenergic receptor. *Proc Natl Acad Sci U S A* **108**, 18684-18689 (2011). https://doi.org/10.1073/pnas.1110499108

8 Mukhaleva, E. *et al.* Cellular lipids regulate the conformational ensembles of the disordered intracellular loop 3 in beta2-adrenergic receptor. *iScience* **27**, 110086 (2024). https://doi.org/10.1016/j.isci.2024.110086

NSMB-A50618B “*Ligands, phosphorylation and PIP$_2$ steer β2-adrenergic receptor–β-arrestin complexes*”

Point-by-point response to the comments of Reviewer #2

Reviewer 2: The authors fully addressed my previous comments.
Congrats for this nice work!

Authors: Thank you for your encouraging and constructive comments, which guided us to improve and revise our work!

NSMB-A50618B "*Ligands, phosphorylation and PIP_2 steer β2-adrenergic receptor–β-arrestin complexes*"

Point-by-point response to the comments of Reviewer #4

Reviewer 4: My main initial concerns were over-interpretations and too strong conclusions drawn from the molecular dynamics simulations that were sufficiently supported by the provided results. In their revision, the authors now provide additional data that helped me and potential readers assess the equilibration of the simulations, which I think for the mostly qualitative conclusions drawn is good enough. Further, there were a few misleading or overly confident statements (e.g., regarding the interaction energies they 'sold' as free energies), which the authors have now carefully tuned down, and I think all these issues have been sufficiently addressed now.

Authors: Thank you for your encouraging and supporting comments, which guided us to revise and improve our work!

Reviewer 4: The only uncertainty remaining is my first comment in the previous round, i.e., whether or not the structure model is accurate enough to draw these conclusions, even if tuned down. After all, it is conceivable that the model is so far off reality that all simulations are stuck in a wrong free energy minimum. Averaging over many trajectories, as advertised in the authors' reply, may help but does not rule out this possibility. The only way to address this issue is to try and calculate observables that can be measured independently and compare. It would be ideal if the authors could provide such a sanity-check against experiment, but I would also be ok with a clear cautionary remark to this effect when discussing their model and its limitations.

Authors: The reviewer recommends that "*Averaging over many trajectories, as advertised in the authors' reply, may help but does not rule out this possibility. The only way to address this issue is to try and calculate observables that can be measured independently and compare. It would be ideal if the authors could provide such a sanity-check against experiment, but I would also be ok with a clear cautionary remark to this effect when discussing their model and its limitations.*" As recommended by the editor handling our manuscript we included a clear cautionary remark to this effect when discussing our model and its limitations. The remark has been included into the methods our manuscript, section '*Molecular dynamics (MD) simulations*'.
